# One- and two-electron coordinatively-induced reduction of *N*-heterocycles by divalent rare earth terphenyl anilide complexes

**DOI:** 10.1039/d6sc03059a

**Published:** 2026-06-09

**Authors:** Ross E. MacKenzie, Benjamin L. L. Réant, Iain J. Cameron, Harry M. Silver, George F. S. Whitehead, Eric J. L. McInnes, Conrad A. P. Goodwin

**Affiliations:** a Centre for Radiochemistry Research, The University of Manchester Oxford Road Manchester M13 9PL UK conrad.goodwin@manchester.ac.uk; b Department of Chemistry, The University of Manchester Oxford Road Manchester M13 9PL UK

## Abstract

Molecular rare-earth (RE) complexes in the divalent oxidation state have been used as potent one-electron reductants, yielding bonding motifs not accessible elsewhere in the periodic table. A deeper understanding of the chemical properties which dictate their reducing power is required to tame their reduction potentials and unlock new controlled reactivity. Here, we report the electrochemical characterisation of a series of divalent *bis*-(tethered)arene complexes, [M^ii^(NHAr^*i*Pr6^)_2_] (1M, M = Y, La, Sm, Eu, Yb, Tm, Lu; NHAr^*i*Pr6^ = {N(H)C_6_H_3_-2,6-(C_6_H_2_-2,4,6-*i*Pr_3_)_2_}), and their reactivity towards the nitrogen heterocycles pyridine and 4,4′-bipyridine. Complexes 1Y, 1La, 1Tm, and 1Lu reduce 4,4′-bipyridine and reductively couple pyridine to afford the dimeric [{M^iii^(NHAr^*i*Pr6^)_2_}_2_(µ-N_2_C_10_H_8_)] (2M, M = Y, La, Tm, Lu) and [{M^iii^(NHAr^*i*Pr6^)_2_}_2_(µ-N_2_C_10_H_10_)] (3M, M = Y, La, Tm, Lu) respectively. These results contrast with electrochemical measurements, which place the RE(iii/ii) reduction potentials for all 1M complexes as insufficiently reducing for either heterocycle. These results imply the reactions proceed through coordination-induced reduction. Synthesis of 2M complexes also results in the formation of 4,4′-bipyridine radical species, which are probed by NMR, EPR, and UV-vis-NIR spectroscopies.

## Abstract 2

Divalent rare earths are potent reductants whose reducing power depends on the coordinating ability of the substrate, and on structural rearrangement during electron transfer.

## Graphical abstract

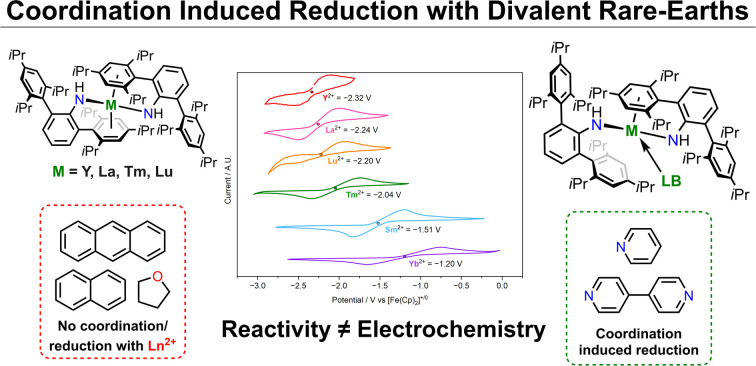

## Introduction

**Fig. 1:**
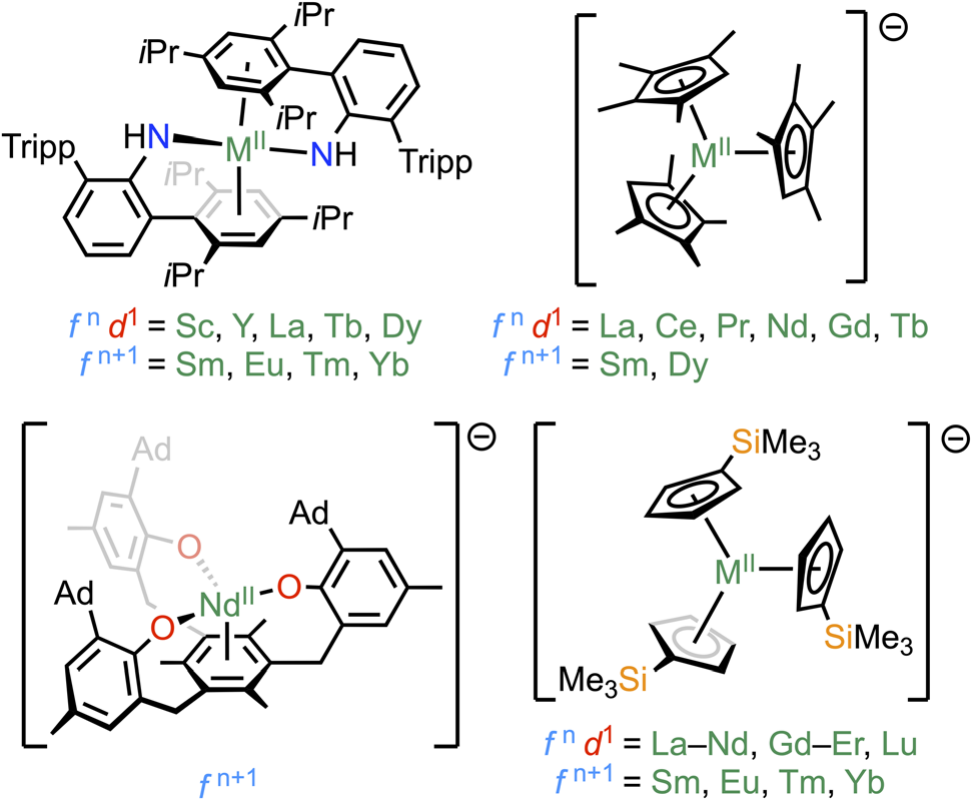
Select examples of RE(ii) complexes showing examples of f^*n*+1^ and f^*n*^ d^1^ valence electron configurations, and how these change depending on the coordination environment.

Fundamental understanding of the redox chemistry of rare-earth (RE) elements has lagged behind that of the transition metals and early actinides. Initial research was limited to Sm(ii) (f^6^), Eu(ii) (f^7^), and Yb(ii) (f^14^) given the increased stability gained from the filled, half-filled, or near half-filled subshells, respectively.^1^ The isolation of a molecular Tm(ii) species in 1997,^2^ followed by Dy(ii),^3^ and Nd(ii),^4^ showed that it was not only the three “classical” divalent rare earths, samarium, europium, and ytterbium, that could yield isolable molecular divalent compounds. Subsequent work has extended the range of known RE(ii) molecules to include the entire series, except for radioactive promethium,^5^ and two distinct electronic structures have emerged: those with f^*n*+1^ electron configurations, or those with f^*n*^ d^1^ configurations (4d^1^ and 3d^1^ for Y and Sc respectively, 5d^1^ for lanthanides, Fig. 1). By-and-large, the former encompasses all Sm(ii), Eu(ii), and Yb(ii) complexes, Tm(ii),^6^ Dy(ii), and Nd(ii)^7–10^ exist at an interface where ligands and coordination geometry direct the ground-state configuration, and all other RE(ii) ions exhibit f^*n*^ d^1^ configurations.^11–25^ Moreover, of those complexes with formal f^*n*^ d^1^ configurations, some have been reported where the highest singly-occupied molecular orbital (SOMO) resembles a {(*n* + 1)s(*n*d_z_^2^)} hybrid atomic orbital (*e.g.* {6s(5d_z_^2^)}),^11–20,26^ and others where the SOMO has significant ligand-character, most commonly with arene rings,^21–25^ which can be used to trap masked low-oxidation state synthons.^27–38^

Owing to their large, negative reduction potentials, divalent rare-earth ions have been used as strong reducing agents capable of performing single-electron transfer (SET) reactions, such as the reduction of N_2_,^39,40^ CO,^41,42^ alkynes,^43,44^ and nitrogenous heterocycles.^45–49^ While the number of divalent rare-earth complexes has grown considerably, electrochemical studies have been limited by the low reduction potentials and high reactivity of these ions. The [RE^iii^(Cp′)_3_] (Cp′ = {C_5_H_4_SiMe_3_}) motif is known for all RE elements save scandium and promethium,^8,11,14–16,50^ which makes it ideal to explore trends in the RE(iii/ii) reduction potential (*E*_1/2_) across these elements.^51^ Across the lanthanide members in this series, the reduction potentials (*vs.* [Fe(Cp)_2_]^+/0^, Cp = {C_5_H_5_}) for the 4f^*n*+1^ ions follow the expected trend based on the stability of their electronic configurations:^1^ Eu(ii) 4f^7^ (−1.07 V) > Yb(ii) 4f^14^ (−1.64 V) > Sm(ii) 4f^6^ (−2.41 V) > Tm(ii) 4f^13^ (−2.83 V), while the non-traditional 4f^*n*^ 5d^1^ ions exhibit more negative reduction potentials that fall within a narrow range (−2.95 V to −3.14 V). This is due to their electronic structures, where the highest-occupied molecular orbital is largely non-bonding, resembling a {6s(5d_z_^2^)} hybrid atomic orbital oriented perpendicular to the molecular *C*_3_-axis. As such, the ligands, which remain constant across the series, define the splitting of the d-orbital manifold, and hence the energy and reducing power of the configuration, so the RE identity has little influence on the reduction potential for these non-traditional RE(ii) ions.

We recently reported [M^ii^(NHAr^*i*Pr6^)_2_] (M = Sc, Y, La, Sm, Eu, Tm, Yb; NHAr^*i*Pr6^ = {NHC_6_H_3_-2,6-Tripp_2_}, Tripp = {C_6_H_2_-2,4,6-*i*Pr_3_}) as a series of neutral, room-temperature-stable, divalent rare-earth complexes covering both the 4f^*n*+1^ (Sm, Eu, Tm, and Yb) and f^*n*^ d^1^ (Sc, Y, and La where *n* = 0) configurations.^25^ In the case of Sc(ii), Y(ii), and La(ii), significant metal–ligand orbital mixing between flanking arene groups and the metal centre gives rise to a one-electron δ-bonding interaction.^25^ The balance of metal and ligand character in this interaction varies with the metal identity, unlike in RE(ii) complexes with a non-bonding {*n*s(*n*−1d)} configuration, which means the reducing power of these complexes might be modulated by the metal identity – but this concept remains unexplored at present.

Herein, we present an electrochemical study of the [M^ii^(NHAr^*i*Pr6^)_2_] (1M, M = Y, La, Sm, Eu, Tm, Yb, Lu) complexes, and their reactivity towards two *N*-heterocycles – 4,4′-bipyridine and pyridine – which have well-defined one- and two-electron redox chemistry in order to quantify their chemical reducing power alongside electrochemical measurements. Well-defined quasi-reversible RE(iii/ii) couples are found with six of the seven complexes, with 1Eu showing no redox events by cyclic voltammetry. The most reducing examples, (1Y, 1La, 1Lu, and 1Tm) reduce 4,4′-bipyridine to its dianion in bimetallic [{M^iii^(NHAr^*i*Pr6^)_2_}_2_(µ-N_2_C_10_H_8_)] (2M, M = Y, La, Tm, Lu), and also reduce pyridine to a radical which subsequently undergoes C_(sp^3^)_–C_(sp^3^)_ coupling to give bimetallic [{M^iii^(NHAr^*i*Pr6^)_2_}_2_(µ-N_2_C_10_H_10_)] (3M, M = Y, La, Tm, Lu). The less reducing examples (1Sm, 1Eu, and 1Yb) do not reduce either heterocycle, and spectroscopic investigations (UV-vis-NIR, NMR) suggest that the metal centres do not coordinate to the heterocycles in solution with these metals.

## Results

### Synthesis and electrochemistry

The divalent complexes 1M (M = Y, La, Sm, Eu, Tm, Yb, Lu) were prepared by the KC_8_-mediated reduction of trivalent iodide precursors, [M^iii^(NHAr^*i*Pr6^)_2_(I)], or directly from divalent metal iodides, using methods described previously.^25^ Cyclic voltammetry was performed on all 1M complexes (M = Y, La, Sm, Eu, Tm, Yb, Lu) under an argon atmosphere as 10 mM solutions in THF with [*n*Bu_4_N][BPh_4_] (50 mM) as the electrolyte with a glassy carbon working electrode, platinum wire counter electrode, and a Ag/AgCl pseudo-reference electrode. All data is reported with reference to the [Fc]^+/0^ couple (Fc = [Fe(Cp)_2_]; Cp = {C_5_H_5_}). Solutions of 1M are unstable in the presence of Fc, though we have not been able to characterise the products of the reaction. Therefore, the [Fc*]^+/0^ (Fc* = [Fe(Cp*)_2_]; Cp* = {C_5_Me_5_}) couple was used instead to provide an internal reference standard for all measurements, which was then calibrated to the [Fc]^+/0^ couple.^52–55^ The use of an internal standard is essential to the reliable determination of the reduction potential of these species. The SI (Section 7) contains full scans used for referencing each complex.

**Fig. 2:**
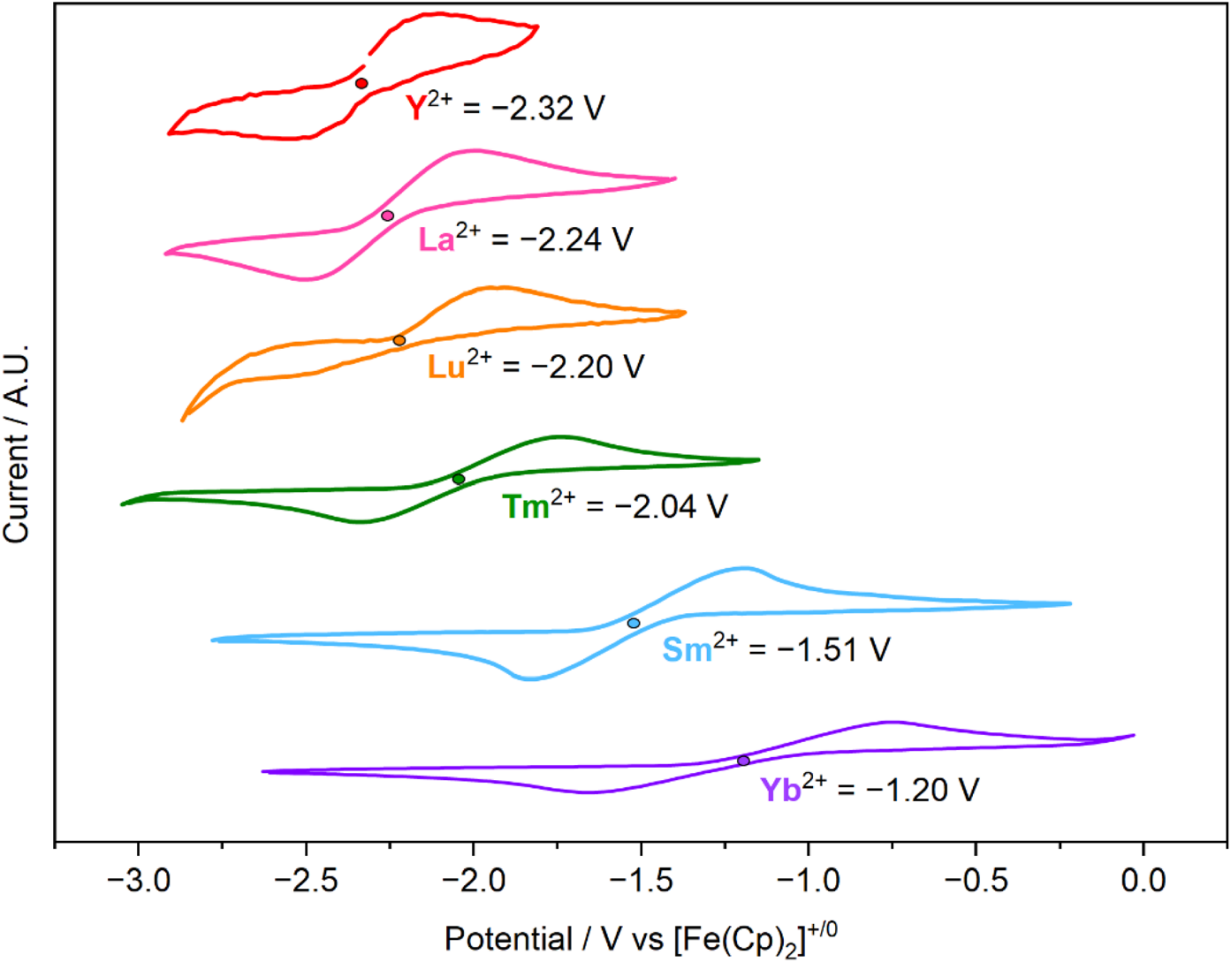
Cyclic voltammograms of 1M (M = Y, La, Lu, Tm, Sm, Yb) in THF (10 mM) supported by [*n*Bu_4_N][BPh_4_] (50 mM) *vs.* [Fc]^+/0^ at 200 mV s^−1^.

Cyclic voltammograms for 1Sm, 1Tm, and 1Yb give *E*_1/2_ values for the [M(NHAr^*i*Pr6^)_2_]^+/0^ couple that correlate well with the standard reduction potentials for each ion,^56^ in order from least to most cathodic: 1Yb (−1.20 V) > 1Sm (−1.51 V) > 1Tm (−2.04 V). The *E*_1/2_ values for the complexes with formal 4f^*n*^ 5d^1^ configurations fall within a small range: 1Y (−2.32 V; 4d^1^), 1La (−2.24 V), and 1Lu (−2.20 V), but are all somewhat more cathodic than those with 4f^*n*+1^ configurations. The trend is analogous to that of the [RE^iii^(Cp′)_3_] series;^51^ however, in every case, *E*_1/2_ for the RE(iii/ii) couple in the 1M series is more anodic. A summary of the RE(iii/ii) potentials is given in Table 1, and in Fig. 2 shows scans for each 1M complex (save 1Eu). In the case of 1Eu, no redox events were observed that could be definitively assigned to 1Eu, despite repeated attempts under both light and dark conditions. An irreversible oxidation wave could be seen at a moderately anodic potential (+0.14 V *vs.* [Fc]^+/0^), which overlapped with the oxidation wave of our electrochemical standard (Fc*; see Fig. S119).

**Table 1:** Summary of electrochemical data for 1M (M = Y, La, Lu, Tm, Sm, Yb). Potentials given *vs.* [Fc]^+/0^, and all data derived from scans at 200 mV s^−1^

Complex	*E*_1/2_ [M^ii^(NHAr^*i*Pr6^)_2_]^+/0^ (V)	*E*_pa_ (V)	*E*_pc_ (V)	Δ*E*_p_ (mV)
1Y	−2.32	−2.19	−2.45	260
1La	−2.24	−1.95	−2.53	580
1Lu	−2.20	−2.08	−2.32	240
1Tm	−2.04	−1.84	−2.24	400
1Sm	−1.51	−1.34	−1.68	340
1Yb	−1.20	−0.87	−1.53	660

For each 1M complex (save 1Eu), a second quasi-reversible reduction event was observed at more negative potentials (from −2.60 V to −3.76 V, see Fig. S50), the order of which is roughly the inverse of the trend in their M(iii/ii) potentials – *i.e.* the second event in 1Yb is the most cathodic, and with 1Y, 1La, and 1Lu, it is the most anodic.

### Spectroscopic assessment of reducing power in solution

**Scheme 1:**
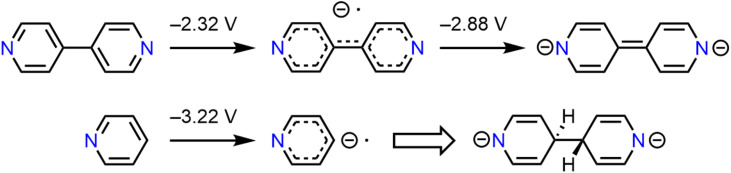
Reduction potentials for 4,4′-bipyridine and pyridine *versus* the [Fc]^+/0^ couple, and see Fig. S122–S125 for measurements.^57–60^

The nitrogen heterocycles 4,4′-bipyridine and pyridine have one- and two-electron (−2.32 V, −2.88 V), and one-electron (−3.22 V), reduction potentials,^57–60^ respectively (Scheme 1), that are close to, or more negative than, the M(iii/ii) couples in the 1M complexes herein (range: −1.20 V to −2.32 V). We first used NMR spectroscopy and UV-vis-NIR titration studies to assess reactions between solutions of 1M (M = Y, La, Sm, Eu, Tm, Yb, Lu) and both pyridine and 4,4′-bipyridine.

^1^H NMR spectra of diamagnetic 1Yb and 4,4′-bipyridine or pyridine in *d*_6_-benzene showed no change compared to that of 1Yb (Fig. S133–135) The paramagnetic nature of 1Sm and 1Eu renders their ^1^H NMR spectra largely uninformative; however, *d*_6_-benzene solutions of either 1Sm or 1Eu in the presence of 4,4′-bipyridine or pyridine showed no change to their NMR spectra (see Table S10), which suggests their oxidation states remain unchanged. UV-vis-NIR spectra were collected for all three complexes (Fig. S136–S138) in toluene in the presence of 4,4′-bipyridine or pyridine. These spectra showed no significant differences compared to those of isolated 1Sm, 1Eu, or 1Yb. Together, these data indicate that 4,4′-bipyridine and pyridine are not reduced by these complexes and are unlikely to coordinate to the metal centre.

Titration of 4,4′-bipyridine into *d*_6_-benzene solutions of 1Y, 1La, or 1Lu, gave diamagnetic ^1^H NMR signals consistent with the 4,4′-bipyridine dianion at *ca*. 5.8 ppm and 5.6 ppm (^3^*J*_HH_ = 7 Hz for both).^49,61^ With 1Y and 1Lu, the intensity of these resonances decreased rapidly as the concentration of 4,4′-bipyridine was increased. Once more than 0.5 equivalents were added, new signals appeared at *δ*_H_ = 8.56 and 6.81 ppm, corresponding to neutral 4,4′-bipyridine. In the case of 1La, signals from the 4,4′-bipyridine dianion do not disappear with increased quantities of 4,4′-bipyridine dianion, but neutral 4,4′-bipyridine was observed (see Fig. S139–S141).

**Fig. 3:**
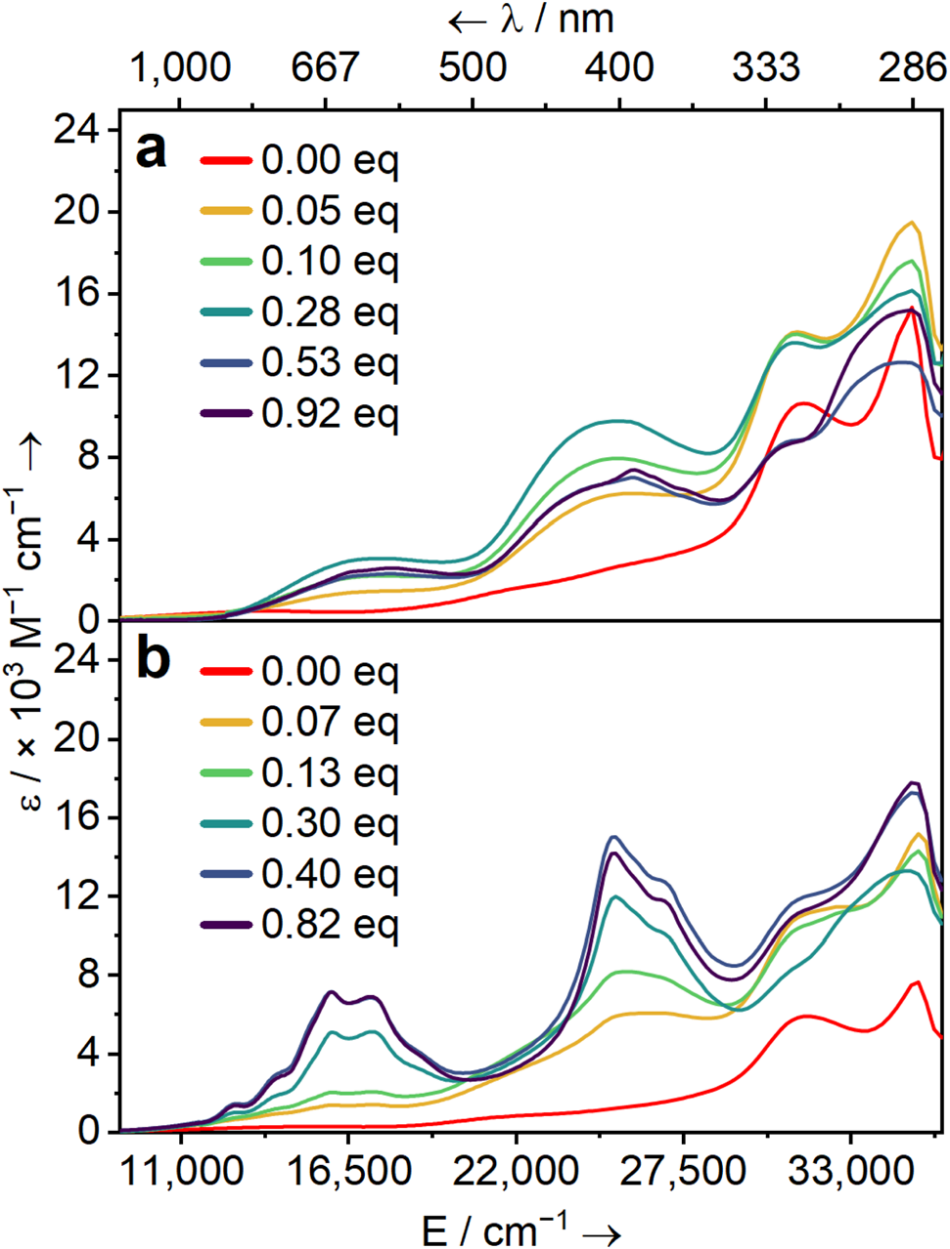
UV-vis-NIR spectra for titration experiments on 1La (a) and 1Lu (b). Each line denotes the number of equivalents of 4,4′-bipyridine added to the solution of 1La or 1Lu.

UV-vis-NIR titration experiments were performed on 1M (M = Y, La, Tm, Lu) in toluene, ranging from 0 to >0.8 equivalents of 4,4′-bipyridine (Fig. 3). Starting with 1La, at low concentrations of 4,4′-bipyridine, the UV-vis-NIR spectrum shows the appearance of strong broad absorbances at 408 nm (24 500 cm^−1^, *ε* = 7.28 × 10^3^ M^−1^ cm^−1^) and 600 nm (16 700 cm^−1^, *ε* = 1.76 × 10^3^ M^−1^ cm^−1^), which increase in intensity as the concentration of 4,4′-bipyridine increases up to *ca*. 0.5 equivalents, before decreasing beyond this. With 1Y and 1Tm (Fig. S149–S152), UV-vis-NIR spectra after the addition of 0.1 equivalents of 4,4′-bipyridine are similar to those of 1La – showing broad absorbances at 382 nm (24 500 cm^−1^: 1Y*ε* = 8.9 × 10^3^ M^−1^ cm^−1^, 1Tm*ε* = 12.6 × 10^3^ M^−1^ cm^−1^) and 610 nm (16 393 cm^−1^: 1Y*ε* = 3.0 × 10^3^ M^−1^ cm^−1^, 1Tm*ε* = 5.1 × 10^3^ M^−1^ cm^−1^). However, higher concentrations (>0.3 equivalents) of 4,4′-bipyridine cause the features to blue-shift slightly, and the appearance of fine structure in the low-energy region in the spectra for both complexes, which is resolved into two broad peaks (586 nm, 17 065 cm^−1^ and 632 nm, 15 823 cm^−1^). In the case of 1Lu, the fine structure was apparent in all spectra, even at the lowest concentrations of 4,4′-bipyridine. Solutions of 1Y, 1La, 1Tm, and 1Lu are green in the presence of low concentrations of 4,4′-bipyridine, but at higher concentrations, those with 1Y, 1Tm, and 1Lu turn blue. These spectra are all similar to that of the 4,4′-bipyridinyl radical anion and the 4,4′-bipyridine dianion,^62–66^ and to an organoboron compound featuring a 4,4′-bipyridine core in three oxidation states,^61^ but are different to several other metal complexes featuring 4,4′-bipyridine anions.^49,67^

The addition of pyridine to solutions of the 1M complexes (M = Y, La, Tm, Lu) resulted in rapid, irreversible colour changes from dark red/green to yellow in all cases.

It is evident from ^1^H NMR and UV-vis-NIR titration data that 1Y, 1La, 1Tm, and 1Lu react with 4,4′-bipyridine to form a product containing the 4,4′-bipyridine dianion, but, except for 1La, each reacts further to give another species that forms even at low concentrations of 4,4′-bipyridine. While the reduction potentials of 4,4′-bipyridine and pyridine are more negative than the [naphthalene]^0/−^ couple (−2.2 V *vs.* [Fc]^+/0^),^68^ and other RE(ii) complexes reduce a range of neutral aromatic hydrocarbons,^3,69–77^ no reaction was observed between 1M (M = Y, La, Lu) and naphthalene or anthracene (−1.9 V *vs*. [Fc]^+/0^)^68^ by NMR spectroscopy (see Fig. S142–S147).

### Preparative-scale reactions with 4,4′-bipyridine and pyridine

**Scheme 2:**
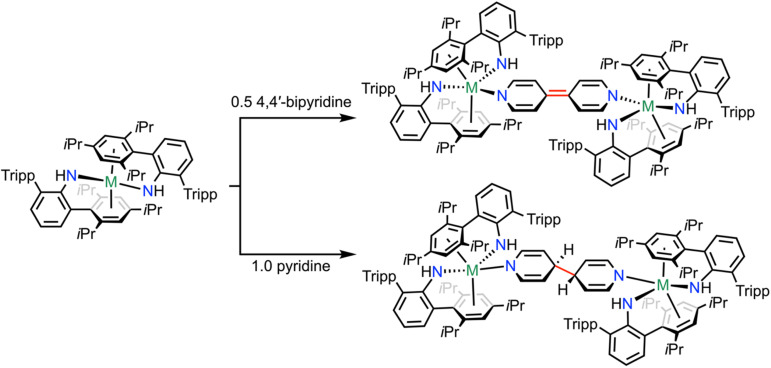
The synthesis of 2M (M = Y, La, Tm, Lu) and 3M (M = Y, La, Tm, Lu) complexes, from the reduction of 4,4′-bipyridine or pyridine with 1M (M = Y, La, Tm, Lu) complexes.

The reaction between two equivalents of 1M (M = Y, La, Tm, Lu) and one equivalent of 4,4′-bipyridine in toluene resulted in a rapid colour change from dark red/green to intense royal blue (M = Y, Tm, Lu) or dark green (M = La). Work-up and crystallisation from *n*-hexane gave the bimetallic [{M^iii^(NHAr^*i*Pr6^)_2_}_2_(µ-N_2_C_10_H_8_)] (2M, M = Y, La, Tm, Lu) complexes in poor to fair isolated crystalline yields (25–48%) (Scheme 2). Similarly, the addition of pyridine to a toluene solution containing an equimolar amount of 1M (M = Y, La, Tm, Lu) produced an immediate colour change from dark red to yellow. Workup and crystallisation from *n*-hexane gave bimetallic [{M^iii^(NHAr^*i*Pr6^)_2_}_2_(µ-N_2_C_10_H_10_)] (3M, M = Y, La, Tm, Lu) complexes in poor to fair crystalline yields (22–52%) (Scheme 2). Similar reactions with 1Sm, 1Eu, and 1Yb gave the starting materials unchanged.

^1^H NMR spectra of isolated 2M (M = Y, La, Lu) in *d*_6_-benzene show they are diamagnetic, exhibiting two resonances at *δ*_H_ = 5.8 (*d*, 4H, ^3^*J*_HH_ = 7.3 Hz) and 5.6 (*d*, 4H, ^3^*J*_HH_ = 7.5 Hz) corresponding to the 2,2′,6,6′ and 3,3′,5,5′ protons of the 4,4′-bipyridyl dianion unit respectively, and are therefore twofold symmetric in solution (Fig. S10–S19). The resonances are shifted upfield relative to those of neutral 4,4′-bipyridine, suggesting reduction and a loss of aromaticity (Scheme 1). For 2Tm, the solution-state molar magnetic susceptibility was found to be 6.07 µ_B_ (using the Evans method), somewhat lower than the typical range for Tm(iii) (7.1–7.5 µ_B_), but consistent with previous observations of dimeric Tm(iii) complexes [{Tm^iii^(C_4_H_10_O_2_)(*η*^2^-C_10_H_8_)}_2_(*η*^4^-C_10_H_8_)] (6.59 µ_B_) and [{Tm^iii^(I)_2_(C_5_H_5_N)_4_}_2_(µ-N_2_C_10_H_10_)] (6.05 µ_B_).^78,79^ The ^1^H NMR spectra for 3Y, 3La, and 3Lu are consistent with diamagnetic compounds, with three resonances at *δ*_H_ = 6.0 (s, 4H), 4.6 (m, 4H), 3.4 (s, 2H), consistent with single electron transfer from the metal centre to the pyridine substituent, followed by C–C coupling at the para position (Fig. S20–S28).

The solution molar magnetic susceptibility for 3Tm (7.5 µ_B_) is in excellent agreement with the theoretical value for two non-interacting Tm(iii) ions. Solution UV-vis-NIR spectra for isolated 2M (M = Y, La, Tm, Lu) show similar features to the titration studies, whereby 2La shows two broad features without fine structure, which is consistent with the 4,4′-bipyridyl dianion, whereas the spectra of 2Y, 2Tm, and 2Lu all show additional features that resemble the 4,4′-bipyridyl radical anion (Fig. S37–S40).^61–64^ Solid-state UV-vis-NIR spectra of all four complexes showed no significant differences (Fig. S41–S44). Solution UV-vis-NIR spectra of all 3M (M = Y, La, Tm, Lu) complexes are largely featureless, with an intense band extending from the UV region to *ca*. 20 000 cm^−1^, which accounts for their strong yellow colouration. The spectrum of 3Tm also features two sharp but weak absorbances at 12 797 cm^−1^ (781 nm, *ε* = 15 M^−1^ cm^−1^) and 14 701 cm^−1^ (680 nm, *ε* = 7 M^−1^ cm^−1^) corresponding to the ^3^H_6_ → ^3^F_4_ and ^3^H_6_ → ^3^F_2/3_ transitions typical for a Tm(iii) ion.^80^

### Molecular structures

**Fig. 4:**
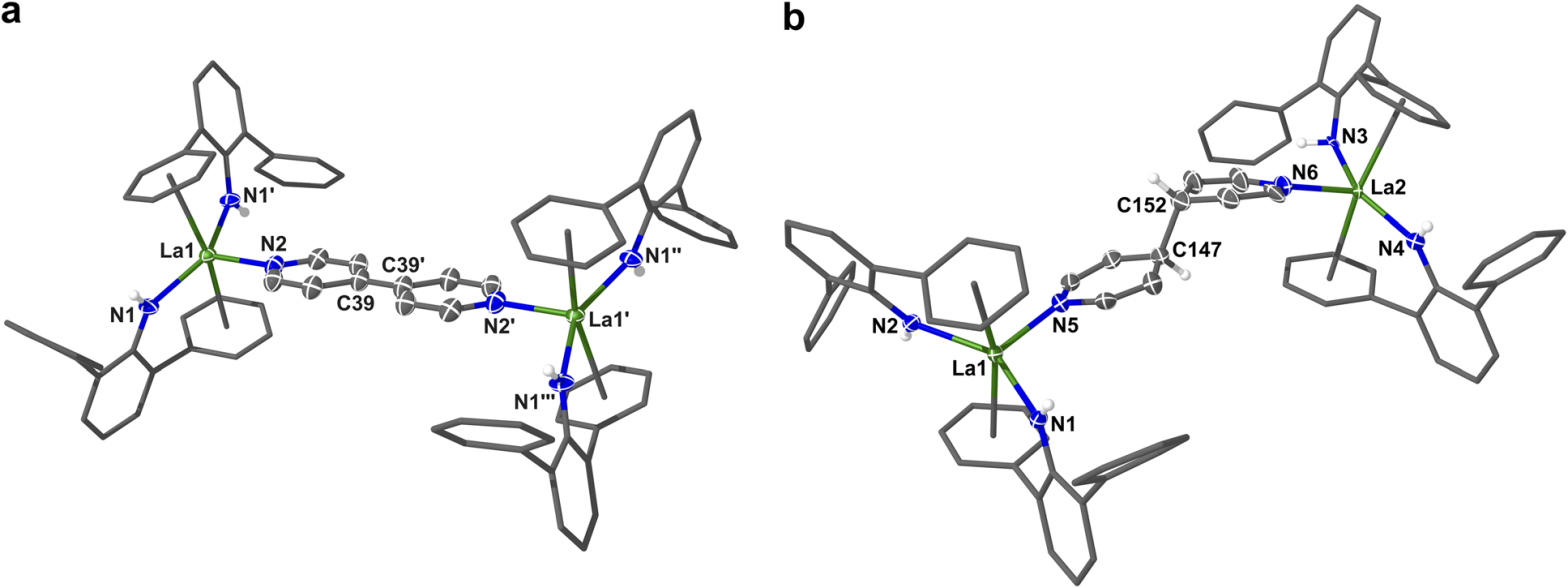
Molecular structures of (a) [{La^iii^(NHAr^*i*Pr6^)_2_}_2_(µ-N_2_C_10_H_8_)] (2La) and (b) [{La^iii^(NHAr^*i*Pr6^)_2_}_2_(µ-N_2_C_10_H_10_)] (3La). Ellipsoids set at 30% probability. H-atoms except those on N–H groups are omitted for clarity, along with lattice solvents and ligand isopropyl groups.

Single-crystal X-ray diffraction studies of all 2M and 3M complexes reveal that they are pseudo-3-coordinate, considering only the anionic donors. The structures of 2La and 3La are shown in Fig. 4 as examples; see the SI (Section 2) for all others.

Complexes 2Y and 2Lu crystalise in the monoclinic space group *P*2_1_/*c* (*Z*′ = 0.5) with an inversion through the central C–C bond of the bipyridyl unit. Complex 2Tm crystalised in the orthorhombic space group *Cmc*2_1_ (*Z*′ = 0.5), and 2La crystalised in the orthorhombic *Cmce* space group with one quarter of the dimeric molecule per asymmetric unit (*Z*′ = 0.25). The most notable feature of 2M complexes is the short central C–C bond length (2Y, 1.355(18) Å; 2La, 1.389(8) Å; 2Tm, 1.323(11) Å; 2Lu, 1.368(4) Å) that are consistent with a C–C double bond and suggest the reduction of 4,4′-bipyridine to its dianion (Scheme 1), consistent with the upfield shift for the bipyridyl protons in the ^1^H NMR spectra.^49,61,81,82^ The M–N_bipy_ bond lengths (2Y, 2.211(7) Å; 2La, 2.336(3) Å; 2Tm, 2.174(5) Å; 2Lu, 2.1613(16) Å) are considerably shorter than those of neutral 4,4′-bipyridine adducts to these metals,^83–88^ and are typical for M–N distances to anionic N-donors. In the solid state, the La-centre in 2La retains coordination to two Tripp substituents, whereas for 2Y, 2Tm, and 2Lu only one remains bound to accommodate the 4,4′-bipyridine unit. However, the ^1^H NMR spectra for all show that this interaction is fluxional on the NMR timescale.

Complexes 3Y and 3Lu crystallised in the monoclinic space groups *P*2_1_/*n* and *P*2_1_ (*Z*′ = 1) respectively, whereas 3La and 3Tm crystallised in the triclinic space group *P*1̄ (*Z*′ = 1). The structural data confirm 3M complexes possess a new coupled C–C single bond (lengths: 3Y, 1.564(5) Å; 3La, 1.558(11) Å; 3Tm, 1.560(4) Å; 3Lu, 1.541(7) Å) at the para position of the pyridine moieties. The two M–N_py_ bond distances are statistically indistinguishable from one another, but are shorter than neutral pyridine adducts with these metals, suggesting they are anionic N-donors.^89–91^ As with the 2M complexes, 3Y, 3Tm, and 3Lu each have one bound Tripp ring, while 3La maintains close contact with two Tripp rings. In 3Y, 3Tm, and 3Lu, the twist about the central C–C single bond is small (N⋯C–C⋯N torsion angle = 3Y, 59.0(3)°; 3Tm, 63.1(4)°; 3Lu, 60.9(6)°), whereas in 3La the two pyridyl units adopt a stepped configuration (N⋯C–C⋯N torsion angle = 173.9(14)°).

### Radical formation in 2M complexes

Although 2M (M = Y, La, Lu) are diamagnetic (see above), frozen-solution and powder electron paramagnetic resonance (EPR) spectroscopy measurements gave signals consistent with the presence of radical species (see Fig. S153–S162). The signals observed in the solution spectra of 2La and 2Tm were very weak and significantly weaker than those for 2Y and 2Lu. In the case of 2La, this observation aligns with the UV-vis-NIR spectra (Fig. S37–S40) of the isolated materials, which show a lower radical content, whereas for 2Tm, it does not. These spectra have poor resolution; that of 2Y has partially resolved structure, which we have been unable to model assuming a single species, hence it is likely due to small amounts of an oxidised form of 2M together with dissociation products.

**Fig. 5:**
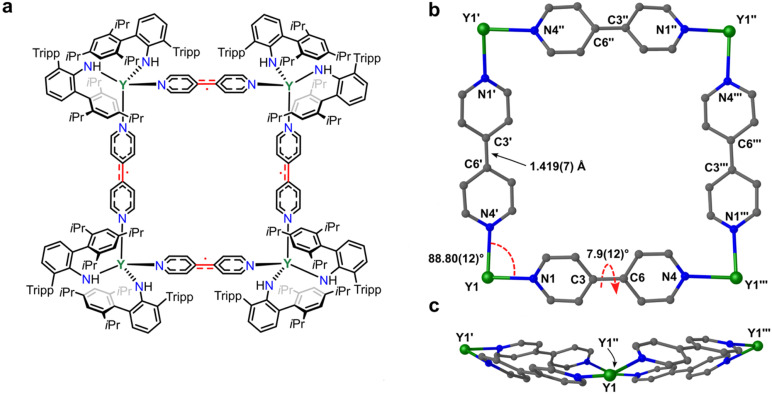
(a) Schematic of complex 4 showing 4,4′-bipyridine radical bridges; (b) square-core of 4Y showing only the Y atoms and 4,4′-bipyridine bridges with select distances and angles; (c) view of 4 along the Y1⋯Y1″ direction, showing the puckered conformation of the square-core in 4.

On one occasion during the preparation of 2Y using an excess of 4,4′-bipyridine, several blue-purple crystals of a tetrameric complex, [{Y^iii^(NHAr^*i*Pr6^)_2_(µ-N_2_C_10_H_10_)}_4_] (4) were isolated – Fig. 5 shows the molecular structure (see Fig. S9 for full structural parameters). Complex 4 crystallised in the cubic space group *I*4̄3*d* (*Z*′ = 1/4). Each Y-centre is 4-coordinate and adopts a pseudo-seesaw geometry coordinating to two terphenyl anilide ligands and two 4,4′-bipyridinyl radicals. The N_bipy_–Y–N_bipy_ angles of 88.67(15)° result in a twisted square arrangement of the four Y-atoms, which is significantly distorted from planarity (Fig. 5c). The Y–N_bipy_ bond lengths (2.389(4) Å and 2.377(4) Å) are longer than in 2Y (2.211(7) Å) by an amount larger than the difference in formal coordination number would suggest (*ca*. 0.05),^92^ though both are significantly shorter than those with neutral 4,4′-bipyridine adducts.^84,86,93^ The central C–C bond of the 4,4′-bipyridine moieties of 1.419(7) Å is longer than in 2Y (1.355(18) Å), and the torsion angle between the two pyridyl units (7.8(2)°) is consistent with each 4,4′-bipyridine unit being singly reduced to its radical form. Therefore, complex 4 possesses four trivalent yttrium centres bridged by four 4,4′-bypridyl radicals.

The targeted synthesis of complex 4 on a larger scale was attempted through two routes: (i) the stoichiometric reaction between 1Y and 4,4′-bipyridine; and (ii) the reaction between 2Y and an additional equivalent of 4,4′-bipyridine. Both reactions produced intractable mixtures, and crystals suitable for SCXRD could not be grown from toluene, mesitylene, or Et_2_O from −30 °C to room temperature.

## Discussion

**Scheme 3:**
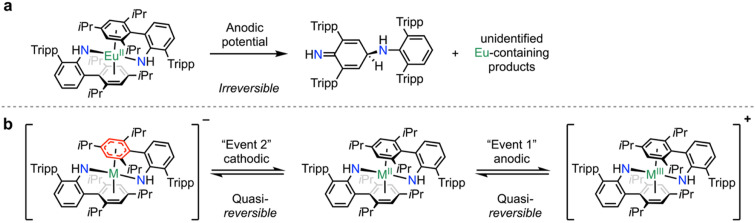
Illustrations of the observed electrochemical processes seen with: (a) 1Eu; and (b) other 1M complexes (M = Y, La, Sm, Tm, Yb). The red {Tripp} ring indicates that the partitioning of charge between the metal centre and the arene ring is not known. Limiting ionic bonding descriptors: M(iii) with {Tripp}^2−^ or M(ii) with {Tripp}˙^−^ are possible, with the former resembling the case of [Ti^iv^(NHAr^*i*Pr6^)_2_].^94^

The measured M(iii/ii) reduction potentials of the 1M (M = Y, La, Sm, Tm, Yb, Lu) complexes herein are all more anodic – the complexes are less reducing – than those of the [RE^ii^(Cp′)_3_]^−^ series,^51^ which reflects the differences in their electronic structure. No clear redox behaviour could be observed with 1Eu save for an irreversible oxidation wave at anodic potentials. We suggest this is because the Eu(ii) ion is significantly stabilised within the {M^ii^(NHAr^*i*Pr6^)_2_} framework, and that ligand oxidation occurs at a more cathodic potential than metal oxidation. Some of us have previously reported the head-to-tail coupling of {NHAr^*i*Pr6^} units during the attempted synthesis of [Yb^iii^(NHAr^*i*Pr6^)_2_(I)] by salt elimination between KNHAr^*i*Pr6^ and [Yb^iii^(I)_3_(THF)_3_], which instead resulted in the Yb(iii) centre acting as an oxidant, giving HNC_6_H_3_-2,6-(2,4,6-*i*Pr_3_)-4-{HN=C_6_H_3_-2,6-(2,4,6-*i*Pr_3_)_2_.^25^ Given that the standard reduction potential for Yb(iii/ii) is more cathodic than that of Eu(iii/ii) – *i.e.* Eu(iii) is more oxidising than Yb(iii) – it follows that the electrochemical generation of Eu(iii) could result in ligand oxidation here (Scheme 3a).

In 1Y^24,25^ and 1La,^25^ computational and spectroscopic data show the unpaired electron resides in a singly occupied molecular orbital (SOMO) resembling a δ-bond between the metal and flanking Tripp rings, whereas in divalent *tris*-Cp′ complexes, the SOMO resembles a non-bonding {(*n* + 1)s(*n*d_z_^2^)} hybrid atomic orbital. The bonding interaction stabilises the SOMO, which is reflected in the reduction potentials of 1Y, 1La, and 1Lu. In the case of the Sm(iii/ii), Tm(iii/ii), and Yb(iii/ii) couples in 1M and the tris-Cp′ complexes, both series follow the trend of their standard reduction potentials, but again, the 1M complexes are less reducing. In this case, it reflects the fact that the 1M complexes are neutral, whereas the [RE^ii^(Cp′)_3_]^−^ complexes are electron-rich anions.^51^

Additional reduction events were found in cyclic voltammograms of the 1M complexes (save 1Eu), with potentials that trend opposite to the M(III/II) couples – *i.e.* the second event with 1Yb is the most cathodic, and with 1Y it is the most anodic. This order correlates with the f → d promotion energy for these ions (where applicable),^95,96^ and we propose that it involves the reduction of one of the flanking Tripp groups, which subsequently forms a δ-bond to the metal centre *via* metal d-orbitals (Scheme 3b). In the case of 1Y, 1La, and 1Lu, this could involve the formation of a closed-shell {Tripp}^2−^ group, similar to the Ti(iv) complex [Ti^iv^(NHAr^*i*Pr6^)_2_].^94^ With 1Sm, 1Tm, and 1Yb, the vacant metal-based orbitals with appropriate symmetry are much higher in energy.

The data reveal an apparent incongruity between the measured reduction potentials and the observed chemical reducing power of the 1M series. Complexes 1Y, 1La, and 1Lu appear sufficiently reducing to access the [naphthalene]^0/−^ (*E*_1/2_ = −2.2 V *vs.* [Fc]^+/0^) and [anthracene]^0/−^ (*E*_1/2_ = −1.9 V *vs.* [Fc]^+/0^) couples,^68^ but no reaction was observed with either (Fig. S142–S147); and none of the 1M complexes show M(iii/ii) couples sufficiently negative for the reduction of pyridine or the double-reduction of 4,4′-bipyridine – by cyclic voltammetry (Fig. 2 and Scheme 1) – yet several are competent in reductions of the latter two *N*-heterocycles. Some of us have previously shown that small changes in the M⋯Tripp distances in 1Y led to large differences in the degree to which the SOMO is localised on the metal, with larger distances increasing the metal localisation.^25^ Furthermore, these data also suggested that, at least in the case of 1Y, the metal centre does not coordinate 2-MeTHF.^25^ While our electrochemical measurements were performed in THF, these complexes may also be crystallised in the presence of THF without apparent coordination.^24^ Therefore, we suggest that the measured reduction potentials for 1M likely correspond to the structures in solution where the {M^ii^(Tripp)_2_} core is unperturbed and similar to the structures from SCXRD.

**Scheme 4:**
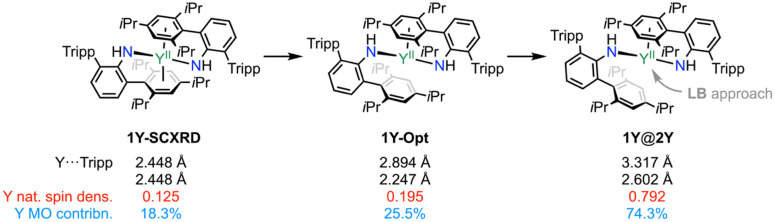
Displacement of the {M^ii^(Tripp)_2_} core by a strong Lewis base increases metal spin density and contribution to the SOMO, and ultimately results in coordination-induced reduction of the Lewis base. 1Y-SCXRD refers to coordinates from the SXRD study of 1Y with the H-atom positions optimised. 1Y-Opt refers to the geometry obtained after optimising 1Y using the coordinates for 1La as a starting point, and it resides at a local minimum by vibrational analysis.^25^1Y@2Y refers to the coordinates from the {Y(Tripp)_2_} unit in 2Y, with the rest of the molecule removed, and the H-atom positions optimised.

*Ab initio* calculations on 1Y using three different geometries: (i) coordinates derived from the SCXRD structures with H-atom positions optimised (1Y-SCXRD);^25^ (ii) using coordinates optimised starting with the geometry of 1La, such that a single Y⋯Tripp interaction remains (1Y-Opt);^25^ (iii) using the coordinates for one half of 2Y with the {4,4′-bipyridine}^2−^ removed (1Y@2Y; see SI Section 11 and Table S16). These revealed significant increases in the natural spin density at the metal,^97^ and in the metal character of the SOMO as the {Y^ii^(Tripp)_2_} core is progressively disrupted. This is shown schematically in Scheme 4, and accounts for the coordination-induced reduction seen in the 2M and 3M series.

Here, we suggest that the change in the {M^ii^(Tripp)_2_} shown above not only alters the distribution of spin in the complexes, but also the M(iii/ii) reduction potential relative to the case in which the {M^ii^(Tripp)_2_} core remains intact. Some 1M complexes have already been shown to reduce P_4_ to (P_4_)^2−^ (reduction potential reported between −1.53 and −1.98 V),^98,99^ and cleave *t*BuNC to give (CN)^−^.^24^ In both cases, the reduced small molecules have heteroatom lone pairs that may coordinate to the metal centre, thereby disrupting the {M^ii^(Tripp)_2_} core in the 1M complexes. In the presence of reducible Lewis-basic molecules such as 4,4′-bipyridine and pyridine, the {M^ii^(Tripp)_2_} core is disrupted, which increases the degree of metal-localisation in the SOMO to the point where electron transfer occurs.

The reduction of pyridine by 1Y, 1La, 1Tm, and 1Lu to give [{M^iii^(NHAr^*i*Pr6^)_2_}_2_(µ-N_2_C_10_H_10_)] (3M, M = Y, La, Tm, Lu) is similar to that of other formally low oxidation state complexes with electropositive metals,^46–48^ or low oxidation state synthons.^35,100,101^ Solutions of 3M remain unchanged over the course of days or weeks by ^1^H NMR spectroscopy, which contrasts the thorium complex [{Th^iv^(Cp′)_3_}_2_(µ-N_2_C_10_H_10_)] (Cp′ = {C_5_H_3_-1,3-(SiMe_3_)_2_}), which slowly reverts to the Th(iii) precursor and unknown byproducts over time.^49^ Similarly, the double-reduction of 4,4′-bipyridine to give bimetallic species bridged by the 4,4′-bipyridine dianion, as with [{M^iii^(NHAr^*i*Pr6^)_2_}_2_(µ-N_2_C_10_H_10_)] (2M, M = Y, La, Tm, Lu), is well-precedented.^49,67,82,101,102^

During the course of isolating 2Y, 2Tm, and 2Lu, it became apparent that while SCXRD and NMR spectroscopic data suggested the bridging 4,4′-bipyridine moiety was doubly reduced due to the short C–C bond length and diamagnetic NMR spectra (except for 2Tm), UV-vis-NIR and EPR spectroscopies of isolated crystalline material suggested the presence of a radical impurity. UV-vis-NIR and NMR titration studies subsequently showed that the 2M complexes (M = Y, Tm, Lu) react with additional equivalents of neutral 4,4′-bipyridine, as evidenced by the isolation of complex 4, which results in trace quantities of radical species in isolated samples of 2M. Crucially, the reaction pathway that gives 4 exists at substoichiometric concentrations of 4,4′-bipyridine. Complex 2La appears unreactive to additional 4,4′-bipyridine. In 2La, as in 1La and 3La, the metal centre retains close contact with two flanking Tripp rings from the ligands, whereas in 2Y, 2Tm, and 2Lu, one of these coordinating Tripp groups moves away to accommodate the additional N donor. Despite the La(iii) ion being the largest of the four,^92^ it is somewhat softer and provides a better size match for the {M^iii^(Tripp)_2_} cavity. This shields the La-centre from the approach of additional 4,4′-bipyridine ligands, and precludes the formation of higher-order aggregates. EPR spectroscopy of isolated 2M samples did not show signals due to the precursor 1M complexes over time, suggesting that 2M and 1M are not in equilibrium with each other.

Taken alongside the titration studies and the unreactivity of 2La towards 4,4′-bipyridine, these data suggest that the formation of 4 proceeds stepwise, requiring the initial formation of 2M, followed by coordination and electron transfer to additional equivalents of 4,4′-bipyridine. Tetrameric radical-bridged lanthanide complexes using the 1,4-pyrazine^103^ (C_4_H_4_N_2_, M = Gd, Dy, Yb) and 1,2,4,5-tetrazene^104^ (C_2_H_2_N_4_, M = Tb) radicals have been reported, which all exhibit a similar square-like arrangement of the four lanthanide metals. Intriguingly, the Gd(iii) and Dy(iii) pyrazine complexes can be isolated in both the dimeric form, like 2M, or as tetrameric species, like 4, either by careful control of the stoichiometry or by dissolution in polar solvents.

Attempts to isolate crystalline samples of complex 4 on a larger scale were unsuccessful. However, this agrees with the solution behaviour described above. The UV-vis-NIR titration experiments show that mixtures of 1M (M = Y, La, Tm, Lu) and 4,4′-bipyridine rapidly form new species even when extremely low equivalents of 4,4′-bipyridine are added – the spectra show broad and strong absorbances across the UV-vis range. As greater equivalents of 4,4′-bipyridine are added to 1Y, 1Tm or 1Lu, new species form, which show somewhat sharper features in the UV-vis range. In the case of 1La, the UV-vis-NIR titration data show that the addition of 4,4′-bipyridine beyond that required to form 2La does not substantially alter the UV-vis spectrum; this data, therefore, provides a spectroscopic handle for the {4,4′-bipyridine}^2−^ dianion in our system (Fig. S150). Revisiting the UV-vis-NIR titration data for 1Y, 1Tm, and 1Lu shows that the broad spectral features we attribute to {4,4′-bipyridine}^2−^ are apparent with low equivalents of 4,4′-bipyridine. However, at 0.23 (1Y), 0.11 (1Tm), or 0.13 (1Lu) equivalents of 4,4′-bipyridine, respectively, the UV-vis-NIR data already show the same sharper spectral features that simply continue to grow in strength with additional 4,4′-bipyridine (Fig. S149, S151, and S152). These sharper features are associated with the {4,4′-bipyridyl}˙^−^ radical anion,^62–66^ which is also present in 4.

In sum, we suggest that when 4,4′-bipyridine is added to solutions of 1M (M = Y, La, Tm, Lu), the components react rapidly to form 2M, even at substoichiometric quantities of 4,4′-bipyridine. In the case of 1La, the product complex (2La) appears almost unreactive to 4,4′-bipyridine except at high ratios (approaching 1 : 1), and so as further 4,4′-bipyridine is added, the formation of additional 2La is all that is observed as the remaining 1La is consumed. With 1Y, 1Tm, and 1Lu, the product complexes (2Y, 2Tm, and 2Lu) appear to react with 4,4′-bipyridine at a similar rate to the 1M complexes, giving rise to a mixture of species, such as 4, and possibly others that are less crystalline and which we have been unable to characterise.

## Conclusions

Cyclic voltammetry of the divalent terphenyl anilide rare earth complexes [M^ii^(NHAr^*i*Pr6^)_2_] (1M, M = Y, La, Sm, Tm, Yb, Lu) using an internal electrochemical standard shows their M(iii/ii) reduction couples span the range −2.32 V to −1.20 V (*vs.* [Fe(Cp)_2_]^+/0^) are more anodic than related divalent rare earth complexes. The Eu(ii) complex showed no electrochemical response, regardless of whether the experiments were performed in the light or dark. Electrochemical measurements do not necessarily correlate with chemical reducing power; therefore, the reactivity of all complexes towards pyridine, 4,4′-bipyridine, naphthalene, and anthracene was explored, given their well-established reduction chemistry.

While the Sm(ii), Eu(ii), and Yb(ii) complexes were unreactive towards 4,4′-bipyridine and pyridine, the Y(ii), La(ii), Tm(ii), and Lu(ii) complexes were found to reduce 4,4′-bipyridine to its dianion in bimetallic [{M^iii^(NHAr^*i*Pr6^)_2_}_2_(µ-N_2_C_10_H_8_)] (1M, M = Y, La, Tm, Lu), and promote the reduction and subsequent C–C coupling of pyridine to give bimetallic [{M^iii^(NHAr^*i*Pr6^)_2_}_2_(µ-N_2_C_10_H_10_)] (3M, M = Y, La, Tm, Lu). The reduction potentials of 4,4′-bipyridine and pyridine are more cathodic than the measured M(iii/ii) couples of the precursor complexes. As none of the divalent precursor complexes showed reactivity towards naphthalene or anthracene, both of which are more easily reduced than pyridine, these serve as examples of coordination-induced reduction.

Single-crystal X-ray diffraction and NMR spectroscopy data indicated full reduction to the 4,4′-bipyridine dianion for all 2M complexes; however, UV-vis-NIR and EPR spectroscopy indicated the trace quantities of the 4,4′-bipyridyl radical anion in isolated samples of 2Y, 2Tm, and 2Lu. UV-vis-NIR and NMR titration studies showed that 2Y, 2Tm, and 2Lu react with additional equivalents of 4,4′-bipyridine, even at substoichiometric ratios, and in one instance, several crystals of the tetrametallic complex [{Y^iii^(NHAr^*i*Pr6^)_2_}_4_(µ-N_2_C_10_H_8_)_4_] (4) were isolated, which contains four bridging 4,4′-bipyridine radical anions. Complex 2La was inert towards additional 4,4′-bipyridine until a large excess was added.

This study highlights the value of assessing rare-earth redox chemistry using both chemical and electrochemical methods and describes a case in which the results are starkly contrasting.

## Author contributions

C. A. P. G. provided the original concept. R. E. M. and H. S. synthesised and characterised the complexes. R. E. M. collected, solved, and refined the single-crystal XRD data, and G. F. S. W. performed final refinement and validation. B. L. L. R performed electrochemical measurements and data analysis. I. J. C. performed EPR spectroscopy measurements, E. J. L. M. supervised the EPR measurements and data interpretation. C. A. P. G. performed DFT calculations and interpretation. R. E. M. wrote the first draft of the manuscript, and C. A. P. G. revised the manuscript with contributions from all other authors.

## Conflicts of interest

There are no conflicts to declare.

## Supplementary Material

SC-017-D6SC03059A-s001

SC-017-D6SC03059A-s002

## Data Availability

CCDC 2478500 (2Y), 2478501 (2La), 2478502 (2Tm), 2478503 (2Lu), 2478504 (3Y), 2478505 (3La), 2478506 (3Tm), 2478507 (3Lu), and 2478508 (4) contain the supplementary crystallographic data for this paper.^106*a*–*i*^ Raw instrument and calculation outputs are deposited at FigShare (DOI: 10.48420/31748533). All other data are available in the supplementary information (SI). A preprint of this article was previously deposited on ChemRxiv.^105^ Supplementary information is available. See DOI: https://doi.org/10.1039/d6sc03059a.
