# Supplementary material for: One- and two-electron coordinatively-induced reduction of N-heterocycles by divalent rare earth terphenyl anilide complexes
Source: Chem Sci. 2026 Jun 9;17(29):14420–31. doi: 10.1039/d6sc03059a (PMC13277779; doi:10.1039/d6sc03059a)
Supplement: SC-017-D6SC03059A-s001 [file SC-017-D6SC03059A-s001.pdf]

Supplementary Information (SI) for Chemical Science.
This journal is © The Royal Society of Chemistry 2026

Supporting Information for:

One- and Two-Electron Coordinatively-Induced Reduction of *N*-Heterocycles by Divalent Rare Earth Terphenyl Anilide Complexes

Ross E. MacKenzie,[1,2] Benjamin L. L. Réant,[1,2] Iain J. Cameron,[1,2] Harry M. Silver,[1,2] George F. S. Whitehead,[2] Eric J. L. McInnes,[2] Conrad A. P. Goodwin*[1,2]

[1] Centre for Radiochemistry Research, The University of Manchester, Oxford Road, Manchester, M13 9PL (UK).

[2] Department of Chemistry, The University of Manchester, Oxford Road, Manchester, M13 9PL (UK).

*Correspondence: conrad.goodwin@manchester.ac.uk

Table of Contents

S1. Experimental Details 3
Equipment, materials, and solvents 3
A note on NMR spectroscopy of paramagnetic samples 4
Synthesis of **2M** (M = Y, La, Tm, Lu) 6
Synthesis of **3M** (M = Y, La, Tm, Lu) 9
Isolation of [{$Y^{III}(NHAr^{iPr6})_2(\mu-N_2C_{10}H_{10})\}_4$] (**4**), and attempted independent synthesis 12
S2. Crystallography 13
General Considerations 13
S3. Molecular structures 18
Complexes **2M** (M = Y, La, Tm, Lu) 18
Complexes **3M** (M = Y, La, Tm, Lu) 22
Bond metrics for **2M** (M = Y, La, Tm, Lu) and **3M** (M = Y, La, Tm, Lu) 26
S4. NMR spectroscopy plots 29
NMR spectra of **2M** (M = Y, La, Tm, Lu) 29
NMR spectra of **3M** (M = Y, La, Tm, Lu) 35
Magnetic moments determined by NMR spectroscopy (Evans method) 39
S5. ATR-IR spectroscopy 40
ATR-IR spectra of **2M** (M = Y, La, Tm, Lu) 40
ATR-IR spectra of **3M** (M = Y, La, Tm, Lu) 42
S6. UV-Vis-NIR spectroscopy 44
UV-Vis-NIR spectra of **2M** (M = Y, La, Tm, Lu) 44
Solid State UV-vis spectra of **2M** (M = Y, La, Tm, Lu) 46
UV-Vis-NIR spectra of **3M** (M = Y, La, Tm, Lu) 49
S8. Reactivity study with 1M (M = Sm, Eu, Yb) 91
NMR spectroscopy 91
UV-Vis-NIR spectroscopy 97
S9. Reactivity study with 1M (M = Y, La, Tm, Lu) 99
NMR spectroscopy 99
Reactions with naphthalene and anthracene 102
UV-Vis-NIR spectroscopy 106
S10. EPR Spectroscopy 111
S11. Computational methodology 117
General Considerations 117
S12. References 118

S1. Experimental Details

Equipment, materials, and solvents

Unless otherwise described, all syntheses and manipulations were conducted under BOC PureShield argon (99.995%) with rigorous exclusion of oxygen and water using Schlenk line and glove box techniques in an MBraun Lab Star™ or a Glovebox Systemtechnik MEGA. 3 Å molecular sieves were activated by heating for 8 hours at 300°C, 10^{-3} mbar. Toluene, mesitylene, Et_2O, and *n*-hexane were degassed by sparging and dried by passage through neutral alumina columns (INERT Corp.), and then degassed under vacuum and stored over a K mirror and used immediately. d_6-benzene (Merck) was dried by refluxing over K metal for 4-5 days followed by vacuum transfer and storage in a J. Youngs valve appended vessel. $[M^{II}(NHAr^{iPr6})_2]$ (**1M**) (M = Y, La, Sm, Eu, Tm, Yb, Lu) were prepared as described previously, or by modification of these routes.[1] Pyridine (Merck) was dried by refluxing over CaH_2 metal for 4-5 days, degassed under vacuum and stored over Å molecular sieves for 7 days before use. 4,4′-bipyridine (Sigma Aldrich) was dried under vacuum (10^{-3} mbar) and stored in the glovebox before use. $[nBu_4N][BPh_4]$ was prepared as described elsewhere and recrystallized three times from DCM layered with hexane then dried under vacuum (10^{-3} mbar) for 24 hours before use. $[Fe^{II}(Cp^*)_2]$ (Fc*; Cp* = $\{C_5Me_5\}$; Merck), and $[Fe^{II}(Cp)_2]$ (Fc; Cp = $\{C_5H_5\}$; Aldrich) were sublimed before to use. All glassware, and glass-fibre filter discs, were stored in an oven (150°C), and glassware was further dried under vacuum (10^{-3} mbar) with heating from a butane flame. Solution phase UV-Vis-NIR spectra were collected at ambient temperature using a PerkinElmer Lambda 1050 UV-Vis-NIR spectrometer. The solution was contained in a low-volume (1 mL) screw-capped quartz cuvette (10 × 4 mm path length fluorescence cell) or a Hellma 1 mm path length absorbance cell. Solids were contained in the Praying Mantis attachment, and Spectralon® was used as the reference. ATR FT-IR spectra of microcrystalline samples were collected using a Bruker ALPHA II FT-IR spectrometer equipped with a Platinum ATR module with a diamond window. NMR spectroscopic data collection was performed on either a Bruker Avance III (400 MHz), Bruker Avance III HD (400 MHz), between 295 K and 299 K. Elemental microanalyses (C/H/N) were carried out by Martin Jennings and Anne Davies at the University of Manchester.

A note on NMR spectroscopy of paramagnetic samples

All spectra were referenced to internal solvent residuals (^{1}H and ^{13}C) or externally to 10% TMS in *d*-chloroform *via* **Eq. S1**, which is the IUPAC recommended convention.

$$\Delta\ (Hz) = \frac{SR^{1H}}{SF^{1H}} \times\ SF^{NUC}$$

Eq. S1.

Where SR^{1H} is the spectrum reference frequency (in Hz) of a reference ^{1}H NMR spectrum collected with TMS set to 0 ppm collected under the same experimental conditions; SF^{1H} is the spectrometer frequency (in MHz) for the ^{1}H nucleus; SF^{NUC} is the spectrometer frequency (in MHz) of the nucleus in question. The answer is given in Hz.

Paramagnetic samples become magnetised in the presence of an external magnetic field, such as that of an NMR spectrometer. The level of magnetisation will approximately follow Curie's law when saturation of magnetisation is not reached ($\mu_B\ \leq\ k_B T$). The magnetic response of a sample is proportional to: (i) sample temperature; (ii) external field strength; and (iii) sample concentration. This necessarily affects the reproducibility of the chemical shifts reported for paramagnetic samples – a sample run at a different concentration, field strength, or temperature will produce a different paramagnetic contribution to the observed chemical shift. Moreover, the direction that an individual chemical shift will change (upfield or downfield) cannot easily be predicted. Finally, modern convention to reference chemical shift relative to solvent residual peaks further complicates the comparison of multiple samples, as the factors listed above will also change the absolute shift of the solvent peak (relative to the spectrometer proton frequency), as the susceptibility of solvent molecules may differ from the ligand atoms surrounding a paramagnetic ion. Though solvent effects even in diamagnetic NMR samples can vary the chemical shift by several ppm for some nuclei.[2]

Considering these caveats, we report our data as it is output from the experiment with rounding to two decimal places, as this is the convention. We defer to the expertise of the reader to interpret the data reported here in a way that is appropriate for their needs.

Table S1. Compound numbering, reaction scale (by metal quantity used), formula, yield.

	Scale (mmol metal)	Formula	Yield (g)	% Yield
**2Y**	0.5	$[\{Y^{III}(NHAr^{iPr6})_2\}_2(\mu\text{-}N_2C_{10}H_8)]$	0.1856	32
**2La**	0.5	$[\{La^{III}(NHAr^{iPr6})_2\}_2(\mu\text{-}N_2C_{10}H_8)]$	0.1469	25
**2Tm**	0.5	$[\{Tm^{III}(NHAr^{iPr6})_2\}_2(\mu\text{-}N_2C_{10}H_8)]$	0.2044	32
**2Lu**	0.5	$[\{Lu^{III}(NHAr^{iPr6})_2\}_2(\mu\text{-}N_2C_{10}H_8)]$	0.2998	48
**3Y**	0.5	$[\{Y^{III}(NHAr^{iPr6})_2\}_2(\mu\text{-}N_2C_{10}H_{10})]$	0.3000	51
**3La**	0.5	$[\{La^{III}(NHAr^{iPr6})_2\}_2(\mu\text{-}N_2C_{10}H_{10})]$	0.3150	52
**3Tm**	0.5	$[\{Tm^{III}(NHAr^{iPr6})_2\}_2(\mu\text{-}N_2C_{10}H_{10})]$	0.2141	34
**3Lu**	0.5	$[\{Lu^{III}(NHAr^{iPr6})_2\}_2(\mu\text{-}N_2C_{10}H_{10})]$	0.1381	22

Note: Yields are representative of a single iteration only and are intended only to indicate what might be expected from these unoptimised reactions.

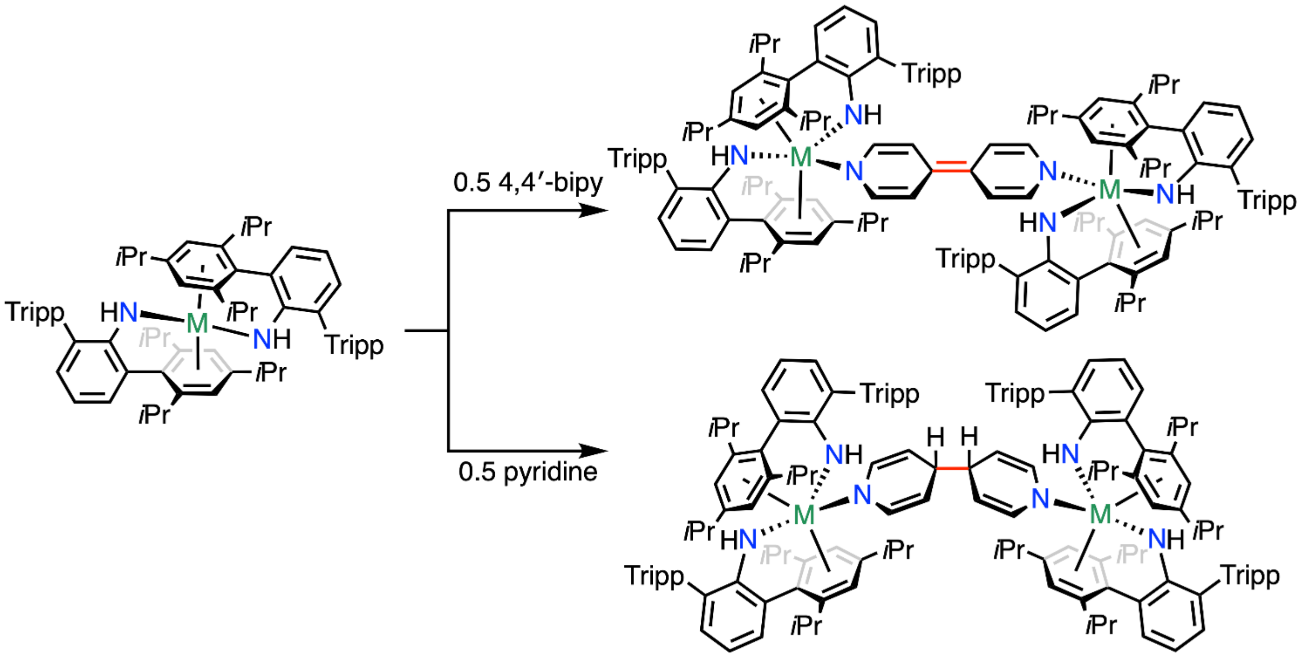

Scheme S1. Synthesis of $[\{M^{III}(NHAr^{iPr6})_2\}_2(\mu\text{-}N_2C_{10}H_8)]$ (**2M**, M = Y, La, Tm, Lu), and $[\{M^{III}(NHAr^{iPr6})_2\}_2(\mu\text{-}N_2C_{10}H_{10})]$ (**3M**, M = Y, La, Tm, Lu).

*Synthesis of **2M** (M = Y, La, Tm, Lu)*

*Synthesis of [{Y^{III}($NHAr^{iPr6}$)$_2$}$_2$(μ-$N_2C_{10}H_8$)]·(C_6H_{14}) (**2Y**)*

Toluene (50 mL) was added to a pre-cooled (–98°C) stirring mixture of solid [Y^{II}($NHAr^{iPr6}$)$_2$] (0.541 g, 0.5 mmol) and 4,4′-bipyridine (0.039 g, 0.25 mmol) in a glass Schlenk vessel equipped with a PTFE-coated stirrer bar. The mixture was stirred for 5 minutes at –98°C and allowed to warm up to room temperature, during which the solution quickly lightened from dark red to deep blue, and the formation of deep blue solids. After stirring for 1 hour, the volatiles were removed under vacuum (10^{-3} mbar), which left a deep blue powder. Hexane (20 mL) was added, and the mixture was vigorously refluxed with manual agitation to loosen the solids from the vessel walls. The deep blue solution was filtered through a glass microfibre filter disc. This mixture was stored at room temperature for 16 hours, followed by –5°C for 24 hours to afford blue plates of **2Y**. Crystals were isolated by decanting the supernatant, then dried under vacuum (10^{-3} mbar, 2 hours). Yield = 0.1856 g (32%).

Elemental analysis on $C_{154}H_{208}N_6Y_2$ calc. (%): C = 79.69, H = 9.03, N = 3.62; found (%): C = 78.19, H = 8.94, N = 3.86.

^{1}H NMR (d_6-benzene, 400.13 MHz, 298 K): δ = 7.35 (m, 16H, Tripp-3,5-*H*), 6.93 (d, $^3J_{HH}$ = 7.5 Hz, 4H, Ar-3,5-*H*), 6.61 (t, $^3J_{HH}$ = 7.4 Hz, 4H, Ar-4-*H*), 5.97 (d, $^3J_{HH}$ = 7.3 Hz, 4H, N-2,6-*H*), 5.63 (d, $^3J_{HH}$ = 7.5 Hz, 4H, N-3,5-*H*), 4.17 (s, 4H, YN*H*), 3.13 – 2.95 (m, 16H, 2,6-C*H*(CH_3)$_2$), 1.63 (m, 48H, 4-CH(C*H*$_3$)$_2$), 1.07 (m, 96H, 2,6-CH(C*H*$_3$)$_2$).

^{13}C{^{1}H} NMR (d_6-benzene, 100.62 MHz, 298 K): δ = 148.07 (Tripp-2,6-*C* and Tripp-*ipso*-*C*), 133.93 (N-2,6-*C*H), 121.15 (Tripp-3,5-*C*H), 113.02 (Ar-4-*C*H), 106.03 (N-3,5-*C*H), 34.49 (4-*C*H(CH_3)$_2$), 30.61 (2,6-*C*H(CH_3)$_2$), 24.45 (4-CH(*C*H$_3$)$_2$), 24.05 (2,6-CH(*C*H$_3$)$_2$), 24.03 (2,6-CH(*C*H$_3$)$_2$).

UV-Vis-NIR (toluene): λ_{max} (cm^{-1}; ε) = 797 (12,543, 4,857), 719 (13,915, 9,509), 634 (15,782, 21,519), 584 (17,130, 21843), 534 (18,732, 15,850).

FT-IR (ATR, microcrystalline): cm^{-1} = 3,043 (vw), 2,957 (s), 2,927 (m), 2,867 (w), 1,966 (vw), 1,630 (vw), 1,599 (vs), 1,461 (m), 1,410 (vs), 1,381 (m), 1,361 (m), 1,317 (w), 1,280 (vw), 1,254 (s), 1,202 (s), 1,102 (w), 1,075 (m), 1,038 (w), 1,015 (s), 962 (vs), 939 (vs), 892 (w), 876 (s), 849 (vs), 830 (s), 797 (m), 775 (s), 750 (vs), 686 (vw), 660 (m), 615 (s), 584 (s), 547 (m), 504 (w), 493 (w), 473 (vw), 460 (vw), 444 (vw), 434 (w), 425 (w), 415 (vw), 407 (vw).

*Synthesis of [{La^{III}($NHAr^{iPr6}$)$_2$}$_2$(μ-$N_2C_{10}H_8$)]·(C_6H_{14}) (**2La**)*

[La^{II}($NHAr^{iPr6}$)$_2$] (0.566 g, 0.5 mmol), 4,4′-bipyridine (0.039 g, 0.25 mmol). Dark green solution and two crops of deep green crystals (yield: 0.1469 g, 25%). Crystals suitable for

SC-XRD were obtained from a solution of **1La** and 0.5 equivalents of 4,4′-bipyridine in mesitylene.

Elemental analysis on $C_{154}H_{208}N_6La_2$ calc. (%): C = 76.40, H = 8.66, N = 3.47; found (%): C = 70.81, H = 8.44, N = 3.55. Repeated measurements from independent batches of material reproducibly gave low values for carbon, but satisfactory values for nitrogen and hydrogen. Therefore, we attribute the deviation in %C to carbide formation.[3]

^{1}H NMR (d_6-benzene, 400.13 MHz, 298 K): δ = 7.55 (m, 4H, Tripp-3,5-*H*), 7.31 (m, 12H, Tripp-3,5-*H*), 6.96 (m, 8H, Ar-3,5-*H*), 6.62 (t, $^3J_{HH}$ = 7.3 Hz, 4H, Ar-4-*H*), 5.96 (d, $^3J_{HH}$ = 7.2 Hz, 4H, N-2,6-*H*), 5.60 (d, $^3J_{HH}$ = 7.5 Hz, 4H, N-3,5-*H*), 4.59 (s, 4H, LaN*H*), 3.25 (m, 4H, 4-C*H*(CH_3)$_2$), 2.95 (m, 16H, 2,6-C*H*(CH_3)$_2$), 2.15 (m, 4H, 4-C*H*(CH_3)$_2$), 1.54 (m, 48H, CH(C*H*$_3$)$_2$), 1.36 (m, 48H, CH(C*H*$_3$)$_2$), 1.09 (m, 36H, CH(C*H*$_3$)$_2$), 0.92 (m, 12H, CH(C*H*$_3$)$_2$).

^{13}C{^{1}H} NMR (d_6-benzene, 100.62 MHz, 298 K): δ = 158.53 (Ar-*C*-N(H)), 148.87 (Tripp-*ipso*-*C*), 148.06 (Tripp-2,6-*C*), 142.95 (Ar-2,6-*C*), 133.85 (N-2,6-*C*H), 132.48 (N-4-*C*), 129.69 (Tripp-4-*C*), 125.33 (Ar-3,5-*C*H), 121.51 (Tripp-3,5-*C*H), 112.62 (N-3,5-*C*H), 106.10 (Ar-4-*C*H), 35.70 (4-*C*H(CH_3)$_2$), 33.02 (2,6-*C*H(CH_3)$_2$), 26.81 (4-CH(*C*H$_3$)$_2$), 26.65 (2,6-CH(*C*H$_3$)$_2$).

UV-Vis-NIR (toluene): λ_{max} (cm^{-1}; ε) = 610 (16,388, 11,435).

FT-IR (ATR, microcrystalline): cm^{-1} = 2,035 (vw), 1,757 (vw), 1,624 (vw), 1,603 (w), 1,583 (vw), 1,566 (vw), 1,459 (s), 1,441 (s), 1,412 (vs), 1,381 (m), 1,361 (s), 1,317 (w), 1,280 (vw), 1,256 (vs), 1,198 (w), 1,167 (vw), 1,102 (vw), 1,071 (s), 1,056 (w), 1,034 (vw), 1,005 (vw), 958 (vs), 933 (vs), 908 (m), 890 (w), 876 (vs), 847 (vs), 830 (s), 795 (w), 777 (w), 746 (vs), 682 (vw), 654 (w), 602 (s), 584 (s), 559 (m), 545 (s), 526 (w), 506 (w), 483 (m), 475 (m), 469 (m), 458 (m), 452 (m), 444 (m), 436 (m), 421 (s).

Synthesis of $[\{Tm^{III}(NHAr^{iPr6})_2\}_2(\mu\text{-}N_2C_{10}H_8)]\cdot(C_6H_{14})$ ***(2Tm)***

$[Tm^{II}(NHAr^{iPr6})_2]$ (0.5808 g, 0.5 mmol), 4,4′-bipyridine (0.039 g, 0.25 mmol). Deep blue solution and two crops of deep blue crystals (yield: 0.2044 g, 32%). Better crystals suitable for SC-XRD were obtained from a solution of **1Tm** and 0.5 equivalents of 4,4′-bipyridine in mesitylene.

Elemental analysis on $C_{154}H_{208}N_6Tm_2$ calc. (%): C = 74.55, H = 8.45, N = 3.39; found (%): C = 72.95, H = 8.24, N = 3.27

^{1}H NMR (d_6-benzene, 400.13 MHz, 298 K): δ = 151.84 (br. s, FWHM = 628 Hz), 143.94 (br. s, FWHM = 260 Hz), 131.19 (br. s, FWHM = 443 Hz), 96.90 (br. s, FWHM = 244 Hz), 83.20 (br. s, FWHM = 240 Hz), 78.04 (br. s, FWHM = 214 Hz), 29.37 (br. s, FWHM = 952 Hz), 20.53 (br. s, FWHM = 194 Hz), 11.75 (br. s, FWHM = 1128 Hz), 9.72 (br. s, FWHM = 177 Hz), 1.29 (br. s, FWHM = 46 Hz), 0.92 (br. s, FWHM = 31 Hz), −12.83 (br. s, FWHM = 108

Hz), −14.13 (br. s, FWHM = 110 Hz), −17.66 (br. s, FWHM = 138 Hz), −19.88 (br. s, FWHM = 192 Hz), −30.07 (br. s, FWHM = 192 Hz), −39.51 (br. s, FWHM = 203 Hz), −47.38 (br. s, FWHM = 245 Hz), −50.57 (br. s, FWHM = 132 Hz), −62.32 (br. s, FWHM = 95 Hz), −72.32 (br. s, FWHM = 157 Hz), −89.67 (br. s, FWHM = 541 Hz), −102.82 (br. s, FWHM = 213 Hz), −108.20 (br. s, FWHM = 494 Hz), −122.32 (br. s, FWHM = 986 Hz), −166.11 (br. s, FWHM = 385 Hz), −173.39 (br. s, FWHM = 290 Hz).

UV-Vis-NIR (toluene): λ_{max} (cm^{-1}; ε) = 1,218 (8,212, 43), 1,130 (8,851, 68), 786 (12,727, 2,015), 708 (14,134, 2,852), 624 (16,034, 3,889), 568 (17,595, 3,982).

FT-IR (ATR, microcrystalline): cm^{-1} = 3,484 (vw), 3,384 (vw), 3,297 (vw), 3,046 (vw), 2,957 (vs), 2,927 (s), 2,867 (m), 1,994 (vw), 1,676 (vw), 1,628 (w), 1,601 (s), 1,583 (w), 1,533 (vw), 1,459 (m), 1,441 (w), 1,410 (vs), 1,381 (s), 1,361 (s), 1,319 (m), 1,254 (vs), 1,200 (s), 1,165 (vw), 1,102 (w), 1,075 (m), 1,036 (w), 1,015 (m), 1,005 (w), 964 (vs), 939 (vs), 896 (m), 876 (s), 847 (vs), 830 (s), 795 (m), 777 (m), 750 (vs), 711 (vw), 686 (vw), 660 (s), 610 (s), 586 (s), 575 (m), 545 (m), 497 (w), 487 (w), 471 (w), 450 (w), 440 (vw), 432 (vw), 419 (m).

Synthesis of $[\{Lu^{III}(NHAr^{iPr6})_2\}_2(\mu\text{-}N_2C_{10}H_8)]\cdot(C_6H_{14})$ ***(2Lu)***

$[Lu^{II}(NHAr^{iPr6})_2]$ (0.584 g, 0.5 mmol), 4,4′-bipyridine (0.039 g, 0.25 mmol). Royal blue solution and two crops of dark blue crystals. (yield: 0.2998 g, 48%). Crystals suitable for SC-XRD were obtained from a concentrated solution in *d*6-benzene.

Elemental analysis on $C_{154}H_{208}N_6Lu_2$ calc. (%): C = 74.19, H = 8.41, N = 3.37; found (%): C = 70.48, H = 8.21, N = 3.24

^{1}H NMR (*d*6-benzene, 400.13 MHz, 298 K): δ = 7.50 – 7.26 (m, 16H, Tripp-3,5-*H*), 6.92 (d, $^3J_{HH}$ = 7.5 Hz, 8H, Ar-3,5-*H*), 6.60 (t, $^3J_{HH}$ = 7.4 Hz, 4H, Ar-4-*H*), 5.83 (d, $^3J_{HH}$ = 7.3 Hz, 4H, N-2,6-*H*), 5.65 (d, $^3J_{HH}$ = 7.5 Hz, 4H, N-3,5-*H*), 4.00 (s, 4H, LuN(*H*)), 3.14 – 2.97 (m, 16H, Tripp-2,6-C*H*(CH_3)$_2$), 2.55 – 2.23 (m, 8H, Tripp-4-C*H*(CH_3)$_2$), 1.73 – 1.37 (m, 48H, Tripp-4-CH(C*H*$_3$)$_2$), 1.33 – 0.96 (m, 96H, Tripp-2,6-CH(C*H*$_3$)$_2$).

^{13}C{^{1}H} NMR (*d*6-benzene, 100.62 MHz, 298 K): δ = 158.06 (Ar-*C*N(H)), 148.84 (Tripp-2,6-*C* and Tripp-*ipso*-*C*), 134.00 (N-2,6-*C*H), 131.95 (Ar-3,5-*C*H), 125.22 (Ar-2,6-*C*), 121.37 (Tripp-3,5-*C*H), 113.65 (Ar-4-*C*H), 107.19 (N-3,5-*C*H), 33.46 (4-*C*H(CH_3)$_2$), 30.97 (4-H(*C*H$_3$)$_2$), 26.64 (2,6-*C*H(CH_3)$_2$), 23.36 (2,6-CH(*C*H$_3$)$_2$), 23.06 (2,6-CH(*C*H$_3$)$_2$).

UV-Vis-NIR (toluene): λ_{max} (cm^{-1}; ε) = 797 (12,543, 4,857), 719 (13,915, 9,509), 634 (15,782, 21,519), 584 (17,130, 21843), 534 (18,732, 15,850).

FT-IR (ATR, microcrystalline): cm^{-1} = 3,037 (vw), 2,955 (vs), 2,927 (s), 2,865 (m), 2,097 (vw), 2,040 (vw), 2,009 (vw), 1,975 (vw), 1,669 (vw), 1,630 (m), 1,603 (w), 1,580 (w), 1,540 (vw), 1,461 (s), 1,414 (vs), 1,381 (s), 1,361 (s), 1,321 (m), 1,278 (m), 1,256 (vs), 1,200 (s),

1,163 (w), 1,100 (m), 1,075 (s), 1,038 (m), 1,023 (vw), 1,017 (vw), 1,005 (w), 964 (vs), 939 (vs), 896 (m), 873 (s), 847 (vs), 830 (s), 795 (w), 777 (w), 750 (vs), 662 (m), 652 (w), 612 (s), 575 (m), 547 (m), 501 (w), 489 (w), 475 (vw), 442 (vw), 415 (w).

*Synthesis of **3M** (M = Y, La, Tm, Lu)*

*Synthesis of $[\{Y^{III}(NHAr^{iPr6})_2\}_2(\mu\text{-}N_2C_{10}H_{10})]\cdot(C_6H_{14})$ (**3Y**)*

Toluene (50 mL) was added to a pre-cooled (–98°C) stirring mixture of solid $[Y^{II}(NHAr^{iPr6})_2]$ (0.5412 g, 0.5 mmol) in a glass Schlenk vessel equipped with a PTFE-coated stirrer bar. Pyridine (0.004 mL, 0.5 mmol) was added via micro syringe. The mixture was stirred for 5 minutes at –98°C and allowed to warm up to room temperature, during which the solution quickly lightened from dark red to bright orange. After stirring for 1 hour, the volatiles were removed under vacuum (10^{-3} mbar), resulting in a bright orange powder. Hexane (10 mL) was added, and the mixture was briefly (<1 min) refluxed with manual agitation to loosen solids from the vessel walls. The bright orange solution was filtered through a glass microfibre filter disc. Concentration of the bright orange supernatant to *ca*. 1 mL yielded a moderate amount of orange solids, which were heated to solution and allowed to cool to room temperature. This mixture was stored at 5°C for 16 hours, followed by –30°C for 24 hours to afford orange blocks of **3Y**. Crystals were isolated by decanting the supernatant and drying under vacuum (10^{-3} mbar, 2 hours). A second crop was obtained in a similar fashion (combined yield = 0.300 g, 51%).

Elemental analysis on $C_{154}H_{210}N_6Y_2$ calc. (%): C = 79.62, H = 9.11, N = 3.62; found (%): C = 78.81, H = 9.08, N = 3.57.

^{1}H NMR (d_6-benzene, 400.13 MHz, 298 K): δ = 7.38 (m, 16H, Tripp-3,5-*H*), 6.96 (m, 8H, Ar-3,5-*H*), 6.64 (t, $^3J_{HH}$ = 7.4 Hz, 4H, Ar-4-*H*), 6.04 (s, 4H, N-2,6-*H*), 4.59 (m, 4H, N-3,5-*H*), 4.16 (s, 4H, YN*H*), 3.52 (m, 2H, N-4-*H*), 3.36–2.81 (m, 24H, C*H*(CH$_3$)$_2$), 1.66 (s, 24H, 4-CH(C*H*$_3$)$_2$), 1.48 (s, 24H, 4-CH(C*H*$_3$)$_2$), 1.26 (m, 60H, 2,6-CH(C*H*$_3$)$_2$), 1.09 (s, 36H, 2,6-CH(C*H*$_3$)$_2$).

UV-Vis-NIR (toluene): λ_{max} (cm^{-1}; ε) = A broad feature extends from ~500 nm (20,000 cm^{-1}) into the UV region, and beyond our spectral range.

FT-IR (ATR, microcrystalline): cm^{-1} = 3,482 (vw), 3,378 (vw), 2,957 (vs), 2,927 (s), 2,867 (s), 2,162 (vw), 2,040 (vw), 1,975 (vw), 1,646 (vw), 1,603 (vw), 1,578 (m), 1,461 (s), 1,441 (m), 1,412 (vs), 1,381 (s), 1,361 (s), 1,321 (s), 1,256 (vs), 1,211 (w), 1,188 (vw), 1,161 (w), 1,106 (m), 1,075 (s), 1,036 (vw), 1,019 (w), 1,005 (w), 972 (vs), 937 (m), 921 (w), 894 (vw),

876 (s), 847 (vs), 830 (s), 795 (w), 775 (m), 750 (vs), 725 (w), 660 (m), 619 (m), 586 (s), 547 (m), 524 (vw), 493 (w), 460 (m), 427 (w).

Synthesis of [{La^{III}(NHAr^{iPr6})$_2$}$_2$(μ-$N_2C_{10}H_{10}$)]·(C_6H_{14}) ***(3La)***

[La^{II}(NHAr^{iPr6})$_2$] (0.566 g, 0.5 mmol), pyridine (0.5 mmol). Yellow solution and two crops of yellow crystals (yield: 0.315 g, 52%).

Elemental analysis on $C_{154}H_{210}N_6La_2$ calc. (%): C = 76.33, H = 8.73, N = 3.47; found (%): C = 69.84, H = 8.53, N = 3.60. Repeated measurements from independent batches of material reproducibly gave low values for carbon, but satisfactory values for nitrogen and hydrogen. Therefore, we attribute the deviation in %C to carbide formation.[3]

^{1}H NMR (d_6-benzene, 400.13 MHz, 298 K): δ = 7.61 (m, 4H, Tripp-3,5-*H*), 7.31 (m, 12H, Tripp-3,5-*H*), 7.00 (m, 8H, Ar-3,5-*H*), 6.83 (t, $^3J_{HH}$ = 7.3 Hz, 4H, Ar-4-*H*), 6.08 (d, $^3J_{HH}$ = 7.5 Hz, 4H, N-2,6-*H*), 4.59 (m, 8H, N-3,5-*H* and LaN*H*), 3.57 (s, 2H, N-4-*H*), 3.27 (m, 4H, 4-C*H*(CH_3)$_2$), 3.27 (m, 16H, 2,6-C*H*(CH_3)$_2$), 2.18 (m, 4H, 4-C*H*(CH_3)$_2$), 1.54 (m, 48H, CH(C*H*$_3$)$_2$), 1.36 (m, 48H, CH(C*H*$_3$)$_2$), 1.09 (m, 36H, CH(C*H*$_3$)$_2$), 0.92 (m, 12H, CH(C*H*$_3$)$_2$).

^{13}C{^{1}H} NMR (d_6-benzene, 100.62 MHz, 298 K): δ = 158.64 (Ar-*C*-N(H)), 148.88 (Tripp-*ipso*-*C*), 148.08 (Tripp-2,6-*C*), 142.96 (Ar-2,6-*C*), 132.66 (N-2,6-*C*H), 128.50 (Tripp-4-*C*), 124.15 (Ar-3,5-*C*H), 120.31 (Tripp-3,5-*C*H), 111.34 (N-3,5-*C*H), 33.64 (4-*C*H(CH_3)$_2$), 29.77 (2,6-*C*H(CH_3)$_2$), 23.61 (4-CH(*C*H$_3$)$_2$), 23.21 (2,6-CH(*C*H$_3$)$_2$), 23.19 (2,6-CH(*C*H$_3$)$_2$).

UV-Vis-NIR (toluene): λ_{max} (cm^{-1}; ε) = A broad feature extends from ~500 nm (20,000 cm^{-1}) into the UV region, and beyond our spectral range.

FT-IR (ATR, microcrystalline): cm^{-1} = 3,482 (vw), 3,383 (vw), 3,052 (vw), 3,017 (vw), 2,957 (s), 2,927 (m), 2,906 (w), 2,867 (m), 2,803 (vw), 1,648 (vw), 1,628 (vw), 1,603 (vw), 1,580 (w), 1,568 (w), 1,543 (vw), 1,459 (m), 1,441 (m), 1,412 (s), 1,381 (m), 1,361 (m), 1,317 (w), 1,258 (vs), 1,215 (vw), 1,186 (vw), 1,167 (vw), 1,151 (vw), 1,095 (vs), 1,073 (vs), 1,056 (s), 1,017 (vs), 986 (s), 960 (vs), 939 (s), 890 (m), 876 (s), 847 (s), 795 (vs), 748 (vs), 734 (s), 723 (m), 703 (w), 660 (s), 623 (s), 604 (m), 578 (s), 557 (m), 547 (m), 524 (w), 514 (w), 481 (w), 473 (w), 460 (w).

Synthesis of [{Tm^{III}(NHAr^{iPr6})$_2$}$_2$(μ-$N_2C_{10}H_{10}$)]·(C_6H_{14}) ***(3Tm)***

[Tm^{II}(NHAr^{iPr6})$_2$] (0.582 g, 0.5 mmol), pyridine (0.5 mmol). Red/orange solution and two crops of red/orange crystals (yield: 0.214 g, 34%).

Elemental analysis on $C_{154}H_{210}N_6Tm_2$ calc. (%): C = 74.49, H = 8.52, N = 3.38; found (%): C = 73.69, H = 8.55, N = 3.51.

^{1}H NMR (d_6-benzene, 400.13 MHz, 298 K): δ = 166.36 (br. s, FWHM = 411 Hz), 152.19 (br. s, FWHM = 127 Hz), 144.31 (br. s, FWHM = 101 Hz), 33.65 (br. s, FWHM = 889 Hz), 13.13 (br. s, FWHM = 614 Hz), 8.55 (br. s, FWHM = 80 Hz), 6.73 (br. s, FWHM = 320 Hz), 3.62 (br. s, FWHM = 55 Hz), 3.04 (br. s, FWHM = 106 Hz), 1.29 (br. s, FWHM = 55 Hz), −36.61 (br. s, FWHM = 249 Hz), −48.38 (br. s, FWHM = 267 Hz), −77.05 (br. s, FWHM = 105 Hz), −83.99 (br. s, FWHM = 1204 Hz), −119.26 (br. s, FWHM = 602 Hz), −177.85 (br. s, FWHM = 214 Hz), −290.20 (br. s, FWHM = 297 Hz).

UV-Vis-NIR (toluene): λ_{max} (cm^{-1}; ε) = 781 (12,797, 15), 680 (14,701, 7).

FT-IR (ATR, microcrystalline): cm^{-1} = 2,957 (vs), 2,927 (s), 2,865 (m), 1,648 (vw), 1,603 (vw), 1,580 (m), 1,459 (s), 1,443 (w), 1,412 (vs), 1,381 (s), 1,361 (s), 1,319 (s), 1,256 (vs), 1,211 (vw), 1,188 (vw), 1,163 (w), 1,102 (m), 1,075 (s), 1,021 (w), 1,005 (m), 989 (w), 974 (vs), 937 (m), 921 (w), 896 (vw), 876 (s), 847 (vs), 830 (s), 795 (w), 777 (s), 750 (vs), 723 (m), 699 (vw), 662 (m), 637 (w), 621 (m), 586 (s), 547 (m), 520 (vw), 495 (w), 483 (vw), 460 (w), 444 (vw), 421 (vw), 405 (vw).

Synthesis of $[\{Lu^{III}(NHAr^{iPr6})_2\}_2(\mu\text{-}N_2C_{10}H_{10})]\cdot(C_6H_{14})$ ***(3Lu)***

$[Lu^{II}(NHAr^{iPr6})_2]$ (0.584 g, 0.5 mmol), pyridine (0.5 mmol). Orange solution and two crops of orange crystals (yield: 0.1381 g, 22%).

Elemental analysis on $C_{154}H_{210}N_6Tm_2$ calc. (%): C = 74.13, H = 8.48, N = 3.37; found (%): C = 73.37, H = 8.64, N = 3.51.

^{1}H NMR (d_6-benzene, 400.13 MHz, 298 K): δ = 7.38 (m, 16H, Tripp-3,5-*H*), 6.95 (d, $^3J_{HH}$ = 7.2 Hz, 8H, Ar-3,5-*H*), 6.64 (t, $^3J_{HH}$ = 7.4 Hz, 4H, Ar-4-*H*), 5.90 (s, 4H, N-2,6-*H*), 4.64 (m, 4H, N-3,5-*H*), 3.99 (s, 4H, LuN*H*), 3.57 (m, 2H, N-4-*H*), 3.17–2.98 (m, 16H, C*H*(CH$_3$)$_2$), 2.36 (m, 8H, C*H*(CH$_3$)$_2$), 1.67 (s, 24H, 4-CH(C*H*$_3$)$_2$), 1.48 (s, 24H, 4-CH(C*H*$_3$)$_2$), 1.29–1.02 (m, 96H, 2,6-CH(C*H*$_3$)$_2$).

^{13}C{^{1}H} NMR (d_6-benzene, 100.62 MHz, 298 K): δ = 156.17 (Ar-*C*-N(H)), 148.08 (Tripp-*ipso*-*C* and Tripp-2,6-*C*), 129.70 (Ar-3,5-*C*H), 125.25 (N-2,6-*C*H), 121.51 (Tripp-3,5-*C*H), 117.40 (N-3,5-*C*H), 113.65 (Ar-4-*C*H), 34.85 (N-4-*C*), 31.97 (4-*C*H(CH$_3$)$_2$), 30.97 (2,6-*C*H(CH$_3$)$_2$), 24.81 (4-CH(*C*H$_3$)$_2$), 24.42 (2,6-CH(*C*H$_3$)$_2$), 24.39 (2,6-CH(*C*H$_3$)$_2$).

UV-Vis-NIR (toluene): λ_{max} (cm^{-1}; ε) = A broad feature extends from ~500 nm (20,000 cm^{-1}) into the UV region, and beyond our spectral range.

FT-IR (ATR, microcrystalline): cm^{-1} = 3,486 (vw), 3,386 (vw), 3,300 (vw), 3,027 (vw), 2,957 (vs), 2,927 (s), 2,867 (s), 2,113 (vw), 2,026 (vw), 1,974 (vw), 1,760 (vw), 1,648 (vw), 1,603 (vw), 1,580 (w), 1,459 (s), 1,441 (m), 1,414 (vs), 1,381 (s), 1,361 (vs), 1,321 (s), 1,256 (vs), 1,245 (vs), 1,211 (vw), 1,163 (w), 1,104 (m), 1,075 (s), 1,056 (w), 1,023 (w), 1,005 (m), 976

(vs), 939 (m), 921 (w), 898 (vw), 876 (vs), 857 (s), 847 (s), 830 (s), 812 (vw), 795 (w), 775 (m), 750 (vs), 723 (m), 662 (s), 654 (m), 637 (w), 621 (m), 586 (s), 547 (m), 524 (w), 495 (w), 473 (w), 458 (m), 444 (w), 415 (m).

*Isolation of $[\{Y^{III}(NHAr^{iPr6})_2(\mu\text{-}N_2C_{10}H_{10})\}_4]$ (**4**), and attempted independent synthesis*

A solution of **1Y** (10.2 mg) and 4,4′-bipyridine (2.1 mg) gave several deep blue/purple crystals from *ca*. 5 ml Et_2O when stored at –30°C for several days. Further attempts to isolate **4** on larger scales proved unsuccessful (see below).

Attempted independent syntheses

Method A: Toluene (5 mL) was added to a stirring mixture of solid **1Y** (0.048 g, 0.04 mmol) and 4,4′-bipyridine (0.007 g, 0.04 mmol). The mixture was stirred for 1 hour at room temperature, during which the solution quickly darkened from dark red to deep blue. After 1 hour, the volatiles were removed under vacuum (10^{-3} mbar), which gave a deep blue powder. Despite numerous attempts to recrystallise the crude material from several solvents and a range of conditions (toluene, mesitylene, Et_2O; room temperature to –30°C), only polycrystalline material could be obtained.

Method B: Addition of 4,4′-bipyridine (0.005 g, 0.03 mmol) to a solution of **2Y** (0.074 g, 0.03 mmol) in toluene (10 mL) gave a highly insoluble, intractable product mixture. Attempts to recrystallise the crude material from several solvents and a range of conditions (toluene, mesitylene, Et_2O; room temperature to –30°C) yielded only polycrystalline material.

S2. Crystallography

General Considerations

Data for **2Y**, **3Y**, and **3Lu** were collected using a Rigaku XtaLAB Synergy DW diffractometer, equipped with a PhotonJet Cu Kα radiation source (λ = 1.54184 Å), using a 4-circle κ goniometer, a HyPix-6000HE hybrid pixel array detector operating in shutterless mode and an Oxford Cryosystems Cryostream 800 nitrogen flow gas system at a temperature of 100K. Data for **2La**, **2Tm**, **2Lu**, **3La, 3Tm** and **4** were collected using a Rigaku FR-X DW diffractometer, equipped with an FR-X high-intensity rotating anode Cu Kα radiation source (λ = 1.54184 Å) and VariMAXTM microfocus optics, using an AFC-11 4-circle κ goniometer, a HyPix-6000HE hybrid pixel array detector operating in shutterless mode and an Oxford Cryosystems Cryostream 800 plus nitrogen flow gas system at a temperature of 100K. All data were integrated and reduced using Rigaku Oxford Diffraction CryAlisPro v1.171.42. Intensities were integrated from data recorded from ω, or ω and φ rotation, at the frame width and exposure times outlined in **Table S2**. The crystal data for all complexes is outlined in **Table S3** to **Table S5**.

Table S2. Data collection parameters and CCDC reference codes for all structures herein.

	Formula	Frame width (°)	Exposure time (s)	CCDC ref.
**2Y**	$[\{Y^{III}(NHAr^{iPr6})_2\}_2(\mu\text{-}N_2C_{10}H_8)]\cdot(C_6H_{14})_2$	1.0	4 / 9	2478500
**2La**	$[\{La^{III}(NHAr^{iPr6})_2\}_2(\mu\text{-}N_2C_{10}H_8)]$	0.5	10 / 15	2478501
**2Tm**	$[\{Tm^{III}(NHAr^{iPr6})_2\}_2(\mu\text{-}N_2C_{10}H_8)]\cdot(C_9H_{12})$	0.5	1.5 / 9	2478502
**2Lu**	$[\{Lu^{III}(NHAr^{iPr6})_2\}_2(\mu\text{-}N_2C_{10}H_8)]\cdot(C_6H_{14})$	1.0	6 / 20	2478503
**3Y**	$[\{Y^{III}(NHAr^{iPr6})_2\}_2(\mu\text{-}N_2C_{10}H_{10})]$	1.0	2 / 5	2478504
**3La**	$[\{La^{III}(NHAr^{iPr6})_2\}_2(\mu\text{-}N_2C_{10}H_{10})]$	0.5	6 / 20	2478505
**3Tm**	$[\{Tm^{III}(NHAr^{iPr6})_2\}_2(\mu\text{-}N_2C_{10}H_{10})]\cdot(C_6H_{14})$	0.5	4.5 / 1	2478506
**3Lu**	$[\{Lu^{III}(NHAr^{iPr6})_2\}_2(\mu\text{-}N_2C_{10}H_{10})]\cdot(C_6H_{14})$	0.5	0.38 / 1.53	2478507
**4**	$[\{Y^{III}(NHAr^{iPr6})_2(\mu\text{-}N_2C_{10}H_8)\}_4]$	0.5	20/50	2478508

CrysAlisPro was used for the final unit cell determination, and parameters were refined from the observed positions of all strong reflections in each data set. An analytical absorption correction was applied. The Olex2[4] GUI was used for structure solution and refinement utilising the ShelX software packages.[5,6] The structures were solved using ShelXT[5]; the datasets were refined by ShelXL[6] using full-matrix least-squares on all unique F^2 values,

with anisotropic displacement parameters for all non-hydrogen atoms, and with constrained riding hydrogen geometries; U_{iso}(H) was set at 1.2 (1.5 for methyl groups if applicable) times *U*eq of the parent atom. The largest features in the final difference syntheses were close to heavy atoms and were of no chemical significance. Olex2[4] combined with POV-Ray,[5] and Gimp[6] were employed for molecular graphics. The following CCDC references contain the supplementary crystal data for this article: **2Y** (2478500), **2La** (2478501), **2Tm** (2478502), **2Lu** (2478503), **3Y** (2478504), **3La** (2478505), **3Tm** (2478506), **3Lu** (2478507), and **4** (2478508). These data can be obtained free of charge from the Cambridge Crystallographic Data Centre via www.ccdc.cam.ac.uk/data_request/cif.

The combined error from two individual metrics that have their own associated errors (estimated standard deviation, or standard uncertainty used interchangeably here) can be calculated as the root of the sum of the square of each error (**Eq. S2**). This is not strictly appropriate for combining more than two individual errors.[7]

$$\sigma_{tot} = \sqrt{{\sigma_1}^2 + {\sigma_2}^2}$$

Eq. S2.

The combined error for the numerical average for multiple (independent) bond lengths, such as the five independent M–C bonds in an M–(η^5-C_5H_5) complex, is calculated using the alternate weighted standard deviation from Parsons and Clegg (**Eq. S3**).[7]

$$\sigma_{tot} = 1/\sqrt{\textstyle\sum_{1\to n} W_n} \qquad W_n = 1/{\sigma_n}^2$$

Eq. S3.

Table S3. Crystallographic data for **2Y**, **2La**, and **2Tm**.

	**2Y**	**2La**	**2Tm**
Identification code	ccapg173	rcapg85	rcapg98
Formula	$C_{196}H_{306}N_6Y_2$	$C_{226}H_{304}N_6La_2$	$C_{226}H_{304}N_6Tm_2$
Fw	2924.37	3382.55	3442.59
Temperature / K	100.00(10)	100.00(10)	100.00(10)
Crystal system	monoclinic	orthorhombic	orthorhombic
Space group	$P2_1/c$	$Cmce$	$Cmc2_1$
a / Å	19.4868(12)	34.3078(3)	32.8841(6)
b / Å	23.7601(12)	30.3275(2)	29.7725(3)
c / Å	21.7768(10)	20.0786(2)	20.4027(3)
α / °	90	90	90
β / °	115.574(7)	90	90
γ / °	90	90	90
Volume / $Å^3$	9095.0(10)	20891.2(3)	19975.1(5)
Z	2	2	2
ρ_{calcg} / cm^3	1.068	1.075	1.145
μ / mm^{-1}	1.22	3.488	2.006
F(000)	3204	7264	7360
Crystal size / mm^3	0.194 × 0.133 × 0.081	0.325 × 0.277 × 0.201	0.342 × 0.261 × 0.137
Radiation	Cu Kα (λ = 1.54184)	Cu Kα (λ = 1.54184)	Cu Kα (λ = 1.54184)
2Θ range / °	5.838 to 124.772	5.874 to 158.044	4.004 to 160.002
Index ranges	–22 ≤ h ≤ 22, –27 ≤ k ≤ 27, –16 ≤ l ≤ 25	-43 ≤ h ≤ 42, –14 ≤ k ≤ 37, –25 ≤ l ≤ 24	-41 ≤ h ≤ 41, -37 ≤ k ≤ 20, –25 ≤ l ≤ 22
No. reflections	63003	44293	40204
Unique reflections	14441 [R_{int} = 0.0882, R_{sigma} = 0.1023]	11117 [R_{int} = 0.0241, R_{sigma} = 0.0228]	16534 [R_{int} = 0.0316, R_{sigma} = 0.0389]
Data / restraints / parameters	14441/4839/1605	11117/593/382	16534/1709/1137
GOOF on F^2	1.053	0.976	0.995
Final R indexes [I ≥ 2σ (I)][a]	R_1 = 0.0994, wR_2 = 0.2436	R_1 = 0.0429, wR_2 = 0.1338	R_1 = 0.0661, wR_2 = 0.1755
Final R indexes [all data]	R_1 = 0.1461, wR_2 = 0.2718	R_1 = 0.0490, wR_2 = 0.1398	R_1 = 0.0759, wR_2 = 0.1846
Largest diff. (peak / hole) / e $Å^{-3}$	0.89/–0.56	0.93/–0.41	3.26/–1.01

[a] $R = \sum||F_O| - |F_C||/\sum|F_O|$; $R_W = [\sum w(F_O^2 - F_C^2)^2/\sum w(F_O^2)^2]^{0.5}$; $S = [\sum w(F_O^2 - F_C^2)^2/(\text{no. data} - \text{no. params})]^{0.5}$ for all data.

Table S4. Crystallographic data for **2Lu**, **3Y**, and **3La**.

	**2Lu**	**3Y**	**3La**
Identification code	rcapg89	ccapg171	rcapg165
Formula	$C_{184}H_{238}N_6Lu_2$	$C_{172}H_{252.1}N_6Y_2$	$C_{174}H_{256.67}N_6La_2$
Fw	2883.82	2581.68	2710.33
Temperature / K	100.00(10)	100.00(10)	99.98(10)
Crystal system	monoclinic	monoclinic	triclinic
Space group	$P2_1/c$	$P2_1/n$	$P\bar{1}$
a / Å	21.72601(10)	22.55645(10)	19.8982(2)
b / Å	13.68984(5)	22.30764(10)	21.7381(2)
c / Å	28.92770(14)	31.33272(14)	22.6385(2)
α / °	90	90	63.1280(10)
β / °	110.1294(5)	102.8007(4)	64.7740(10)
γ / °	90	90	76.4950(10)
Volume / Å^3	8078.29(7)	15374.19(12)	7891.72(16)
Z	2	2	2
ρ_{calcg} / cm^3	1.186	1.115	1.141
μ / mm^{-1}	2.667	1.384	4.497
F(000)	3052	5615	2913
Crystal size / mm^3	0.062 × 0.058 × 0.04	0.216 × 0.194 × 0.091	0.33 × 0.206 × 0.091
Radiation	Cu Kα (λ = 1.54184)	Cu Kα (λ = 1.54184)	Cu Kα (λ = 1.54184)
2Θ range / °	4.332 to 158.102	5.642 to 160.654	4.562 to 156.246
Index ranges	–27 ≤ h ≤ 27, –17 ≤ k ≤ 16, -36 ≤ l ≤ 36	–28 ≤ h ≤ 28, –27 ≤ k ≤ 28, -39 ≤ l ≤ 40	–25 ≤ h ≤ 25, –27 ≤ k ≤ 27, –27 ≤ l ≤ 28
No. reflections	128299	147295	119140
Unique reflections	17188 [R_{int} = 0.0356, R_{sigma} = 0.0215]	33112 [R_{int} = 0.0170, R_{sigma} = 0.0355]	33011 [R_{int} = 0.0239, R_{sigma} = 0.0299]
Data / restraints / parameters	17188/126/913	33112/716/1653	33011/6010/2181
GOOF on F^2	1.077	1.061	1.046
Final R indexes [I ≥ 2σ (I)][a]	R_1 = 0.0290, wR_2 = 0.0721	R_1 = 0.0455, wR_2 = 0.1063	R_1 = 0.0418, wR_2 = 0.1125
Final R indexes [all data]	R_1 = 0.0328, wR_2 = 0.0738	R_1 = 0.0552, wR_2 = 0.1111	R_1 = 0.0462, wR_2 = 0.1157
Largest diff. (peak / hole) / e Å^{-3}	1.07/–0.91	0.39/–0.58	1.86/–0.85

[a] $R = \sum||F_O| - |F_C||/\sum|F_O|$; $R_W = [\sum w(F_O^2 - F_C^2)^2/\sum w(F_O^2)^2]^{0.5}$; $S = [\sum w(F_O^2 - F_C^2)^2/(\text{no. data} - \text{no. params})]^{0.5}$ for all data.

Table S5. Crystallographic data for **3Tm**, **3Lu**, and **4**.

	**3Tm**	**3Lu**	**4**
Identification code	lcapg83	ccapg155	rcapg146
Formula	$C_{172}H_{252}N_6Tm_2$	$C_{166}H_{237}N_6Lu_2$	$C_{416}H_{652}N_{16}O_{22}Y_4$
Fw	2741.64	2666.54	6585.14
Temperature / K	100.0(4)	99.9(4)	100.00(12)
Crystal system	triclinic	monoclinic	cubic
Space group	$P\bar{1}$	$P2_1$	$I\bar{4}3d$
a / Å	17.8032(2)	15.1114(3)	49.4285(2)
b / Å	21.5845(5)	25.1229(5)	49.4285(2)
c / Å	22.3123(5)	20.3887(4)	49.4285(2)
α / °	71.411(2)	90	90
β / °	89.6694(15)	107.732(2)	90
γ / °	74.5806(16)	90	90
Volume / $Å^3$	7804.9(3)	7372.6(3)	120762.3(16)
Z	2	2	12
$\rho_{calc}g$ / cm^3	1.167	1.201	1.087
μ / mm^{-1}	2.441	2.879	1.197
F(000)	2928	2834	43104
Crystal size / mm^3	0.198 × 0.085 × 0.044	0.208 × 0.103 × 0.07	0.082 × 0.065 × 0.036
Radiation	Cu Kα (λ = 1.54184)	Cu Kα (λ = 1.54184)	Cu Kα (λ = 1.54184)
2Θ range / °	4.194 to 153.272	5.752 to 165.132	4.378 to 106.814
Index ranges	–22 ≤ h ≤ 22, –27 ≤ k ≤ 26, –27 ≤ l ≤ 28	–19 ≤ h ≤ 18, -31 ≤ k ≤ 31, –26 ≤ l ≤ 25	–30 ≤ h ≤ 51, –46 ≤ k ≤ 51, –44 ≤ l ≤ 51
No. reflections	122786	60421	64707
Unique reflections	31868 [R_{int} = 0.0535, R_{sigma} = 0.0471]	27745 [R_{int} = 0.0212, R_{sigma} = 0.0428]	11926 [R_{int} = 0.0193, R_{sigma} = 0.0315]
Data / restraints / parameters	31868/0/1583	27745/2236/1965	11926/1582/1055
GOOF on F^2	1.027	1.05	1.032
Final R indexes [I ≥ 2σ (I)][a]	R1 = 0.0421, wR2 = 0.1019	R1 = 0.0321, wR2 = 0.0749	R1 = 0.0406, wR2 = 0.1000
Final R indexes [all data]	R1 = 0.0485, wR2 = 0.1045	R1 = 0.0341, wR2 = 0.0772	R1 = 0.0491, wR2 = 0.1044
Largest diff. (peak / hole) / e $Å^{-3}$	1.03/–1.19	0.79/–0.84	0.24/–0.13

[a] $R = \sum||F_O| - |F_C||/\sum|F_O|$; $R_W = [\sum w(F_O^2 - F_C^2)^2/\sum w(F_O^2)^2]^{0.5}$; $S = [\sum w(F_O^2 - F_C^2)^2/(\text{no. data} - \text{no. params})]^{0.5}$ for all data.

S3. Molecular structures

*Complexes **2M** (M = Y, La, Tm, Lu)*

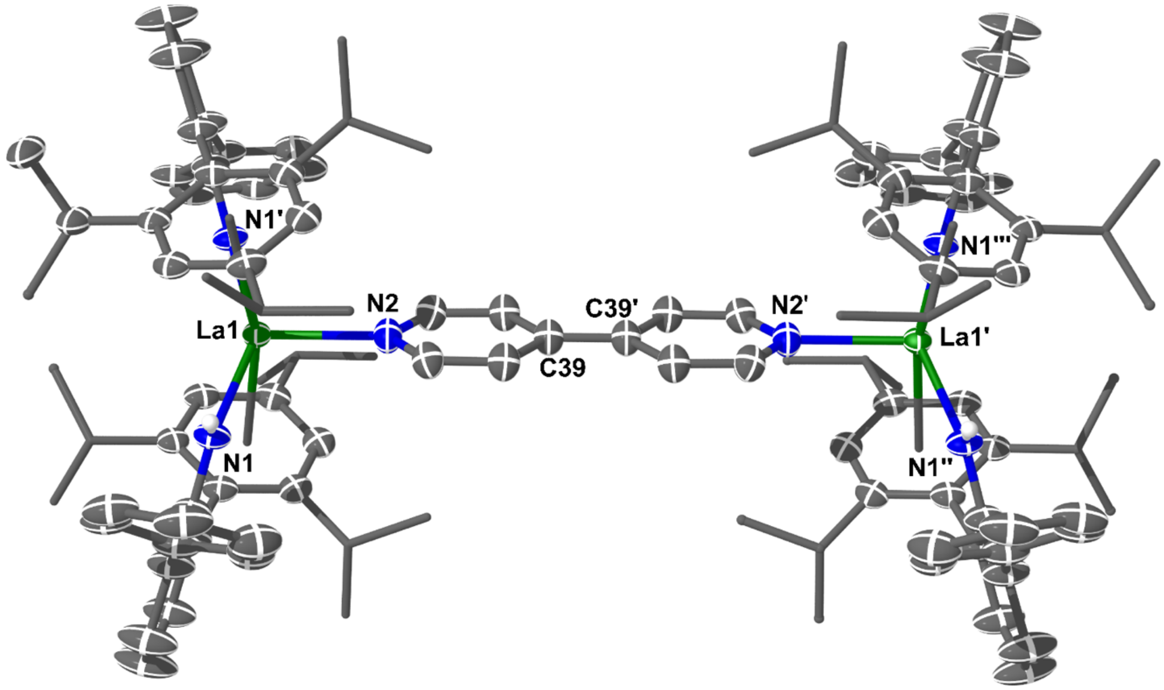

Figure S1. Molecular structure of **2La**. Ellipsoids set at 30% probability. H–atoms except those on N-H groups are omitted for clarity, along with lattice solvents and ligand isopropyl groups (operations: X, Y, Z; 1−X, 1−Y, 1−Z; +X, 1−Y, 1−Z; 1−X, +Y, +Z).

La(1)–N(1) = 2.436(2) Å, La(1)–N(2) = 2.336(3) Å, La(1)–C_{range} = 3.075(2)–3.400(3) Å, La(1)···$C_{6\text{-centroid}}$ = 2.9068(9) Å, C(39)–C(39′) = 1.389(8) Å, N(1)–La(1)–N(1’) = 149.93(12)°, N(1)–La(1)–N(2) = 105.03(6)°, $C_{6\text{-centroid}}$···La(1)···$C_{6\text{-centroid}}$ = 167.16(5)°.

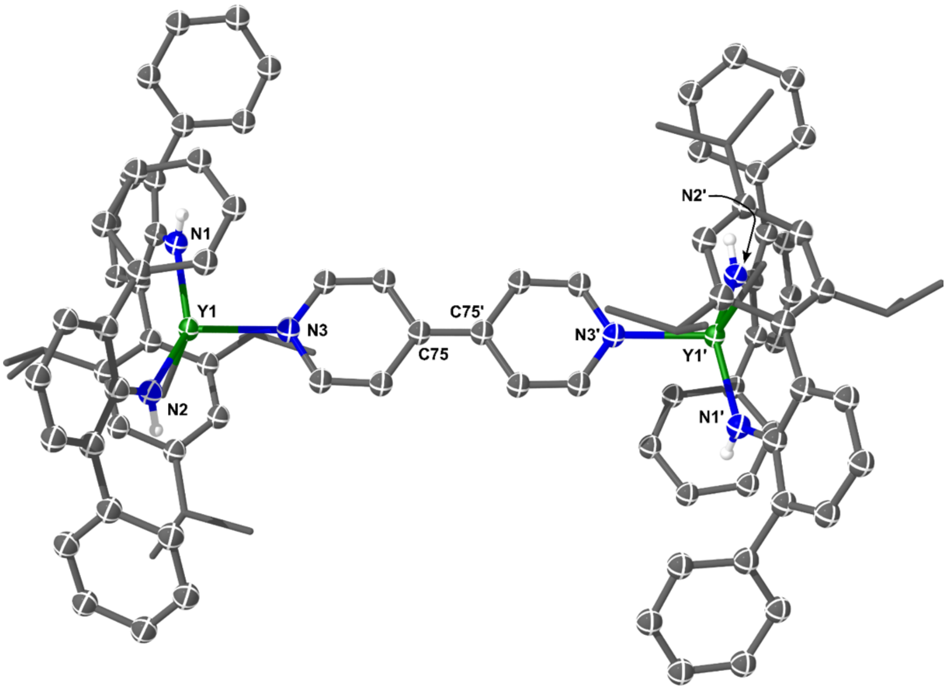

Figure S2. Molecular structure of **2Y**. Ellipsoids set at 50% probability. H–atoms except those on N-H groups are omitted for clarity, along with lattice solvents and ligand isopropyl groups (operations: X, Y, Z; 1−X, 1−Y, 1−Z).

Y(1)–N(1) = 2.275(6) Å, Y(1)–N(2) = 2.225(6) Å, Y(1)–N(3) = 2.211(7) Å, Y(1)–C_{range} = 2.769(7)–3.146(10) Å, Y(1)⋯$C_{6\text{-centroid}}$ = 2.602(4) Å, C(75)–C(75′) = 1.355(18) Å, N(1)–Y(1)–N(2) = 145.9(2)°, N(1)–Y(1)–N(3) = 101.2(3)°, N(2)–Y(1)–N(3) = 105.7(3)°.

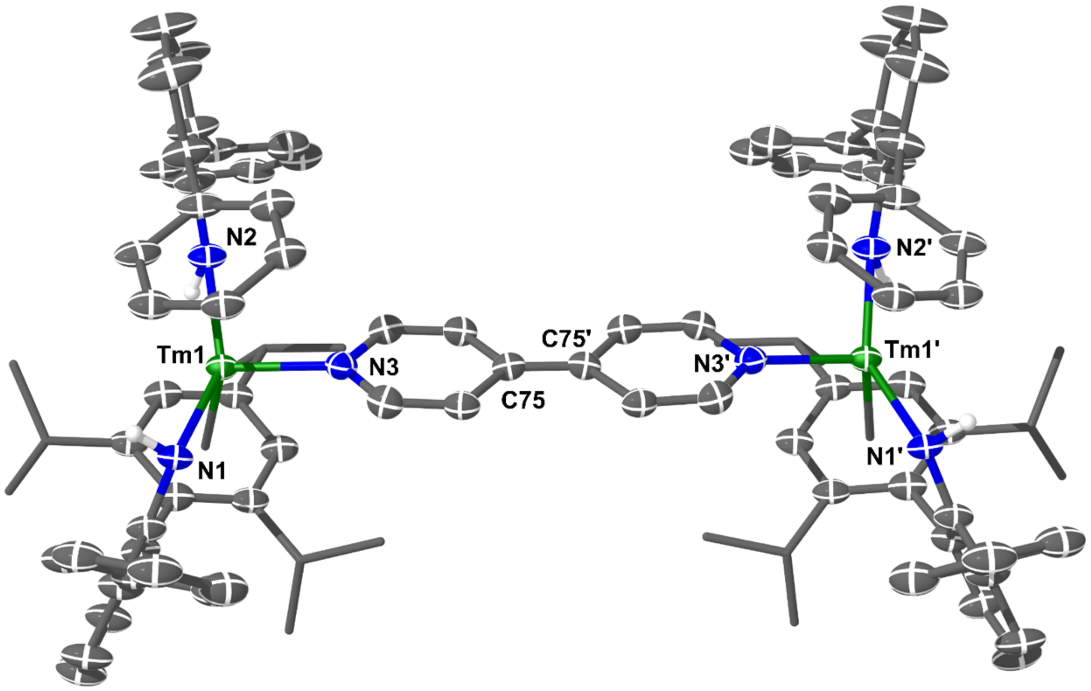

Figure S3. Molecular structure of **2Tm**. Ellipsoids set at 50% probability. H–atoms except those on N-H groups are omitted for clarity, along with lattice solvents and ligand isopropyl groups. A second molecule of **2Tm** has also been omitted (operations: X, Y, Z; 1−X, +Y, +Z).

Tm(1)–N(1) = 2.225(7) Å, Tm(1)–N(2) = 2.271(4) Å, Tm(1)–N(3) = 2.174(5) Å, Tm(1)–C_{range} = 2.729(7)–3.050(9) Å, Tm(1)···$C_{6\text{-centroid}}$ = 2.540(5) Å, C(75)–C(75') = 1.323(11) Å, N(1)–Tm(1)–N(2) = 146.0(3)°, N(1)–Tm(1)–N(3) = 103.4(3)°, N(2)–Tm(1)–N(3) = 100.43(18)°.

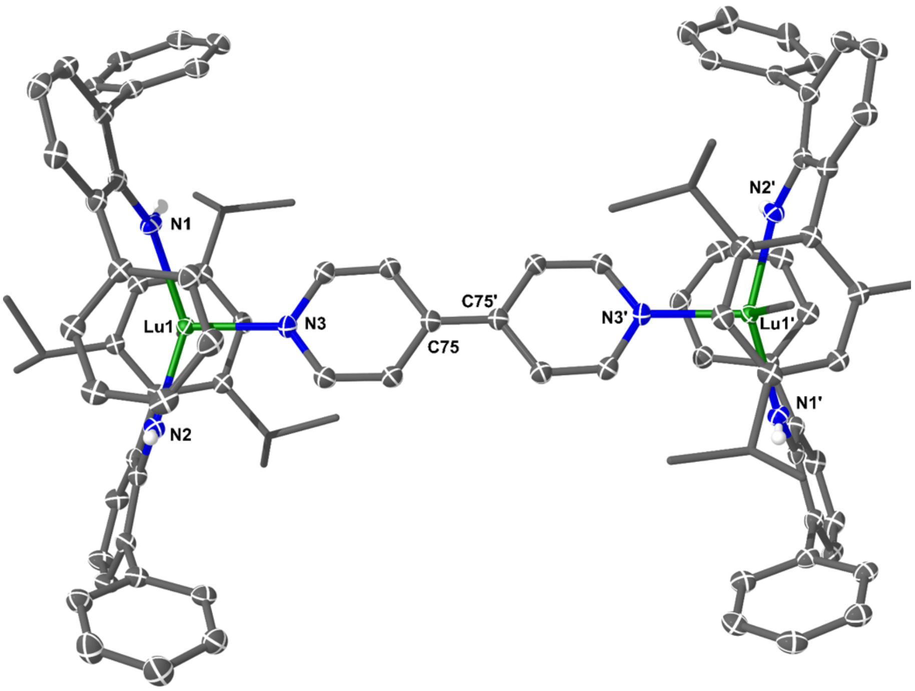

Figure S4. Molecular structure of **2Lu**. Ellipsoids set at 50% probability. H–atoms except those on N-H groups are omitted for clarity, along with lattice solvents and ligand isopropyl groups (operations: X, Y, Z; 1−X, 1−Y, 1−Z).

Lu(1)–N(1) = 2.2102(16) Å, Lu(1)–N(2) = 2.2010(16) Å, Lu(1)–N(3) = 2.1613(16) Å, Lu(1)–C_{range} = 2.6991(19)–2.9817(18) Å, Lu(1)···$C_{6\text{-centroid}}$ = 2.4674(10) Å, C(75)–C(75') = 1.368(4) Å, N(1)–Lu(1)–N(2) = 139.15(6)°, N(1)–Tm(1)–N(3) = 102.06(6)°, N(2)–Lu(1)–N(3) = 104.87(6)°.

*Complexes **3M** (M = Y, La, Tm, Lu)*

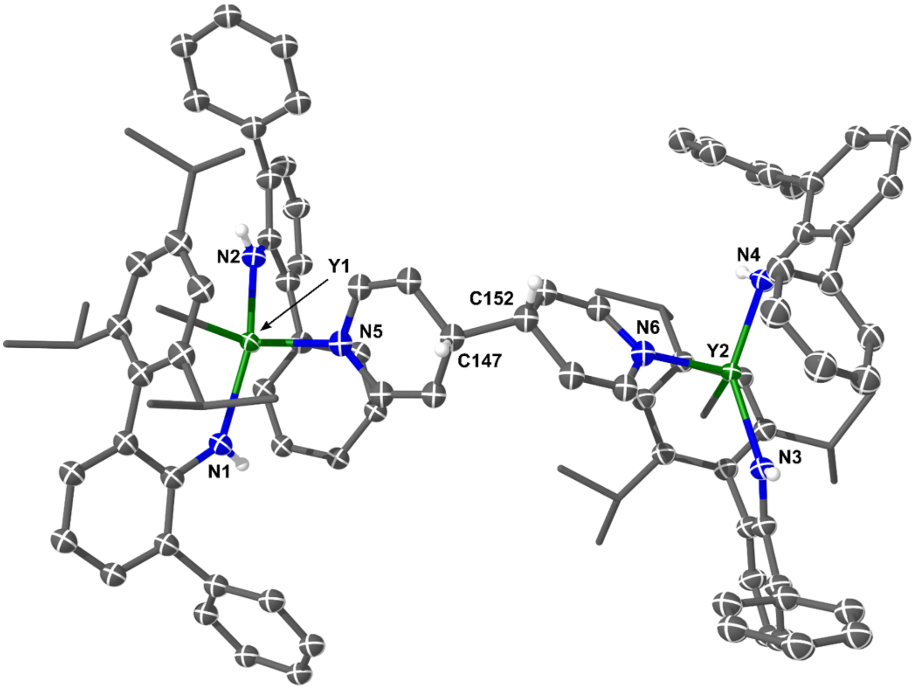

Figure S5. Molecular structure of **3Y**. Ellipsoids set at 50% probability. H–atoms except those on N-H groups are omitted for clarity, along with lattice solvents and ligand isopropyl groups (operations: X, Y, Z).

Y(1)–N(1) = 2.2472(15) Å, Y(1)–N(2) = 2.2880(15) Å, Y(1)–N(5) = 2.2058(17) Å, Y(2)–N(3) = 2.2795(16) Å, Y(2)–N(4) = 2.2557(16) Å, Y(2)–N(6) = 2.1868(17) Å, Y(1)–C_{range} = 2.800(2)–3.151(2) Å, Y(1)···$C_{6\text{-centroid}}$ = 2.6196(9) Å, Y(2)–C_{range} = 2.805(2)–3.089(2) Å, Y(2)···$C_{6\text{-centroid}}$ = 2.5864(11) Å, C(147)–C(152) = 1.552(3) Å, N(1)–Y(1)–N(2) = 145.48(6)°, N(1)–Y(1)–N(5) = 106.61(6)°, N(2)–Y(1)–N(5) = 101.32(6)°, N(3)–Y(2)–N(4) = 142.60(7)°, N(3)–Y(2)–N(6) = 103.98(6)°, N(4)–Y(2)–N(6) = 104.45(6)°, N(5)···C(147)–C(152)···N(6) torsion angle = 59.0(3)°.

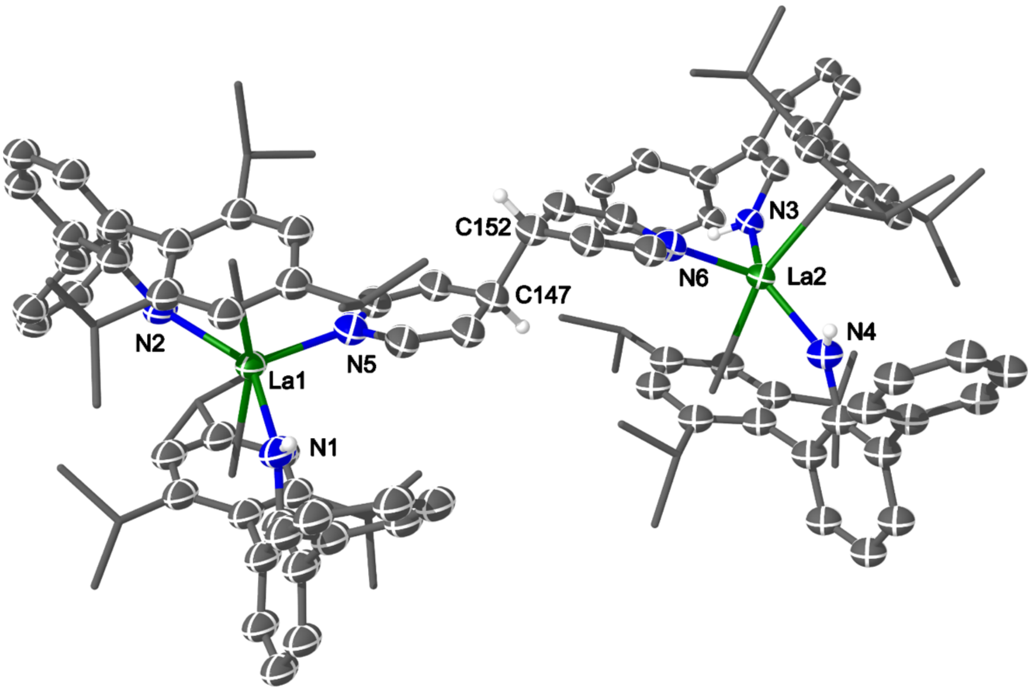

Figure S6. Molecular structure of **2La**. Ellipsoids set at 30% probability. H–atoms except those on N-H groups are omitted for clarity, along with lattice solvents and ligand isopropyl groups (operations: X, Y, Z).

La(1)–N(1) = 2.365(3) Å, La(1)–N(2) = 2.419(15) Å, La(1)–N(5) = 2.361(15) Å, La(2)–N(3) = 2.459(2) Å, La(2)–N(4) = 2.4313(19) Å, La(2)–N(6) = 2.337(2) Å, La(1)–C_{range} = 2.98(2)–3.332(11) Å, La(1)–C_{range} = 3.024(3)–3.354(3) Å, La(1)···$C_{6\text{-centroid}}$ = 2.851(6) Å, La(1)···$C_{6\text{-centroid}}$ = 2.881(3) Å, La(2)–C_{range} = 3.066(2)–3.339(3) Å, La(2)–C_{range} = 3.079(2)–3.391(2) Å, La(2)···$C_{6\text{-centroid}}$ = 2.8726(11) Å, La(2)···$C_{6\text{-centroid}}$ = 2.909(1) Å, C(147)–C(152) = 1.558(11) Å, N(1)–La(1)–N(2) = 153.6(6)°, N(1)–La(1)–N(5) = 109.4(5)°, N(2)–La(1)–N(5) = 96.9(7)°, N(3)–La(2)–N(4) = 143.66(7)°, N(3)–La(2)–N(6) = 107.45(8)°, N(4)–La(2)–N(6) = 108.84(8)°, N(5)···C(147)–C(152)···N(6) torsion angle = 173.9(14)°.

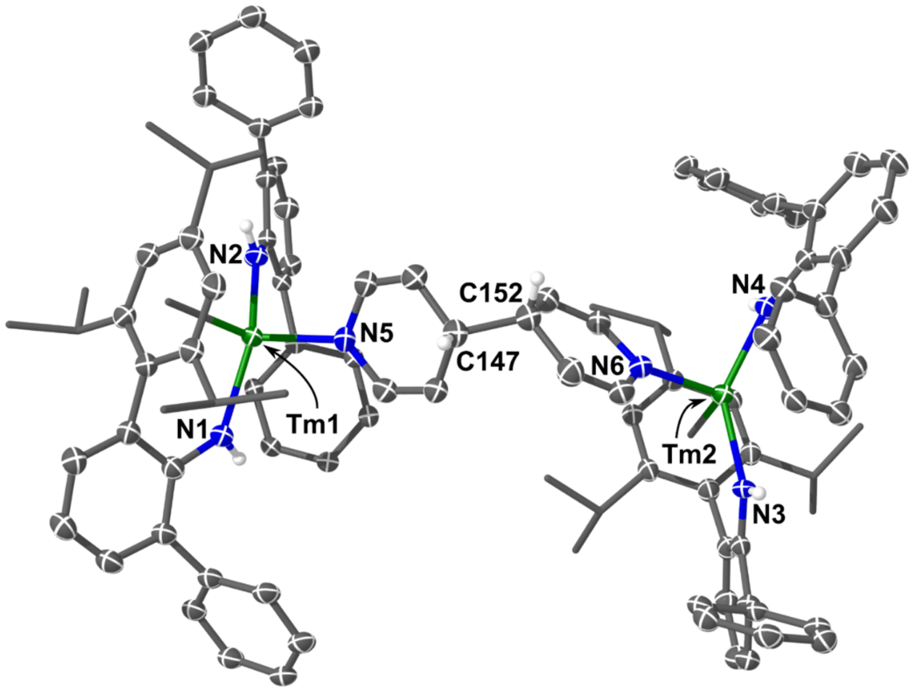

Figure S7. Molecular structure of **3Tm**. Ellipsoids set at 50% probability. H–atoms except those on N-H groups are omitted for clarity, along with lattice solvents and ligand isopropyl groups (operations: X, Y, Z).

Tm(1)–N(1) = 2.214(2) Å, Tm(1)–N(2) = 2.244(2) Å, Tm(1)–N(5) = 2.175(2) Å, Tm(2)–N(3) = 2.207(2) Å, Tm(2)–N(4) = 2.254(2) Å, Tm(2)–N(6) = 2.190(2) Å, Tm(1)–C_{range} = 2.754(3)–3.038(3) Å, Tm(1)···$C_{6\text{-centroid}}$ = 2.5296(13) Å, Tm(2)–C_{range} = 2.745(3)–3.092(3) Å, Tm(2)···$C_{6\text{-centroid}}$ = 2.5506(10) Å, C(147)–C(152) = 1.560(4) Å, N(1)–Tm(1)–N(2) = 138.76(9)°, N(1)–Tm(1)–N(5) = 103.81(9)°, N(2)–Tm(1)–N(5) = 104.80(8)°, N(3)–Tm(2)–N(4) = 141.73(8)°, N(3)–Tm(2)–N(6) = 105.07(8)°, N(4)–Tm(2)–N(6) = 100.50(8)°, N(5)···C(147)–C(152)···N(6) torsion angle = 63.1(4)°.

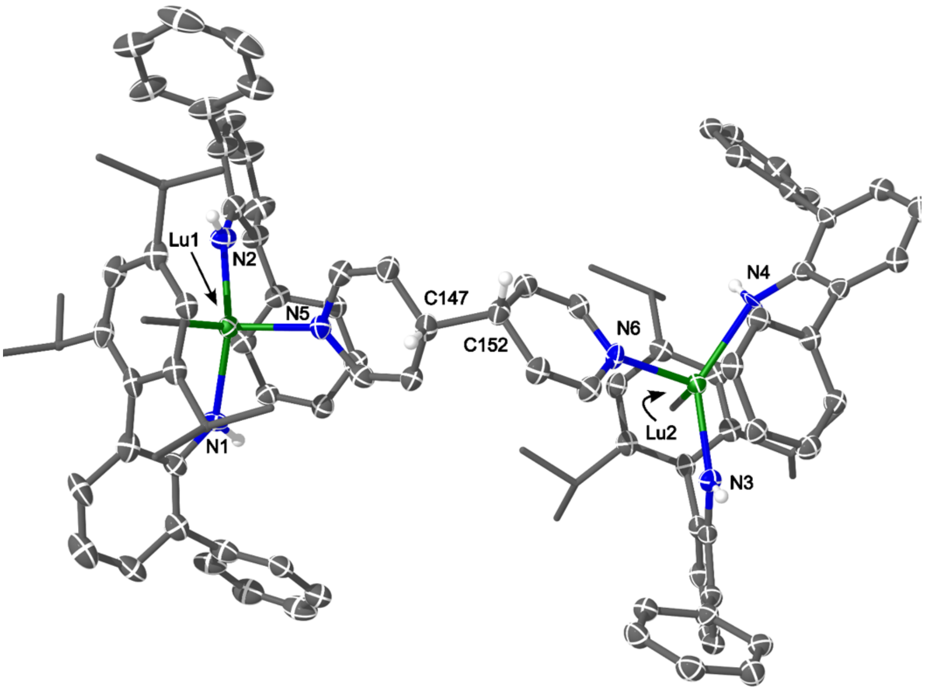

Figure S8. Molecular structure of **3Lu**. Ellipsoids set at 50% probability. H–atoms except those on N-H groups are omitted for clarity, along with lattice solvents and ligand isopropyl groups (operations: X, Y, Z).

Lu(1)–N(1) = 2.210(4) Å, Lu(1)–N(2) = 2.198(3) Å, Lu(1)–N(5) = 2.164(4) Å, Lu(2)–N(3) = 2.211(4) Å, Lu(2)–N(4) = 2.188(4) Å, Lu(2)–N(6) = 2.146(4) Å, Lu(1)–C_{range} = 2.713(5)–2.979(5) Å, Lu(1)···$C_{6\text{-centroid}}$ = 2.473(2) Å, Lu(2)–C_{range} = 2.712(5)–2.995(5) Å, Lu(2)···$C_{6\text{-centroid}}$ = 2.479(2) Å, C(147)–C(152) = 1.541(7) Å, N(1)–Lu(1)–N(2) = 137.57(14)°, N(1)–Lu(1)–N(5) = 101.49(15)°, N(2)–Lu(1)–N(5) = 106.06(15)°, N(3)–Lu(2)–N(4) = 137.98(16)°, N(3)–Lu(2)–N(6) = 103.45(16)°, N(4)–Lu(2)–N(6) = 105.9(2)°, N(5)···C(147)–C(152)···N(6) torsion angle = 60.9(6)°.

*Bond metrics for **2M** (M = Y, La, Tm, Lu) and **3M** (M = Y, La, Tm, Lu)*

Table S6. Bond lengths (Å) and angles (°) for **2M** (M = Y, La, Tm, Lu).

	**2Y**	**2La**	**2Tm**	**2Lu**
M(1)–N(1)	2.275(6)	2.436(2)	2.225(7)	2.2102(16)
M(1)–N(2)	2.225(6)	2.336(3)[B]	2.271(4)	2.2010(16)
M(1)–N(3)	2.211(7)	[A]	2.174(5)	2.1613(16)
M(1)–C_{range}	2.769(7)–3.146(10)	3.075(2)–3.400(3)	2.729(7)–3.050(9)	2.6991(19)–2.9817(18)
M(1)···$C_{6\text{-centroid}}$	2.602(4)	2.9068(9)	2.540(5)	2.4674(10)
C(75)–C(75′)	1.355(18)	–	1.323(11)	1.368(4)
C(39)–C(39′)	–	1.389(8)	–	
N(1)–M(1)–N(2)	145.9(2)	149.93(12)	146.0(3)	139.15(6)
N(1)–M(1)–N(3)	101.2(3)	105.03(6)	103.4(3)	102.06(6)
N(2)–M(1)–N(3)	105.7(3)	105.03(6)	100.43(18)	104.87(6)

[A] The solid-state shows a quarter-molecule in the asymmetric unit cell. [B] For **2La**, N(2) refers to the La–N_{bipy} bond length.

Table S7. Bond lengths (Å) and angles (°) for **3M** (M = Y, La, Tm, Lu).

	**3Y**	**3La**	**3Tm**	**3Lu**
M(1)–N(1)	2.2472(15)	2.365(3)	2.214(2)	2.210(4)
M(1)–N(2)	2.2880(15)	2.419(15)	2.215(3)	2.198(3)
M(1)–N(5)	2.2058(17)	2.361(15)	2.175(2)	2.164(4)
M(2)–N(3)	2.2795(16)	2.459(2)	2.207(2)	2.211(4)
M(2)–N(4)	2.2557(16)	2.4313(19)	2.254(2)	2.188(4)
M(2)–N(6)	2.1868(17)	2.340(2)	2.190(2)	2.146(4)
M(1)–C_{range}	2.800(2)–3.151(2)	2.98(2)–3.332(11)<br>3.024(3)–3.354(3)	2.754(3)–3.038(3)	2.713(5)–2.979(5)
M(1)···$C_{6\text{-centroid}}$	2.6196(9)	2.851(6)<br>2.881(3)	2.5296(13)	2.473(2)
M(2)–C_{range}	2.805(2)–3.089(2)	3.069(2)–3.339(3)<br>3.079(2)–3.391(2)	2.745(3)–3.092(3)	2.712(5)–2.995(5)
M(2)···$C_{6\text{-centroid}}$	2.5501(12)	2.8726(11)<br>2.909(1)	2.5506(10)	2.479(2)
C(147)–C(152)	1.564(5)	1.558(11)	1.560(4)	1.541(7)
N(1)–M(1)–N(2)	145.48(6)	153.6(6)	138.76(9)	137.57(14)
N(1)–M(1)–N(5)	106.61(6)	109.4(5)	103.81(9)	101.49(15)
N(2)–M(1)–N(5)	101.32(6)	96.9(7)	104.80(8)	106.06(15)
N(3)–M(2)–N(4)	142.60(7)	143.66(7)	141.73(8)	137.98(16)
N(3)–M(2)–N(6)	103.98(6)	107.45(8)	105.07(8)	103.45(16)
N(4)–M(2)–N(6)	104.45(6)	108.84(8)	100.50(8)	102.55(16)
N···C–C···N torsion angle	59.0(3)	173.9(14)	63.1(4)	60.9(6)

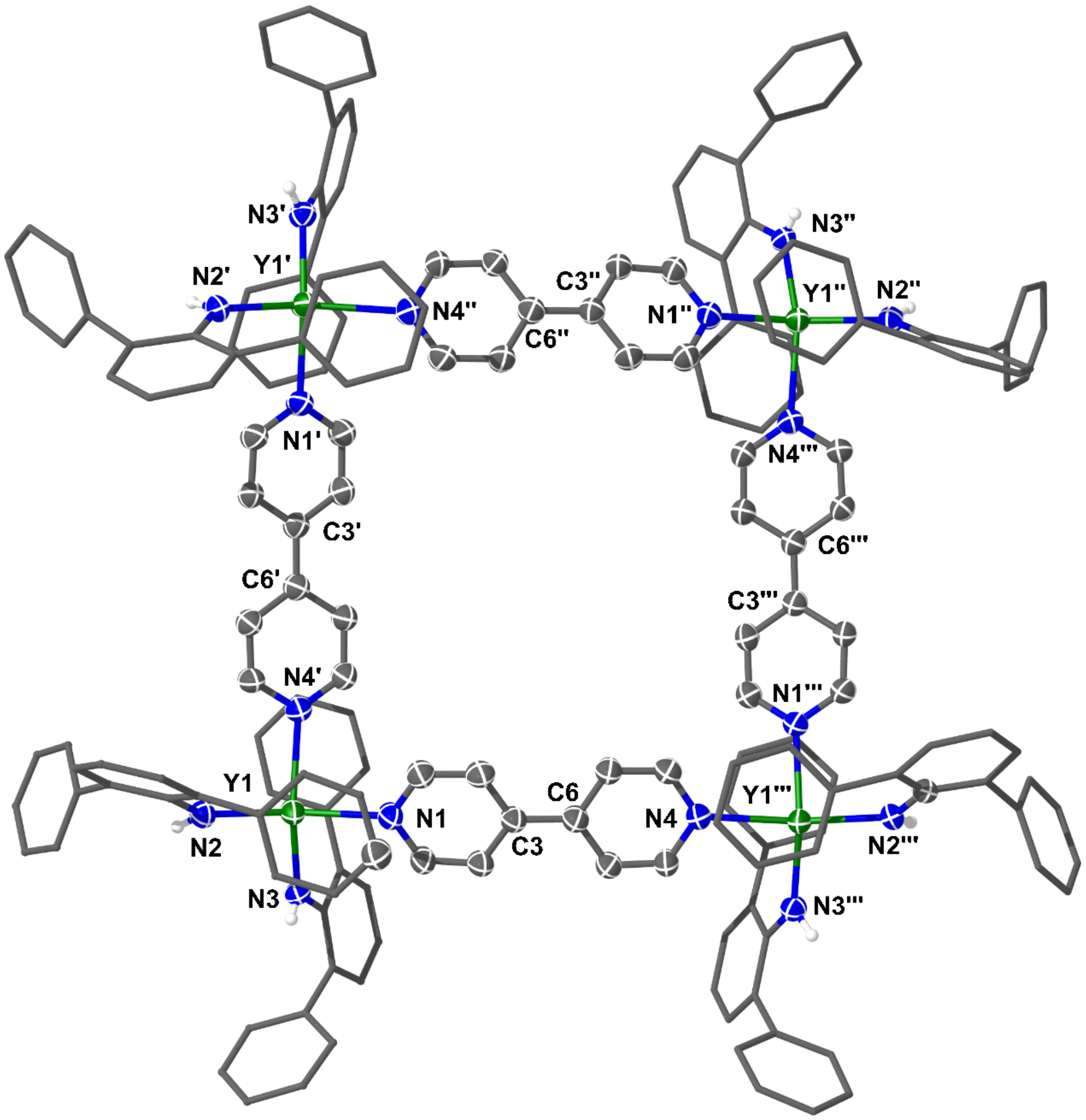

Figure S9. Molecular structure of **4**. Ellipsoids set at 50% probability. H–atoms except those on N-H groups are omitted for clarity, along with lattice solvents and ligand isopropyl groups (operations: X, Y, Z; ¾–X, –¼+Z, ¼–Y; +X, –Y, ½–Z; ¾–X, ¼–Z, ¼–Y).

Y(1)–N(1) = 2.389(4) Å, Y(1)–N(2) = 2.218(4) Å, Y(1)–N(3) = 2.213(4) Å, Y(1)–N(4′) = 2.377(4) Å, C(3)–C(6) = 1.419(7) Å, N(1)–Y(1)–N(2) = 151.25(17)°, N(1)–Y(1)–N(3) = 93.28(15)°, N(1)–Y(1)–N(4) = 88.67(15)°, N(2)–Y(1)–N(3) = 102.27(15)°, N(2)–Y(1)–N(4) = 93.69(15)°, N(3)–Y(2)–N(4) = 141.56(17)°, Torsion angle along C(3)–C(6) = 7.8(2)°.

S4. NMR spectroscopy plots

Broad features present in the ^1H NMR spectra below are attributed to fluxionality of the eight Tripp rings in solution.

*NMR spectra of **2M** (M = Y, La, Tm, Lu)*

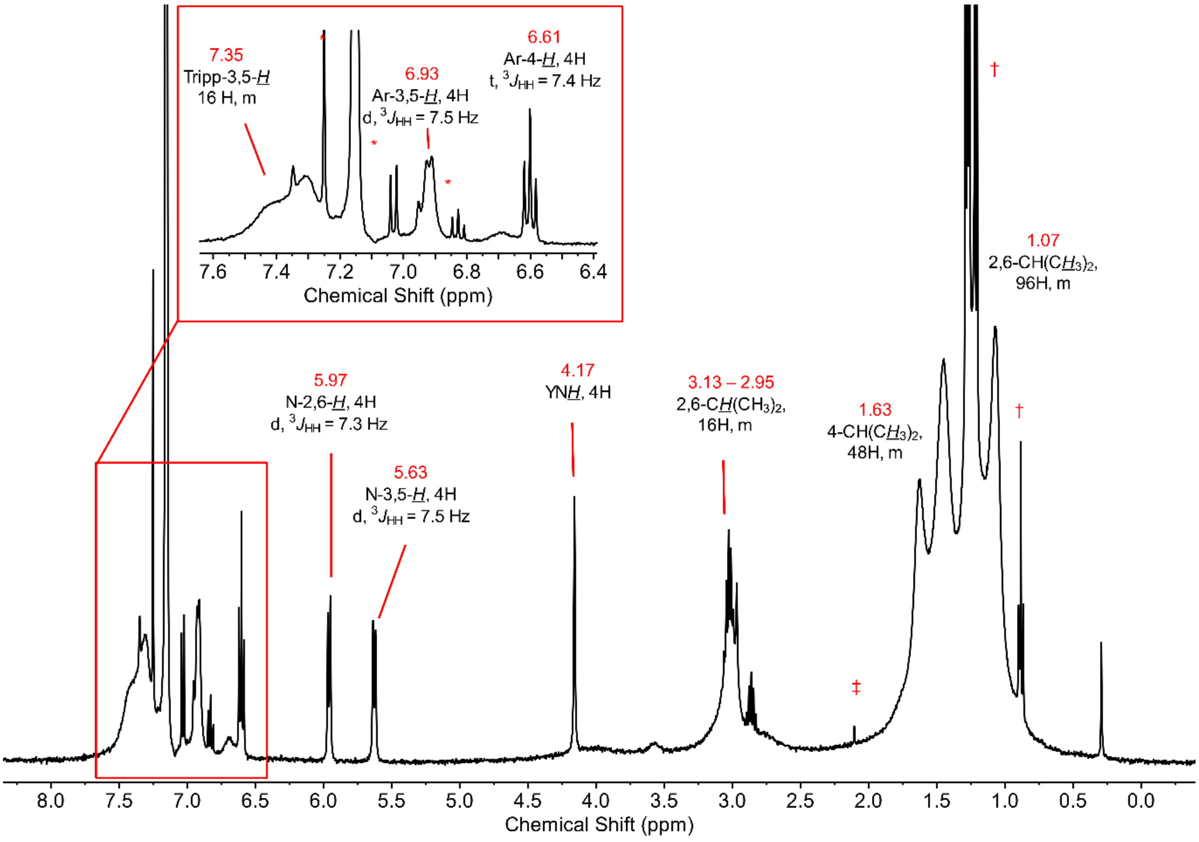

Figure S10. ^1H NMR spectrum of **2Y** in d_6-benzene. ‡ and † denote residual toluene and *n*-hexane respectively, * denotes protic ligand (H_2NAr^{iPr6}).

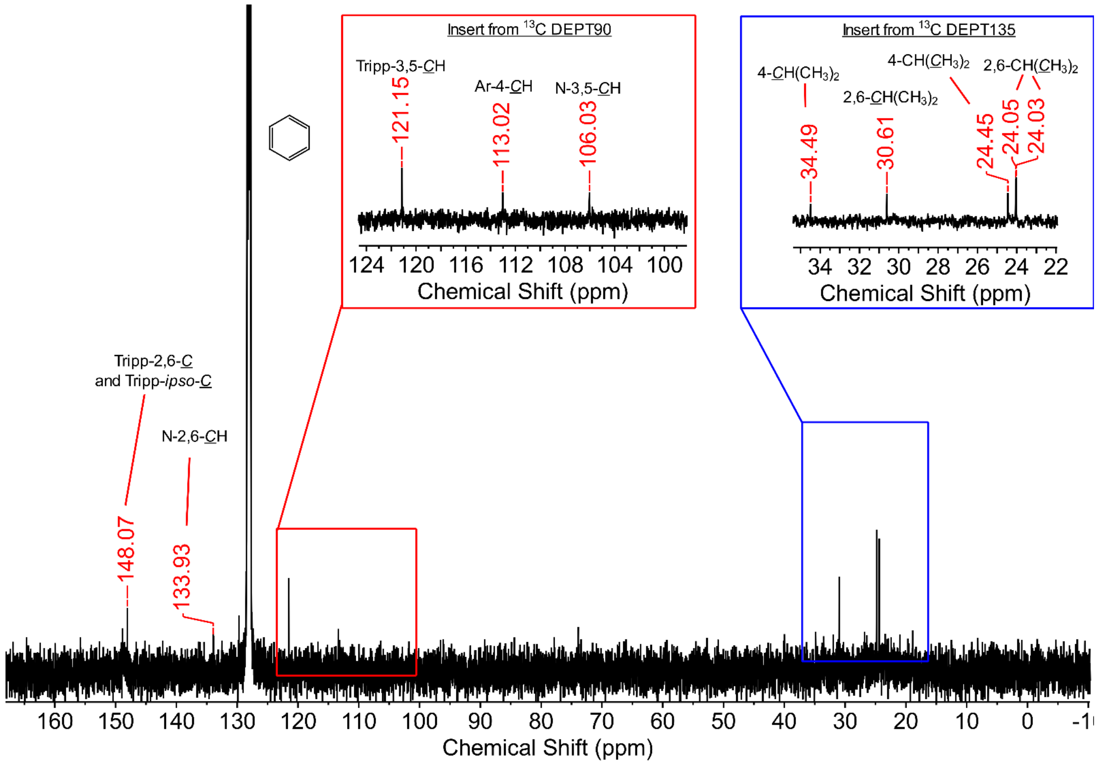

Figure S11. $^{13}C\{^{1}H\}$ NMR spectrum of **2Y** in d_6-benzene. ‡ and † denote residual toluene and *n*-hexane respectively, * denotes protic ligand (H_2NAr^{iPr6}).

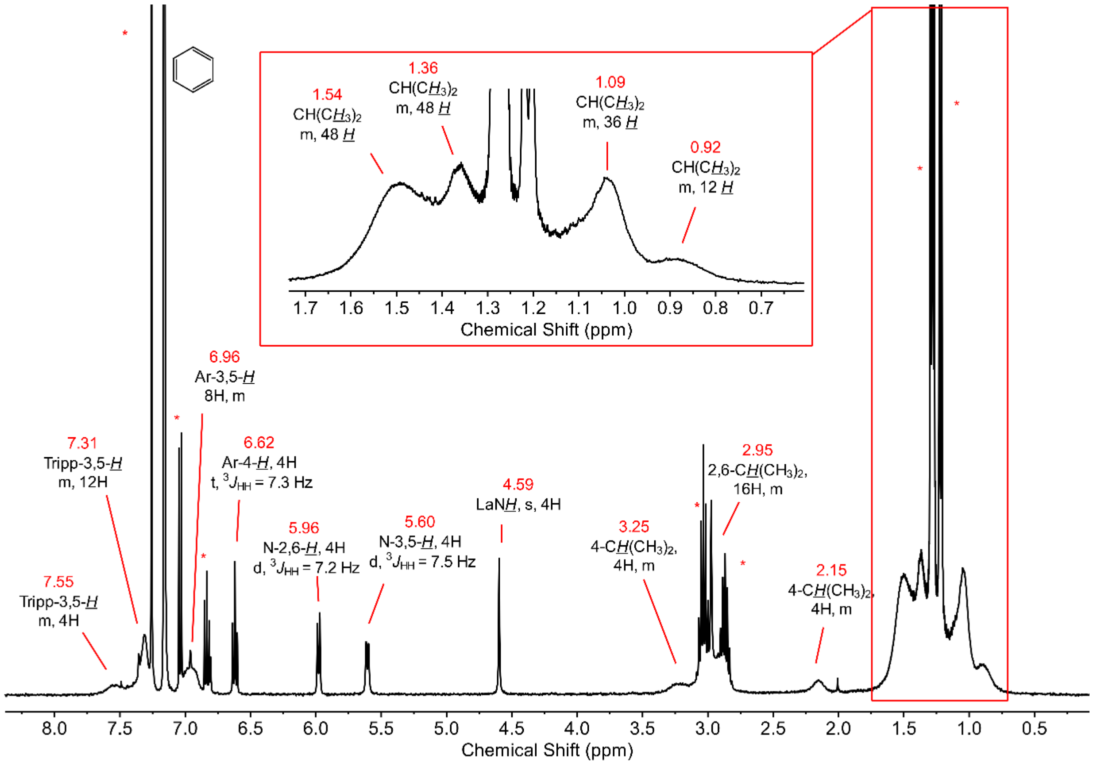

Figure S12. ^{1}H NMR spectrum of **2La** in d_6-benzene. ‡ and † denote residual toluene and *n*-hexane respectively, * denotes protic ligand (H_2NAr^{iPr6}).

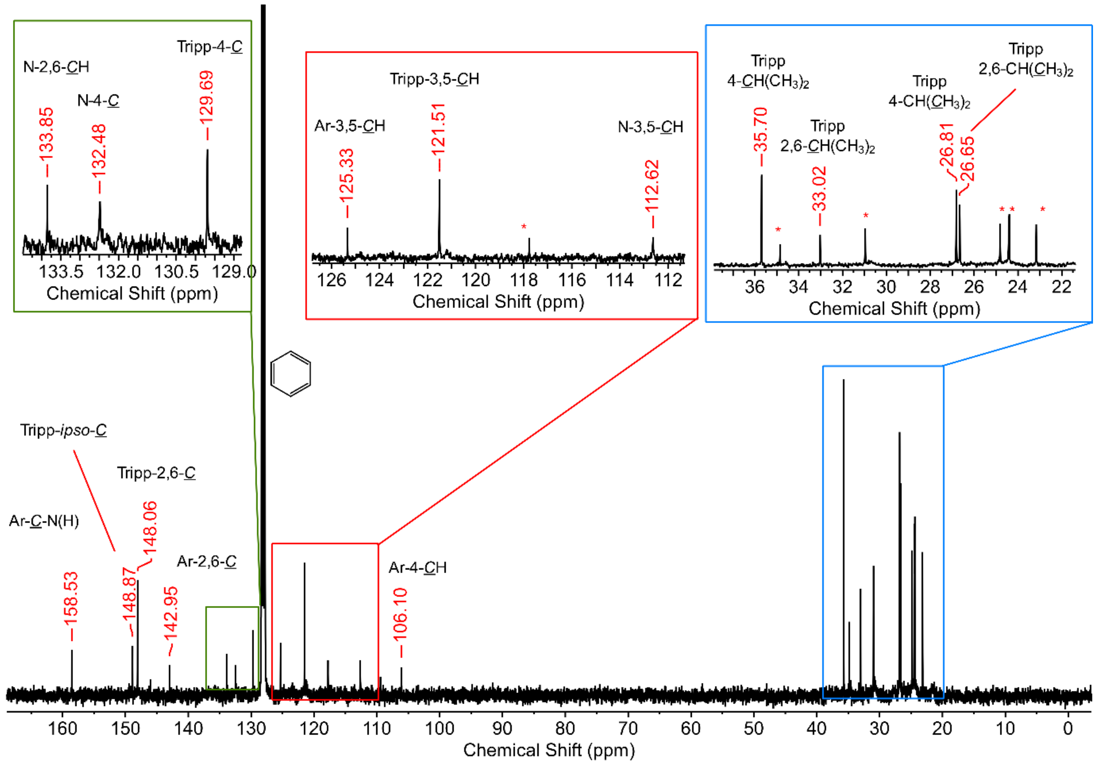

Figure S13. $^{13}C\{^{1}H\}$ NMR spectrum of **2La** in d_6-benzene. ‡ and † denote residual toluene and *n*-hexane respectively, * denotes protic ligand (H_2NAr^{iPr6}).

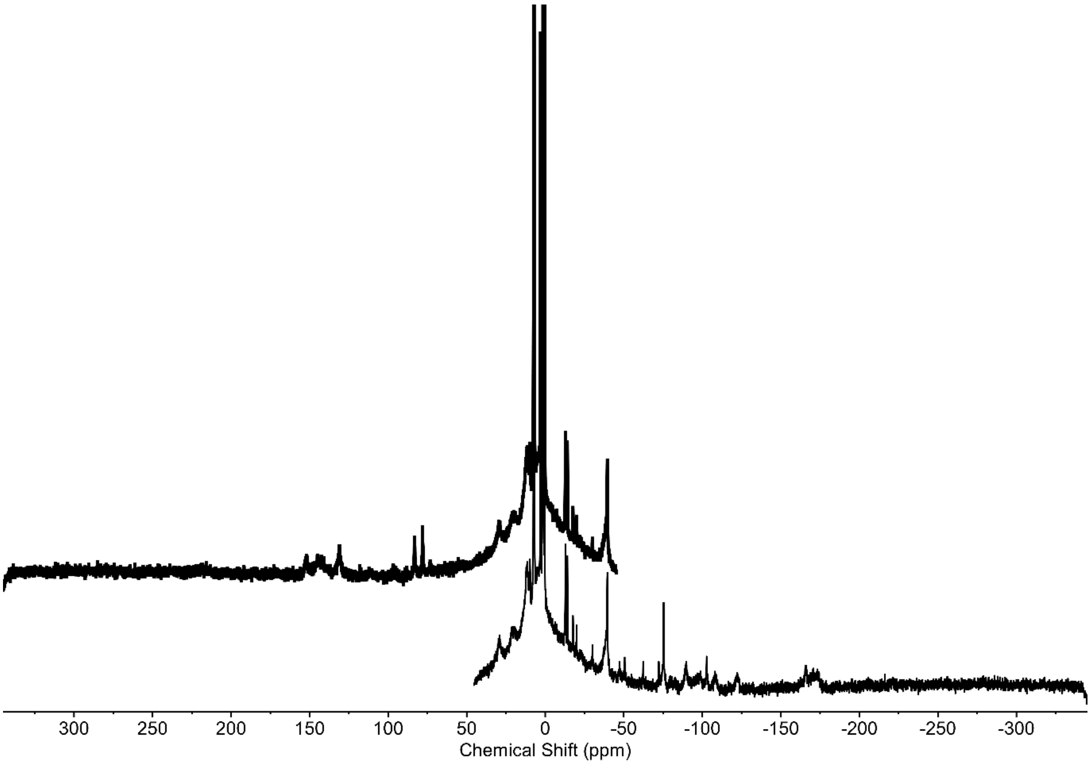

Figure S14. ^{1}H NMR spectrum of **2Tm** in d_6-benzene from +345 to −345 ppm showing the only observable peaks.

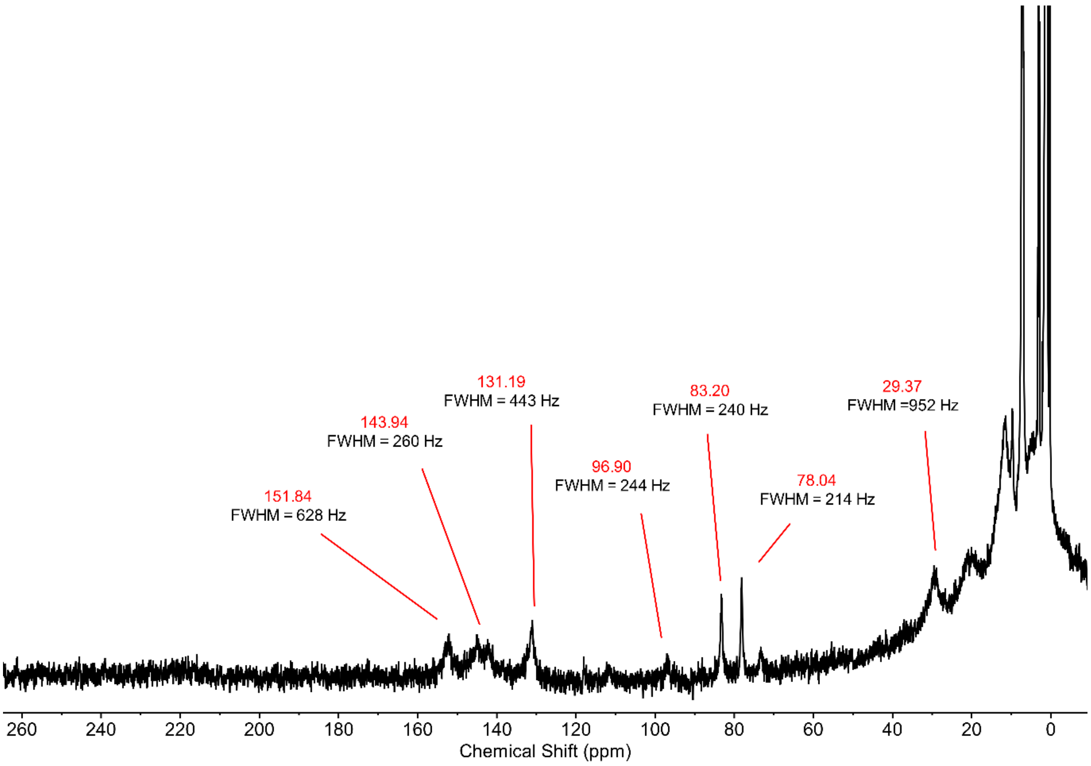

Figure S15. ^{1}H NMR spectrum of **2Tm**) in d_6-benzene from +260 to 0 ppm showing the only observable peaks. Exponential (15.0 Hz) line broadening was applied.

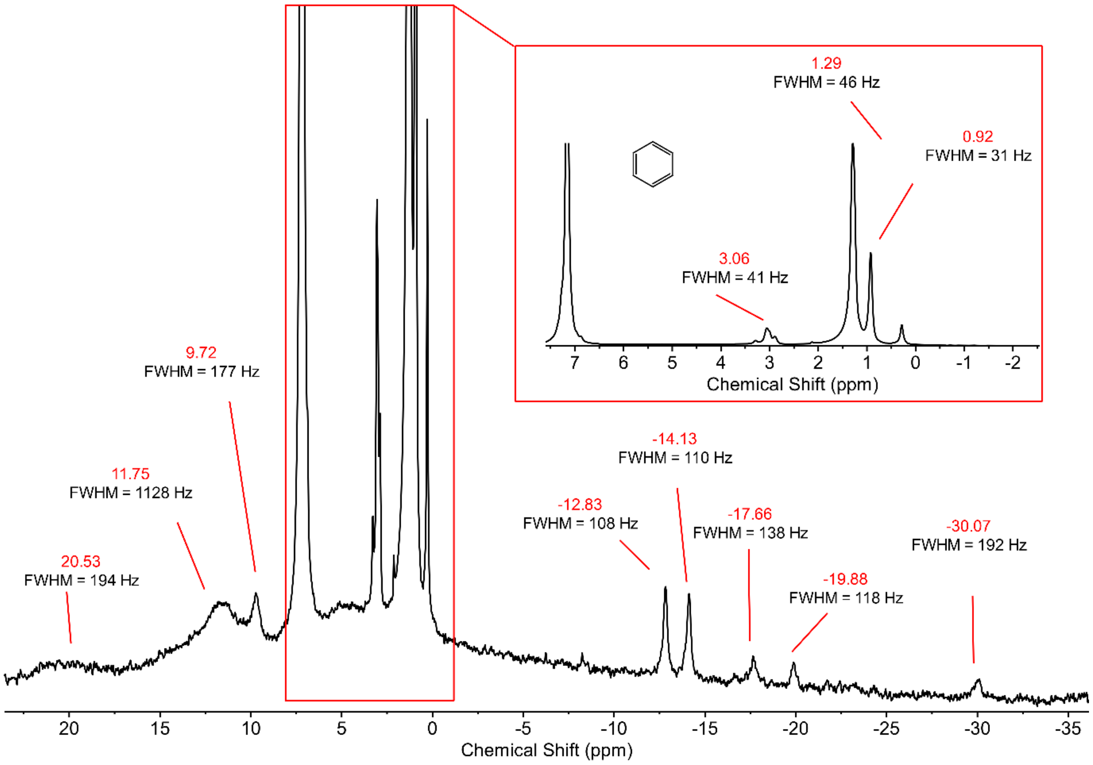

Figure S16. ^{1}H NMR spectrum of **2Tm** in d_6-benzene from +25 to −35 ppm showing the only observable peaks. Exponential (15.0 Hz) line broadening was applied.

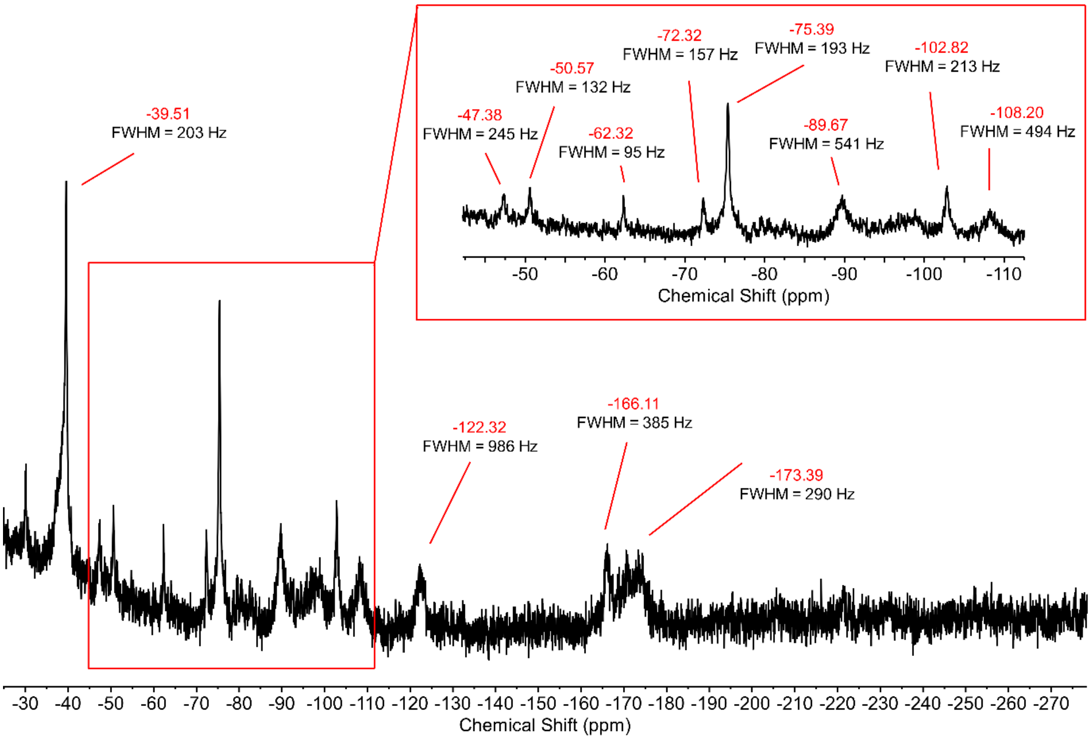

Figure S17. ^{1}H NMR spectrum of **2Tm** in d_6-benzene from −30 to −280 ppm showing the only observable peaks. Exponential (15.0 Hz) line broadening was applied.

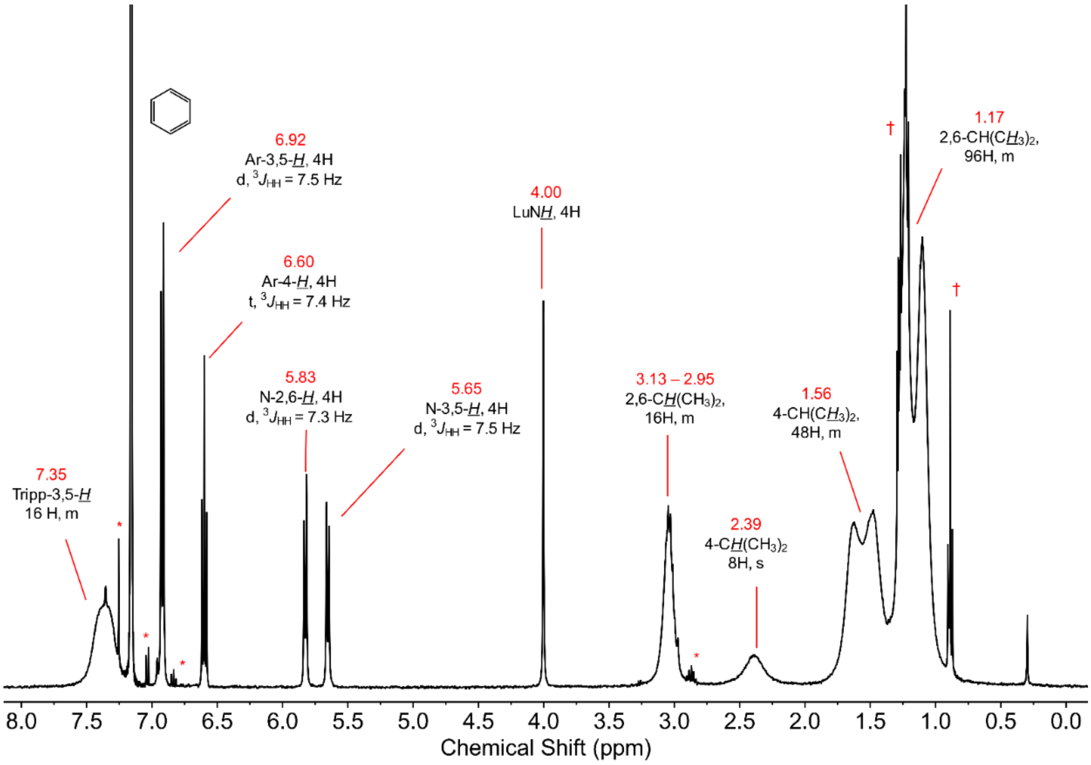

Figure S18. ^{1}H NMR spectrum of **2Lu** in d_6-benzene. ‡ and † denote residual toluene and *n*-hexane respectively, * denotes protic ligand (H_2NAr^{iPr6}).

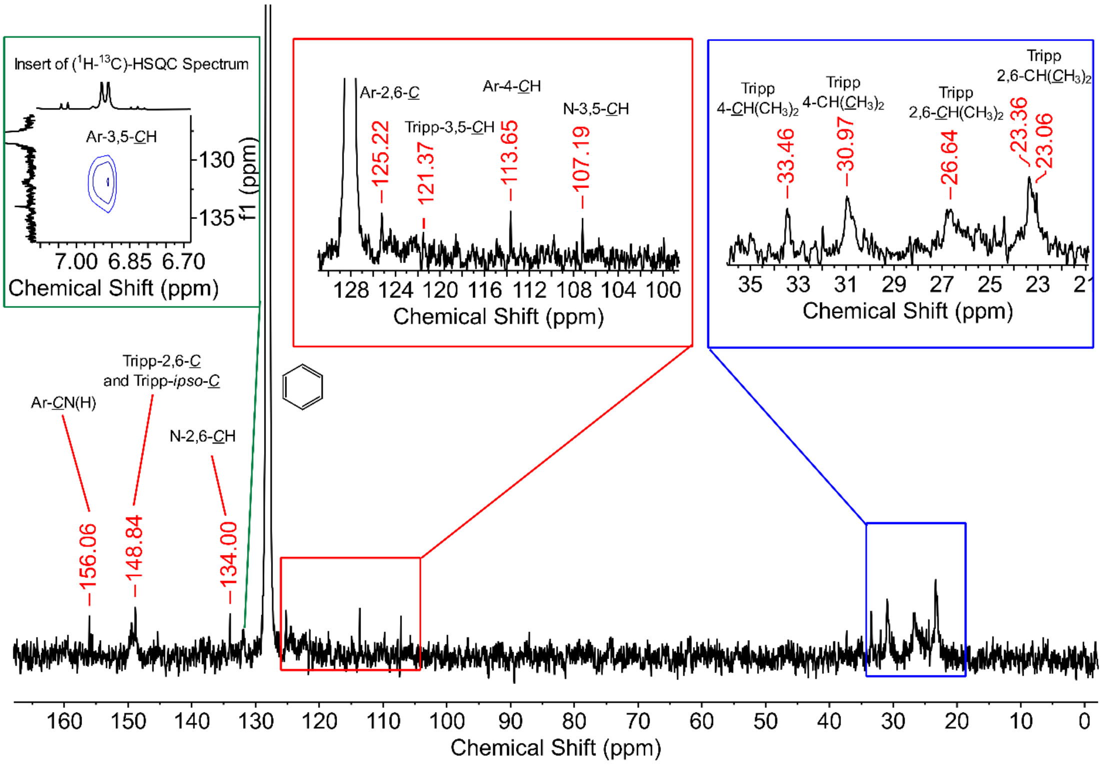

Figure S19. $^{13}C\{^1H\}$ NMR spectrum of **2Lu** in d_6-benzene. ‡ and † denote residual toluene and *n*-hexane respectively, * denotes protic ligand (H_2NAr^{iPr6}).

*NMR spectra of **3M** (M = Y, La, Tm, Lu)*

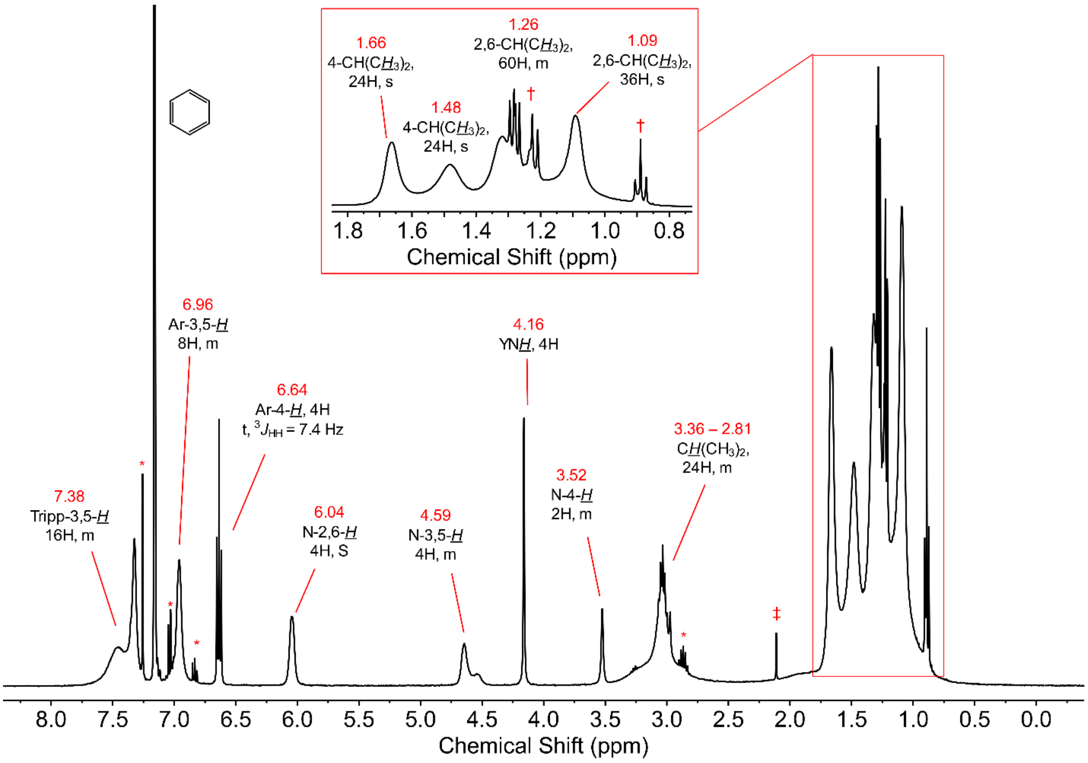

Figure S20. ^{1}H NMR spectrum of **3Y** in d_6-benzene. ‡ and † denote residual toluene and *n*-hexane respectively, * denotes protic ligand (H_2NAr^{iPr6}).

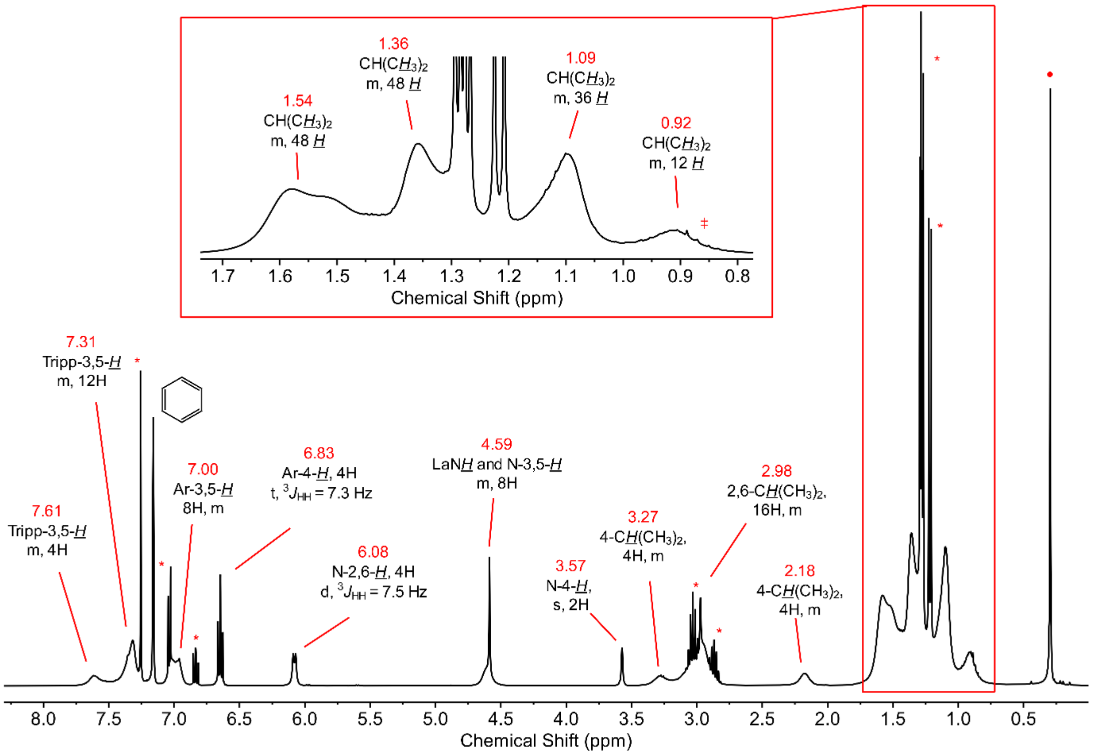

Figure S21. ^{1}H NMR spectrum of **3La** in d_6-benzene. ‡ and † denote residual toluene and *n*-hexane respectively, * denotes protic ligand (H_2NAr^{iPr6}).

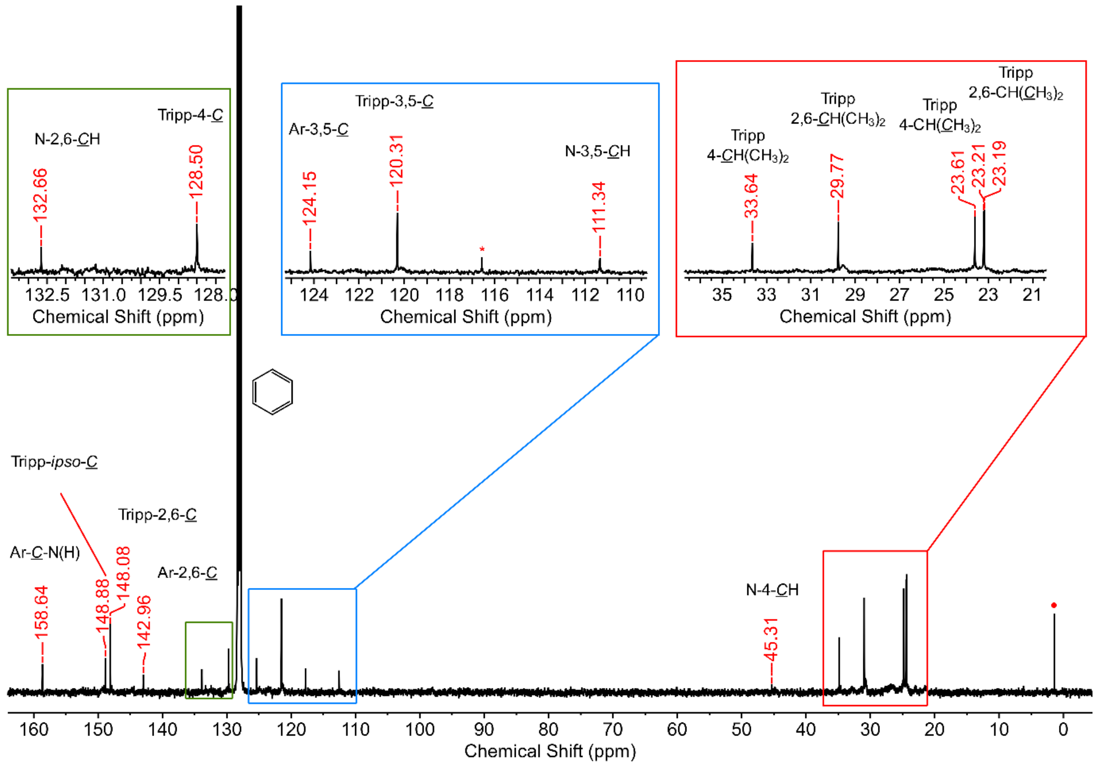

Figure S22. $^{13}C\{^{1}H\}$ NMR spectrum of **3La** in d_6-benzene. ‡ and † denote residual toluene and *n*-hexane respectively, * denotes protic ligand (H_2NAr^{iPr6}).

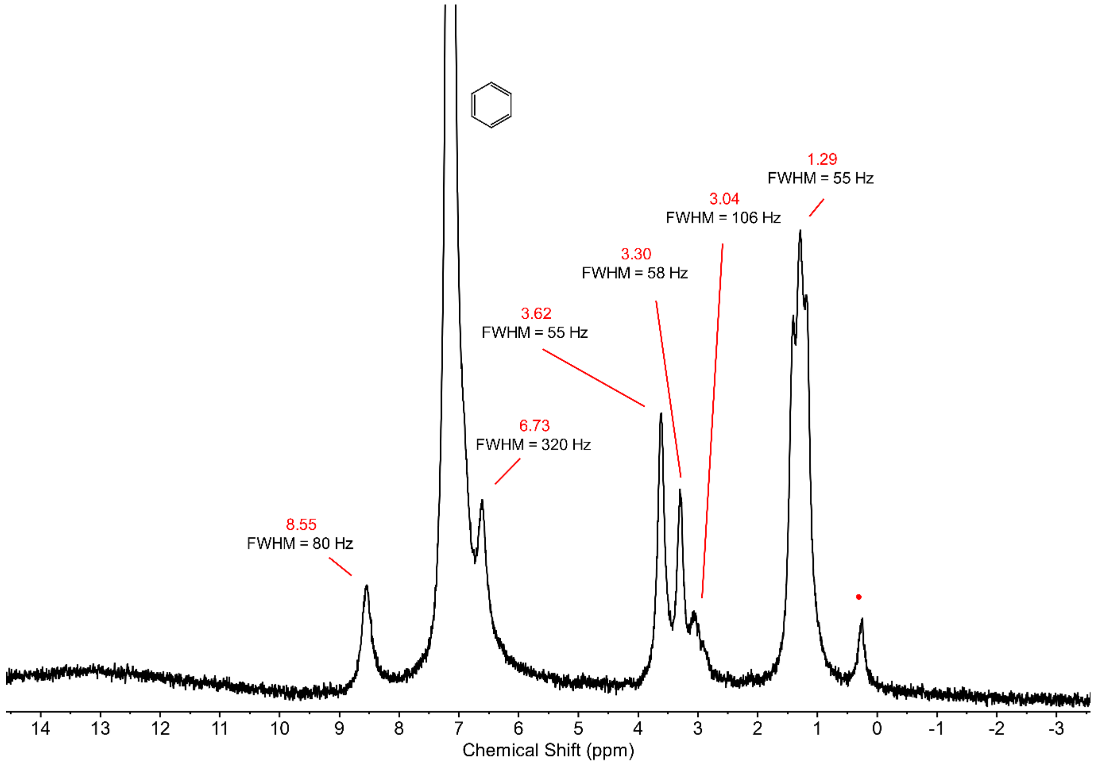

Figure S23. ^{1}H NMR spectrum of **3Tm** in d_6-benzene from +15 to -4 ppm showing the only observable peaks. Exponential (5.0 Hz) line broadening was applied.

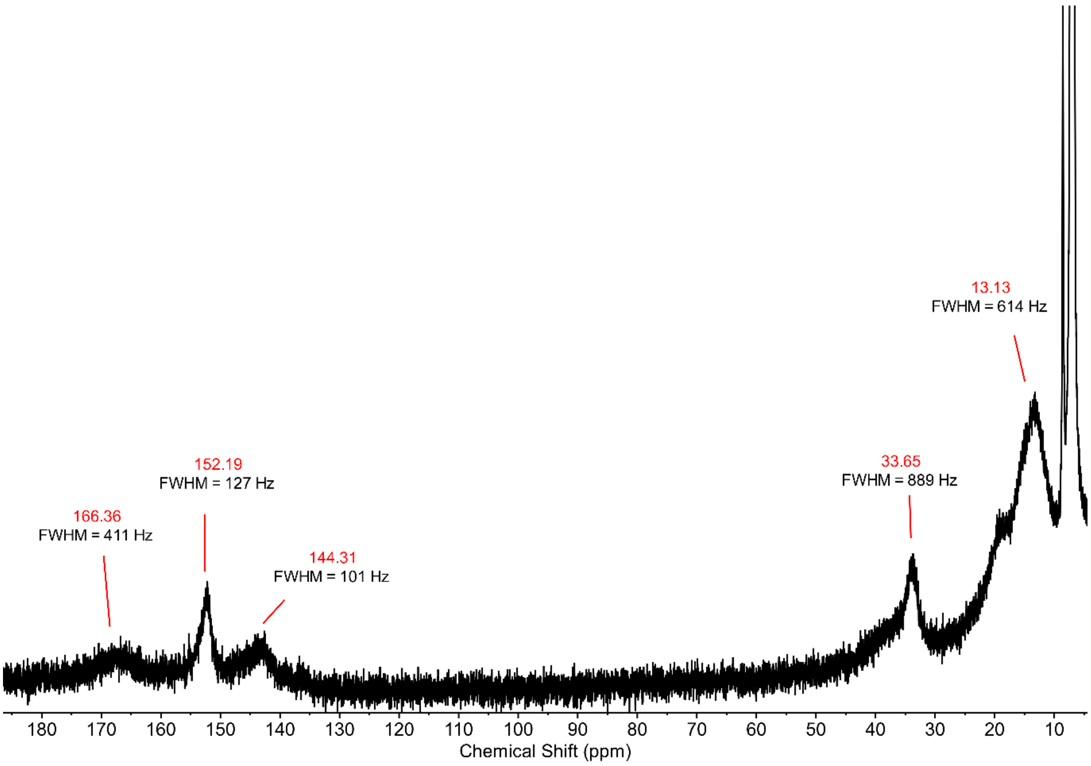

Figure S24. ^{1}H NMR spectrum of **3Tm** in d_6-benzene from +190 to 0 ppm showing the only observable peaks. Exponential (5.0 Hz) line broadening was applied.

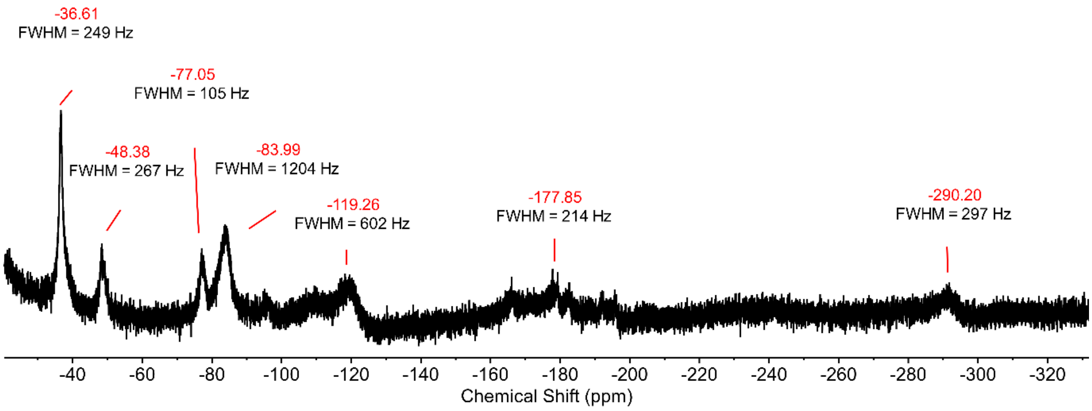

Figure S25. ^{1}H NMR spectrum of **3Tm** in d_6-benzene from –30 to –330 ppm showing the only observable peaks. Exponential (5.0 Hz) line broadening was applied.

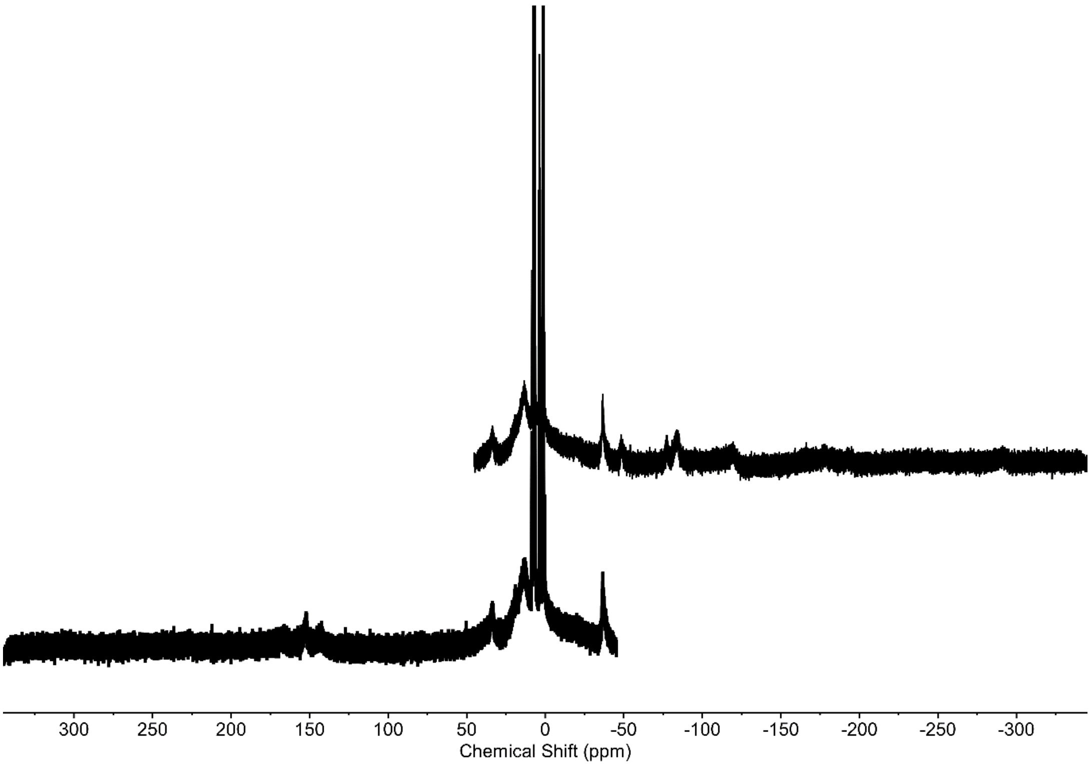

Figure S26. ^{1}H NMR spectrum of **3Tm** in d_6-benzene from +345 to −345 ppm showing the only observable peaks.

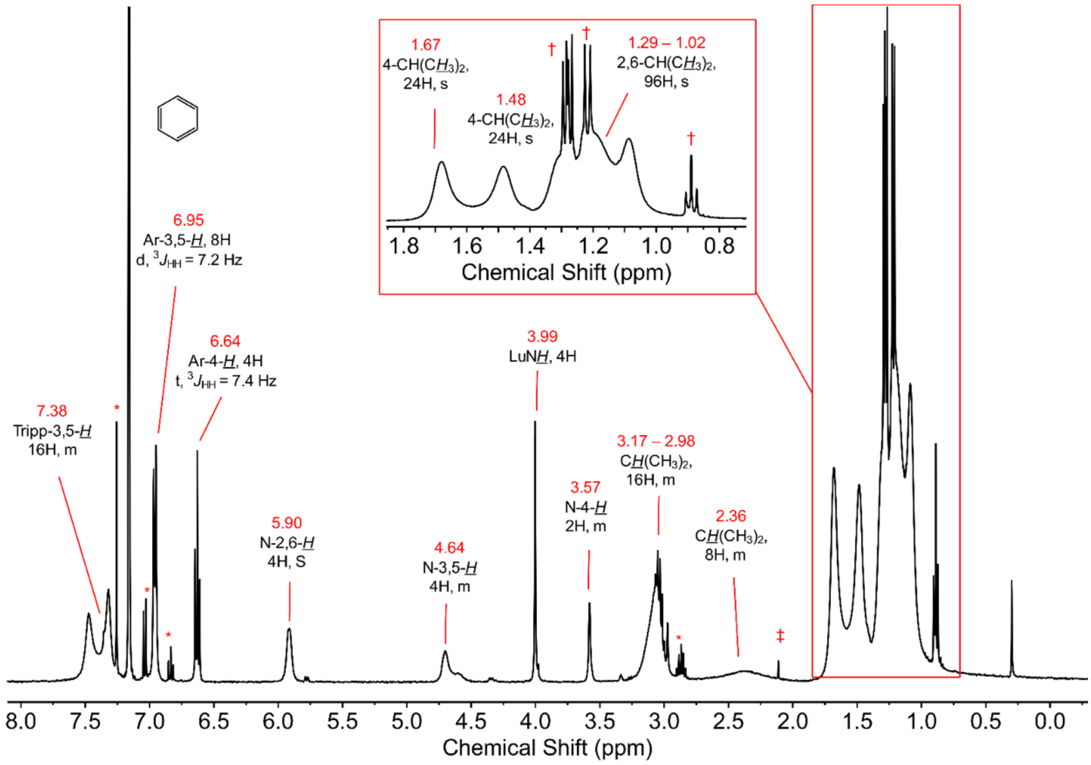

Figure S27. ^{1}H NMR spectrum of **3Lu** in d_6-benzene. ‡ and † denote residual toluene and *n*-hexane respectively, * denotes protic ligand (H_2NAr^{iPr6}).

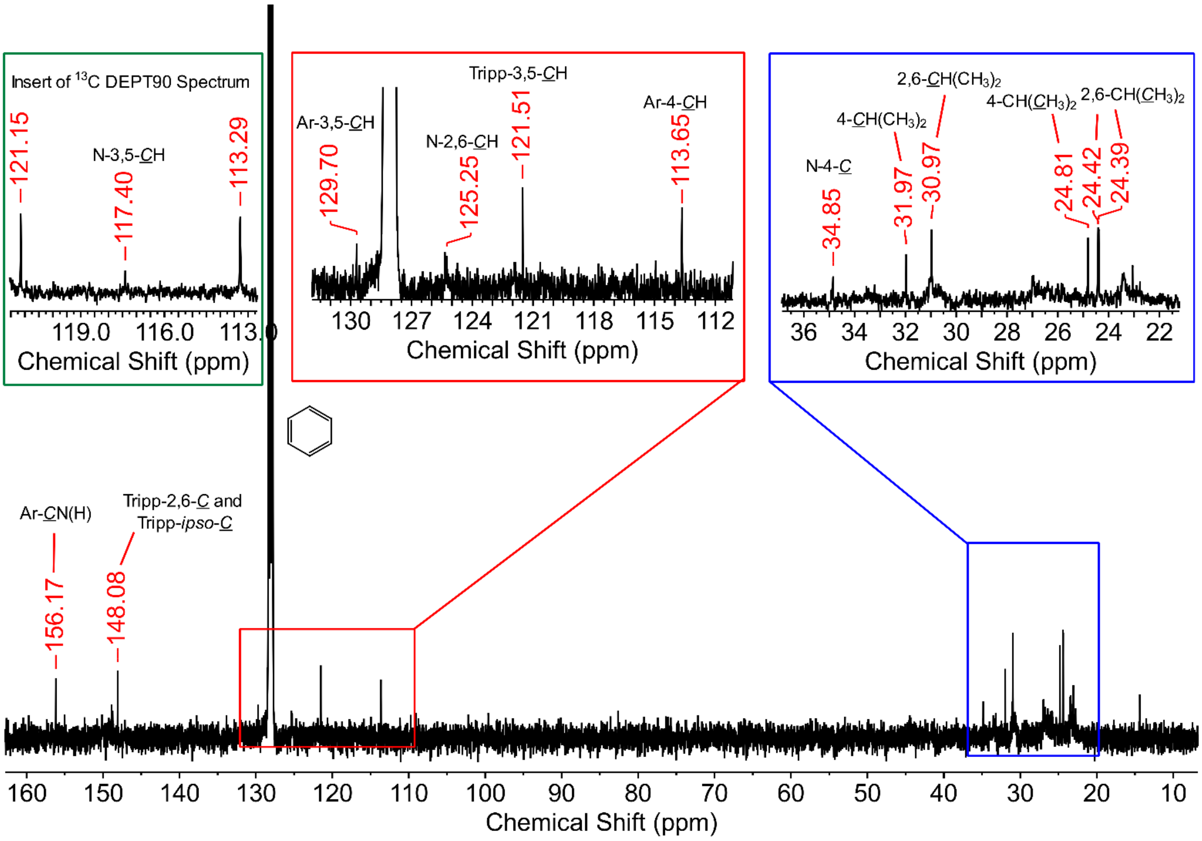

Figure S28. $^{13}C\{^1H\}$ NMR spectrum of **3Lu** in d_6-benzene. ‡ and † denote residual toluene and *n*-hexane respectively, * denotes protic ligand (H_2NAr^{iPr6}).

Magnetic moments determined by NMR spectroscopy (Evans method)

Table S8. Data for the determination of the magnetic moments of complexes **2Tm** and **3Tm**.

Sample / peak	μ_{eff} / B.M mol^{-1}	mass of sample / g [A]	mass of solvent / g	M_r / g mol^{-1}	Δ peak / Hz [B]
**2Tm**	6.071	0.0151	0.5840	1240.61	498.64
**3Tm**	7.498	0.0148	0.7820	1241.61	563.83

[A] The small masses engender large errors in this methodology, the results should be cautiously interpreted along with other data. [B] Spectrometer frequency 400.170 MHz. Diamagnetic correction of M_r / −2,000,000 applied. ρd_6-benzene = 0.950 g mL^{-1}.

S5. ATR-IR spectroscopy

*ATR-IR spectra of **2M** (M = Y, La, Tm, Lu)*

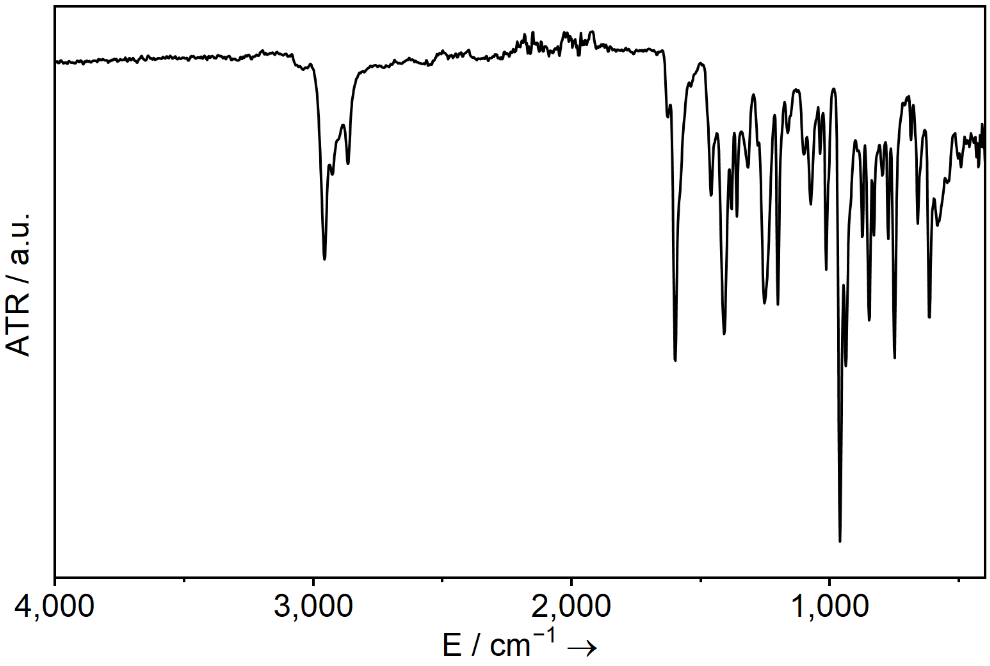

Figure S29. ATR-IR spectrum of microcrystalline **2Y**.

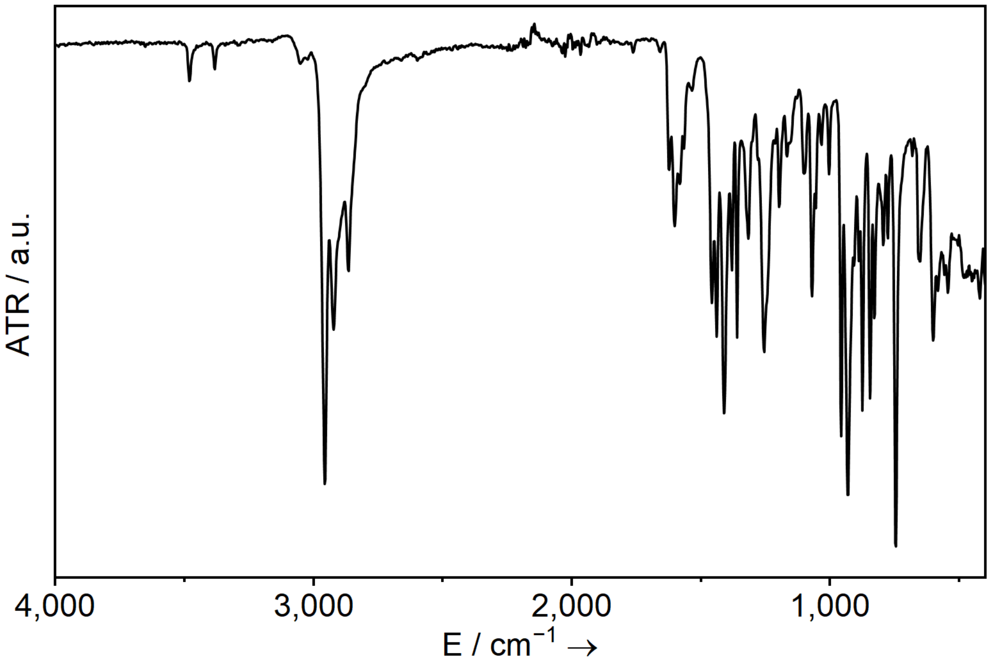

Figure S30. ATR-IR spectrum of microcrystalline **2La**.

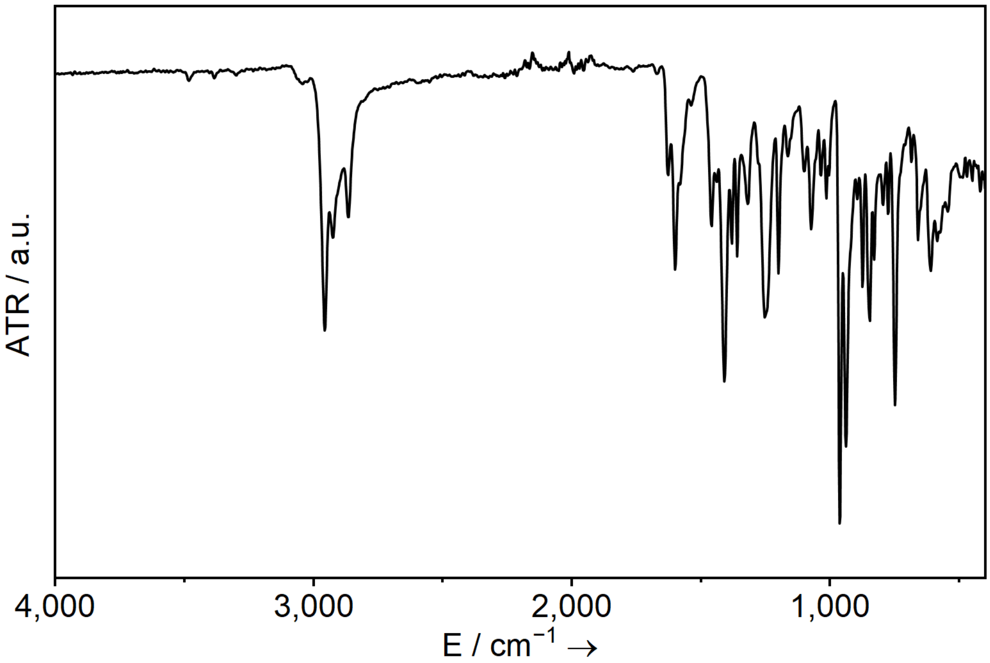

Figure S31. ATR-IR spectrum of microcrystalline **2Tm**.

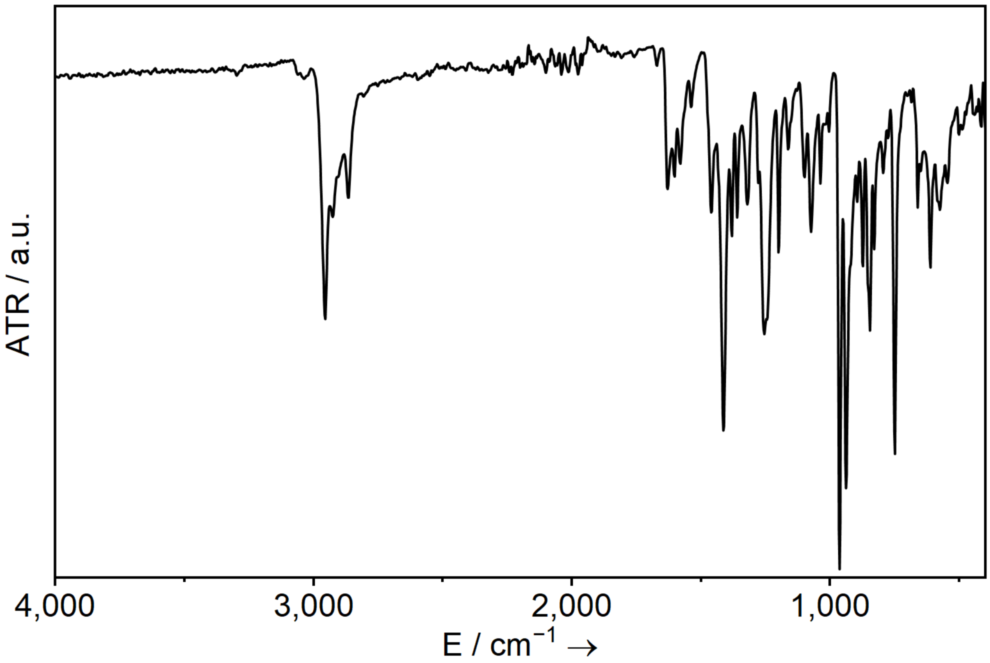

Figure S32. ATR-IR spectrum of microcrystalline **2Lu**.

*ATR-IR spectra of **3M** (M = Y, La, Tm, Lu)*

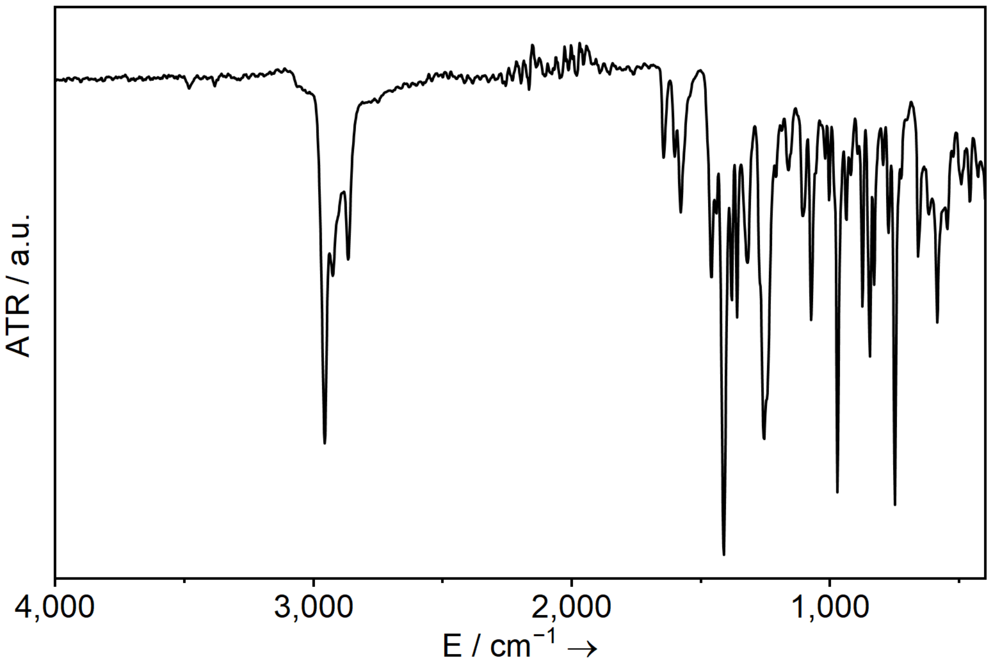

Figure S33. ATR-IR spectrum of microcrystalline **3Y**.

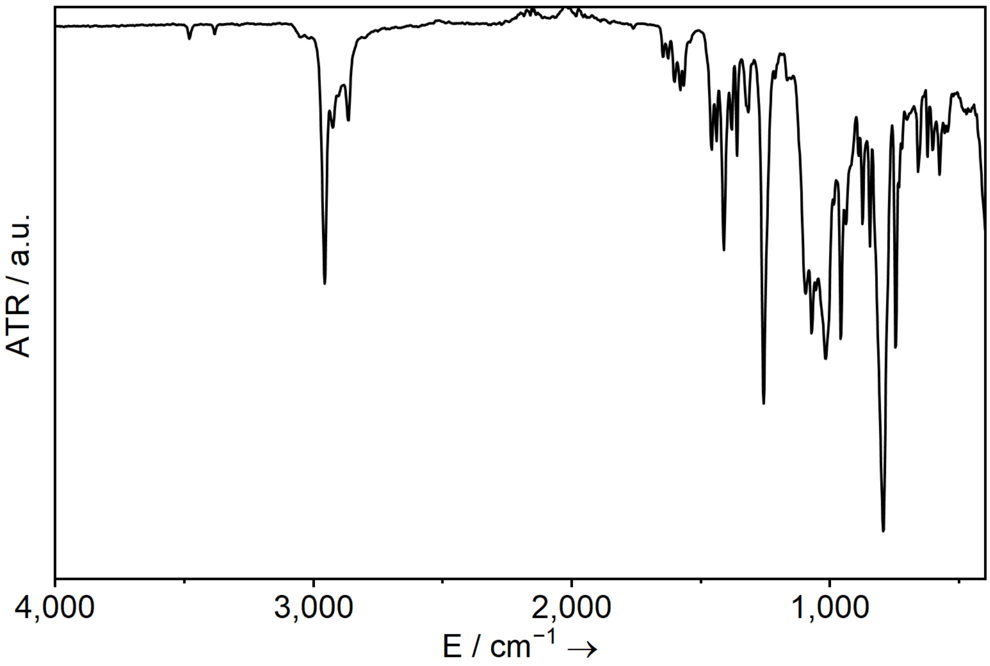

Figure S34. ATR-IR spectrum of microcrystalline **3La**.

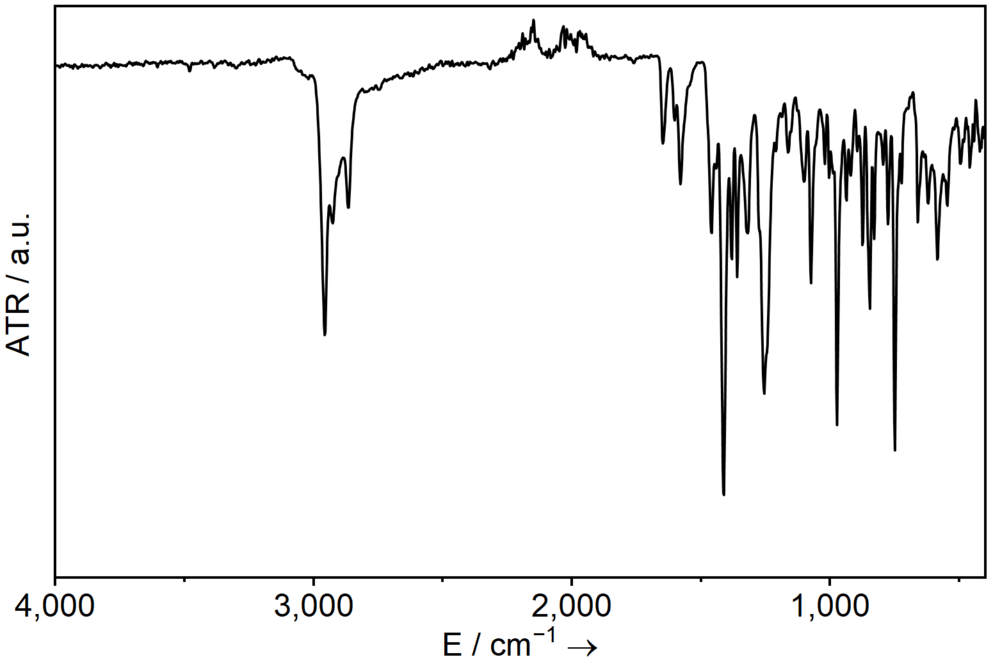

Figure S35. ATR-IR spectrum of microcrystalline **3Tm**.

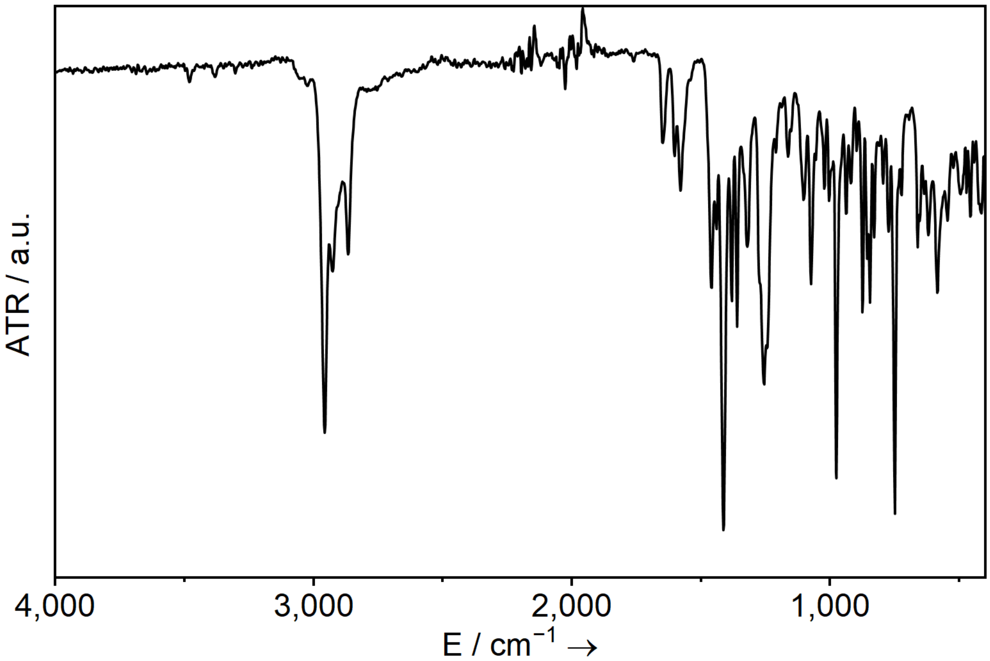

Figure S36. ATR-IR spectrum of microcrystalline **3Lu**.

S6. UV-Vis-NIR spectroscopy

UV-Vis-NIR spectra of ***2M*** *(M = Y, La, Tm, Lu)*

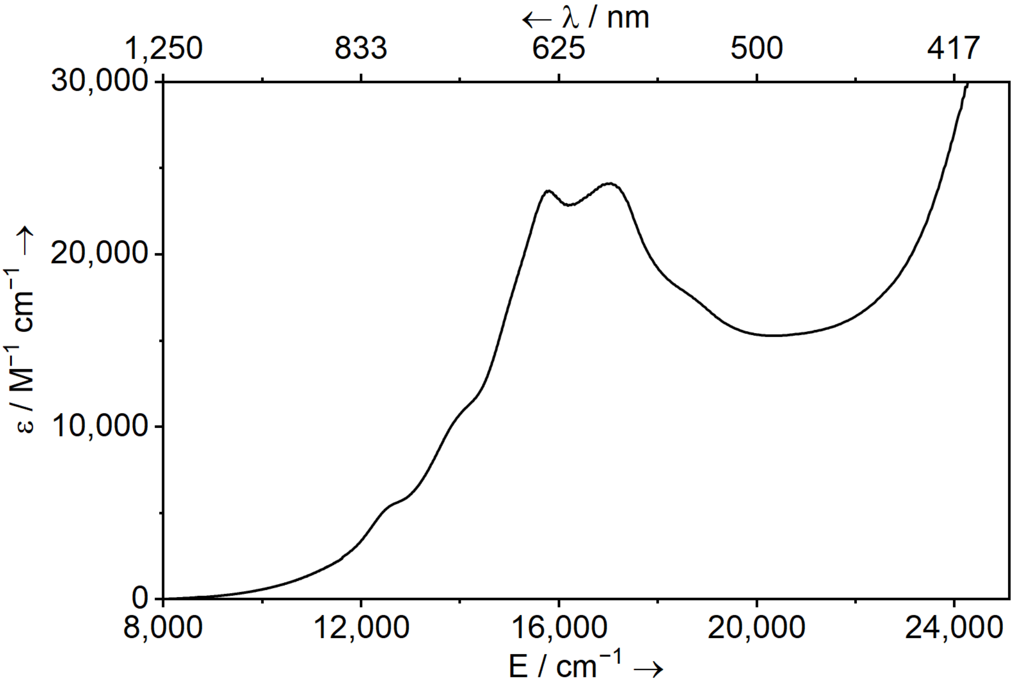

Figure S37. Solution UV-Vis-NIR spectrum of **2Y** (0.1 mM) in toluene shown between 8,000–25,000 cm^{-1} (1,250–400 nm) at ambient temperature.

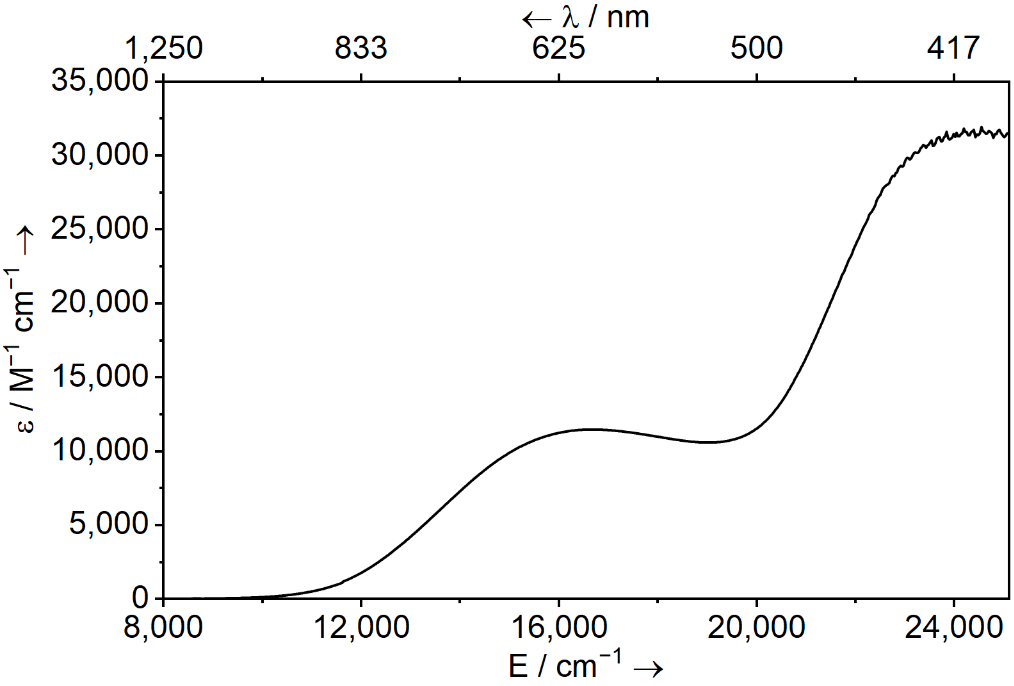

Figure S38. Solution UV-Vis-NIR spectrum of **2La** (0.1 mM) in toluene shown between 8,000–25,000 cm^{-1} (1,250–400 nm) at ambient temperature.

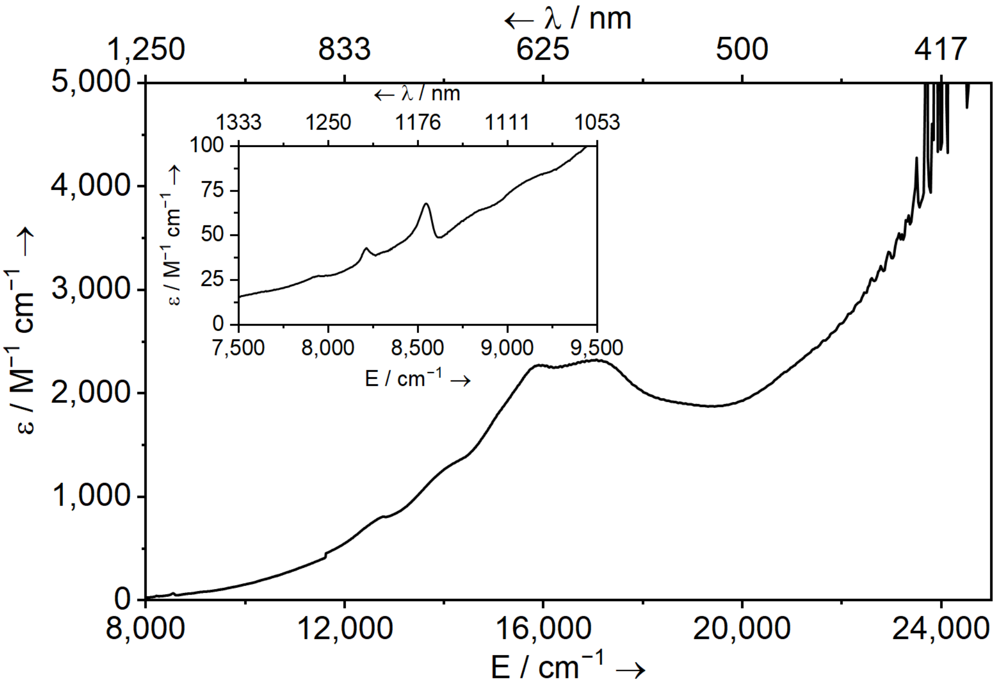

Figure S39. Solution UV-Vis-NIR spectrum of **2Tm** (0.1 mM) in toluene shown between 8,000–25,000 cm^{-1} (1,250–400 nm) at ambient temperature.

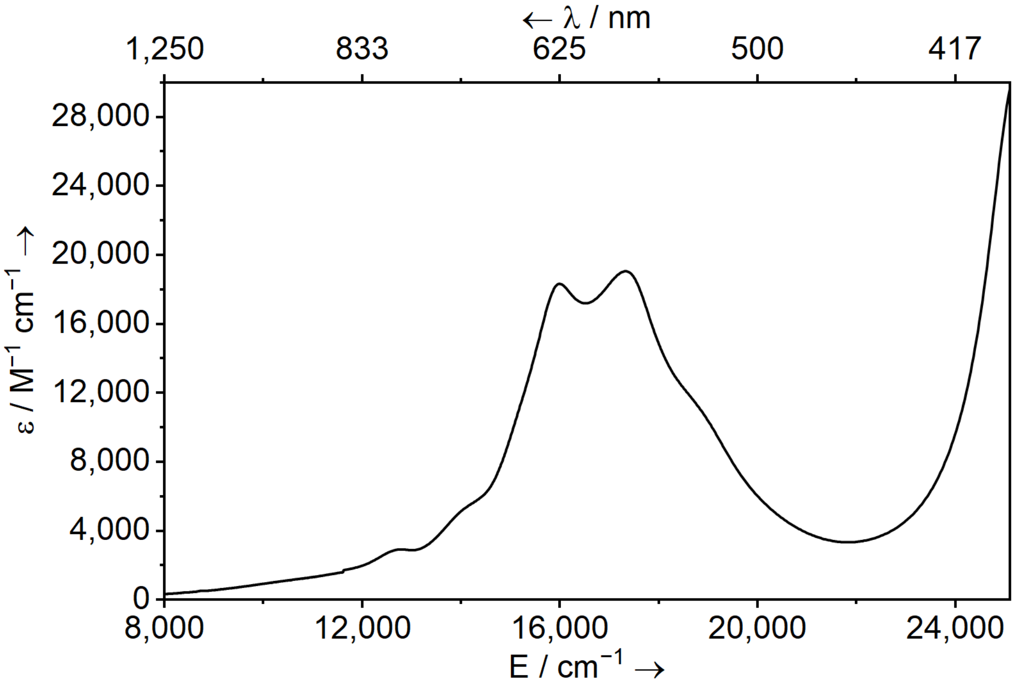

Figure S40. Solution UV-Vis-NIR spectrum of **2Lu** (0.1 mM) in toluene shown between 8,000–25,000 cm^{-1} (1,250–400 nm) at ambient temperature.

*Solid State UV-vis spectra of **2M** (M = Y, La, Tm, Lu)*

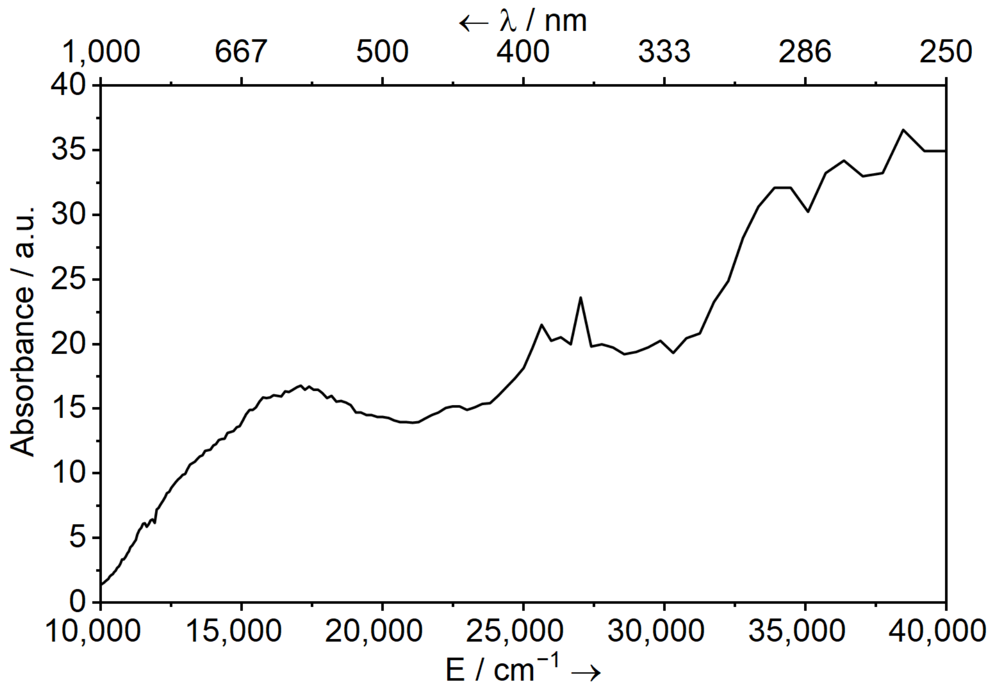

Figure S41. Solid state UV-Vis spectrum of **2Y** shown between 10,000–40,000 cm^{-1} (1,000–250 nm) at ambient temperature.

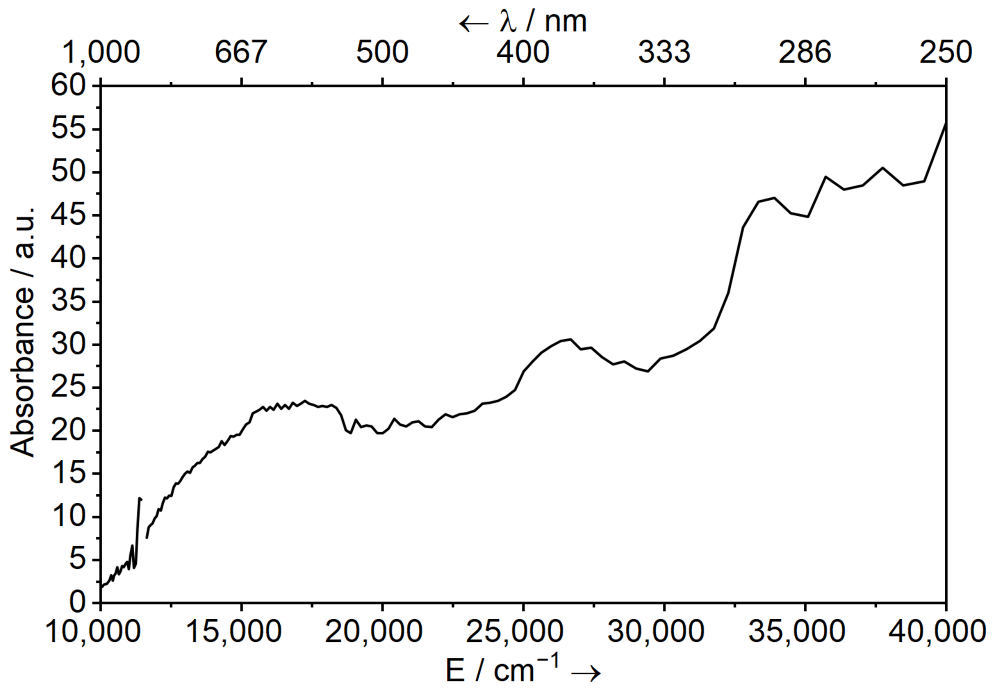

Figure S42. Solid state UV-Vis spectrum of **2La** shown between 10,000–40,000 cm^{-1} (1,000–250 nm) at ambient temperature.

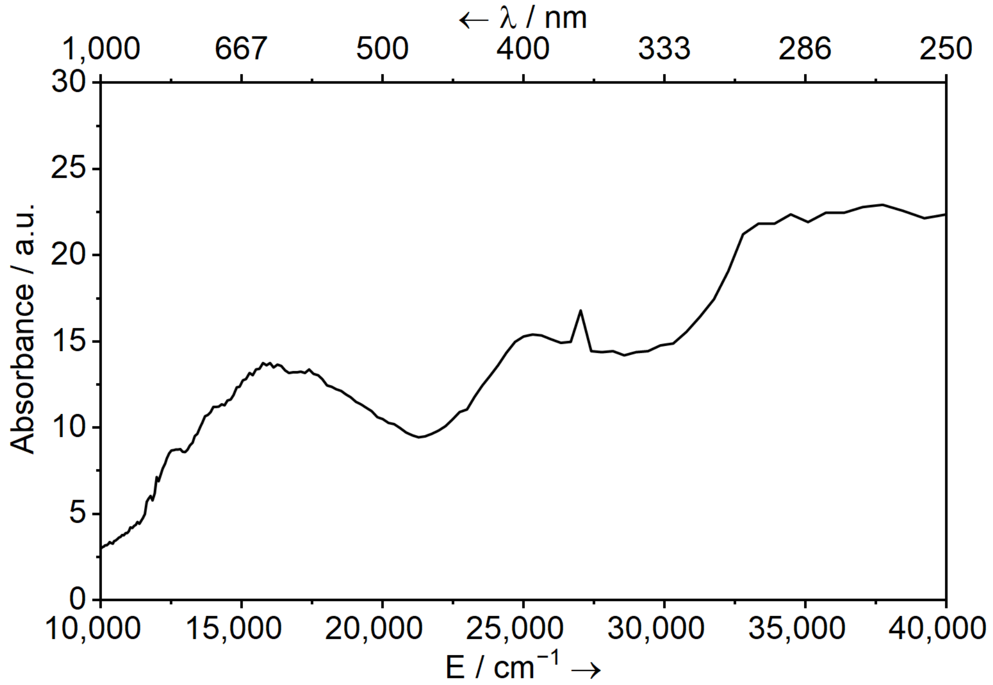

Figure S43. Solid state UV-Vis spectrum of **2Tm** shown between 10,000–40,000 cm^{-1} (1,000–250 nm) at ambient temperature.

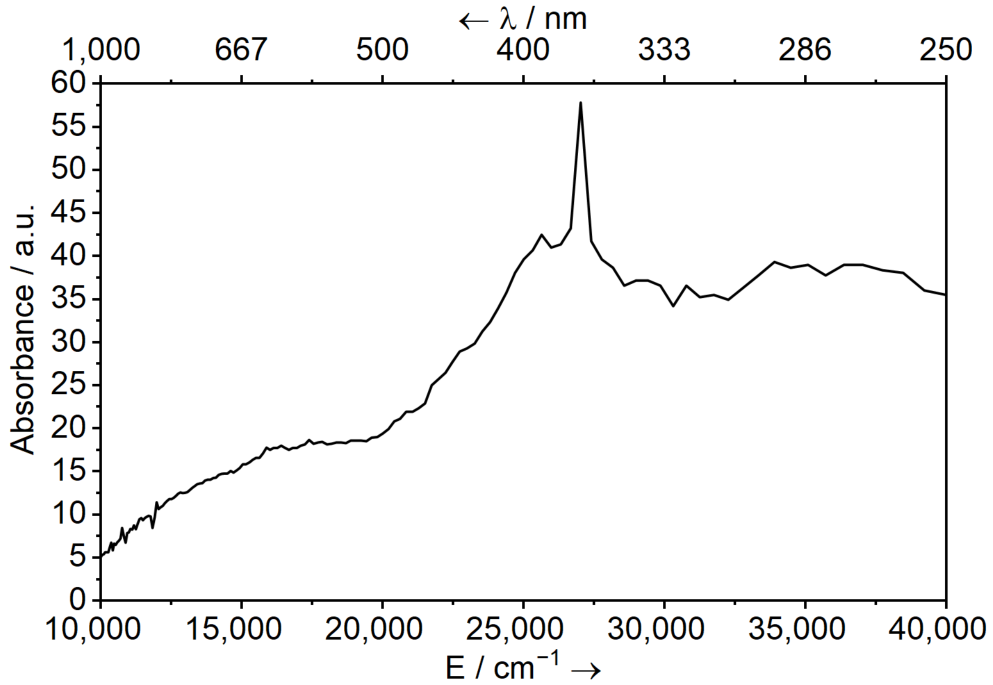

Figure S44. Solid state UV-Vis spectrum of **2Lu** shown between 10,000–40,000 cm^{-1} (1,000–250 nm) at ambient temperature.

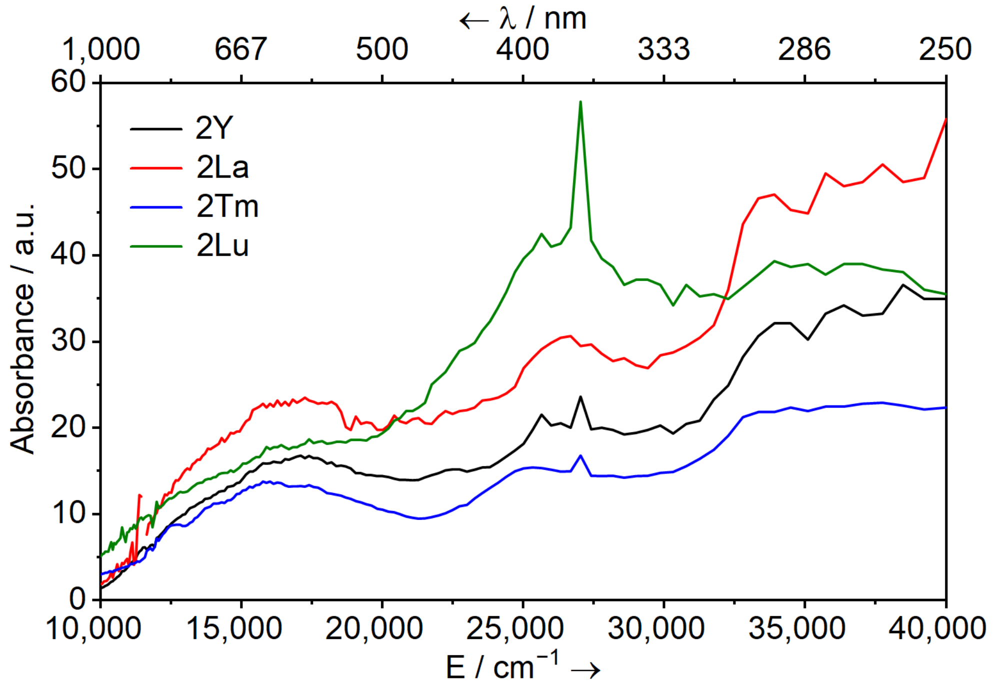

Figure S45. Stacked solid state UV-Vis spectra of **2M** (M = Y, La, Tm, Lu) complexes.

*UV-Vis-NIR spectra of **3M** (M = Y, La, Tm, Lu)*

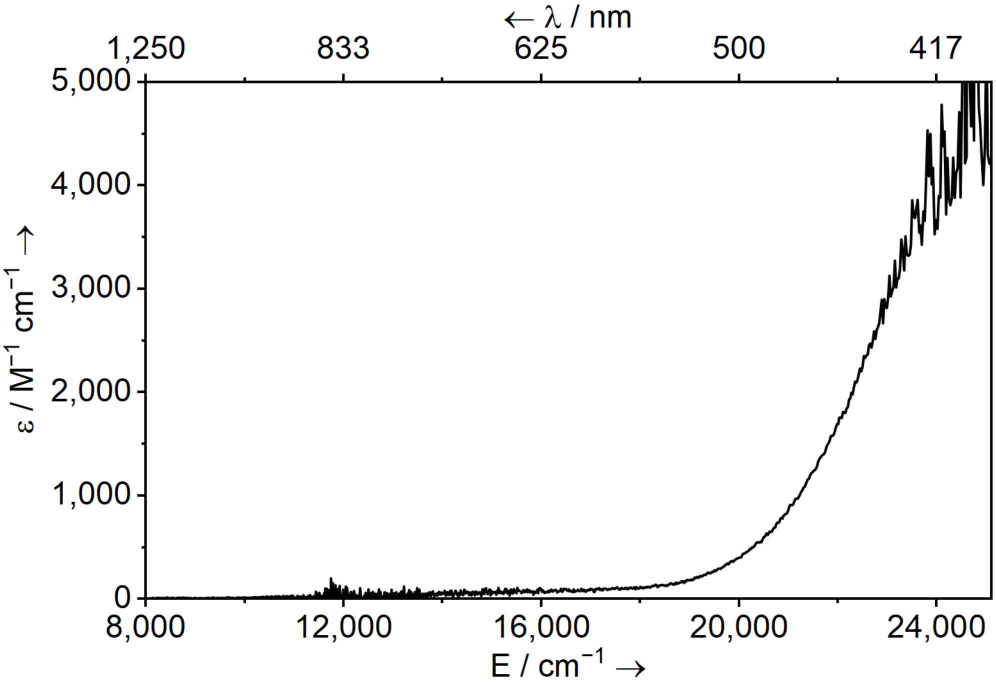

Figure S46. Solution UV-Vis-NIR spectrum of **3Y** (0.5 mM) in toluene shown between 8,000–25,000 cm^{-1} (1,250–400 nm) at ambient temperature.

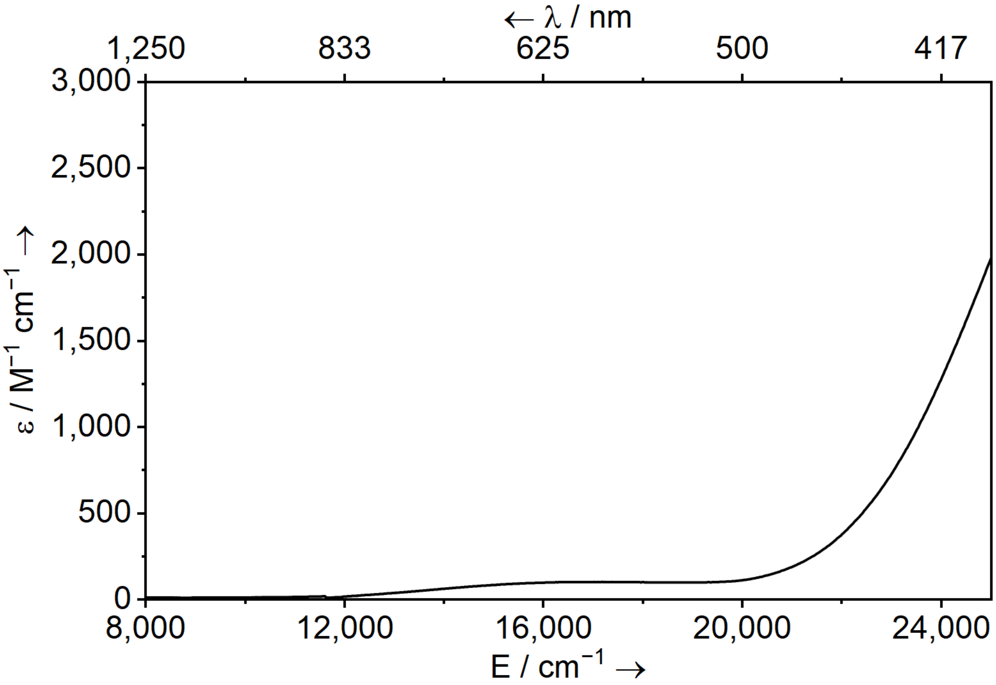

Figure S47. Solution UV-Vis-NIR spectrum of **3La** (0.5 mM) in toluene shown between 8,000–25,000 cm^{-1} (1,250–400 nm) at ambient temperature.

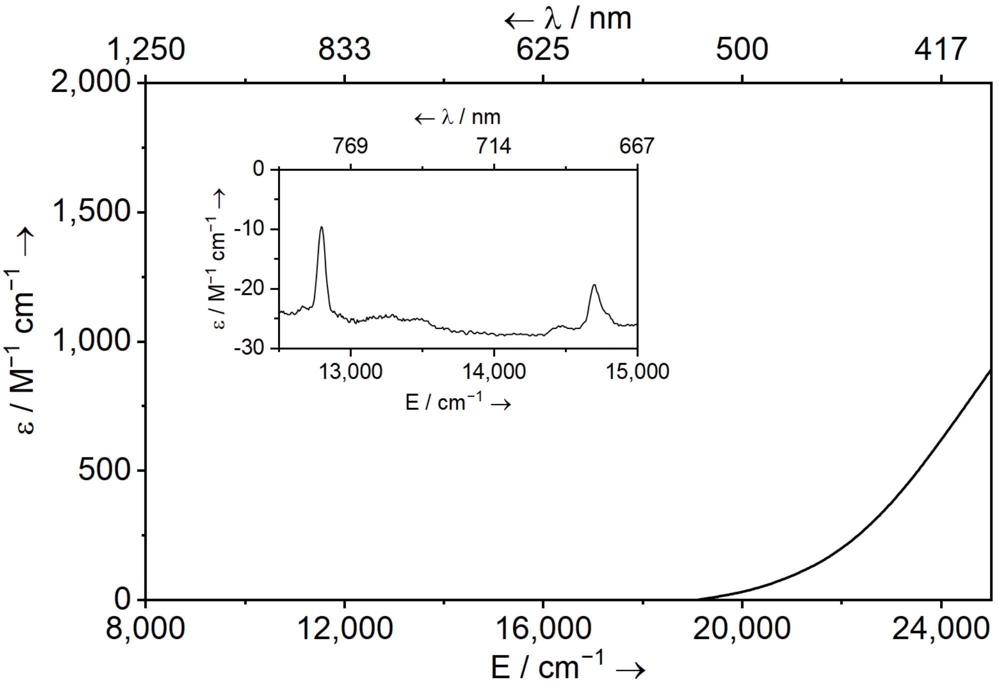

Figure S48. Solution UV-Vis-NIR spectrum of **3Tm** (0.5 mM) in toluene shown between 8,000–25,000 cm^{-1} (1,250–400 nm) at ambient temperature.

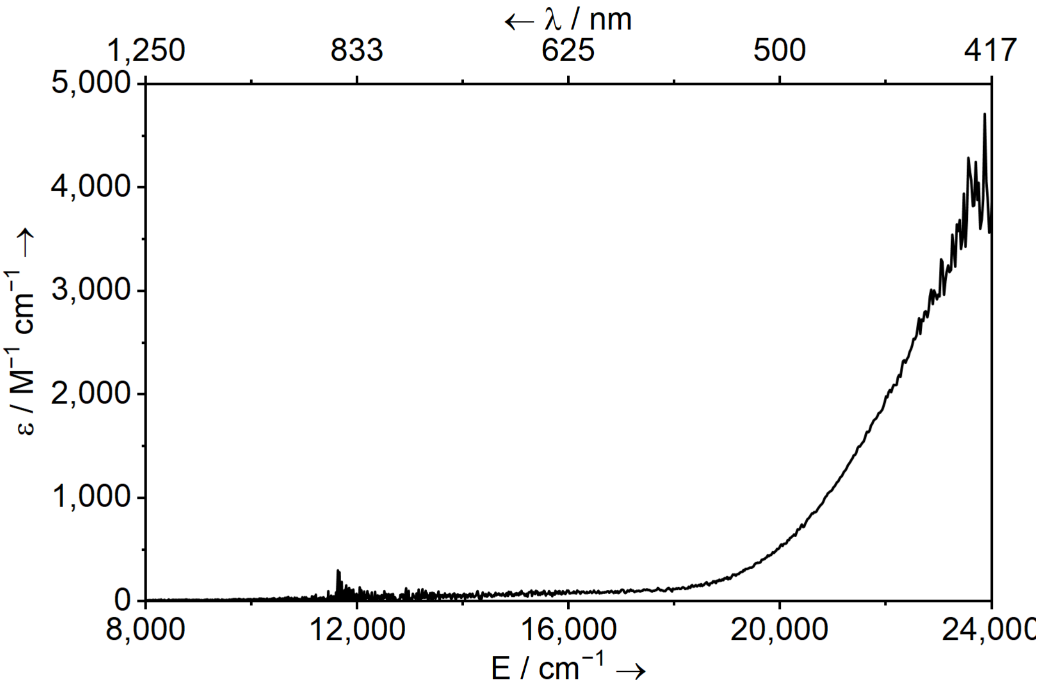

Figure S49. Solution UV-Vis-NIR spectrum of **3Lu** (0.5 mM) in toluene shown between 8,000–25,000 cm^{-1} (1,250–400 nm) at ambient temperature.

S7. Electrochemical studies on 1M complexes and 2Y

General Methods

Electrochemical data were measured using a Ivium CompactStat.h20250 24-bit measurement and 20-bit generation Potentiostat/Galvanostat. Data was collected using the IviumSoft 4.1141 software operating in Basic Mode. Measurements were performed in a glovebox under an atmosphere of argon with a glass carbon working electrode (3.0mm diameter, BASi MF-2012), a Ag/AgCl wire reference electrode (prepared by treating Ag wire with an aqueous $Fe^{III}Cl_3$ solution), and a platinum wire auxiliary counter electrode (~7.5cm length, BASi MW-1032) at ambient temperature (~298 K). [*n*Bu_4N][BPh_4] was prepared from the reaction of Na[BPh_4] and [*n*Bu_4N]Cl in H_2O, the product was extracted with DCM and a crude white powder was afforded after workup, which was recrystallised three times from acetone and dried under vacuum (10^{-3} mbar) at 60°C for 24 hours prior to use. Na[BPh_4] (Fluorochem) and [*n*Bu_4N]Cl (Merck) were used without further purification. Electrolyte solutions comprising of 50 mM [*n*Bu_4N][BPh_4] and 10 mM **1M** (M = Y, La, Sm, Eu, Tm, Yb, Lu) or 2.5 mM **2Y** were prepared in THF (degassed by sparging and dried by passage through neutral alumina columns (INERT Corp.), stored and degassed under vacuum over pre-activated (heating for 8 hours at 300°C, 10^{-3} mbar) 3 Å molecular sieves for 7 days before use). Decamethylferrocene ([Fe(Cp*)$_2$], Fc*, Cp* = {C_5Me_5}), was purchased from Merck and sublimed prior to use, and was used as the internal standard. Voltammograms were thus referenced by adding *ca.* 3 mg of Fc* to electrolyte solutions at the end of the experiment collection. All potentials are reported *vs* $[Fe(Cp)_2]^{+/0}$ ($[Fc]^{+/0}$), using the conversion of decamethylferrocene $E°'$ = –0.5 V *vs* $[Fc]^{+/0}$ using 50 mM [*n*Bu_4N][BPh_4] in THF as reported by La Pierre.[8]

Treatment of Data

Fc* is added to the analyte solution at the end of the experiment as an internal standard that is more chemically inert than Fc. The potential step used was 0.02 V, and therefore, the minimum error is ±0.02 V. The data for the second redox event in **Table S9** were internally corrected to the [**1M**]$^{+/0}$, M(III/II), couple for scans outside the range of the $[Fc]^{+/0}$ couple.

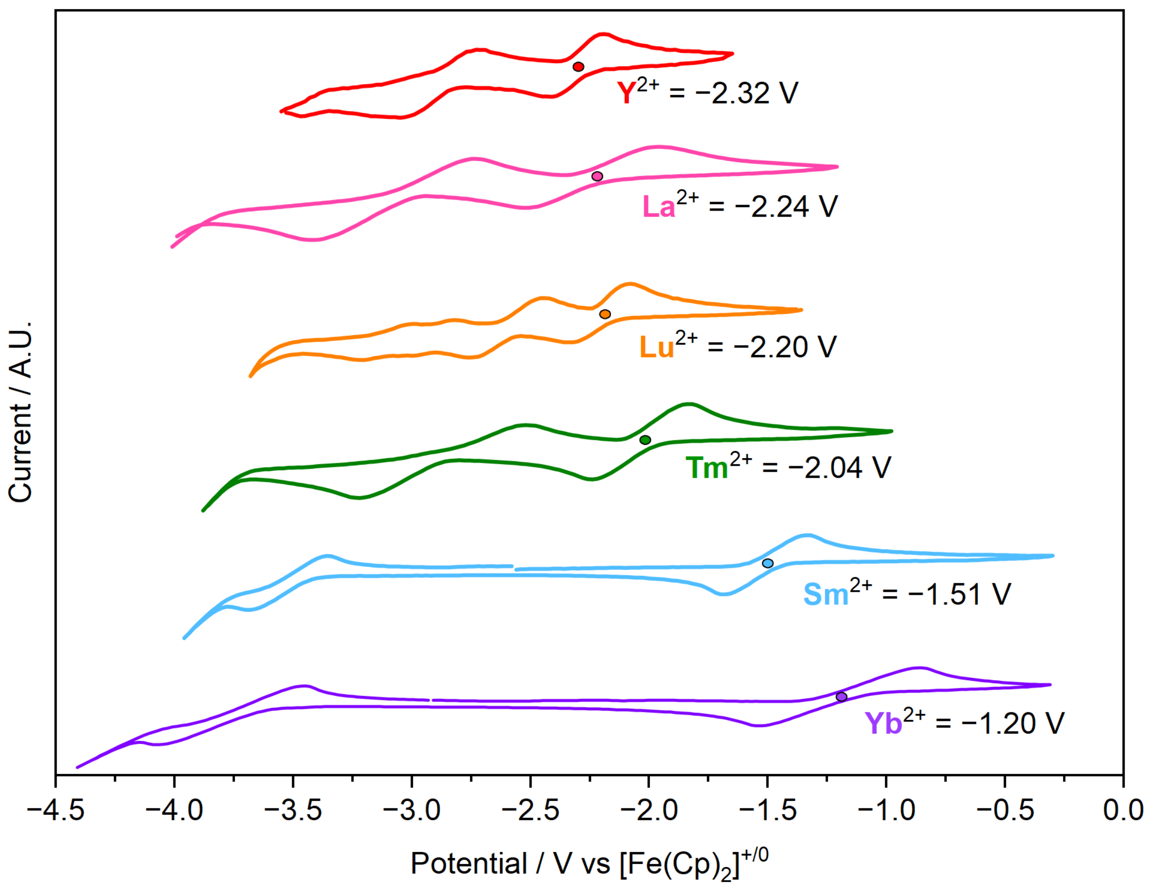

Figure S50. Cyclic voltammogram of **1M** (M = Y, La, Lu, Tm, Sm, Yb) in THF (10 mM) supported by [nBu$_4$N][BPh$_4$] (50 mM) vs [Fc]$^{+/0}$ couple at 200 mV s^{-1}.

Table S9. Summary of redox potentials for series **1M** (M = Y, La, Lu, Tm, Sm, Yb) vs Fc. Measured as 10 mM solutions in a 50 mM [nBu$_4$N][BPh$_4$] electrolyte THF solution. Redox potentials provided in Event 1 order with most to least negative. Note for **1Eu** no redox events were observed.

Complex	Event 1 – [M^{III}NHAr^{iPr6})$_2$]$^{+/0}$ /V	Error /V	Event 2 /V	Error /V
**1Y**	–2.32	± 0.02	–2.89	± 0.02
**1La**	–2.24	± 0.02	–3.07	± 0.05
**1Lu**	–2.20	± 0.02	–2.60	± 0.02
**1Tm**	–2.04	± 0.02	–2.87	± 0.08
**1Sm**	–1.51	± 0.02	–3.52	± 0.06
**1Yb**	–1.20	± 0.02	–3.76	± 0.08

***1Y** – [Y^{II}($NHAr^{iPr6}$)$_2$]*

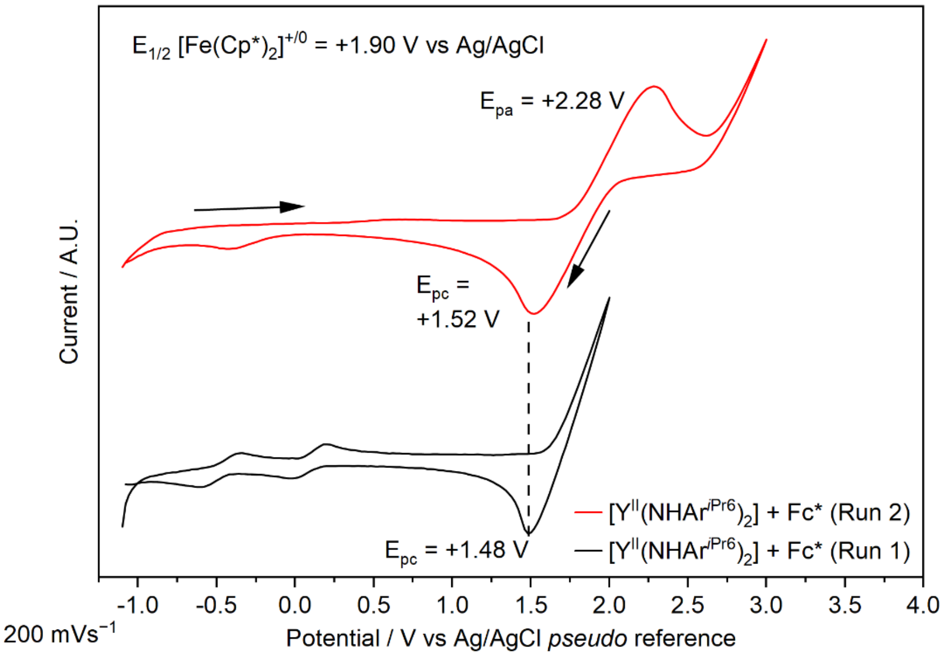

Figure S51. Cyclic voltammogram of **1Y** with Fc* internal standard in THF (10 mM) supported by [nBu$_4$N][BPh$_4$] (50 mM) vs Ag/AgCl pseudo reference at 200 mV s^{-1}.

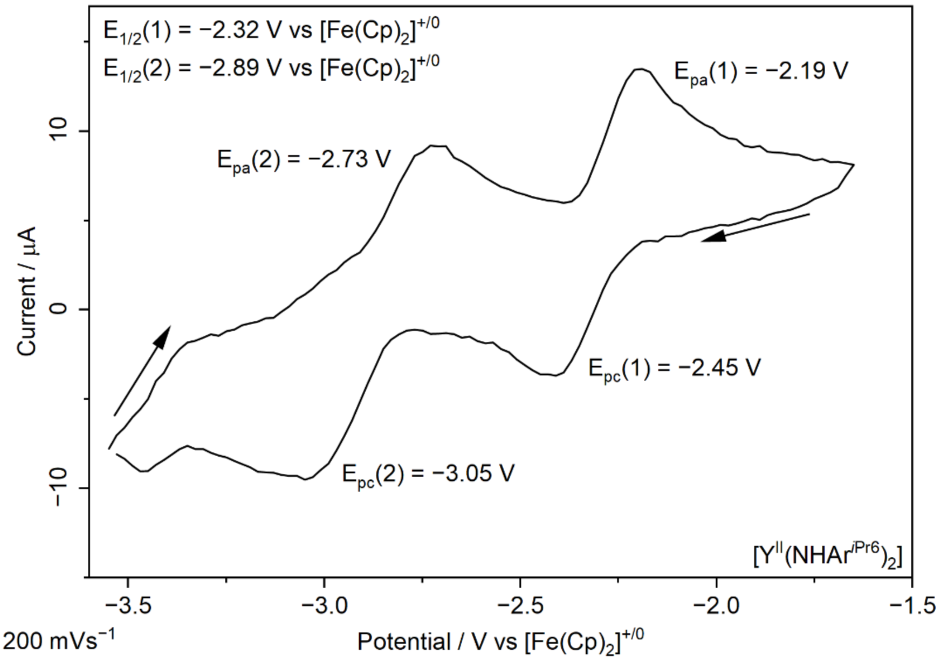

Figure S52. Cyclic voltammogram of **1Y** in THF (10 mM) supported by [nBu$_4$N][BPh$_4$] (50 mM) vs [Fc]$^{+/0}$ couple at 200 mV s^{-1}.

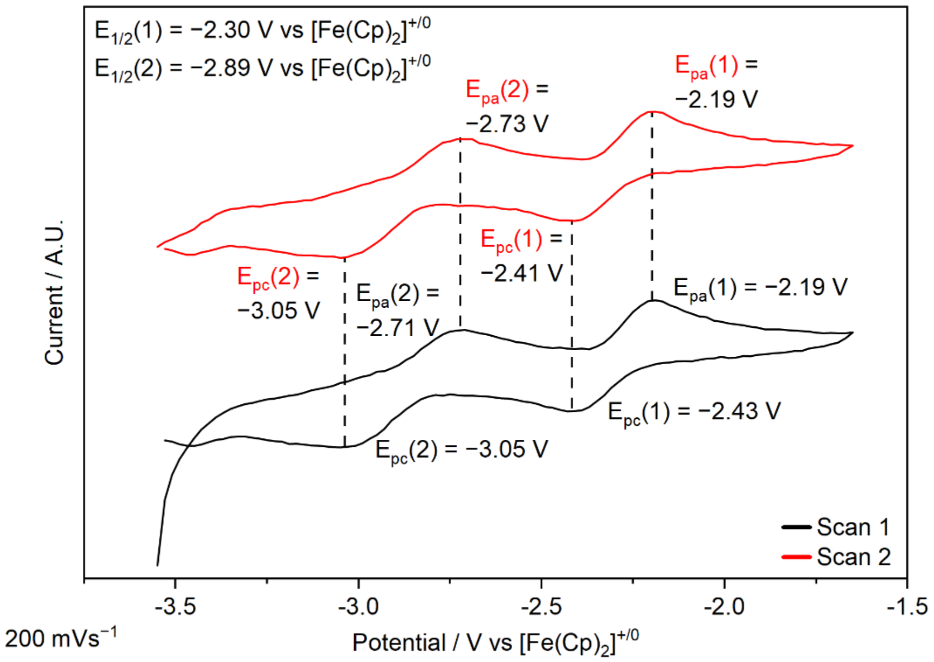

Figure S53. Cyclic voltammogram of **1Y** in THF (10 mM) supported by [*n*Bu4N][BPh4] (50 mM) vs $[Fc]^{+/0}$ couple at 200 mV s^{-1} with repeat scans to depict no electrochemical changes.

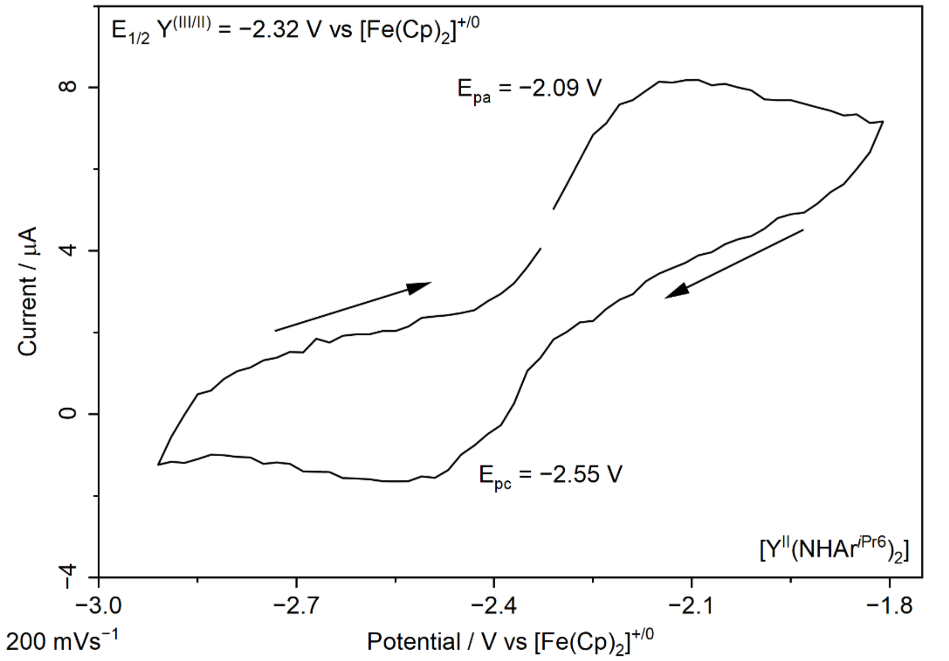

Figure S54. Cyclic voltammogram of **1Y** in THF (10 mM) supported by [*n*Bu4N][BPh4] (50 mM) vs $[Fc]^{+/0}$ couple at 200 mV s^{-1} of the $[\mathbf{1Y}]^{+/0}$ couple.

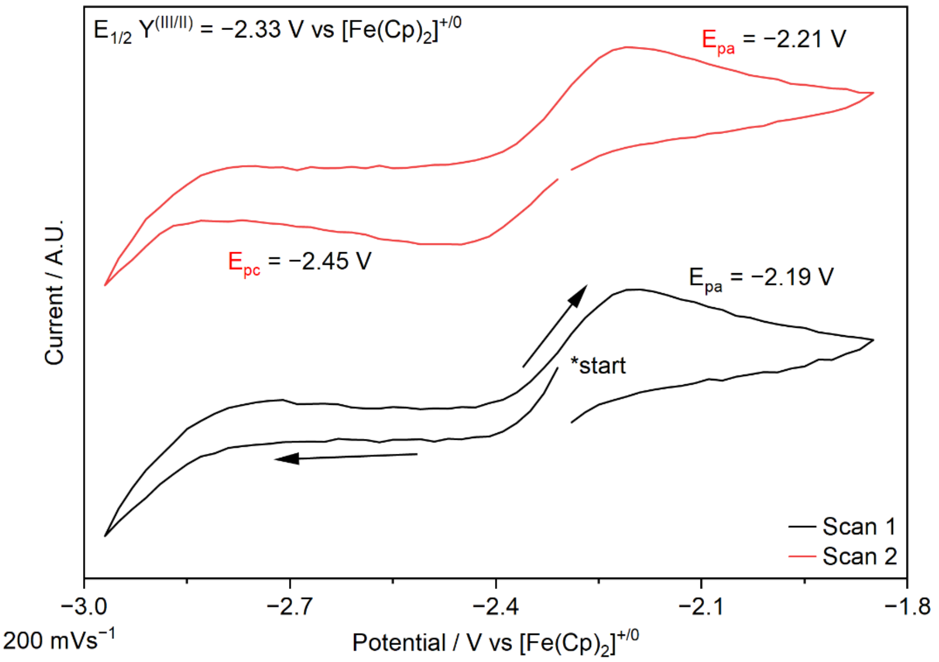

Figure S55. Cyclic voltammogram of **1Y** in THF (10 mM) supported by [*n*Bu4N][BPh4] (50 mM) vs $[Fc]^{+/0}$ couple at 200 mV s^{-1}. Scans indicate this is the $[\mathbf{1Y}]^{+/0}$ couple as Scan 1 shows no reduction event until the analyte is first oxidised.

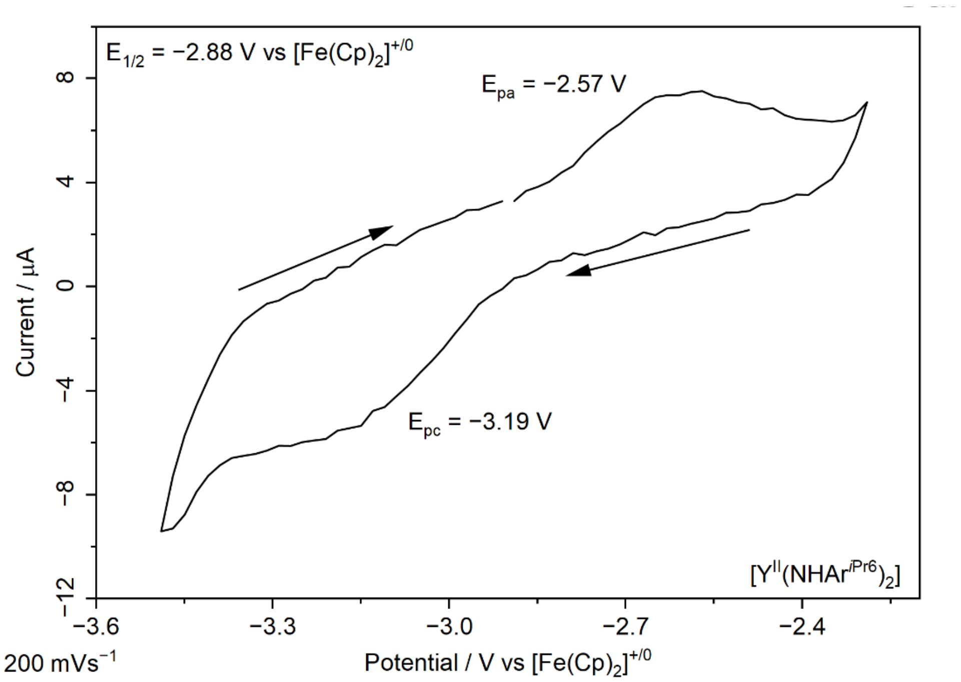

Figure S56. Cyclic voltammogram of **1Y** in THF (10 mM) supported by [*n*Bu4N][BPh4] (50 mM) vs $[Fc]^{+/0}$ couple at 200 mV s^{-1} of the second redox event.

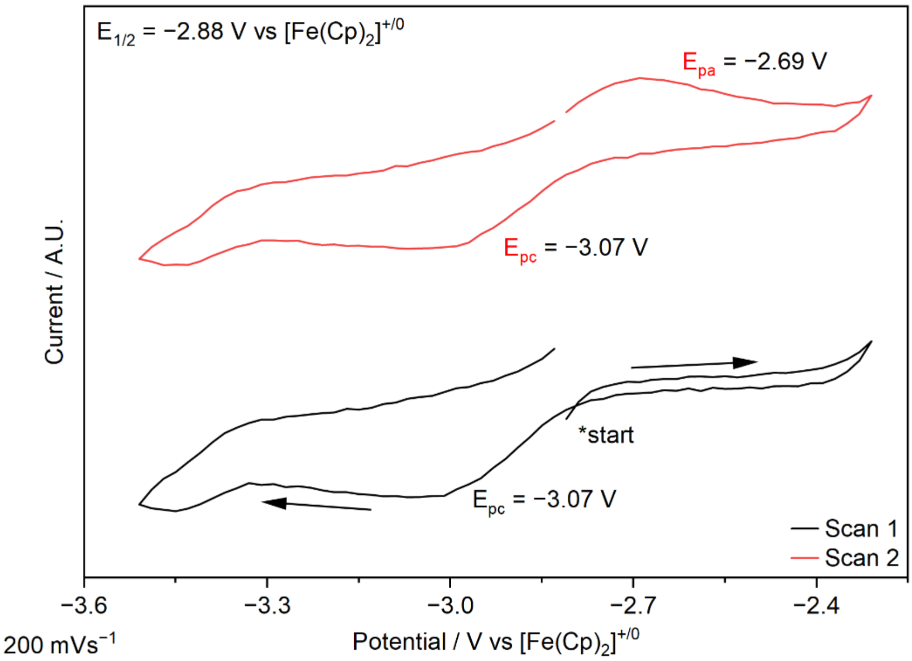

Figure S57. Cyclic voltammogram of **1Y** in THF (10 mM) supported by [*n*Bu4N][BPh4] (50 mM) vs $[Fc]^{+/0}$ couple at 200 mV s^{-1}. Scans indicate that the second redox event is a reduction derived event as Scan 1 shows no oxidation event until the analyte is first reduced.

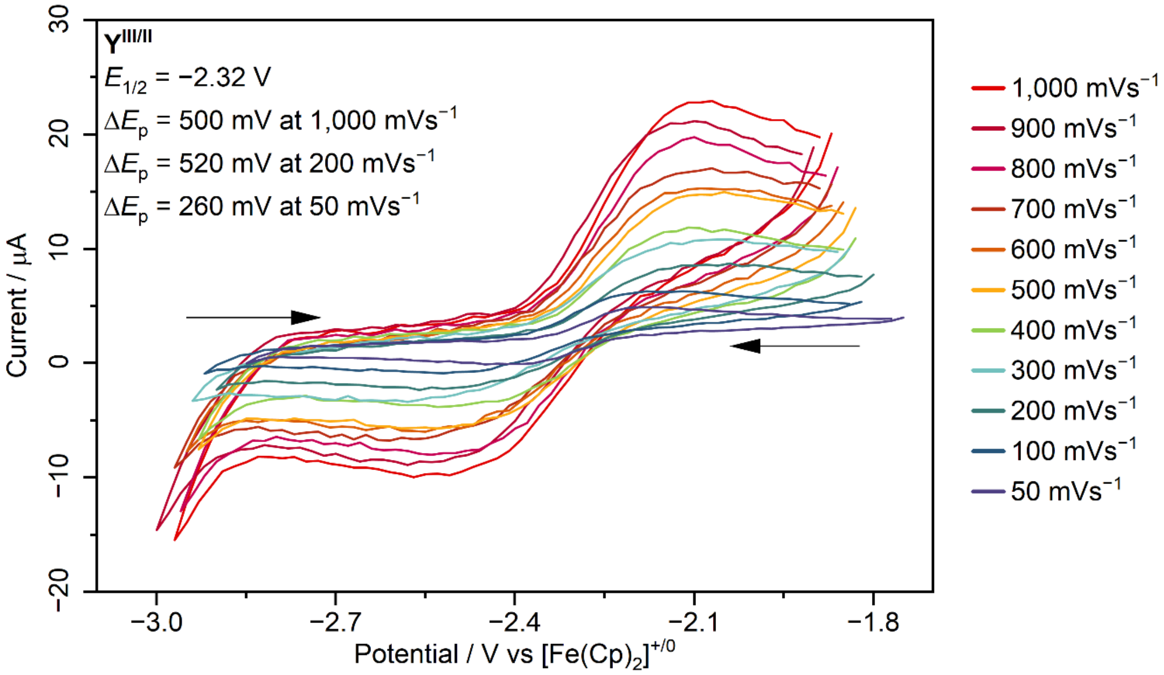

Figure S58. Scan-rate dependence of cyclic voltammogram of **1Y** in THF (10 mM) supported by [*n*Bu4N][BPh4] (50 mM) vs $[Fc]^{+/0}$ couple of the $[\mathbf{1Y}]^{+/0}$ couple.

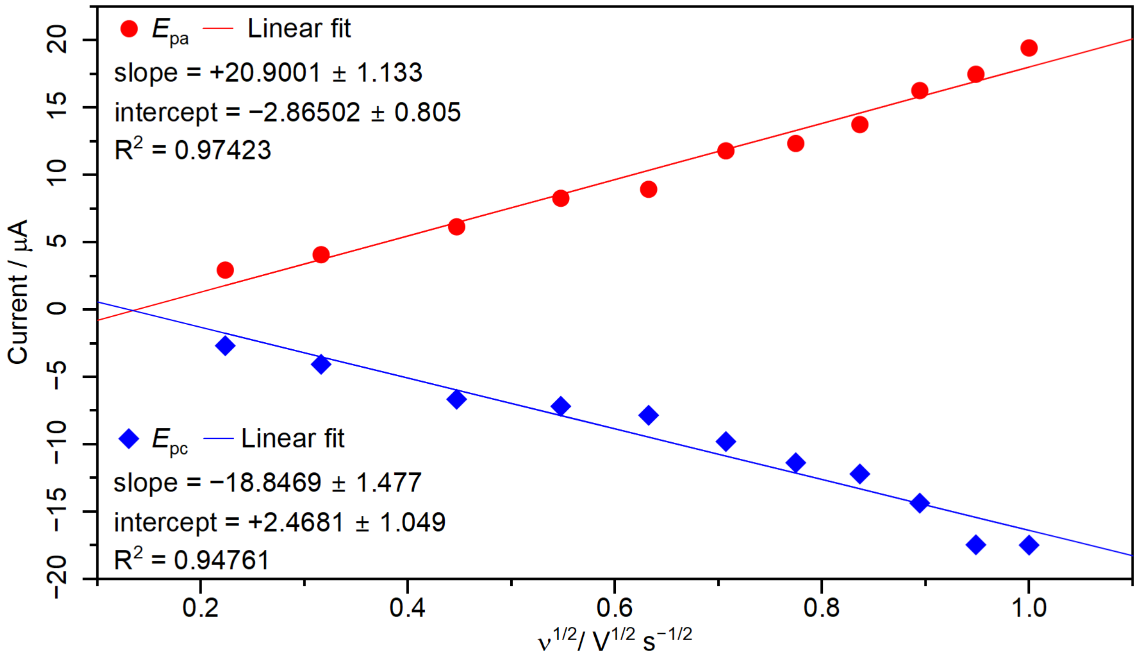

Figure S59. Randles-Sevcik plot of the [**1Y**]$^{+/0}$ couple of **1Y**.

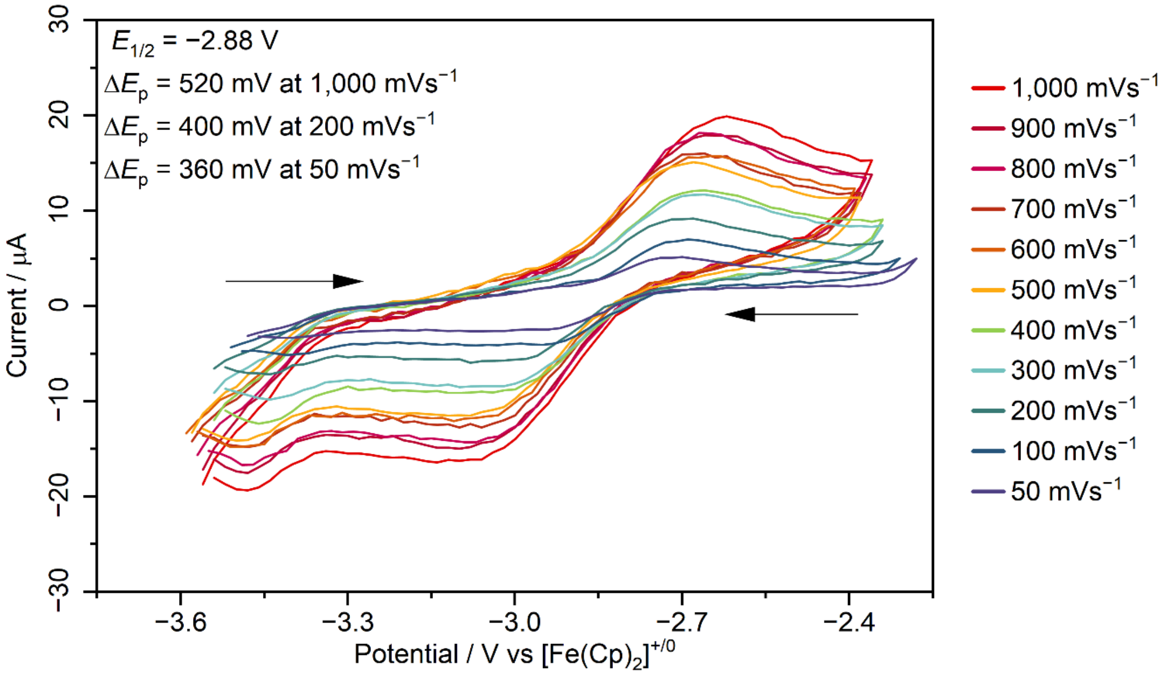

Figure S60. Scan-rate dependence of cyclic voltammogram of **1Y** in THF (10 mM) supported by [*n*Bu$_4$N][BPh$_4$] (50 mM) vs [Fc]$^{+/0}$ couple of the second redox event.

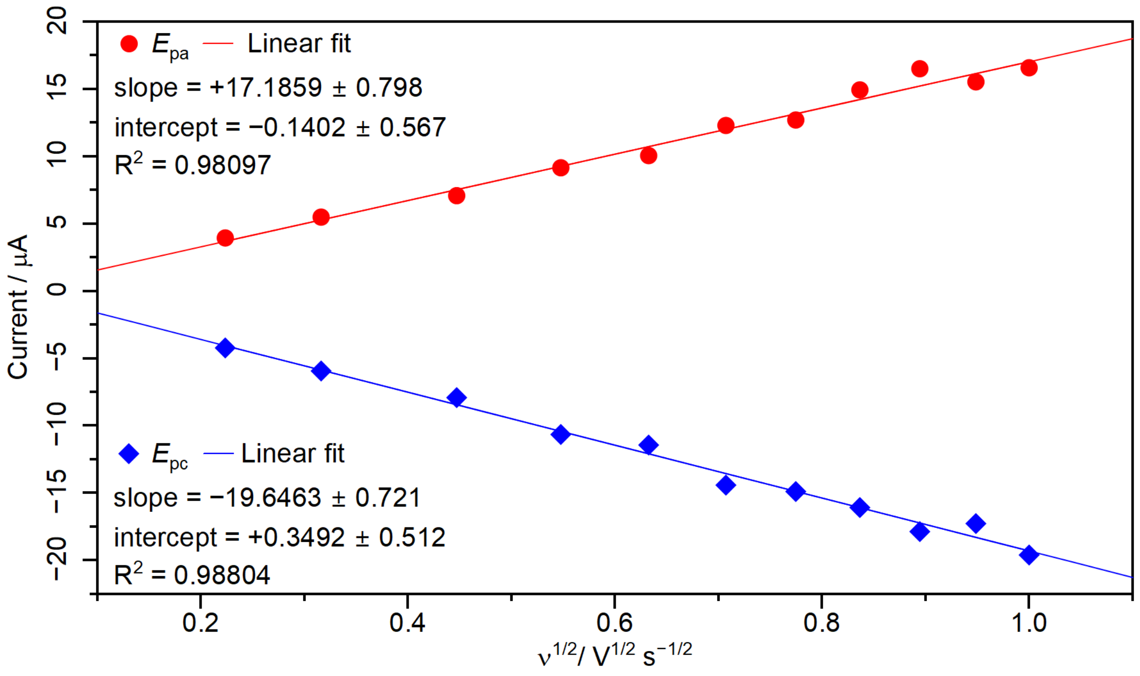

Figure S61. Randles-Sevcik plot of the second redox event of **1Y**.

***1La** – [La^{II}(NHAr^{iPr6})$_2$]*

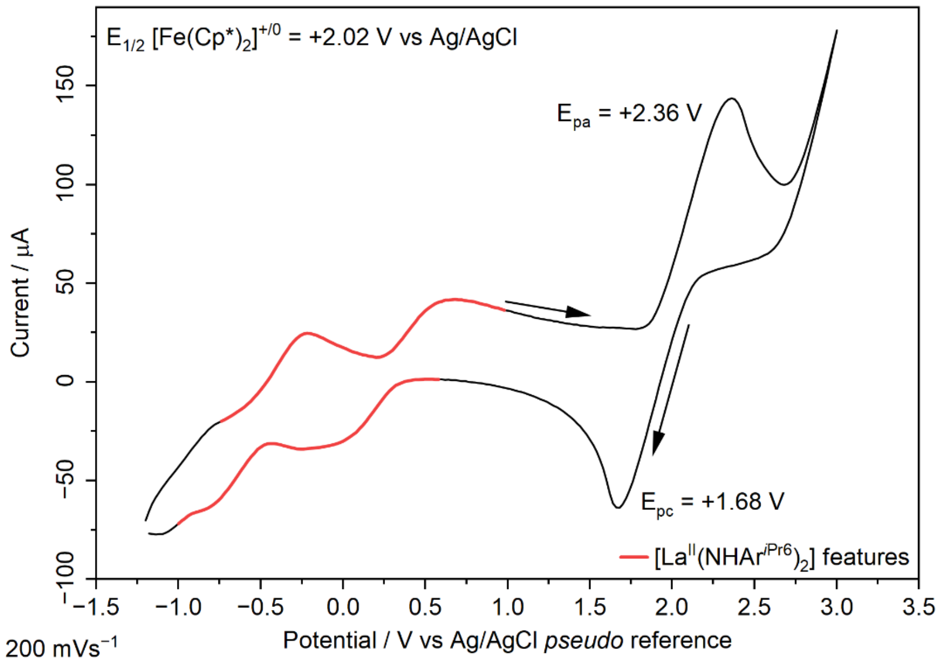

Figure S62. Cyclic voltammogram of **1La** with Fc* internal standard in THF (10 mM) supported by [nBu$_4$N][BPh$_4$] (50 mM) vs Ag/AgCl pseudo reference at 200 mV s^{-1}.

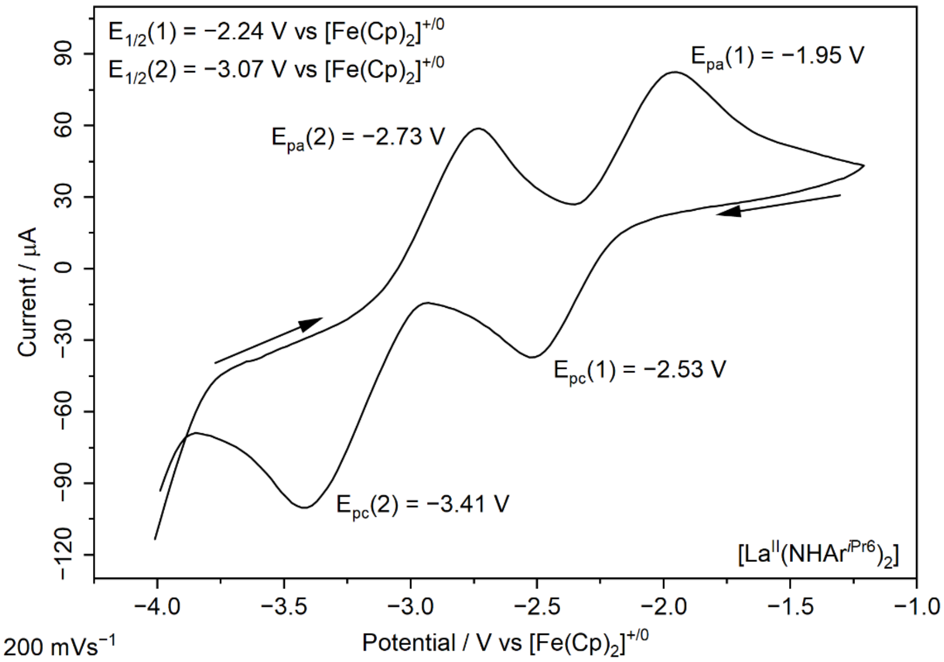

Figure S63. Cyclic voltammogram of **1La** in THF (10 mM) supported by [*n*Bu4N][BPh4] (50 mM) vs $[Fc]^{+/0}$ couple at 200 mV s^{-1}.

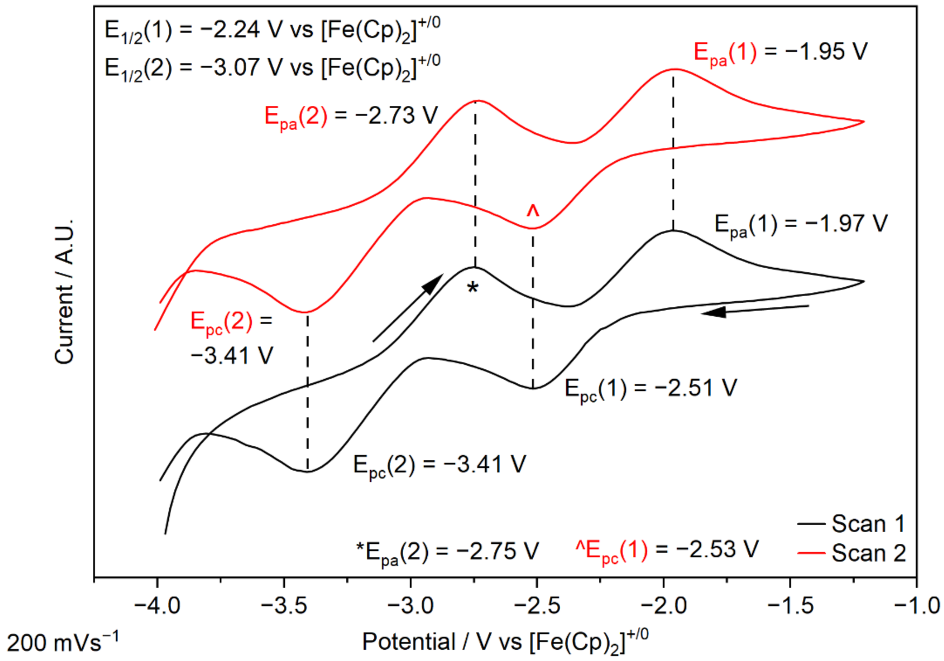

Figure S64. Cyclic voltammogram of **1La** in THF (10 mM) supported by [*n*Bu4N][BPh4] (50 mM) vs $[Fc]^{+/0}$ couple at 200 mV s^{-1} with repeat scans to depict no electrochemical changes.

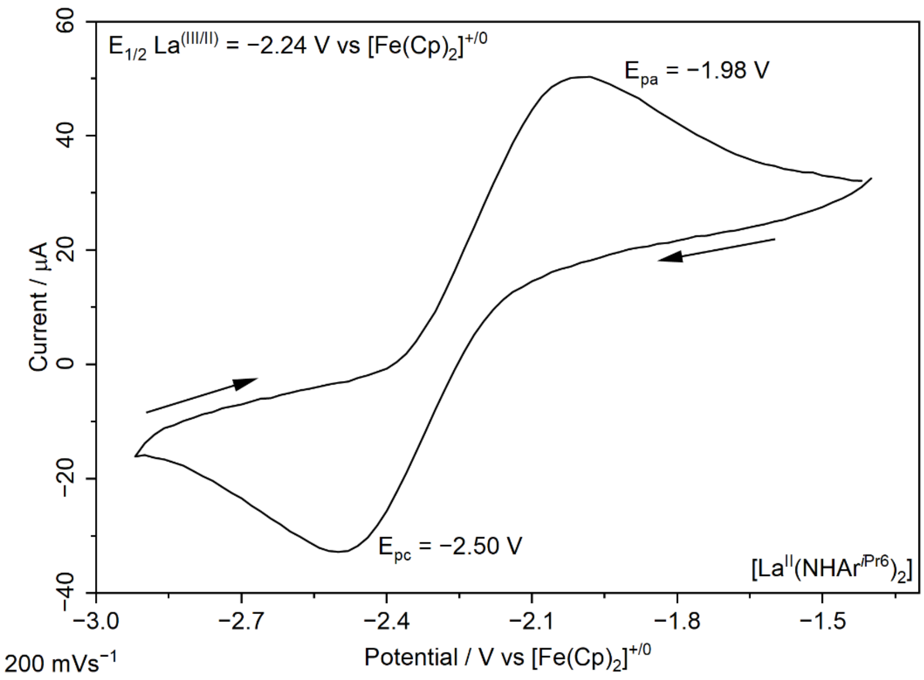

Figure S65. Cyclic voltammogram of **1La** in THF (10 mM) supported by [*n*Bu4N][BPh4] (50 mM) vs $[Fc]^{+/0}$ couple at 200 mV s^{-1} of the $[\mathbf{1La}]^{+/0}$ couple.

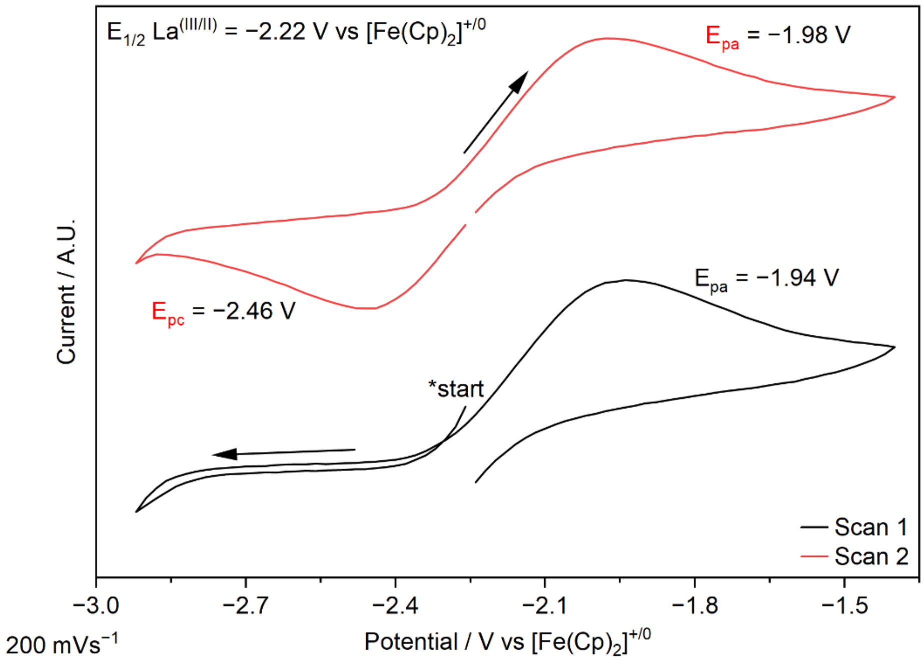

Figure S66. Cyclic voltammogram of **1La** in THF (10 mM) supported by [*n*Bu4N][BPh4] (50 mM) vs $[Fc]^{+/0}$ couple at 200 mV s^{-1}. Scans indicate this is the $[\mathbf{1La}]^{+/0}$ couple as Scan 1 shows no reduction event until the analyte is first oxidised.

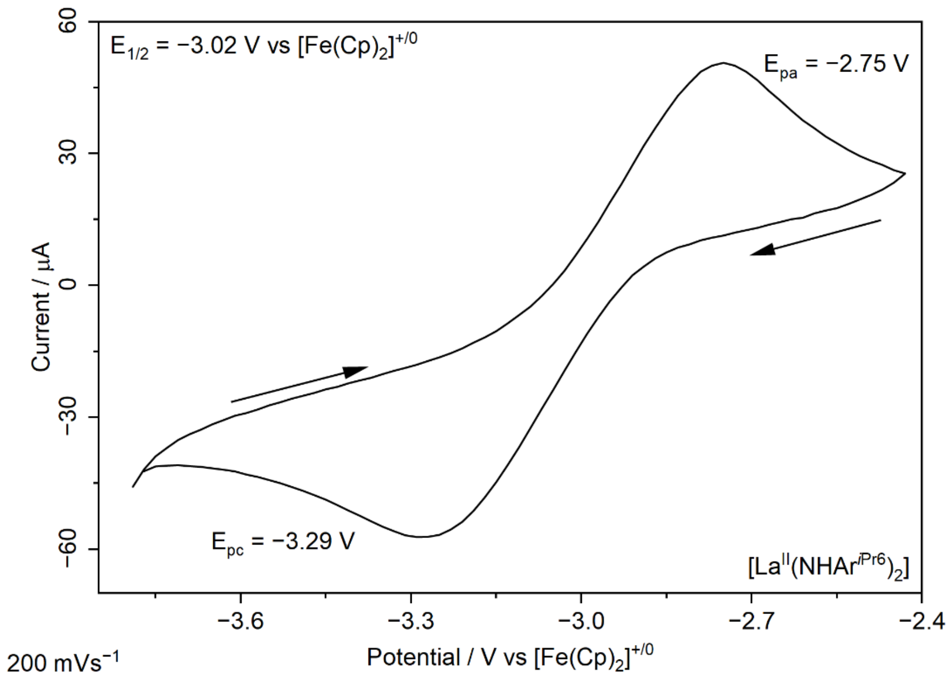

Figure S67. Cyclic voltammogram of **1La** in THF (10 mM) supported by [*n*Bu4N][BPh4] (50 mM) vs [Fc]$^{+/0}$ couple at 200 mV s^{-1} of the second redox event.

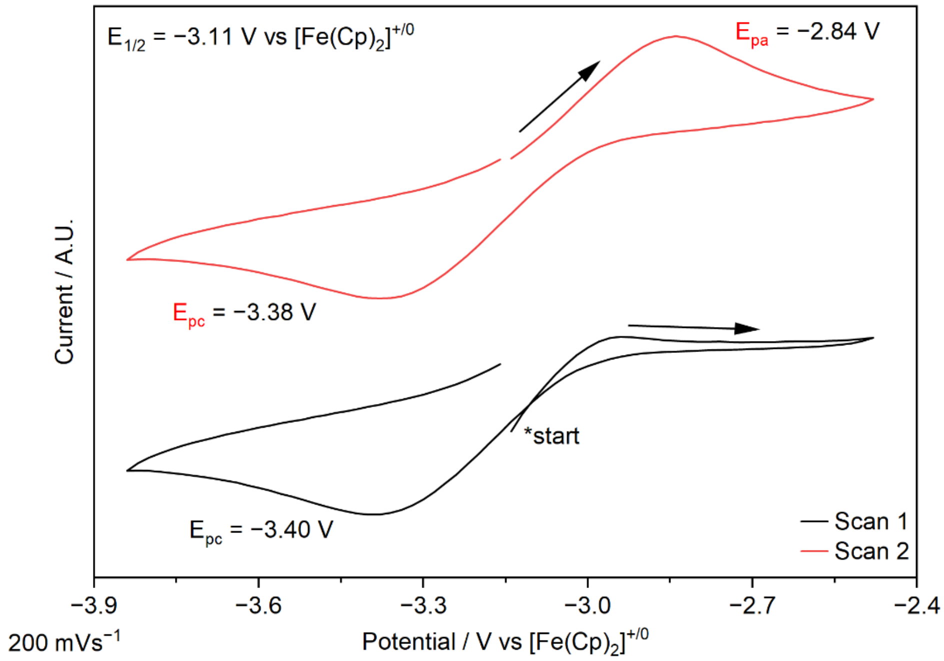

Figure S68. Cyclic voltammogram of **1La** in THF (10 mM) supported by [*n*Bu4N][BPh4] (50 mM) vs [Fc]$^{+/0}$ couple at 200 mV s^{-1}. Scans indicate that the second redox event is a reduction derived event as Scan 1 shows no oxidation event until the analyte is first reduced.

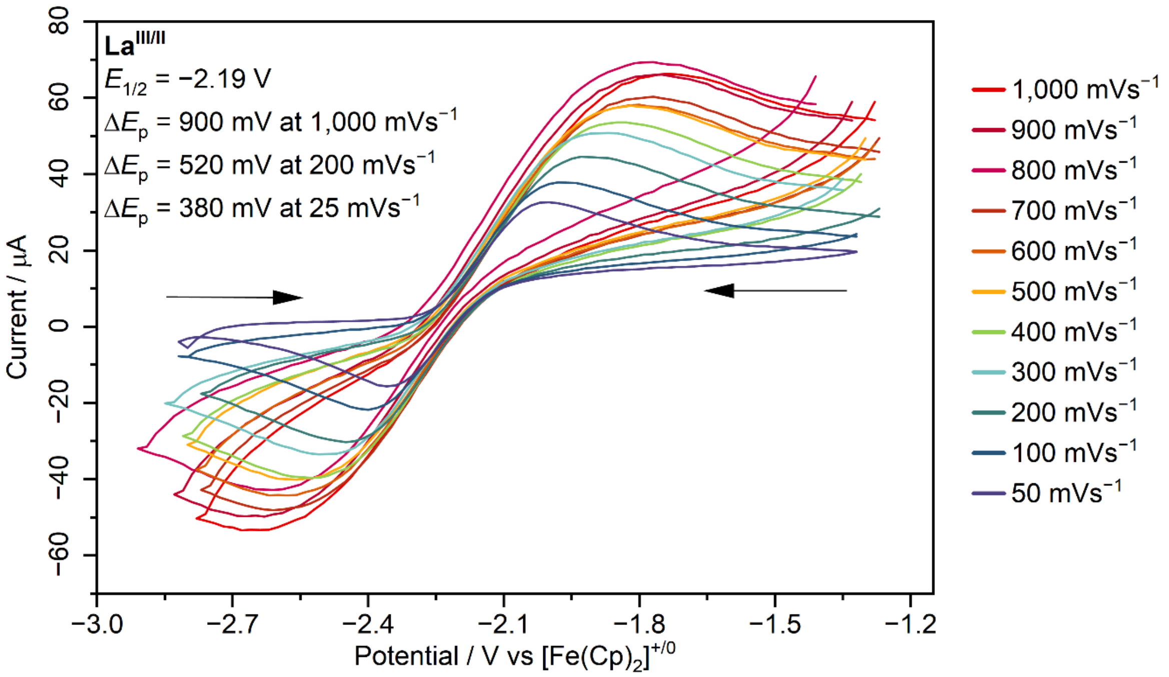

Figure S69. Scan-rate dependence of cyclic voltammogram of **1La** in THF (10 mM) supported by [nBu$_4$N][BPh$_4$] (50 mM) vs [Fc]$^{+/0}$ couple of the [**1La**]$^{+/0}$ couple.

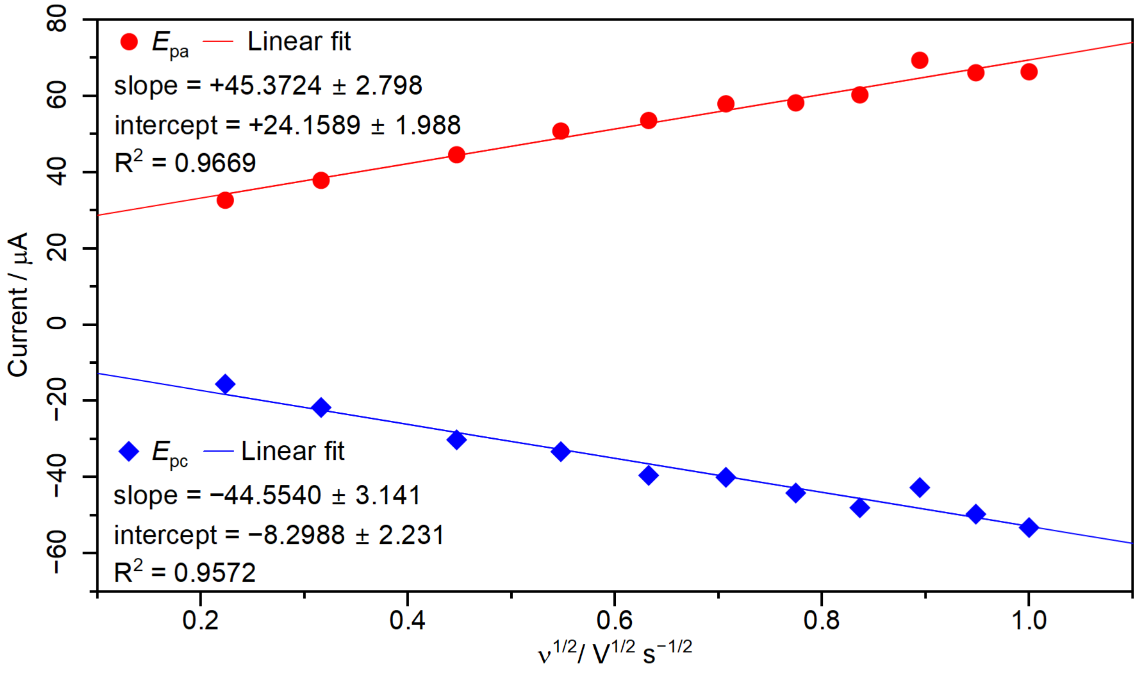

Figure S70. Randles-Sevcik plot of the [**1La**]$^{+/0}$ couple of **1La**.

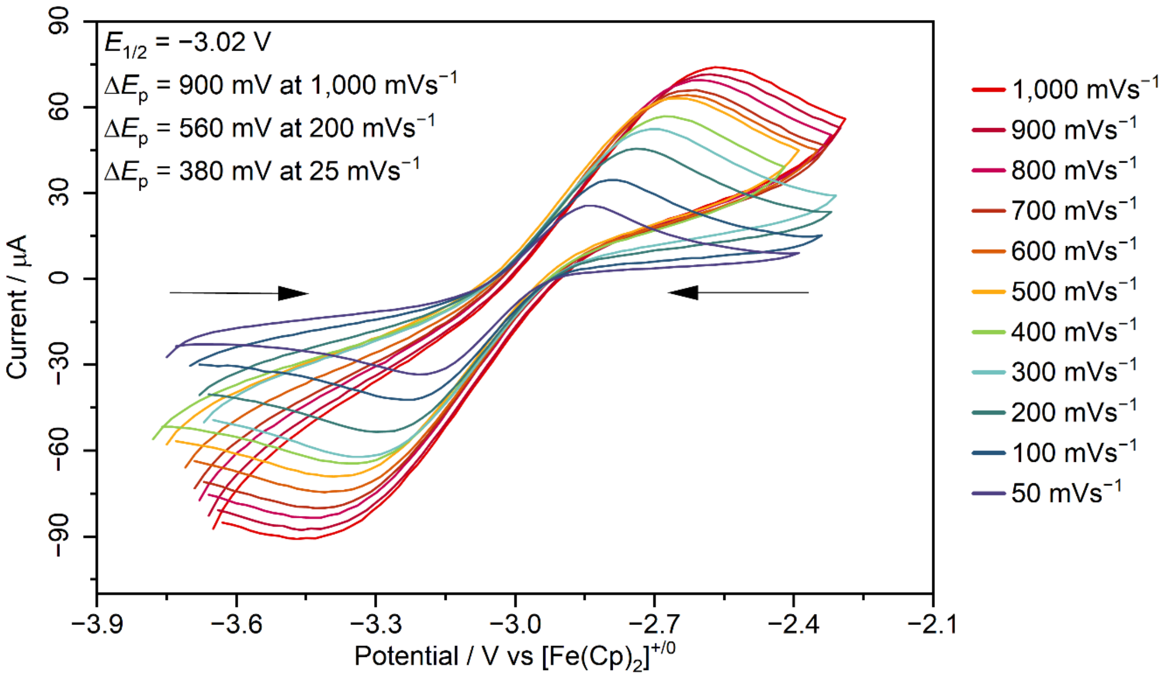

Figure S71. Scan-rate dependence of cyclic voltammogram of **1La** in THF (10 mM) supported by $[n\text{Bu}_4\text{N}][\text{BPh}_4]$ (50 mM) vs $[\text{Fc}]^{+/0}$ couple of the second redox event.

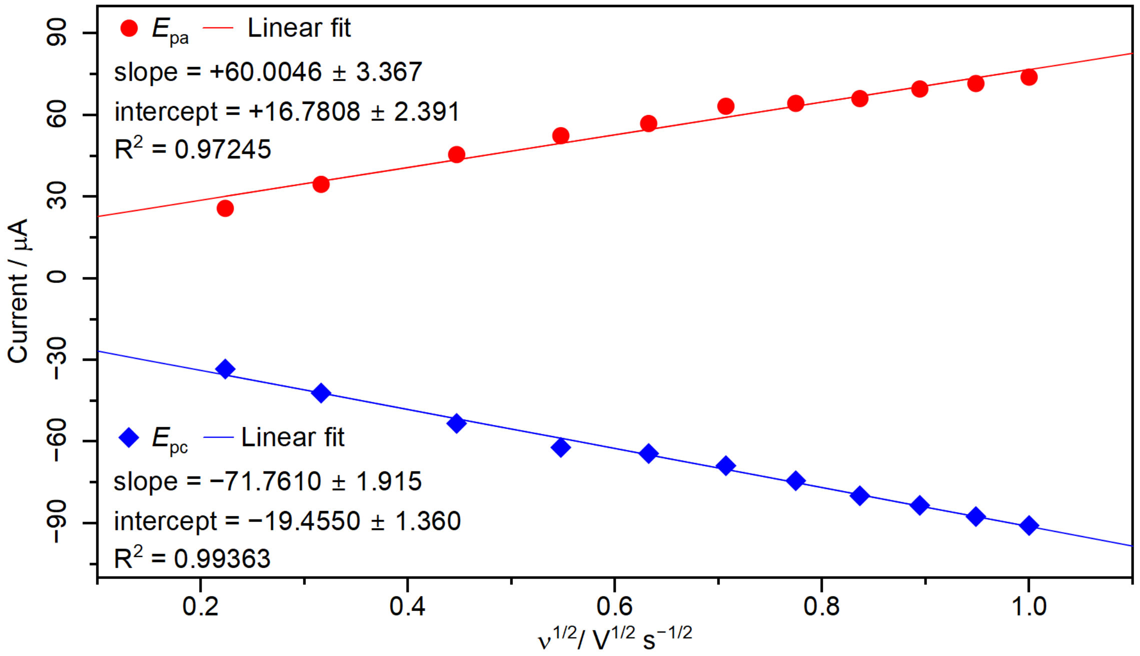

Figure S72. Randles-Sevcik plot of the second redox event of **1La**.

***1Lu** – [Lu^II^(NHAr^iPr6^)~2~]*

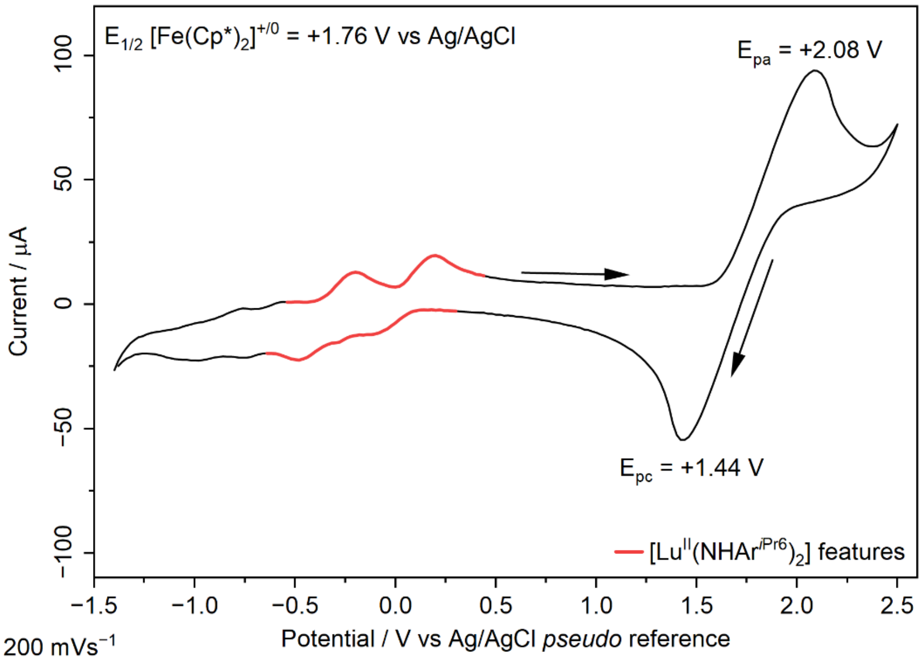

Figure S73. Cyclic voltammogram of **1Lu** with Fc* internal standard in THF (10 mM) supported by [nBu$_4$N][BPh$_4$] (50 mM) vs Ag/AgCl pseudo reference at 200 mV s^{-1}.

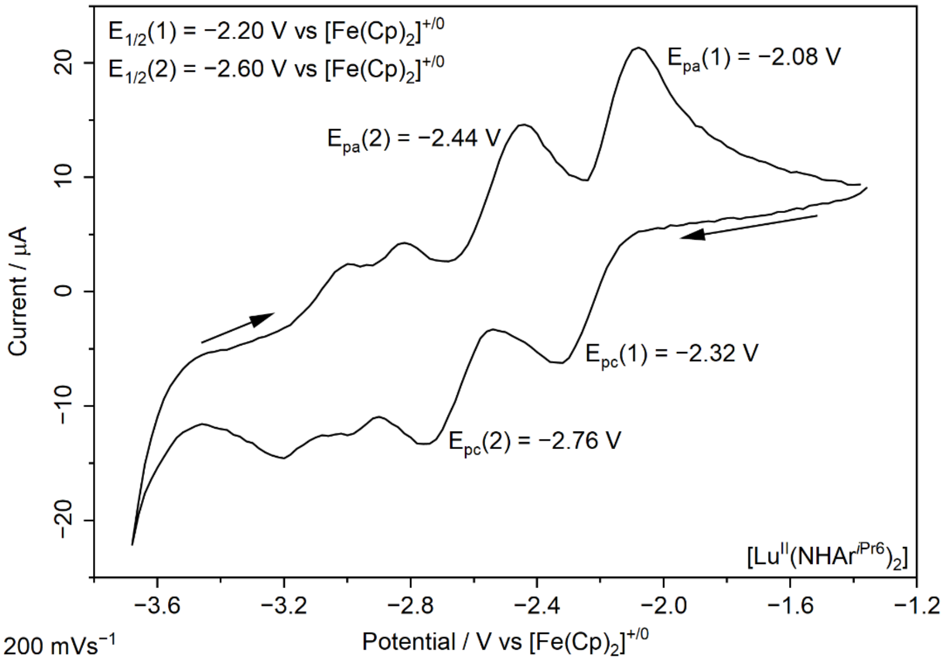

Figure S74. Cyclic voltammogram of **1Lu** in THF (10 mM) supported by [nBu$_4$N][BPh$_4$] (50 mM) vs [Fc]$^{+/0}$ couple at 200 mV s^{-1}.

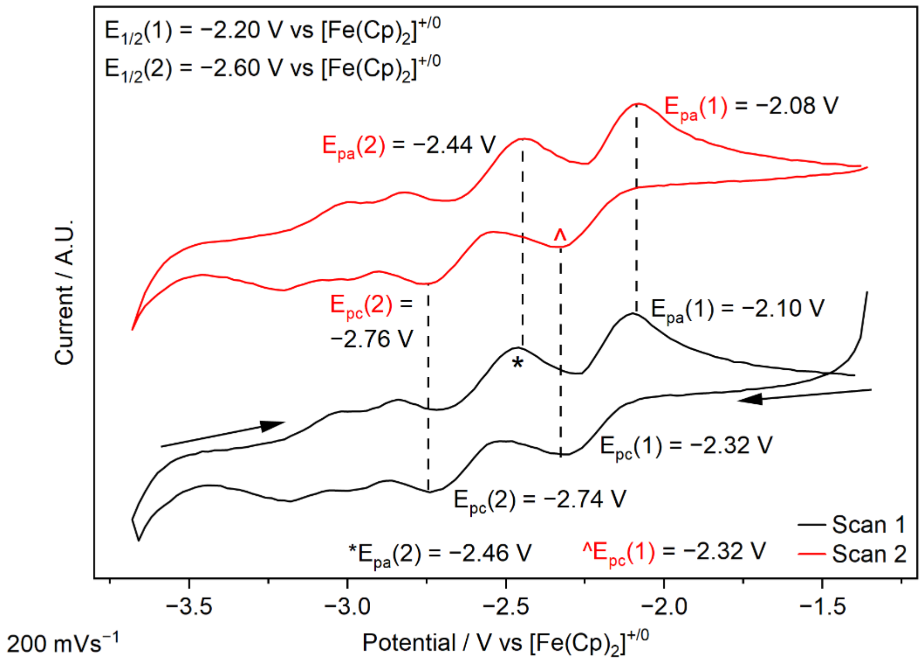

Figure S75. Cyclic voltammogram of **1Lu** in THF (10 mM) supported by [nBu$_4$N][BPh$_4$] (50 mM) vs $[Fc]^{+/0}$ couple at 200 mV s^{-1} with repeat scans to depict no electrochemical changes.

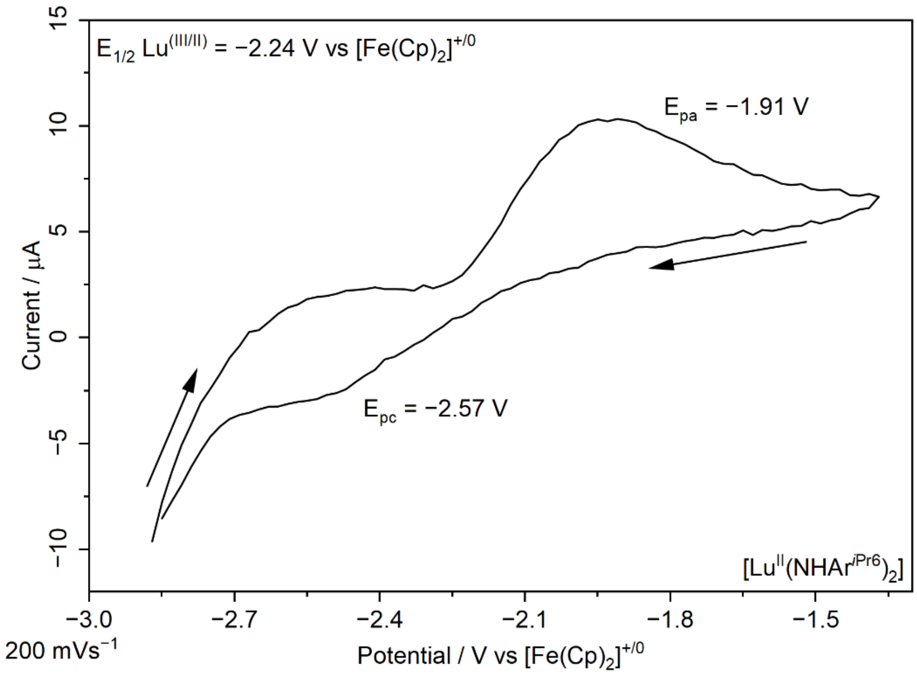

Figure S76. Cyclic voltammogram of **1Lu** in THF (10 mM) supported by [nBu$_4$N][BPh$_4$] (50 mM) vs $[Fc]^{+/0}$ couple at 200 mV s^{-1} of the $[\mathbf{1Lu}]^{+/0}$ couple.

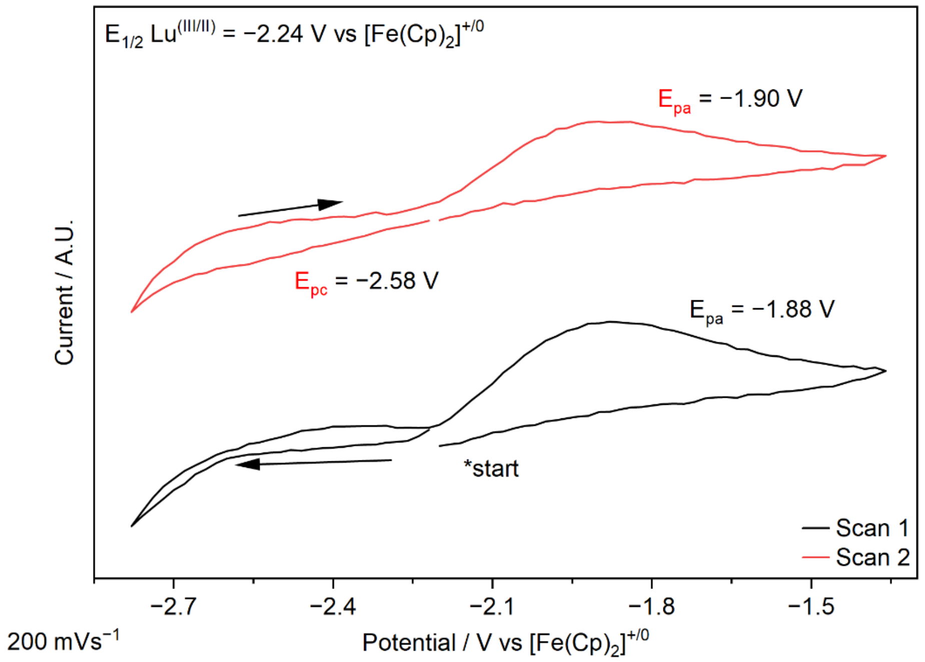

Figure S77. Cyclic voltammogram of **1Lu** in THF (10 mM) supported by [*n*Bu4N][BPh4] (50 mM) vs $[Fc]^{+/0}$ couple at 200 mV s^{-1}. Scans indicate this is the $[\mathbf{1Lu}]^{+/0}$ couple as Scan 1 shows no reduction event until the analyte is first oxidised.

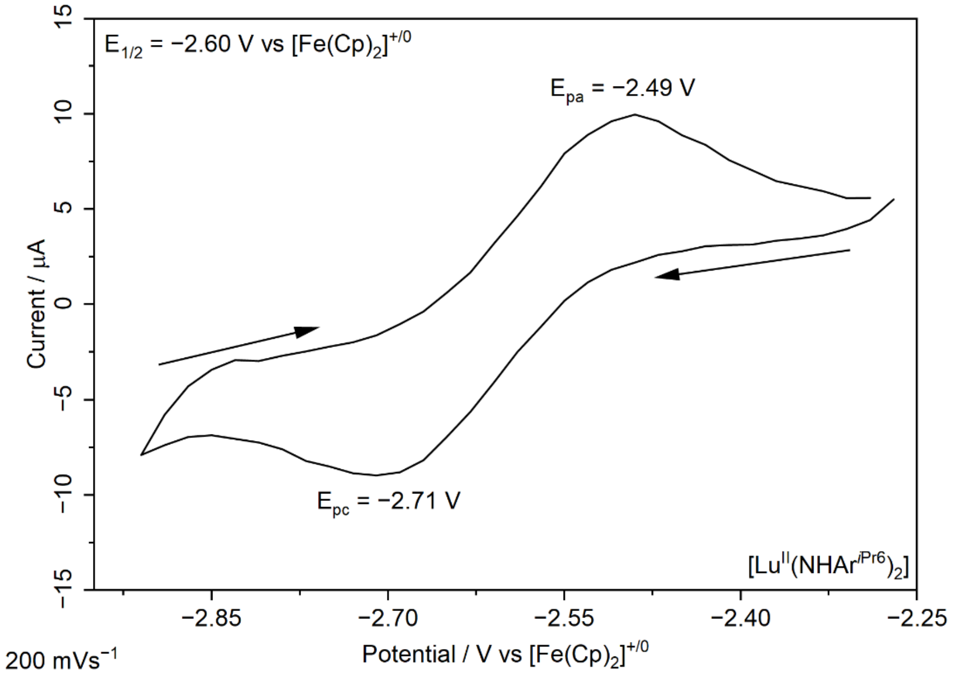

Figure S78. Cyclic voltammogram of **1Lu** in THF (10 mM) supported by [*n*Bu4N][BPh4] (50 mM) vs $[Fc]^{+/0}$ couple at 200 mV s^{-1} of the second redox event.

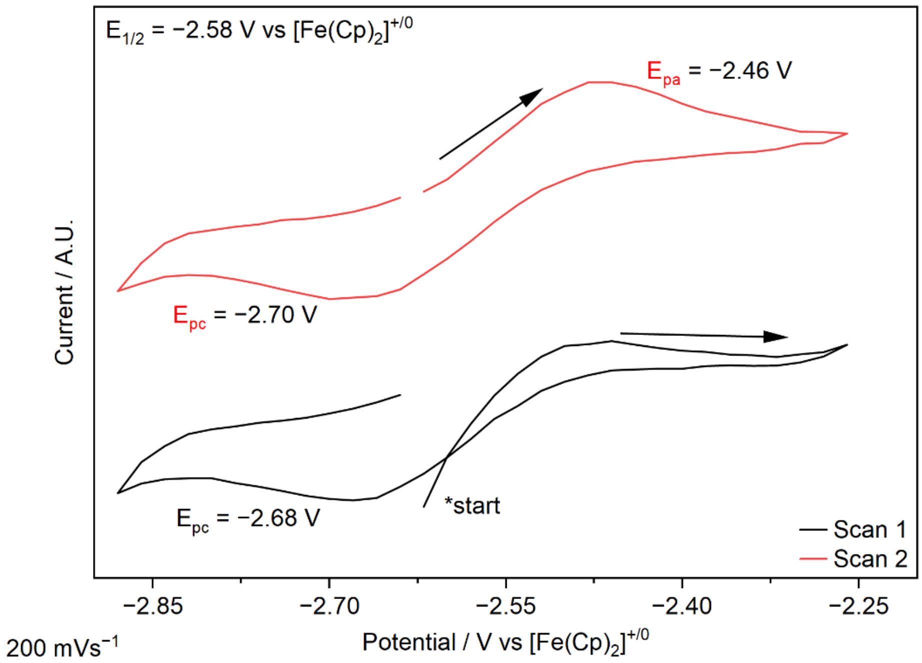

Figure S79. Cyclic voltammogram of **1Lu** in THF (10 mM) supported by [nBu$_4$N][BPh$_4$] (50 mM) vs $[Fc]^{+/0}$ couple at 200 mV s^{-1}. Scans indicate that the second redox event is a reduction derived event as Scan 1 shows no oxidation event until the analyte is first reduced.

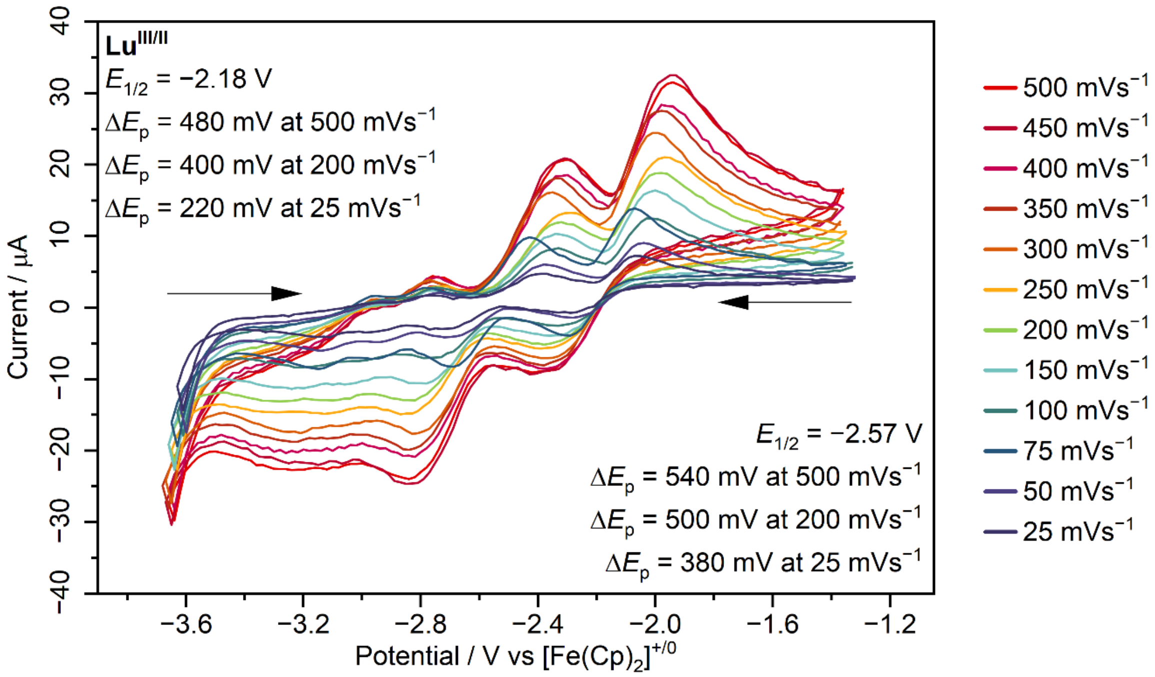

Figure S80. Scan-rate dependence of cyclic voltammogram of **1Lu** in THF (10 mM) supported by [nBu$_4$N][BPh$_4$] (50 mM) vs $[Fc]^{+/0}$ couple with scan rates from 25 mV s^{-1} to 500 mV s^{-1}.

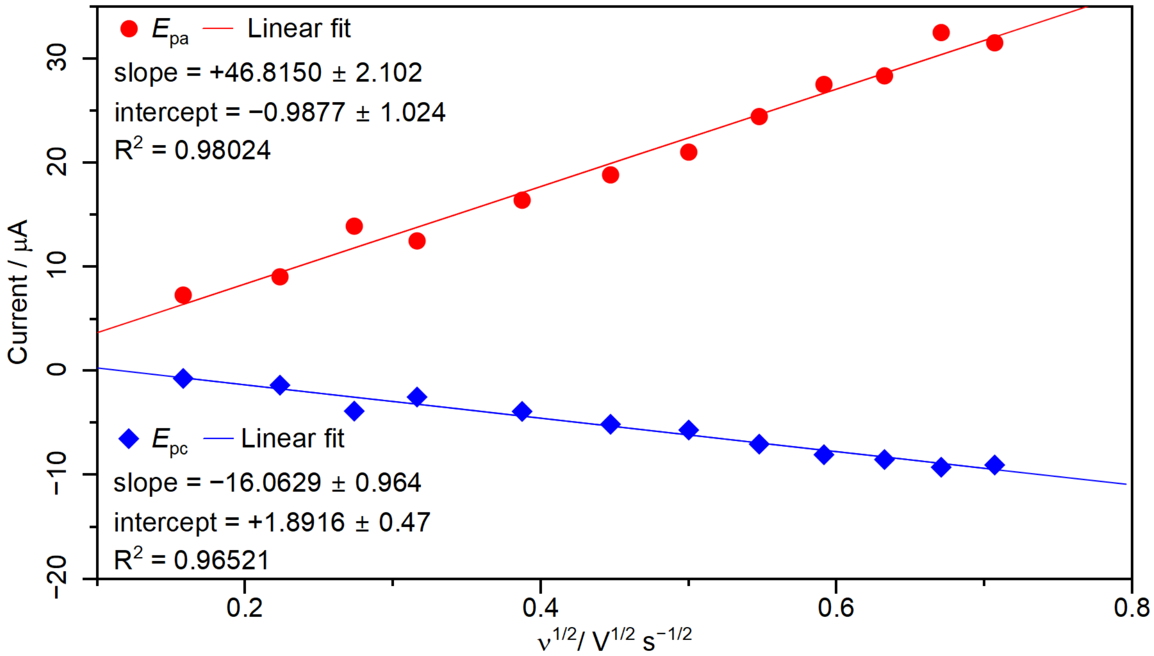

Figure S81. Randles-Sevcik plot of the [**1Lu**]$^{+/0}$ couple of **1Lu** from the scan-rate dependence of 25 mV s^{-1} to 500 mV s^{-1} scan rates.

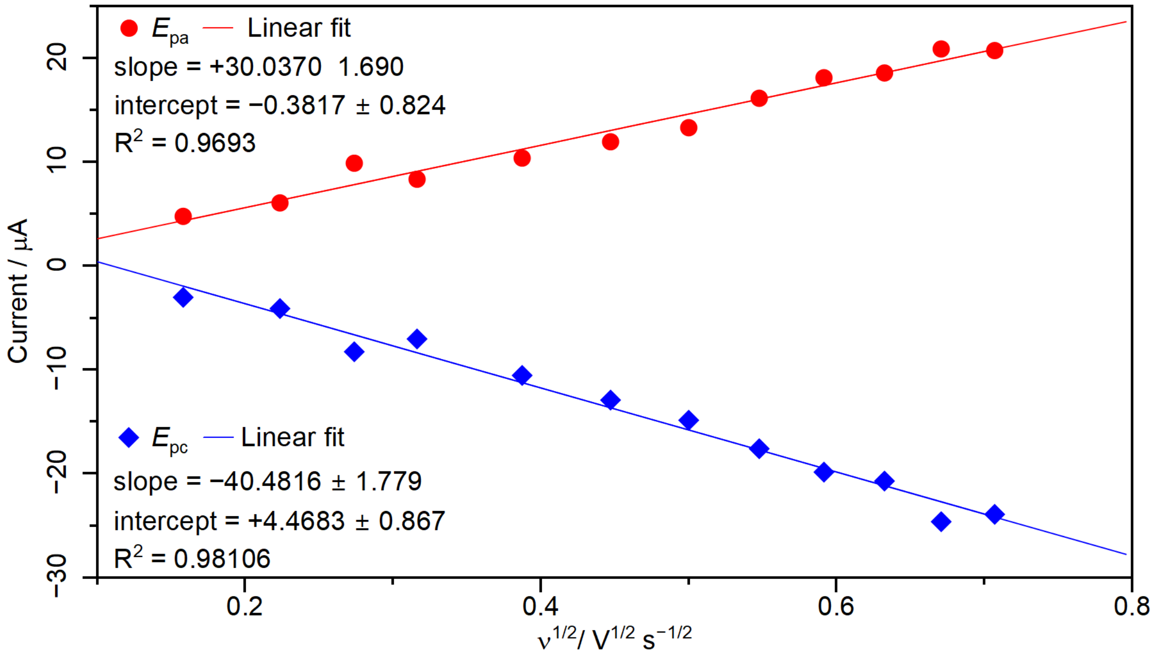

Figure S82. Randles-Sevcik plot of the second redox event of **1Lu** from the scan-rate dependence of 25 mV s^{-1} to 500 mV s^{-1} scan rates.

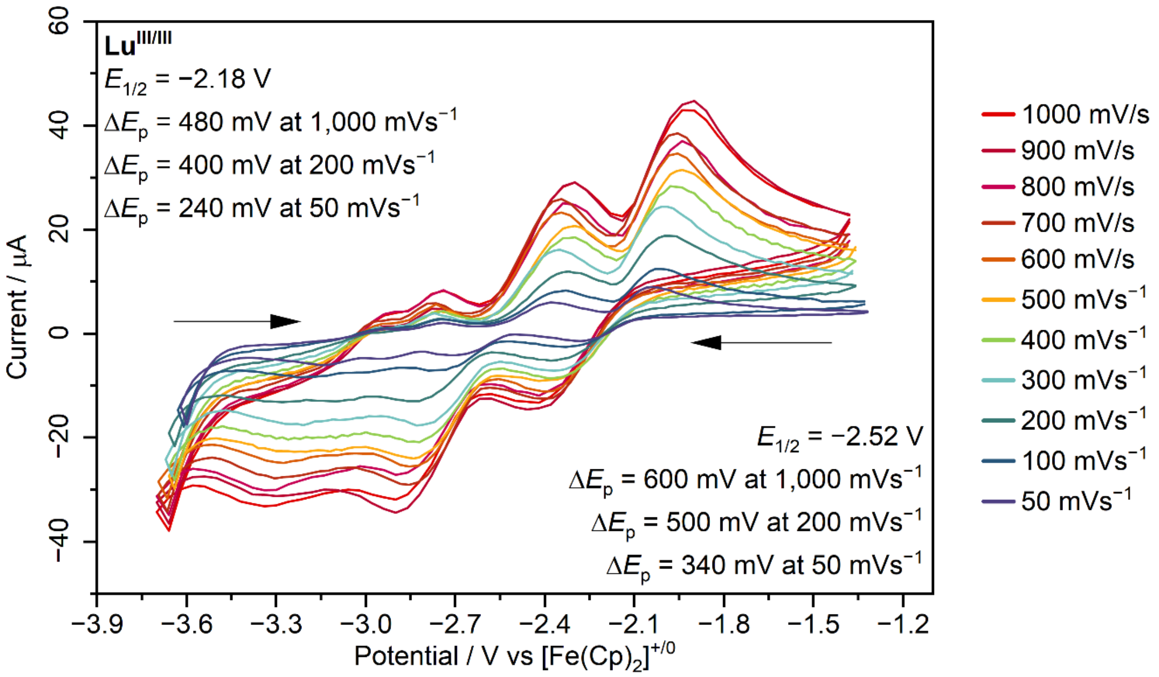

Figure S83. Scan-rate dependence of cyclic voltammogram of **1Lu** in THF (10 mM) supported by $[nBu_4N][BPh_4]$ (50 mM) vs $[Fc]^{+/0}$ with scan rates from 50 mV s^{-1} to 1,000 mV s^{-1}.

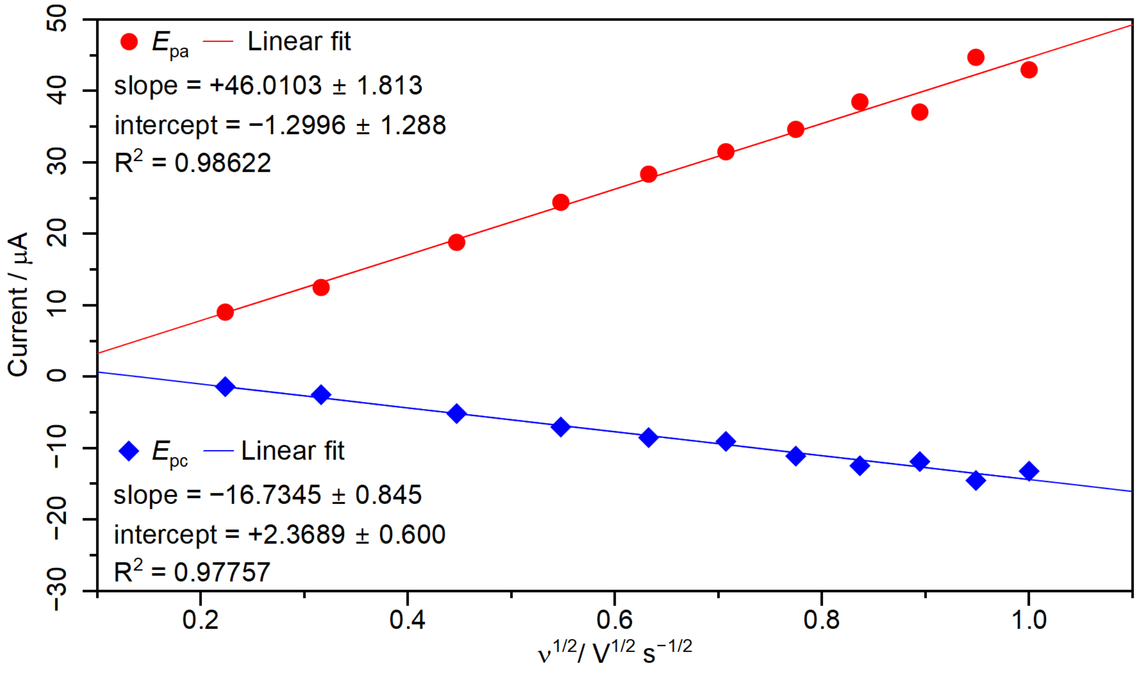

Figure S84. Randles-Sevcik plot of the $[\mathbf{1Lu}]^{+/0}$ couple of **1Lu** from the scan-rate dependence of 50 mV s^{-1} to 1,000 mV s^{-1} scan rates.

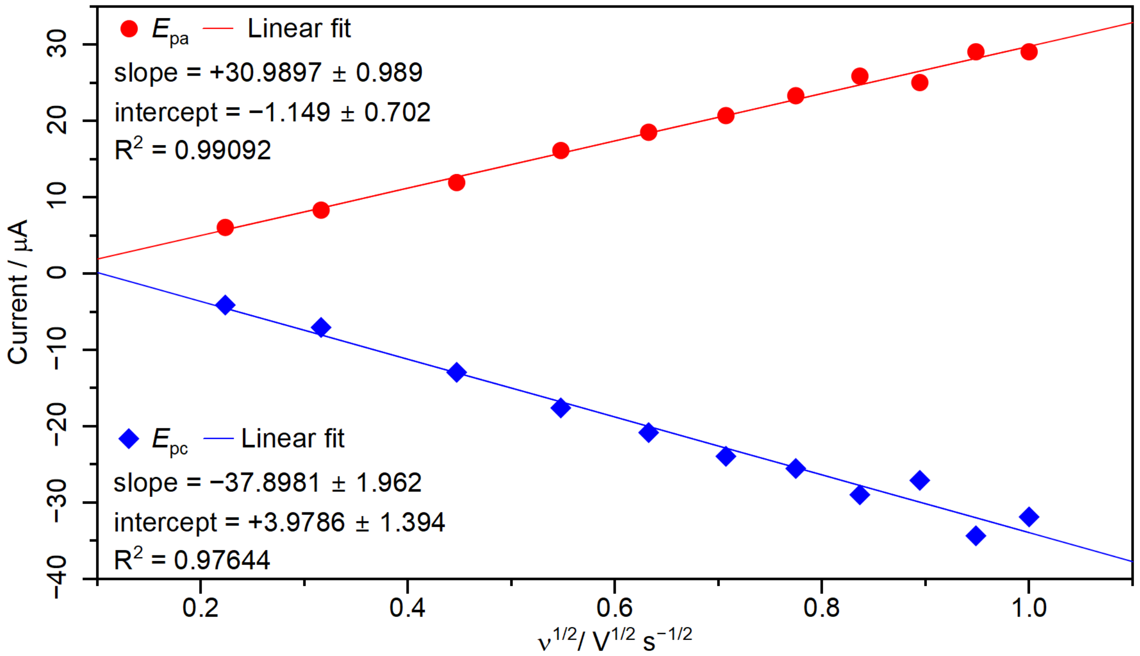

Figure S85. Randles-Sevcik plot of the second redox event of **1Lu** from the scan-rate dependence of 50 mV s^{-1} to 1,000 mV s^{-1} scan rates.

1Tm *– [$Tm^{II}(NHAr^{iPr6})_2$]*

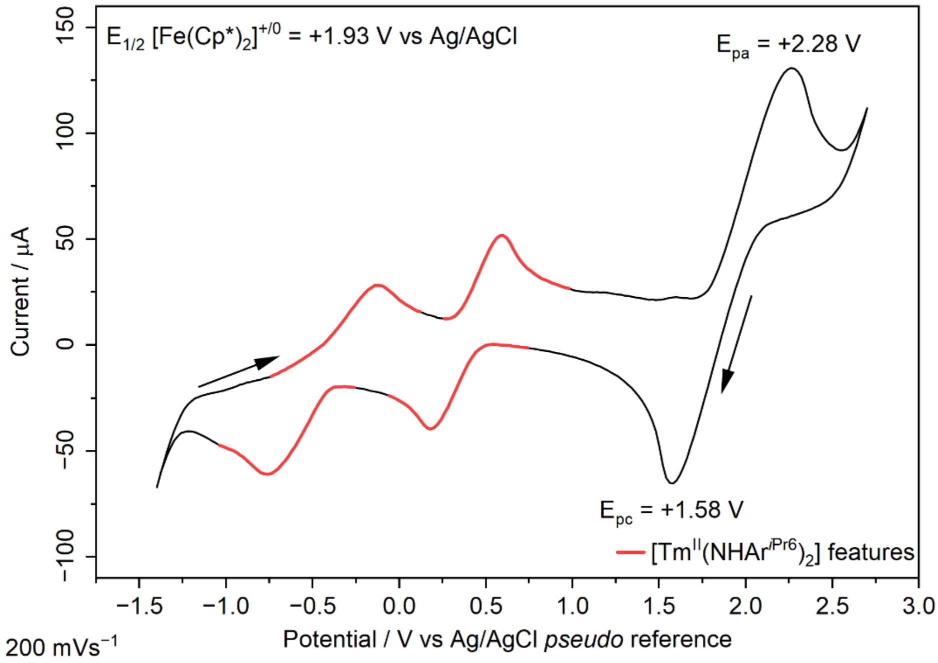

Figure S86. Cyclic voltammogram of **1Tm** with Fc* internal standard in THF (10 mM) supported by [nBu$_4$N][BPh$_4$] (50 mM) vs Ag/AgCl pseudo reference at 200 mV s^{-1}.

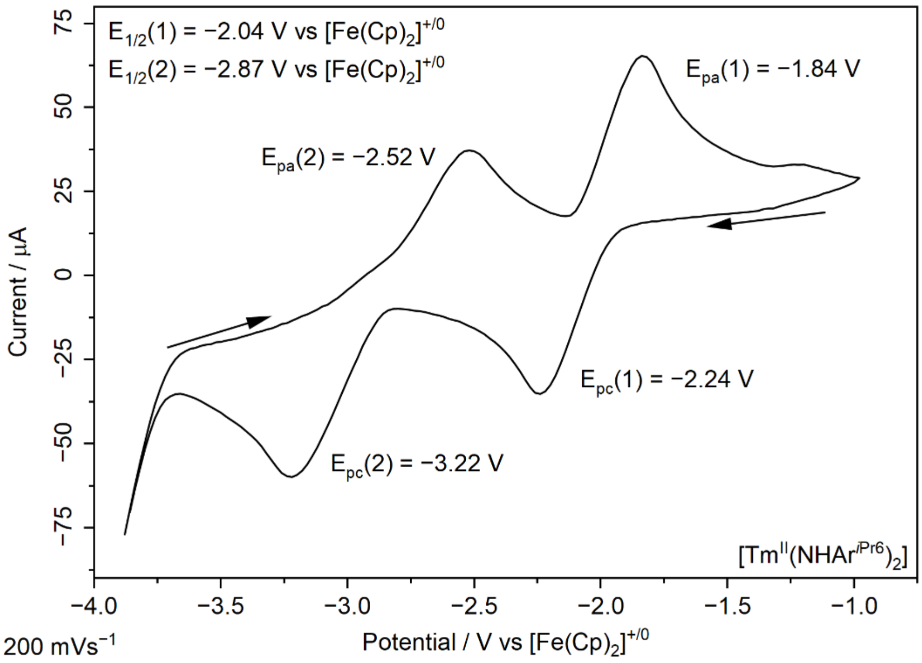

Figure S87. Cyclic voltammogram of **1Tm** in THF (10 mM) supported by [*n*Bu_4N][BPh_4] (50 mM) vs $[Fc]^{+/0}$ couple at 200 mV s^{-1}.

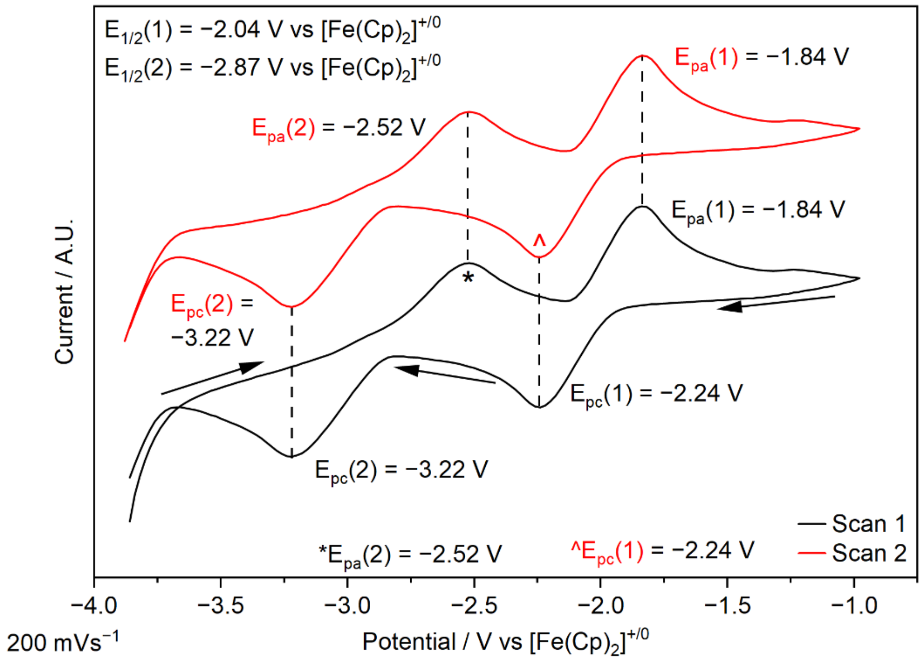

Figure S88. Cyclic voltammogram of **1Tm** in THF (10 mM) supported by [*n*Bu_4N][BPh_4] (50 mM) vs $[Fc]^{+/0}$ couple at 200 mV s^{-1} with repeat scans to depict no electrochemical changes.

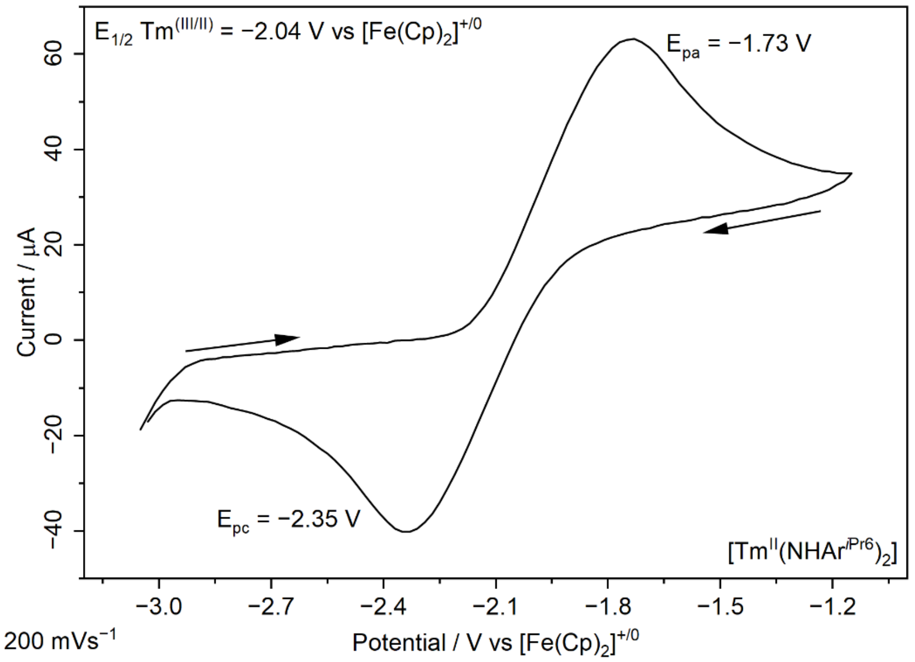

Figure S89. Cyclic voltammogram of **1Tm** in THF (10 mM) supported by [nBu$_4$N][BPh$_4$] (50 mM) vs [Fc]$^{+/0}$ couple at 200 mV s^{-1} of the [**1Tm**]$^{+/0}$ couple.

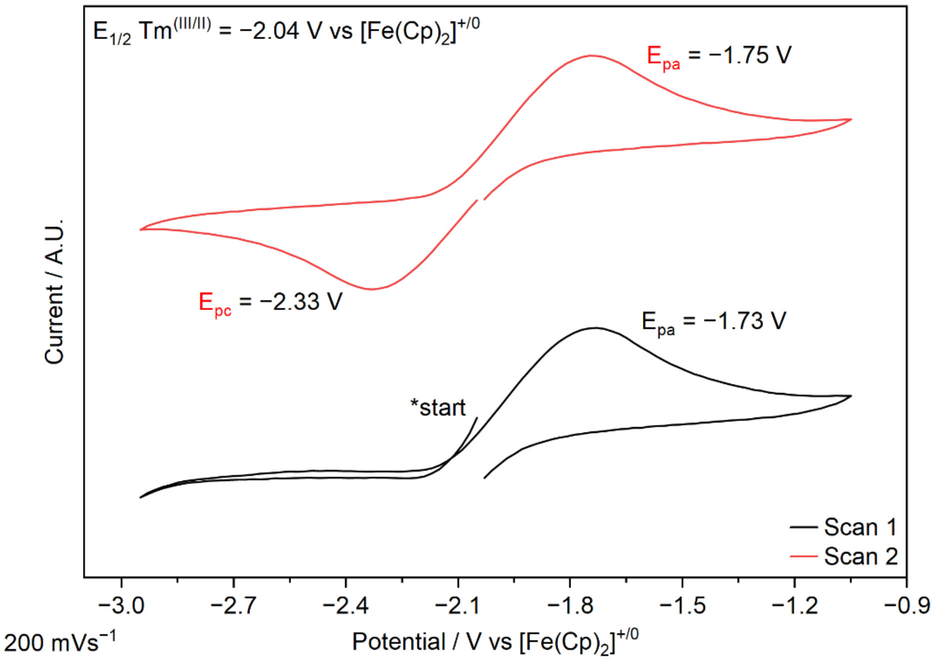

Figure S90. Cyclic voltammogram of **1Tm** in THF supported by [nBu$_4$N][BPh$_4$] (50 mM) vs [Fc]$^{+/0}$ couple at 200 mV s^{-1}. Scans indicate this is the [**1Tm**]$^{+/0}$ couple as Scan 1 shows no reduction event until the analyte is first oxidised.

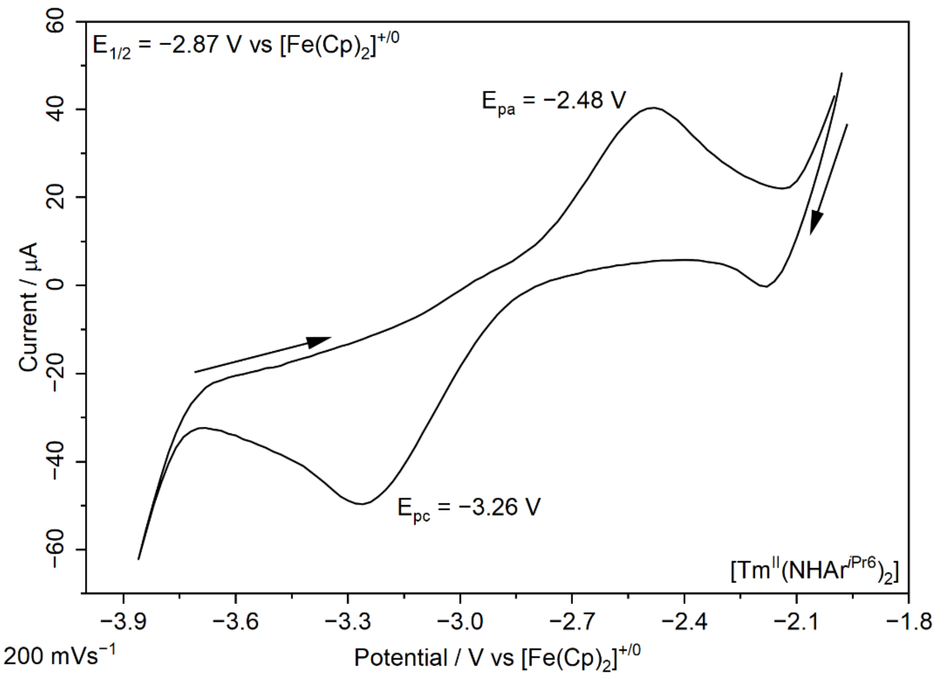

Figure S91. Cyclic voltammogram of **1Tm** in THF (10 mM) supported by [*n*Bu_4N][BPh_4] (50 mM) vs $[Fc]^{+/0}$ couple at 200 mV s^{-1} of the second redox event.

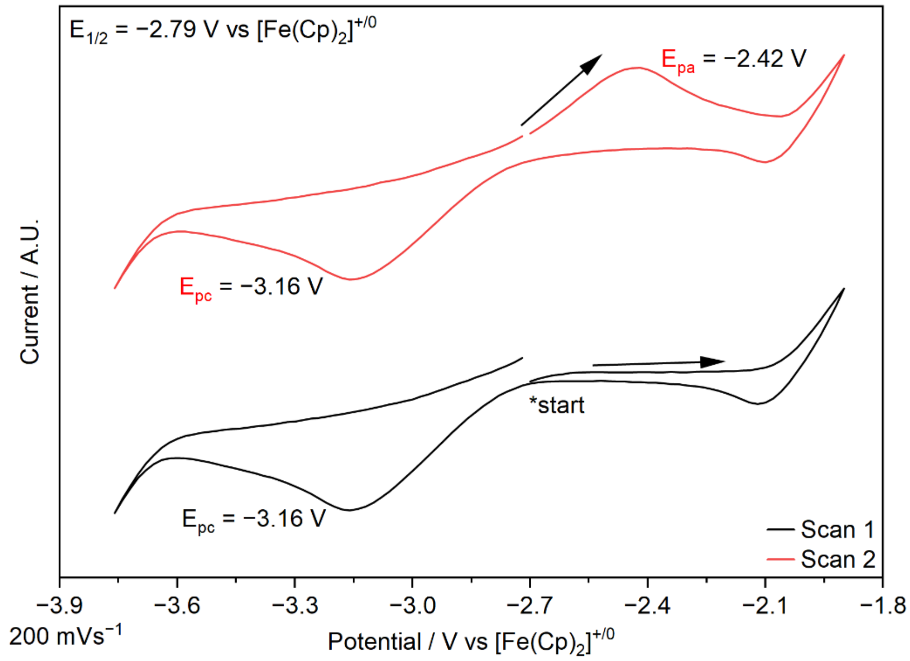

Figure S92. Cyclic voltammogram of **1Tm** in THF (10 mM) supported by [*n*Bu_4N][BPh_4] (50 mM) vs $[Fc]^{+/0}$ couple at 200 mV s^{-1}. Scans indicate that the second redox event is a reduction derived event as Scan 1 shows no oxidation event until the analyte is first reduced.

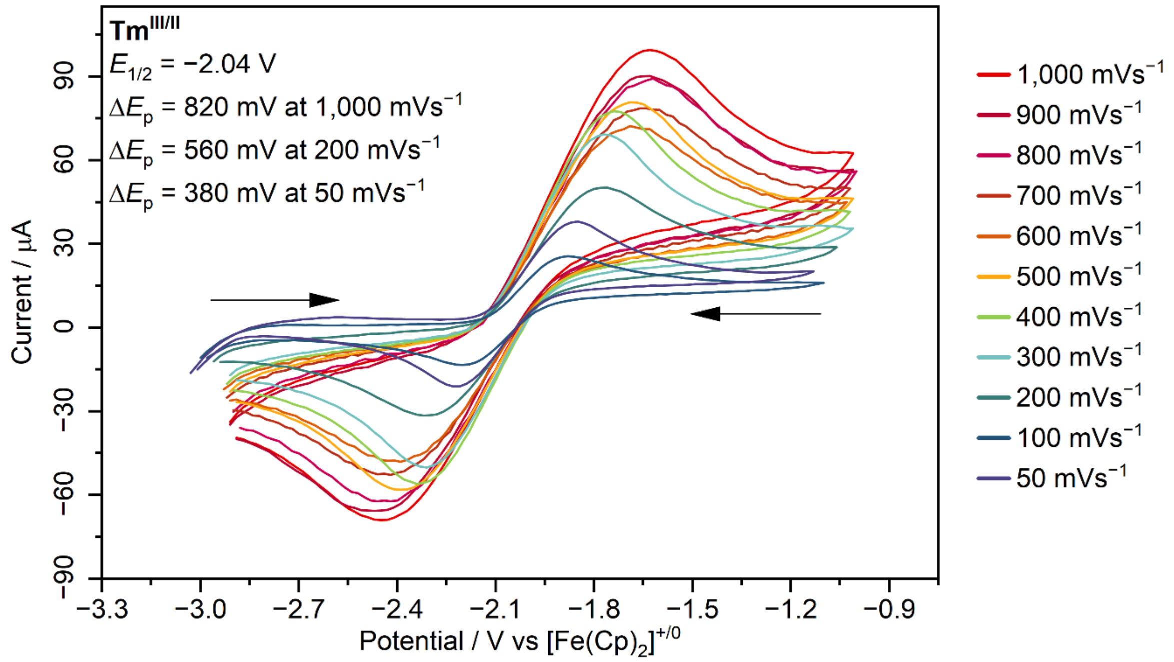

Figure S93. Scan-rate dependence of cyclic voltammogram of **1Tm** in THF (10 mm) supported by [nBu$_4$N][BPh$_4$] (50 mM) vs $[Fc]^{+/0}$ couple of the $\mathbf{1Tm}^{+/0}$ couple.

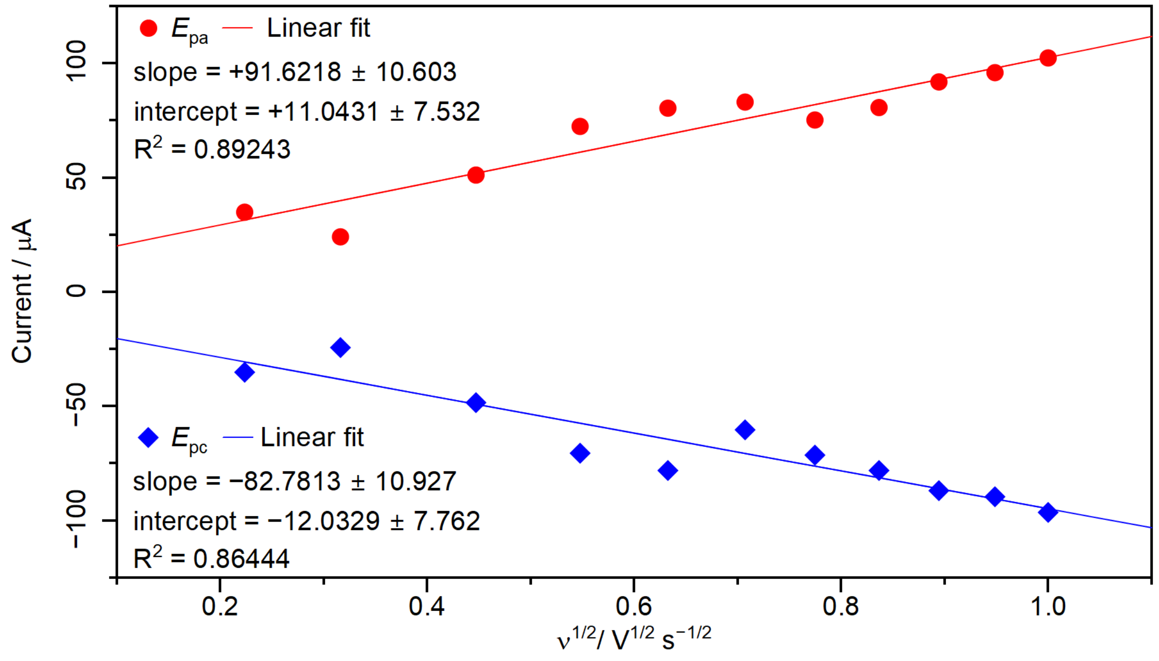

Figure S94. Randles-Sevcik plot of the $[\mathbf{1Tm}]^{+/0}$ couple of **1Tm**.

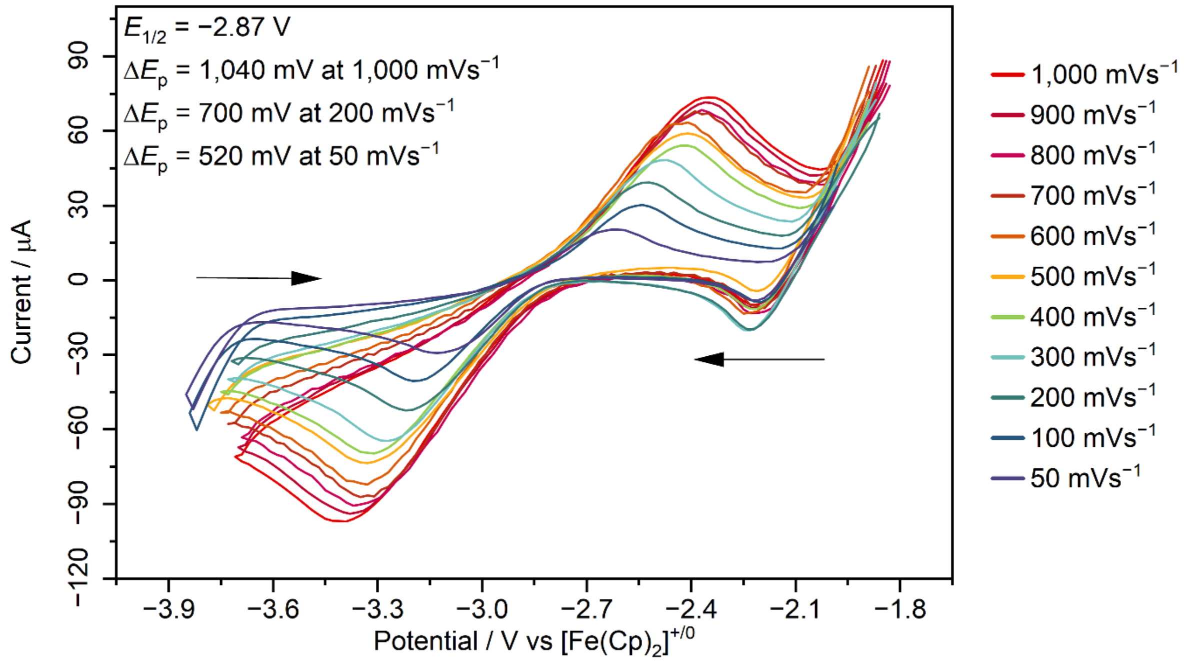

Figure S95. Scan-rate dependence of cyclic voltammogram of **1Tm** in THF (10 mM) supported by [nBu$_4$N][BPh$_4$] (50 mM) vs [Fc]$^{+/0}$ couple of the second redox event.

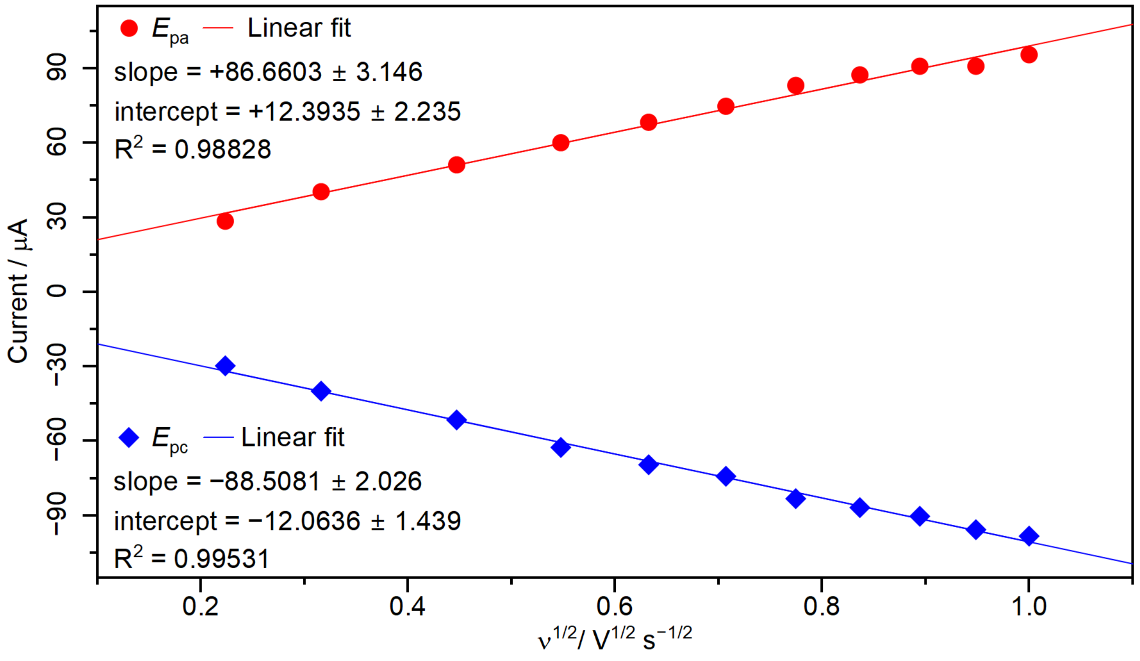

Figure S96. Randles-Sevcik plot of the second redox event of **1Tm**.

***1Sm** – [Sm^{II}(NHAr^{iPr6})$_2$]*

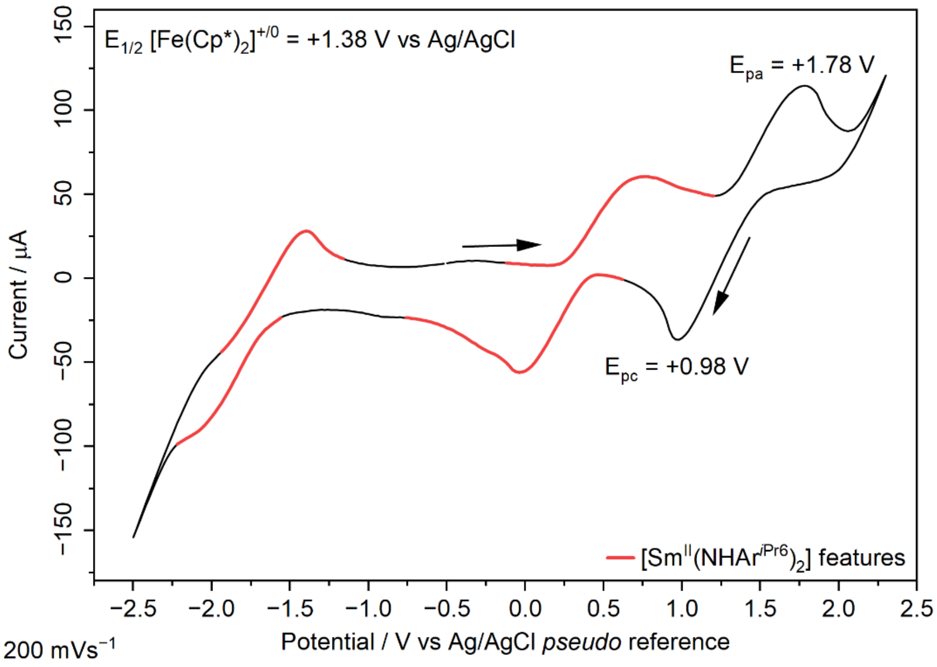

Figure S97. Cyclic voltammogram of **1Sm** with Fc* internal standard in THF (10 mM) supported by [*n*Bu$_4$N][BPh$_4$] (50 mM) vs Ag/AgCl pseudo reference at 200 mV s^{-1}.

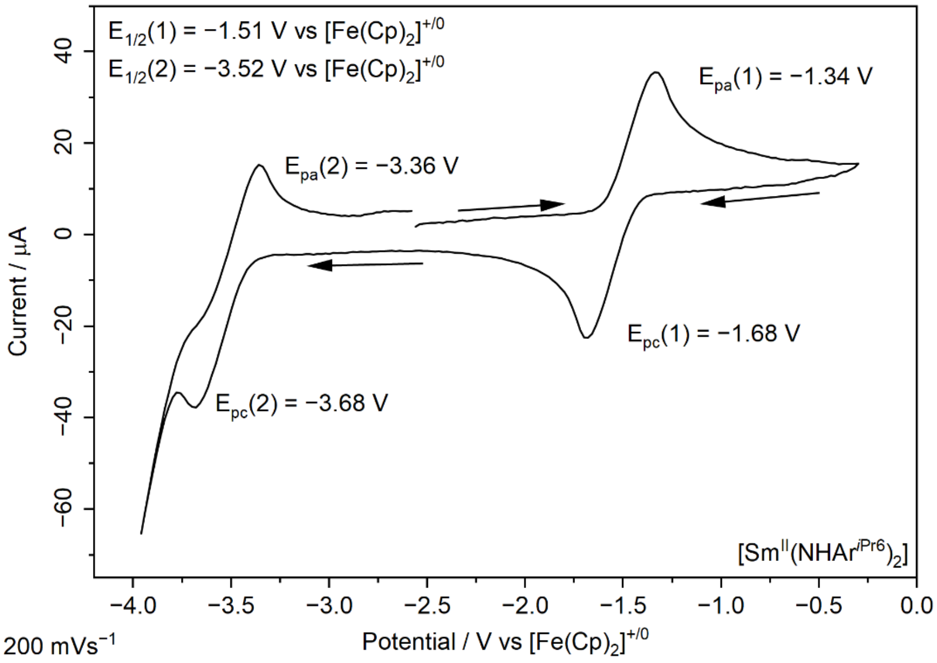

Figure S98. Cyclic voltammogram of **1Sm** in THF (10 mM) supported by [*n*Bu$_4$N][BPh$_4$] (50 mM) vs [Fc]$^{+/0}$ couple at 200 mV s^{-1}.

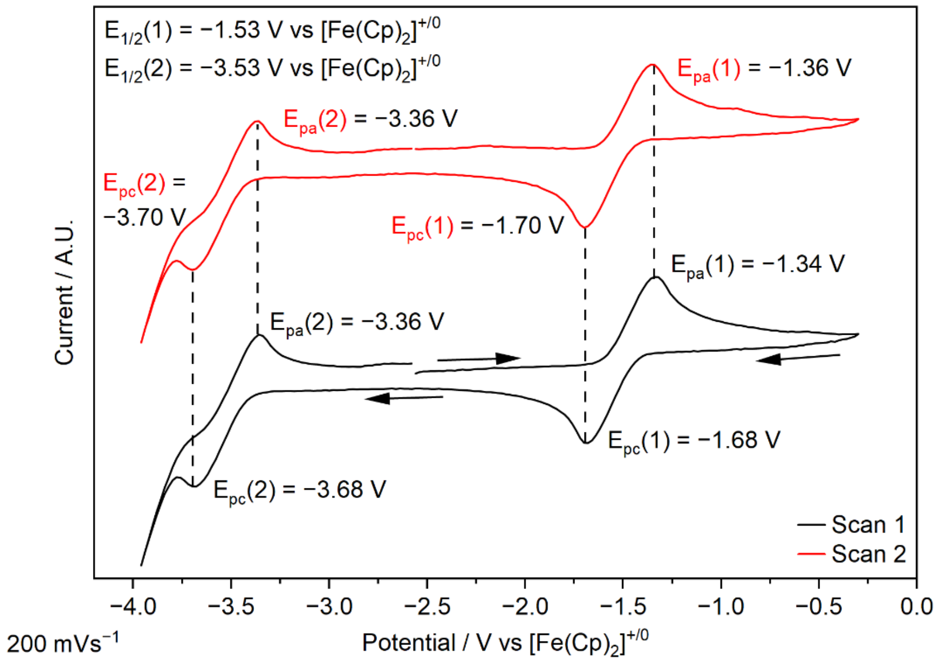

Figure S99. Cyclic voltammogram of **1Sm** in THF (10 mM) supported by [*n*Bu4N][BPh4] (50 mM) vs $[Fc]^{+/0}$ couple at 200 mV s^{-1} with repeat scans to depict no electrochemical changes.

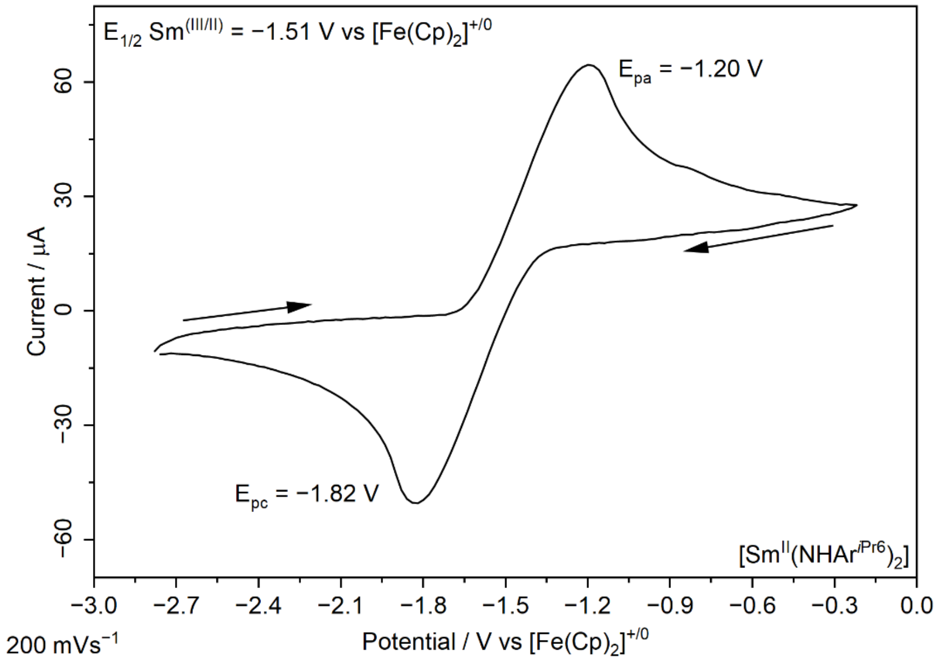

Figure S100. Cyclic voltammogram of **1Sm** in THF (10 mM) supported by [*n*Bu4N][BPh4] (50 mM) vs $[Fc]^{+/0}$ couple at 200 mV s^{-1} of the $[\mathbf{1Sm}]^{+/0}$ couple.

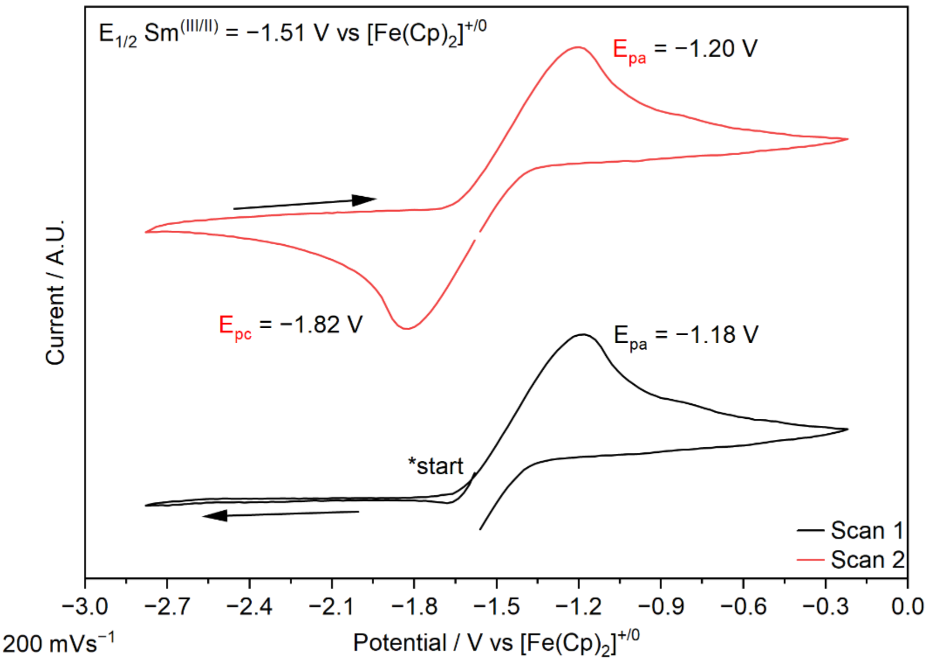

Figure S101. Cyclic voltammogram of **1Sm** in THF (10 mM) supported by [*n*Bu4N][BPh4] (50 mM) vs $[Fc]^{+/0}$ couple at 200 mV s^{-1}. Scans indicate this is the $[\mathbf{1Sm}]^{+/0}$ couple as Scan 1 shows no reduction event until the analyte is first oxidised.

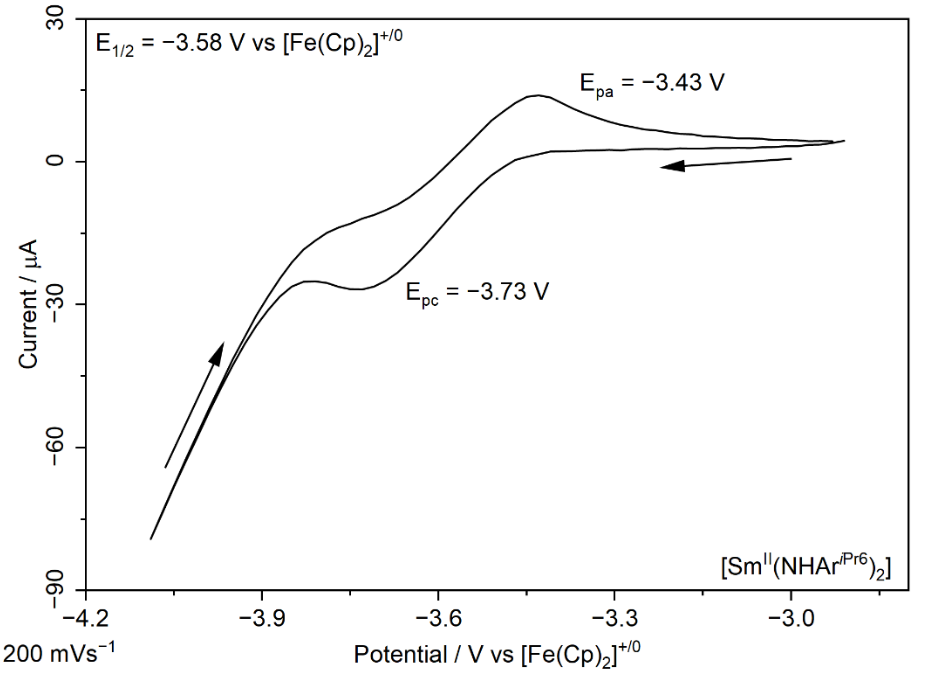

Figure S102. Cyclic voltammogram of **1Sm** in THF (10 mM) supported by [*n*Bu4N][BPh4] (50 mM) vs $[Fc]^{+/0}$ couple at 200 mV s^{-1} of the second redox event.

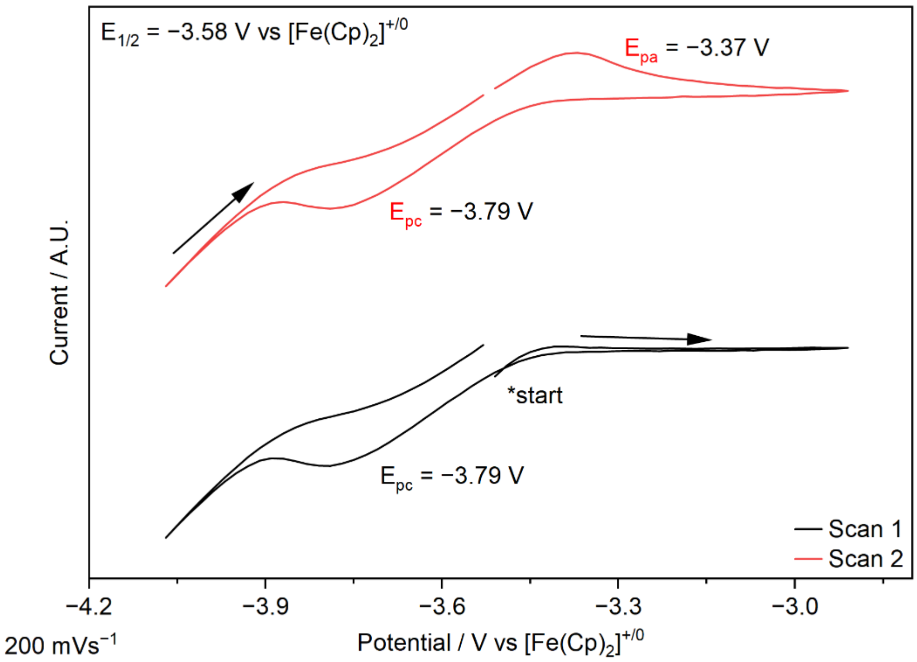

Figure S103. Cyclic voltammogram of **1Sm** in THF (10 mM) supported by [*n*Bu4N][BPh4] (50 mM) vs $[Fc]^{+/0}$ couple at 200 mV s^{-1}. Scans indicate that the second redox event is a reduction derived event as Scan 1 shows no oxidation event until the analyte is first reduced.

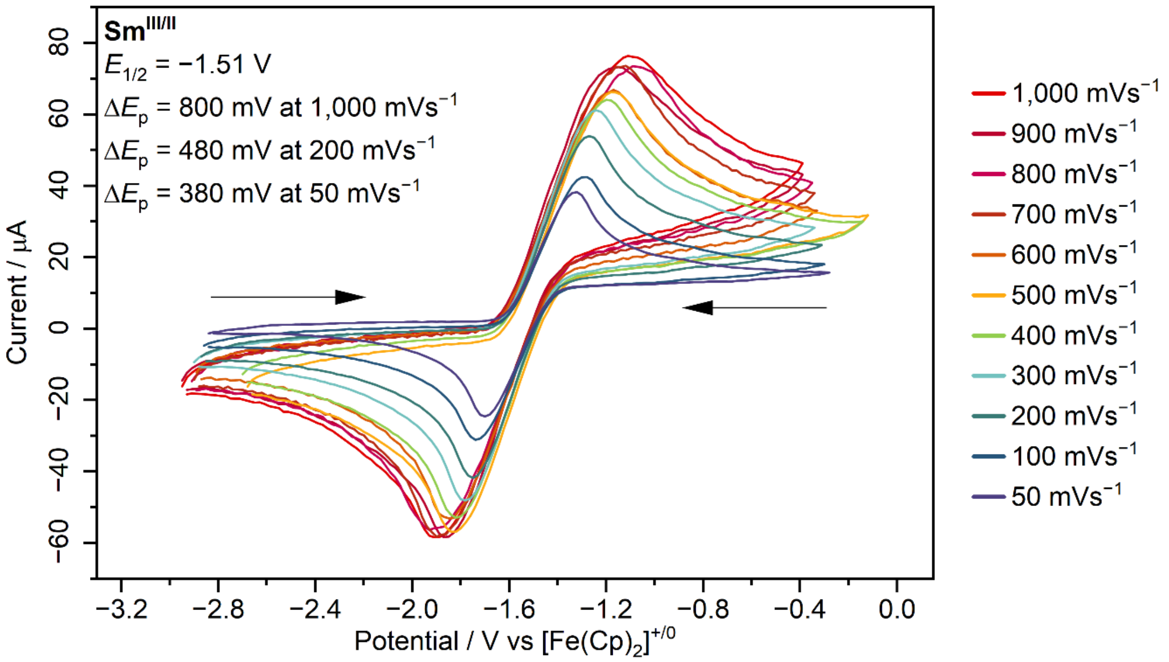

Figure S104. Scan-rate dependence of cyclic voltammogram of **1Sm** in THF (10 mM) supported by [*n*Bu4N][BPh4] (50 mM) vs $Fc^{+/0}$ couple of the $\mathbf{1Sm}^{+/0}$ couple.

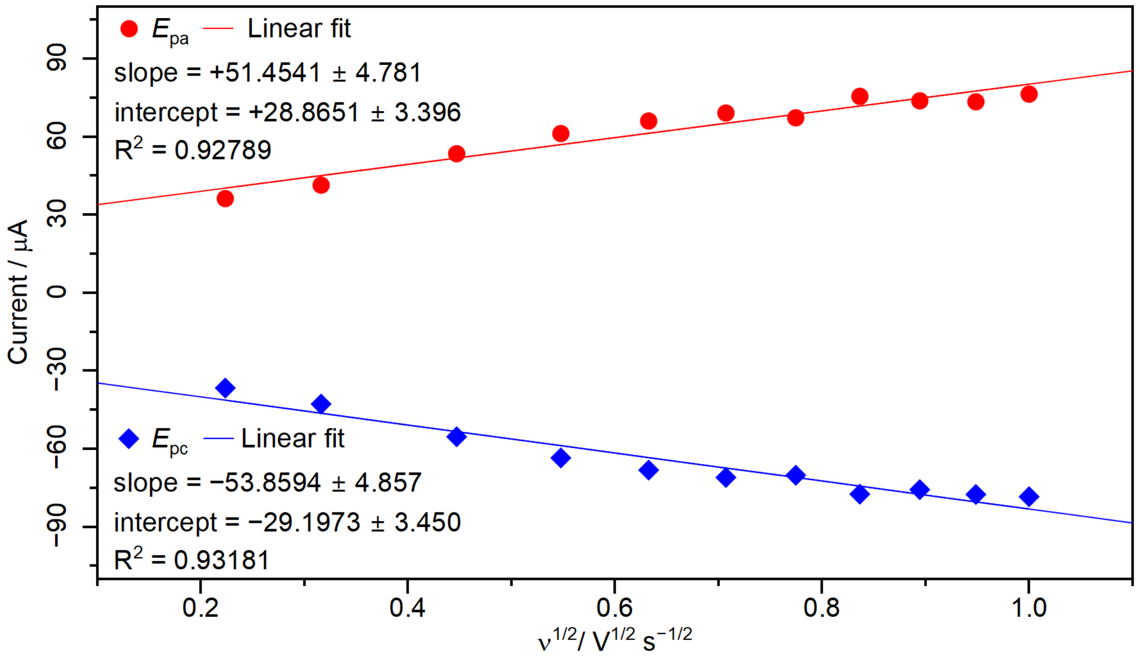

Figure S105. Randles-Sevcik plot of the $[\mathbf{1Sm}]^{+/0}$ couple of **1Sm**.

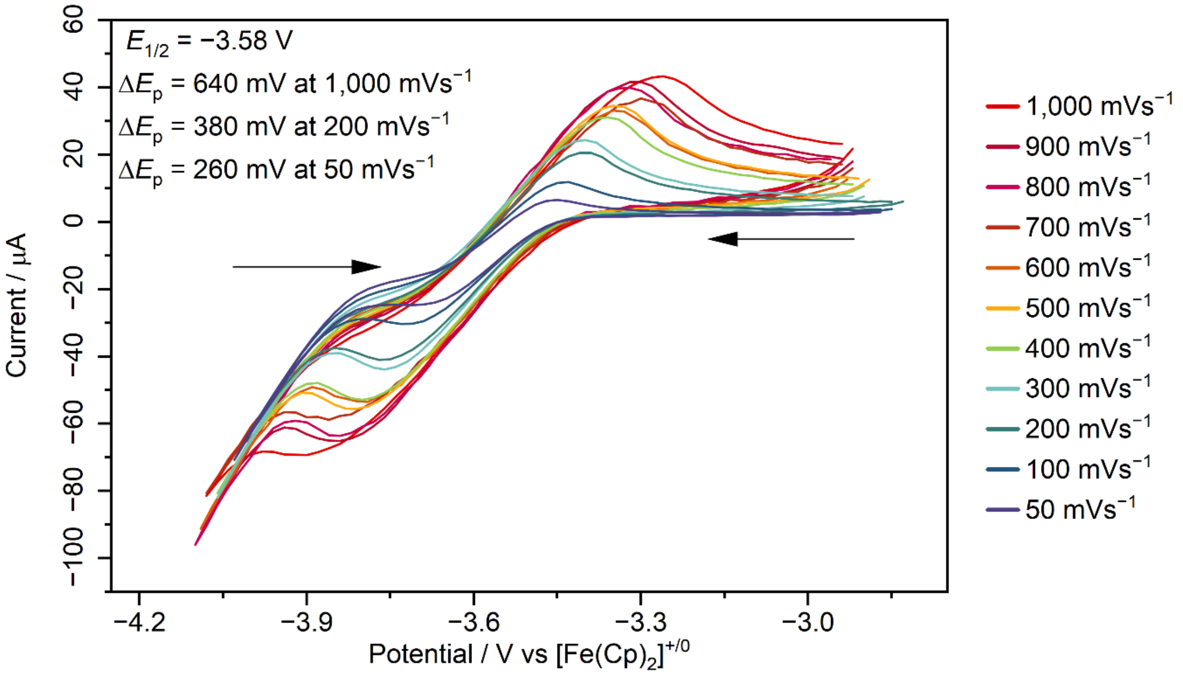

Figure S106. Scan-rate dependence of cyclic voltammogram of **1Sm** in THF (10 mM) supported by [nBu$_4$N][BPh$_4$] (50 mM) vs $[Fc]^{+/0}$ couple of the second redox event.

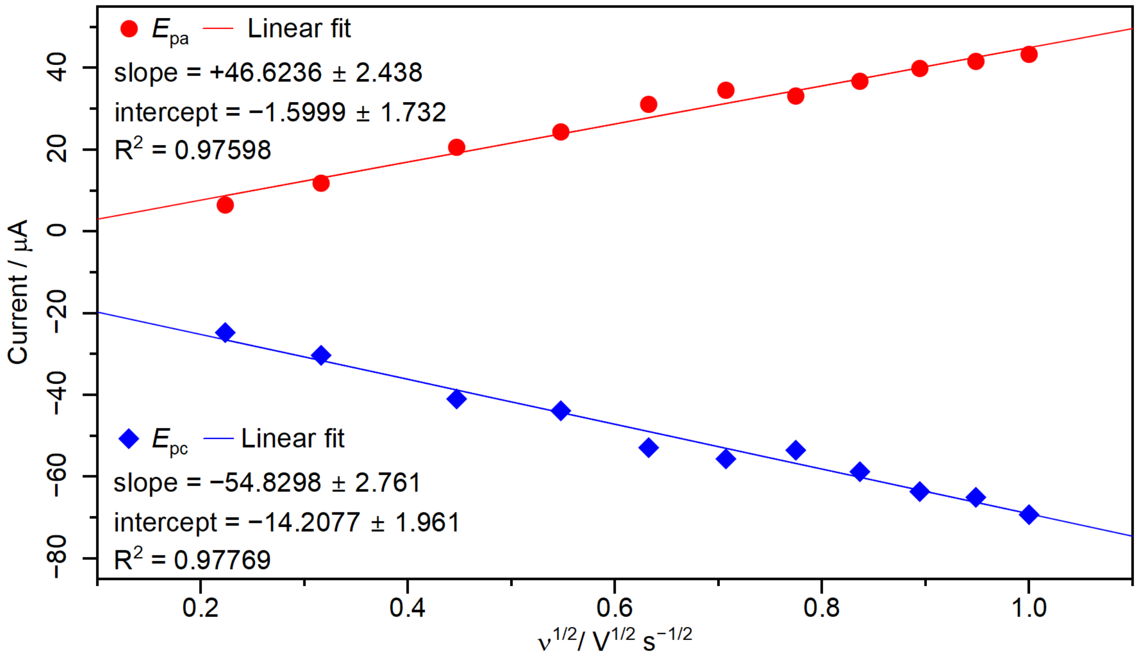

Figure S107. Randles-Sevcik plot of the second redox event of **1Sm**.

***1Yb** – [Yb^{II}(NHAr^{iPr6})$_2$]*

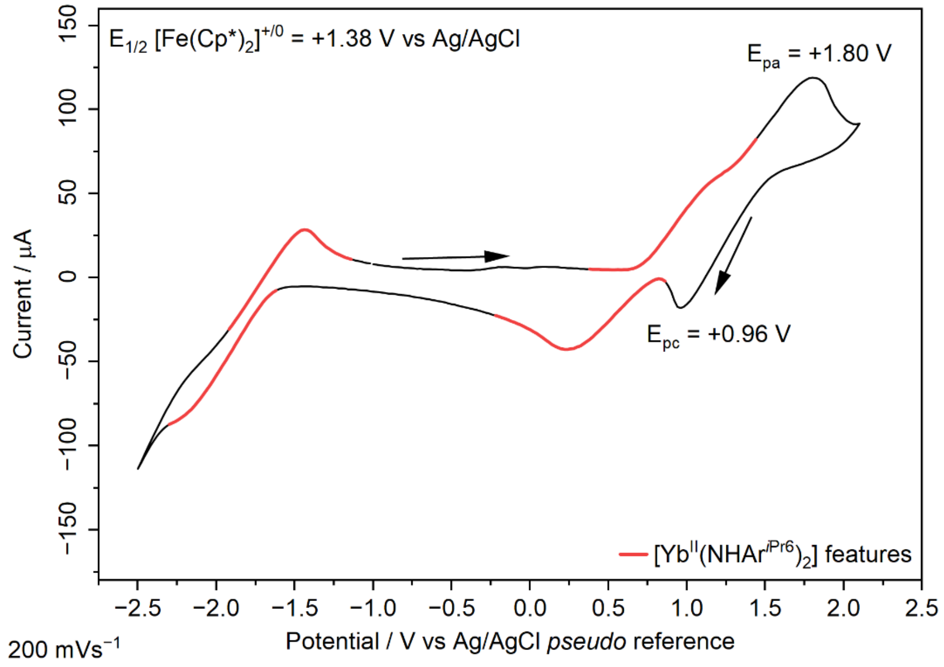

Figure S108. Cyclic voltammogram of **1Yb** with Fc* internal standard in THF (10 mM) supported by [nBu$_4$N][BPh$_4$] (50 mM) vs Ag/AgCl pseudo reference at 200 mV s^{-1}.

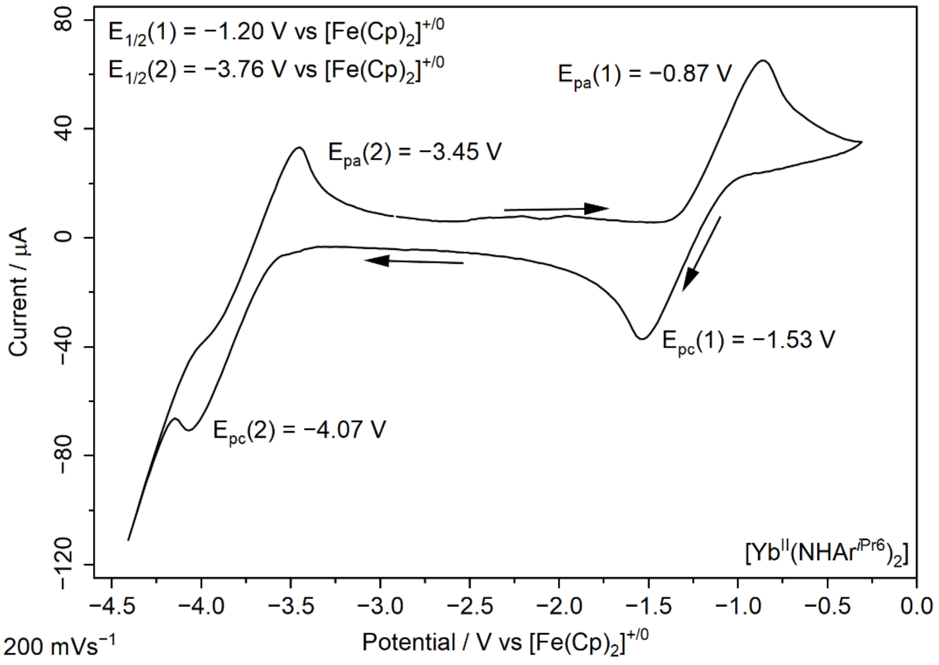

Figure S109. Cyclic voltammogram of **1Yb** in THF (10 mM) supported by [*n*Bu_4N][BPh_4] (50 mM) vs $[Fc]^{+/0}$ couple at 200 mV s^{-1}.

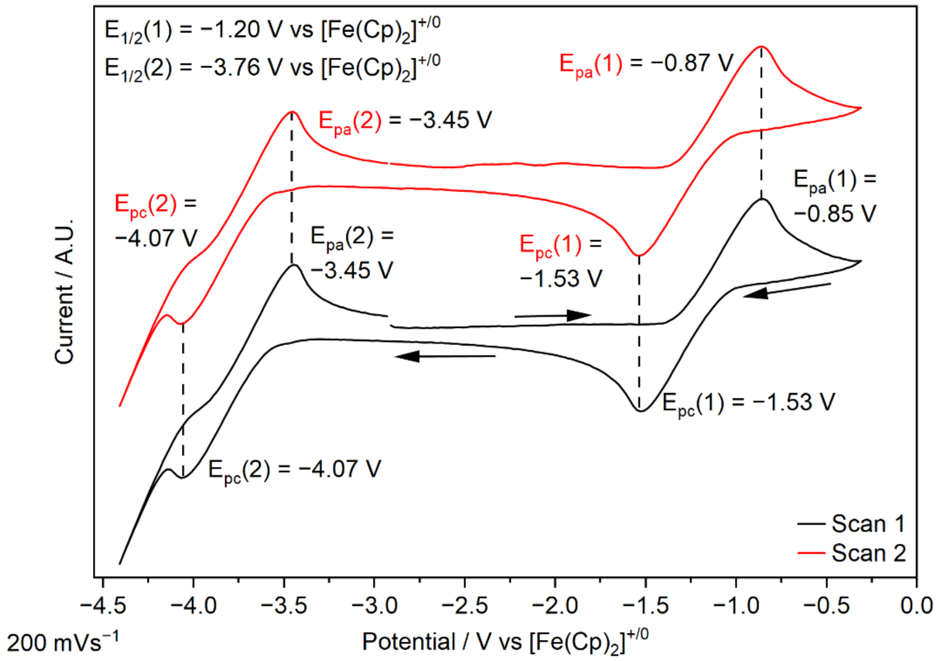

Figure S110. Cyclic voltammogram of **1Yb** in THF (10 mM) supported by [*n*Bu_4N][BPh_4] (50 mM) vs $[Fc]^{+/0}$ couple at 200 mV s^{-1} with repeat scans to depict no electrochemical changes.

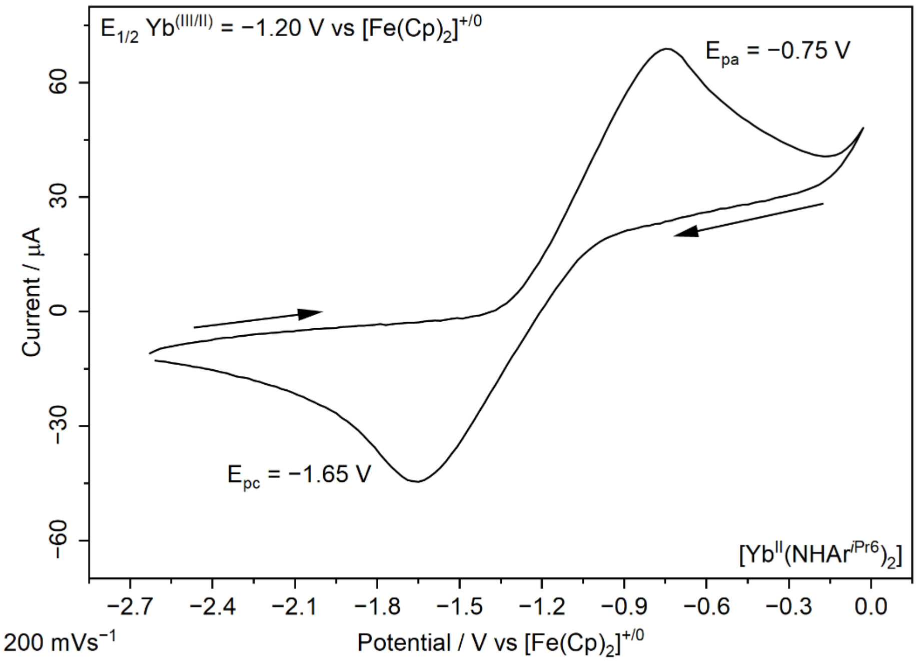

Figure S111. Cyclic voltammogram of **1Yb** in THF (10 mM) supported by [*n*Bu4N][BPh4] (50 mM) vs $[Fc]^{+/0}$ couple at 200 mV s^{-1} of the $[\mathbf{1Yb}]^{+/0}$ couple.

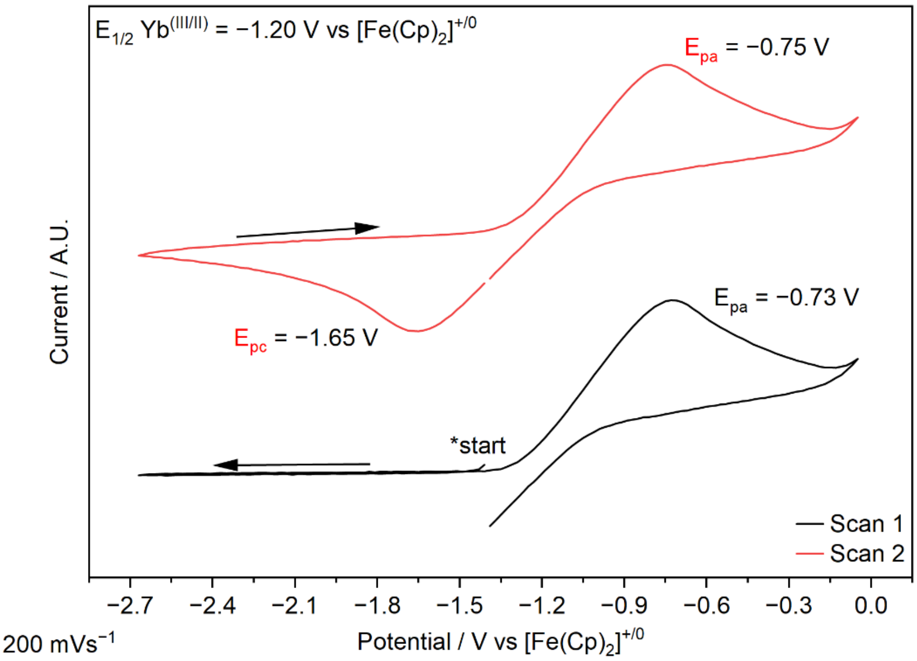

Figure S112. Cyclic voltammogram of **1Yb** in THF (10 mM) supported by [*n*Bu4N][BPh4] (50 mM) vs $[Fc]^{+/0}$ couple at 200 mV s^{-1}. Scans indicate this is the $[\mathbf{1Yb}]^{+/0}$ couple as Scan 1 shows no reduction event until the analyte is first oxidised.

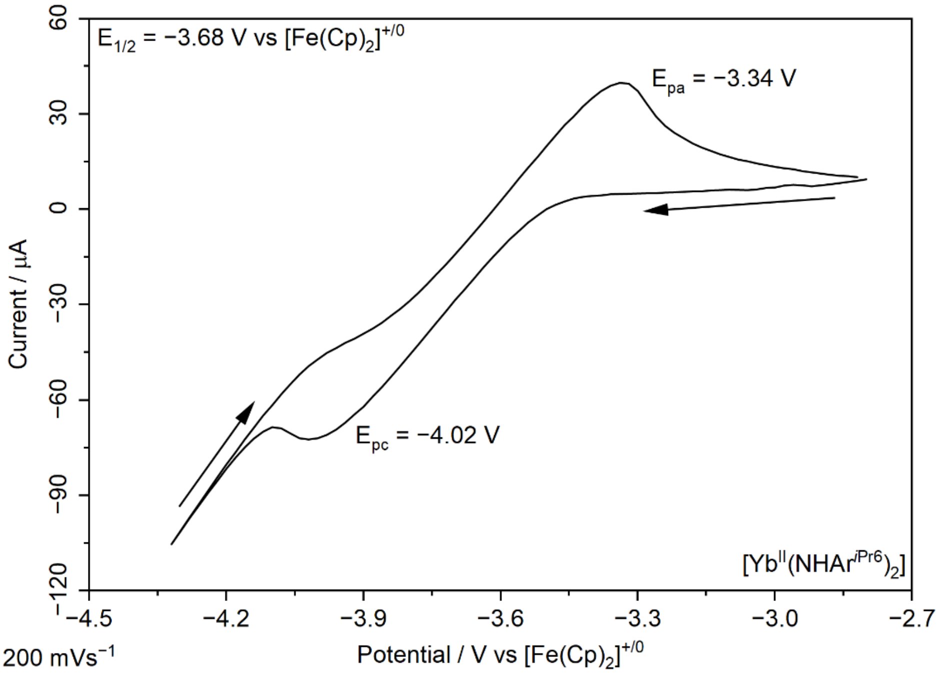

Figure S113. Cyclic voltammogram of **1Yb** in THF (10 mM) supported by [*n*Bu4N][BPh4] (50 mM) vs $[Fc]^{+/0}$ couple at 200 mV s^{-1} of the second redox event.

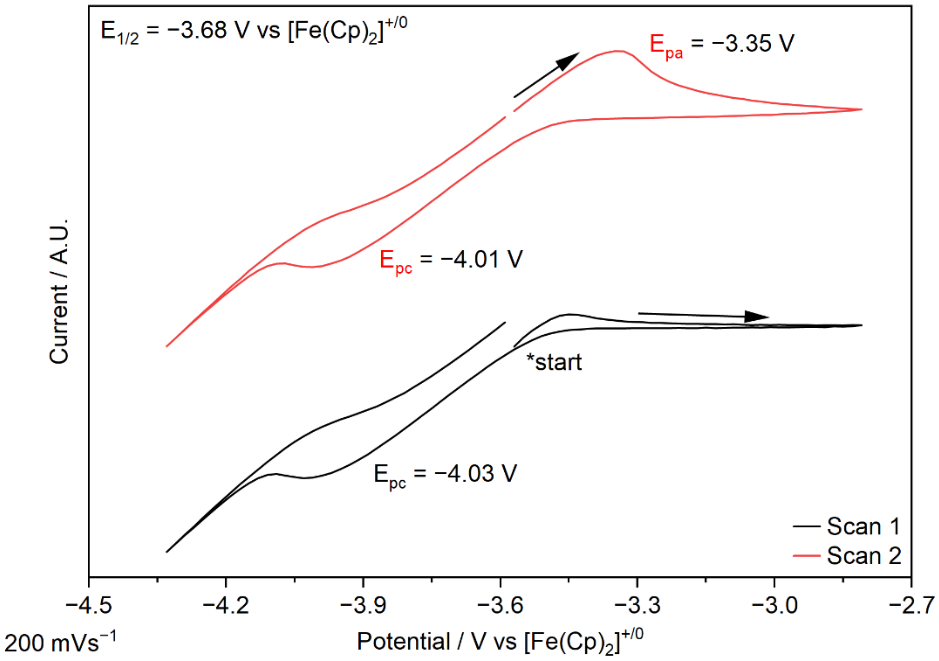

Figure S114. Cyclic voltammogram of **1Yb** in THF (10 mM) supported by [*n*Bu4N][BPh4] (50 mM) vs $[Fc]^{+/0}$ couple at 200 mV s^{-1}. Scans indicate that the second redox event is a reduction derived event as Scan 1 shows no oxidation event until the analyte is first reduced.

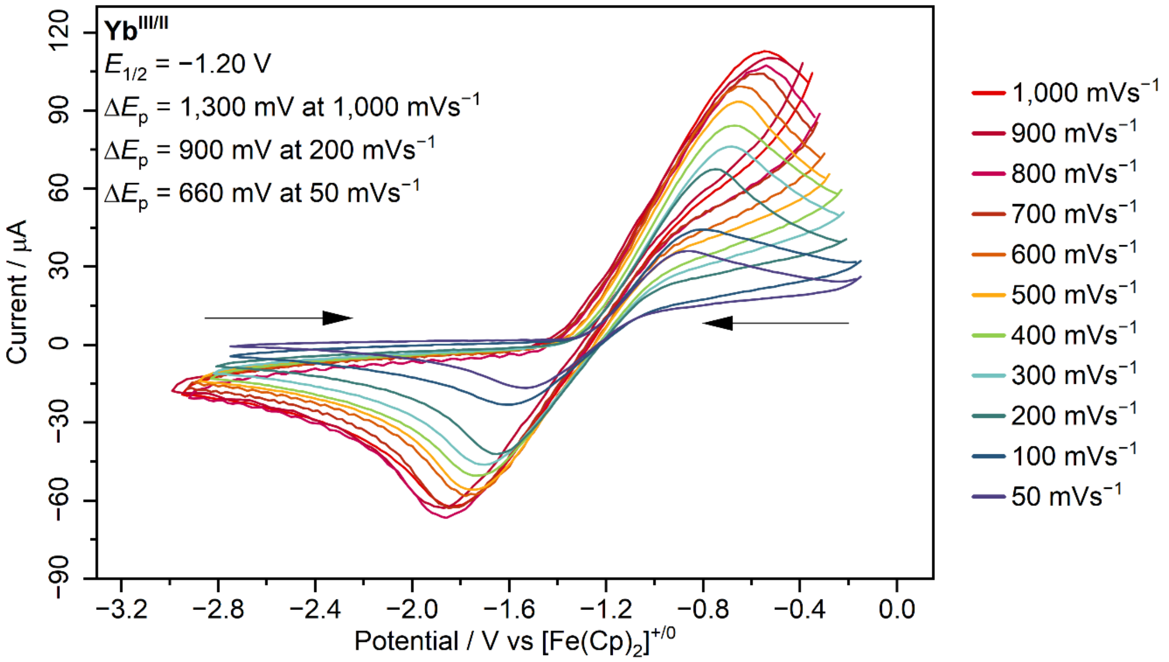

Figure S115. Scan-rate dependence of cyclic voltammogram of **1Yb** in THF (10 mM) supported by [*n*Bu_4N][BPh_4] (50 mM) vs $[Fc]^{+/0}$ couple of the $[\mathbf{1Yb}]^{+/0}$ couple.

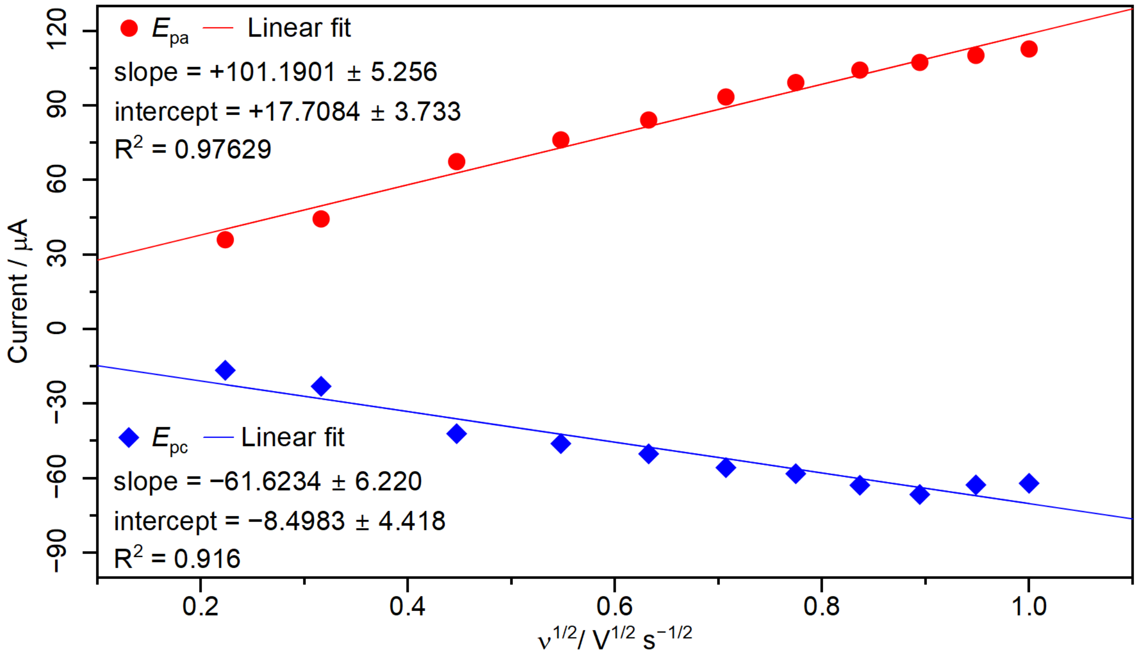

Figure S116. Randles-Sevcik plot of the $\mathbf{1Yb}^{+/0}$ couple of **1Yb**.

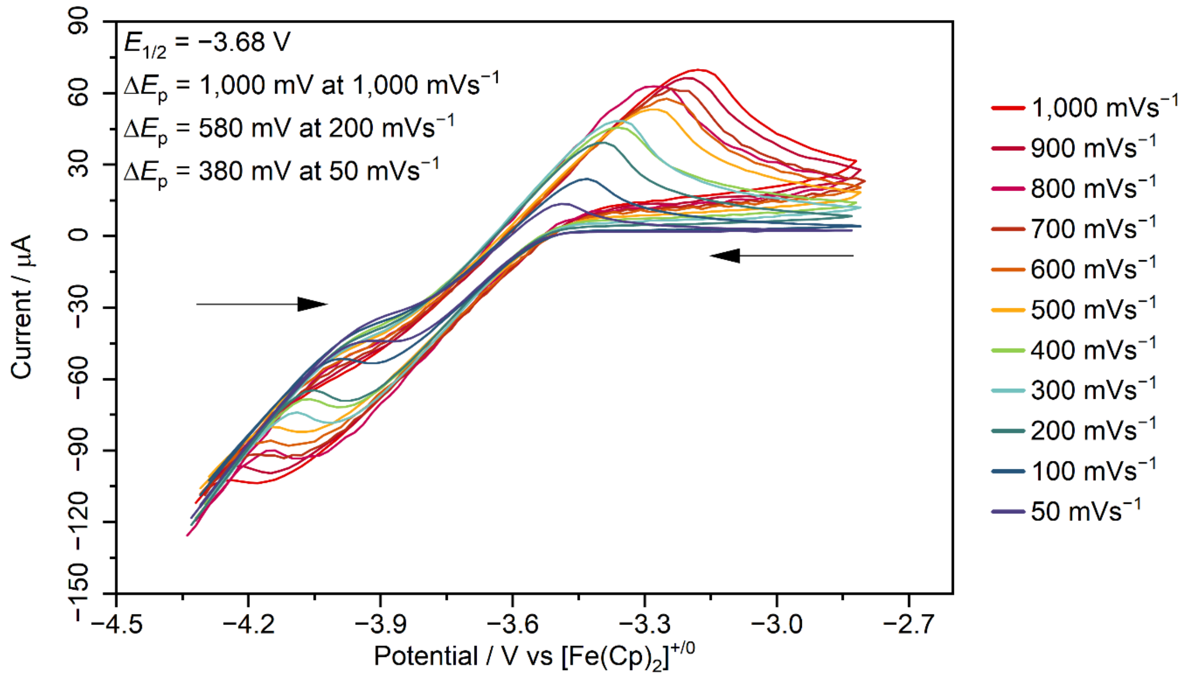

Figure S117. Scan-rate dependence of cyclic voltammogram of **1Yb** in THF (10 mM) supported by [nBu$_4$N][BPh$_4$] (50 mM) vs [Fc]$^{+/0}$ couple of the second redox event.

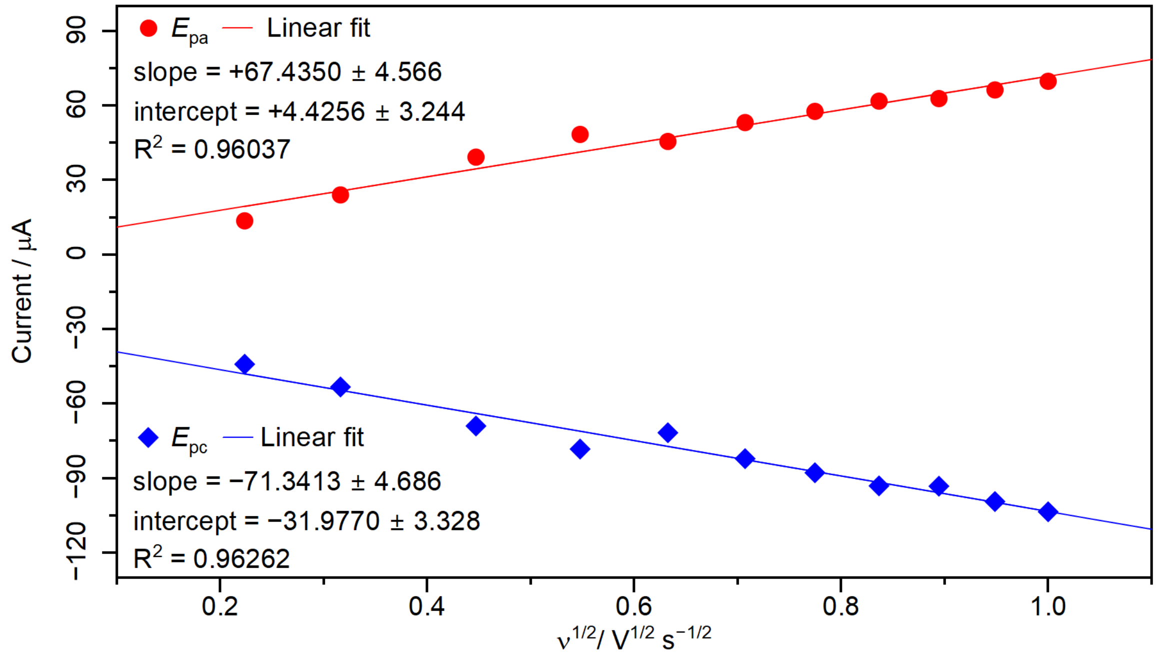

Figure S118. Randles-Sevcik plot of the second redox event of **1Yb**.

1Eu – [$Eu^{II}(NHAr^{iPr6})_2$]

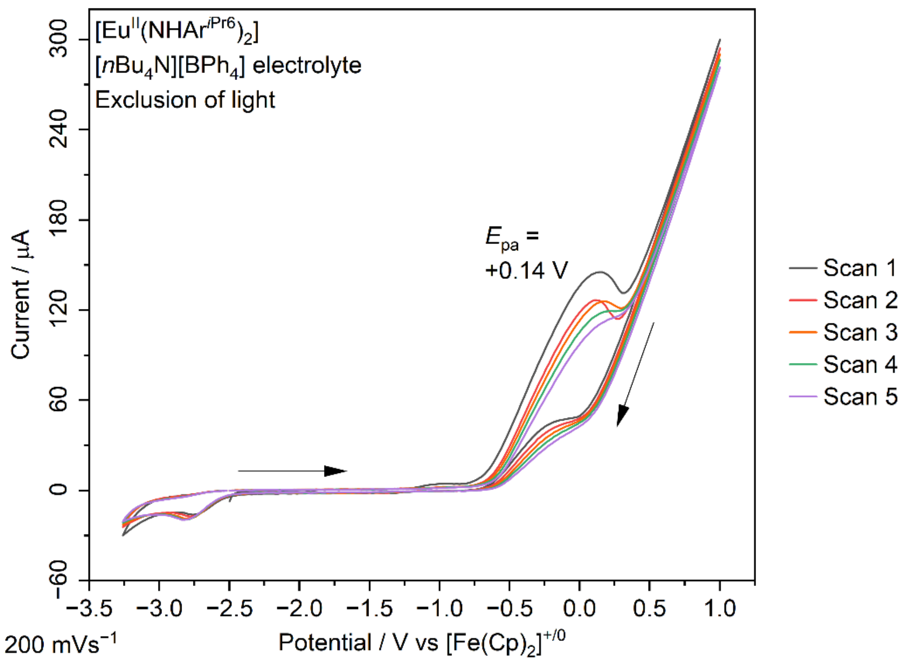

Figure S119. Cyclic voltammogram of **1Eu** in THF (10 mM) supported by [nBu$_4$N][BPh$_4$] (50 mM) vs [Fc]$^{+/0}$ couple at 200 mV s^{-1}, measured under the exclusion of light. Multiple scans recorded showing a progressive decrease in current density for the process shown. No reductive event was observed.

[$K(NHAr^{iPr6})_2$]

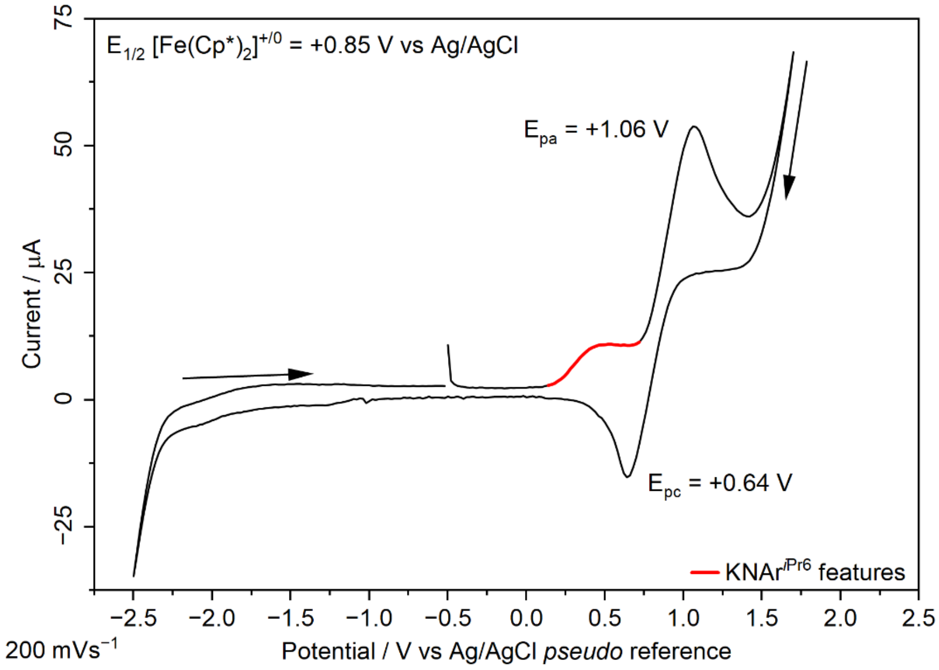

Figure S120. Cyclic voltammogram of KNHAr^{iPr6} (10 mM) with Fc* internal standard in THF supported by [nBu$_4$N][BPh$_4$] (50 mM) vs Ag/AgCl pseudo reference at 200 mV s^{-1}.

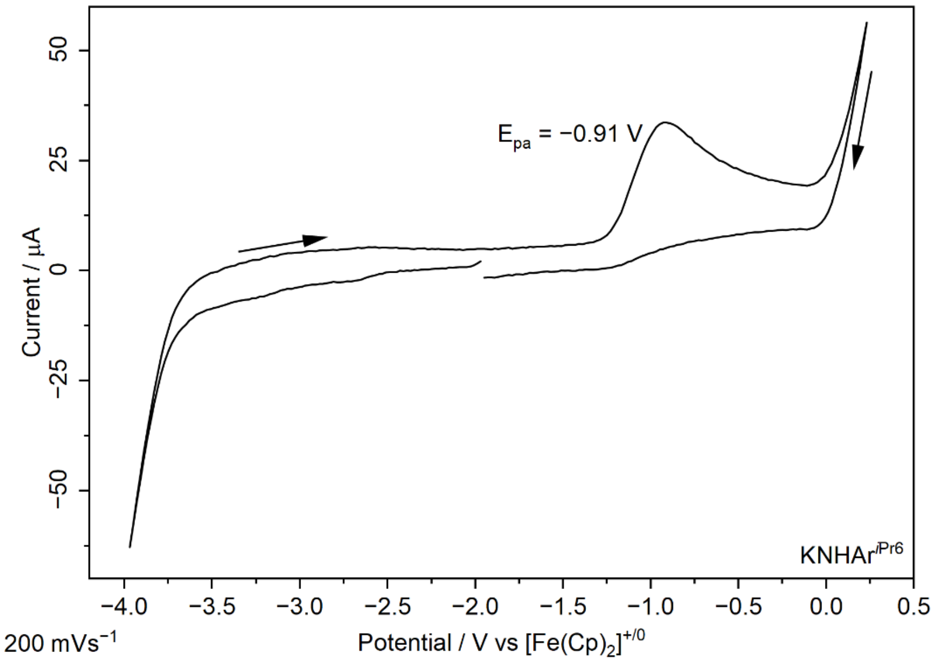

Figure S121. Cyclic voltammogram of KNHAr^{iPr6} (10 mM) in THF supported by [nBu$_4$N][BPh$_4$] (50 mM) vs [Fc]$^{+/0}$ couple at 200 mV s^{-1}.

Cyclic voltammetry of pyridine

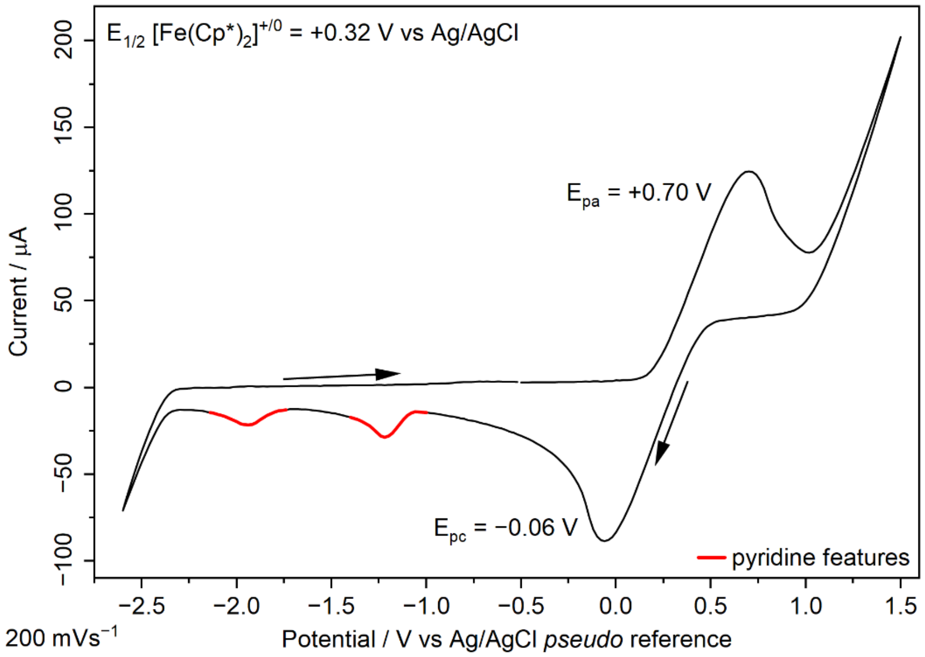

Figure S122. Cyclic voltammogram of pyridine (2.0 M) with Fc* internal standard in THF supported by [nBu$_4$N][BPh$_4$] (50 mM) vs Ag/AgCl pseudo reference at 200 mV s^{-1}.

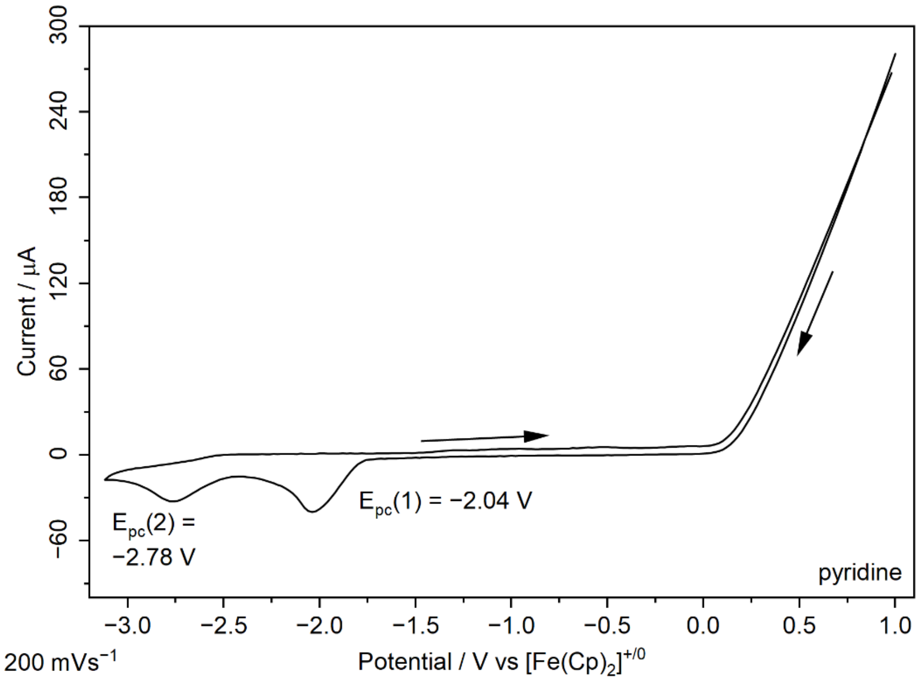

Figure S123. Cyclic voltammogram of pyridine (2.0 M) in THF supported by [nBu$_4$N][BPh$_4$] (50 mM) vs [Fc]$^{+/0}$ couple at 200 mV s^{-1}.

Cyclic voltammetry of 4,4'-bipyridine

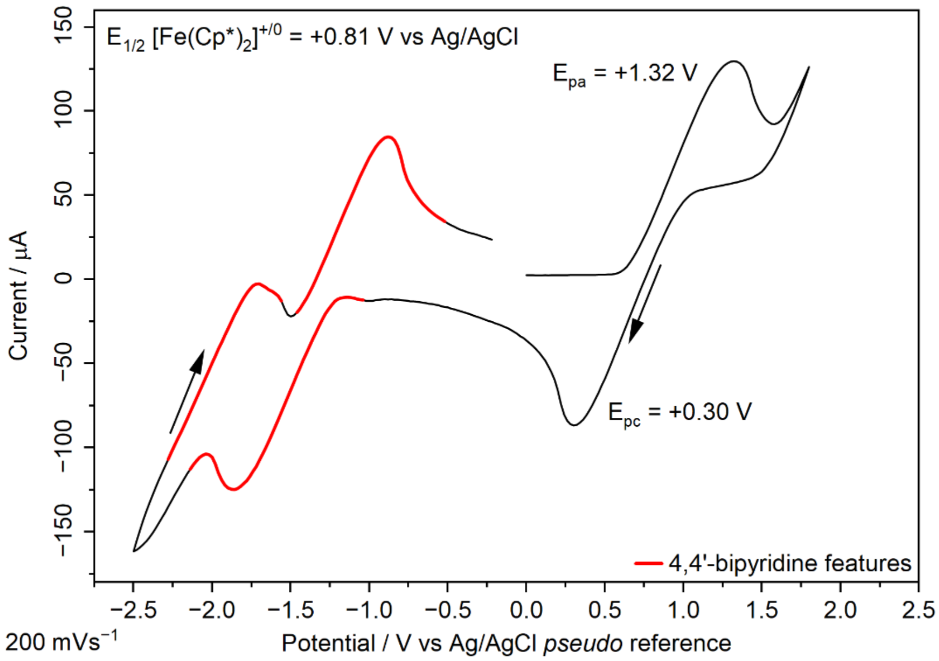

Figure S124. Cyclic voltammogram of 4,4'-bipyridine (10 mM) with Fc* internal standard in THF supported by [nBu$_4$N][BPh$_4$] (50 mM) vs Ag/AgCl pseudo reference at 200 mV s^{-1}. Note that the second reduction event could not be observed.

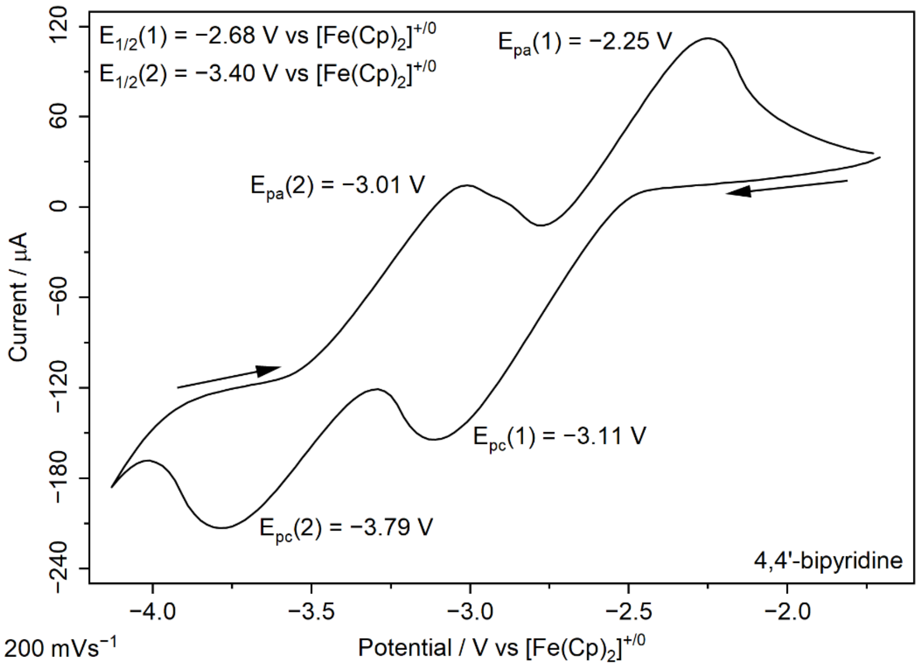

Figure S125. Cyclic voltammogram of 4,4'-bipyridine (10 mM) in THF supported by [nBu$_4$N][BPh$_4$] (50 mM) vs [Fc]$^{+/0}$ couple at 200 mV s^{-1}.

***2Y** – [{Y^{III}(NHAr^{iPr6})$_2$}$_2$(μ-N$_2$C$_{10}$H$_{10}$)]*

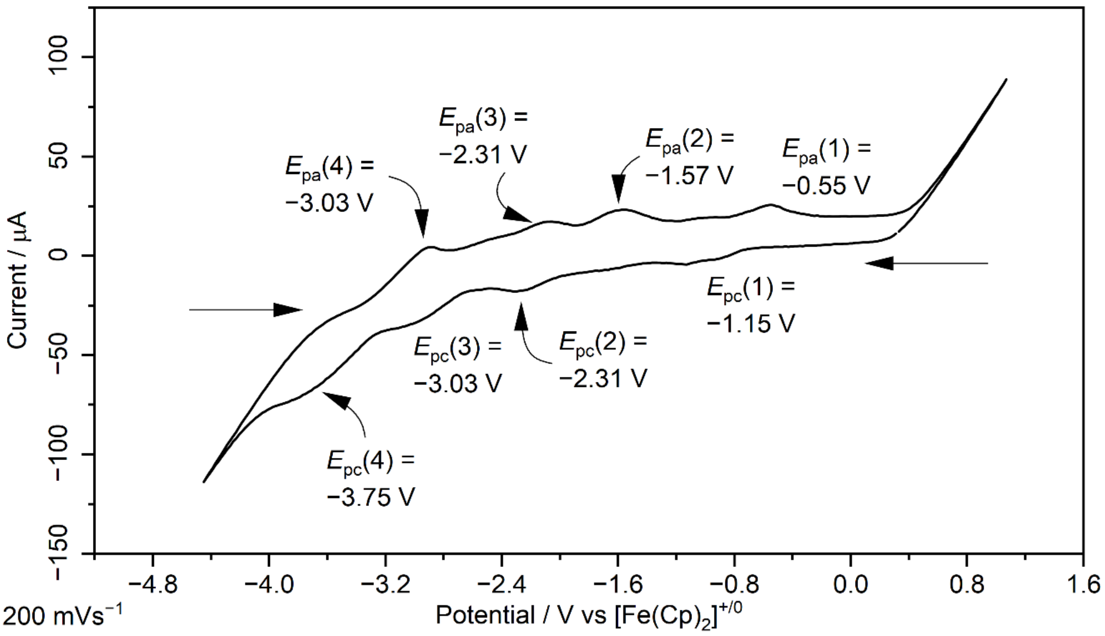

Figure S126. Cyclic voltammogram of **2Y** in THF (2.5 mM) supported by [nBu$_4$N][BPh$_4$] (50 mM) vs Fc]$^{+/0}$ couple at 200 mV s^{-1}. We found several oxidative and reductive processes that appeared independent of each other.

S8. Reactivity study with 1M (M = Sm, Eu, Yb)

NMR spectroscopy

Table S10. Data for the determination of magnetic susceptibilities (where applicable) and quantities of complexes **1Sm**, **1Eu**, and **1Yb** used in NMR studies with 4,4′-bipyridine and pyridine.

Sample	μ_{eff} B.M mol^{-1}	mass of sample g [A]	mass substrate g	mass of solvent g	M_r g mol^{-1}	Δ peak Hz [B]
**1Sm**	3.12	0.0162	–	0.5242	1143.93	163.07
**1Sm** + 4,4′-bipyiridine	3.30	0.0174	0.0009	0.5010		227.27
**1Sm** + pyridine	3.55	0.0103	0.0195	0.5725		102.51
**1Eu**	7.68	0.0155	–	0.5695	1145.54	838.04
**1Eu** + 4,4′-bipyiridine	7.27	0.0148	0.0011	0.7215		700.19
**1Eu** + pyridine	7.68	0.0155	0.0098	0.5695		838.04
**1Yb** + 4,4′-bipyiridine	–	0.0153	0.0009		–	–
**1Yb** + pyridine	–	0.0150	0.0011		–	–

[A] The small masses engender large errors in this methodology, the results should be cautiously interpreted along with other data. [B] Spectrometer frequency 400.130 MHz. Diamagnetic correction of M_r / −2,000,000 applied. ρd_6-benzene = 0.950 g mL^{-1}.

No change was observed in the ^{1}H NMR spectra of **1Sm**, **1Eu**, and **1Yb** upon addition of pyridine or 4,4′-bipyridine. For **1Sm** and **1Eu** the magnetic susceptibility was determined by Evans method and shows no change in oxidation state upon addition of 4,4′-bipyridine or pyridine (see **Table S10**). For **1Yb** the ^{1}H NMR scale reactions with 4,4′-bipyridine and pyridine yielded diamagnetic spectra that are consistent with a 4f^{14} Yb(II) ion, and are identical to **1Yb**, with the only additional peaks corresponding to unreacted 4,4′-bipyridine and pyridine (**Figure S134** and **Figure S135**). The structure remains C_2 symmetric in solution, as shown by the six doublets for the CH_3-*i*Pr groups which suggests that no adduct has formed.

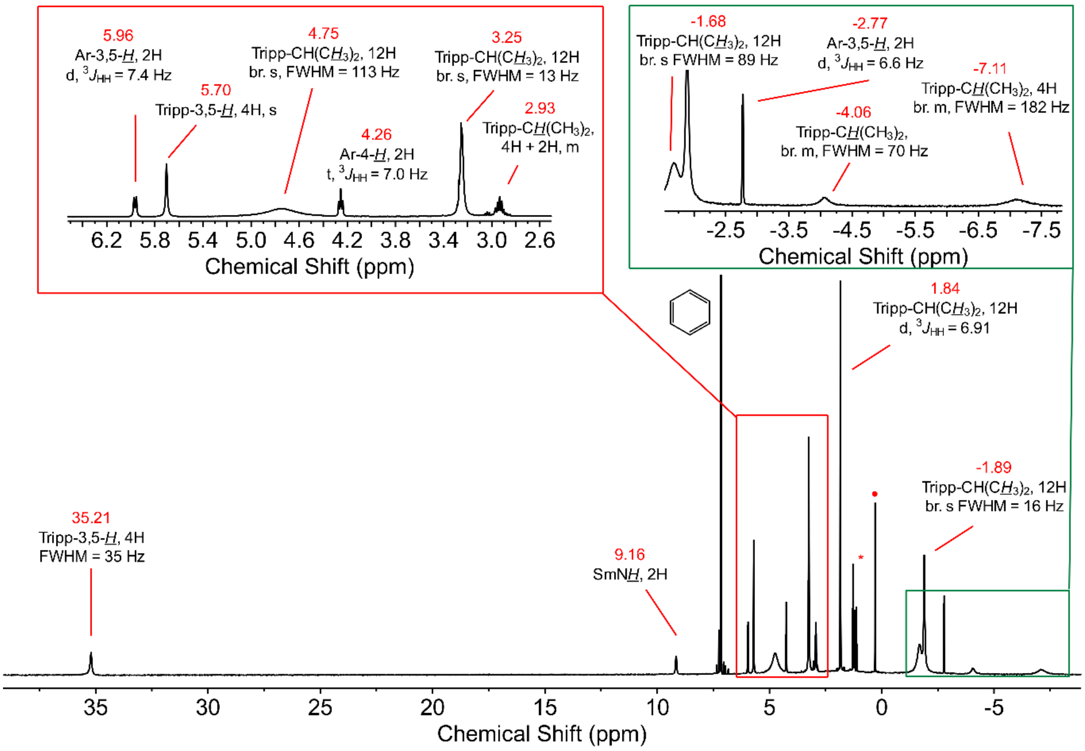

Figure S127. ^{1}H NMR spectrum of **1Sm** in d_6-benzene. * and • denotes protic ligand (H_2NAr^{iPr6}) and silicone grease respectively.

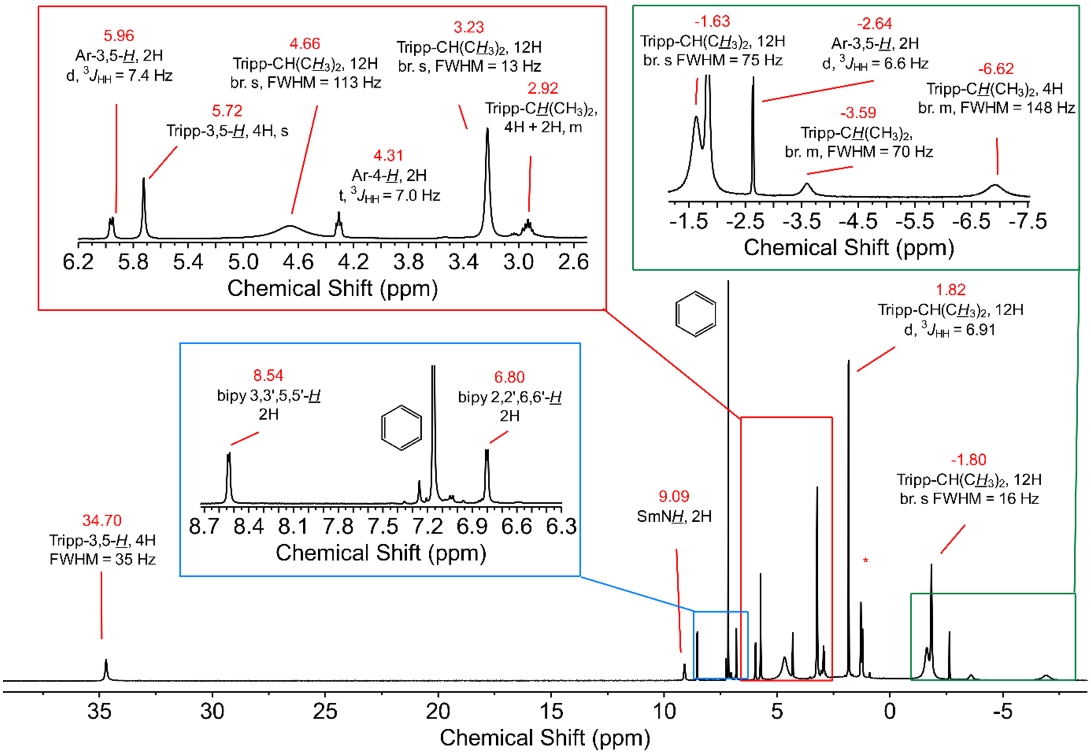

Figure S128. ^{1}H NMR spectrum of **1Sm** and 4,4′-bipyridine in d_6-benzene. * and • denotes protic ligand (H_2NAr^{iPr6}) and silicone grease respectively.

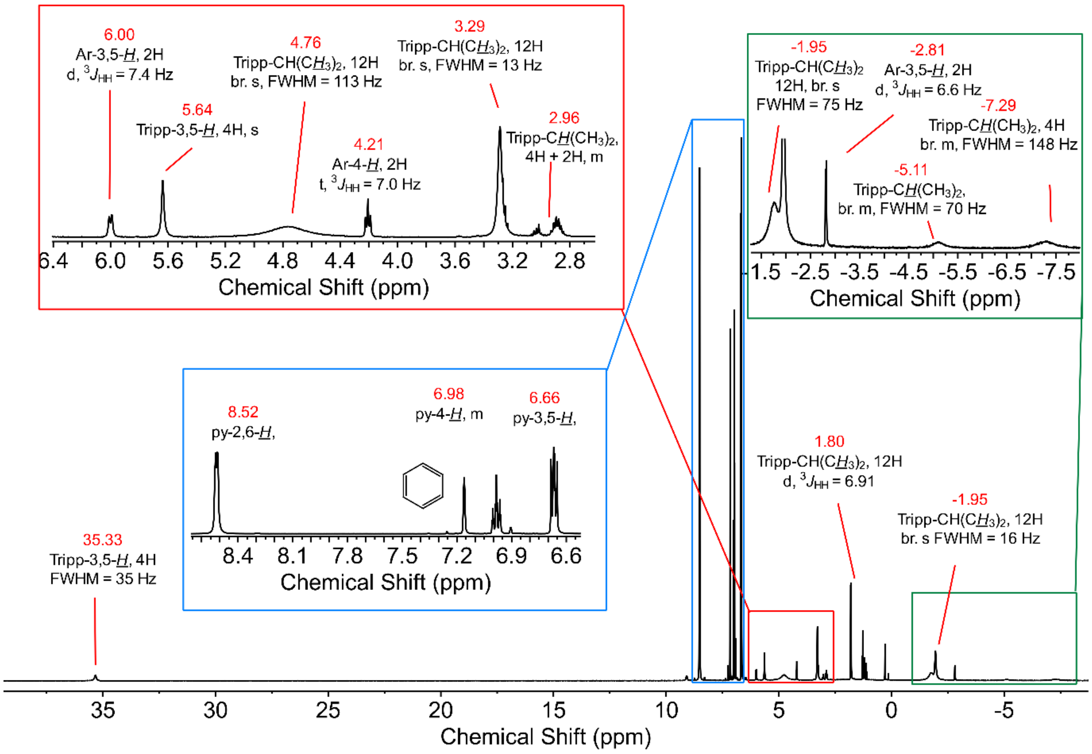

Figure S129. ^{1}H NMR spectrum of **1Sm** and pyridine (27 eq.) in d_6-benzene.

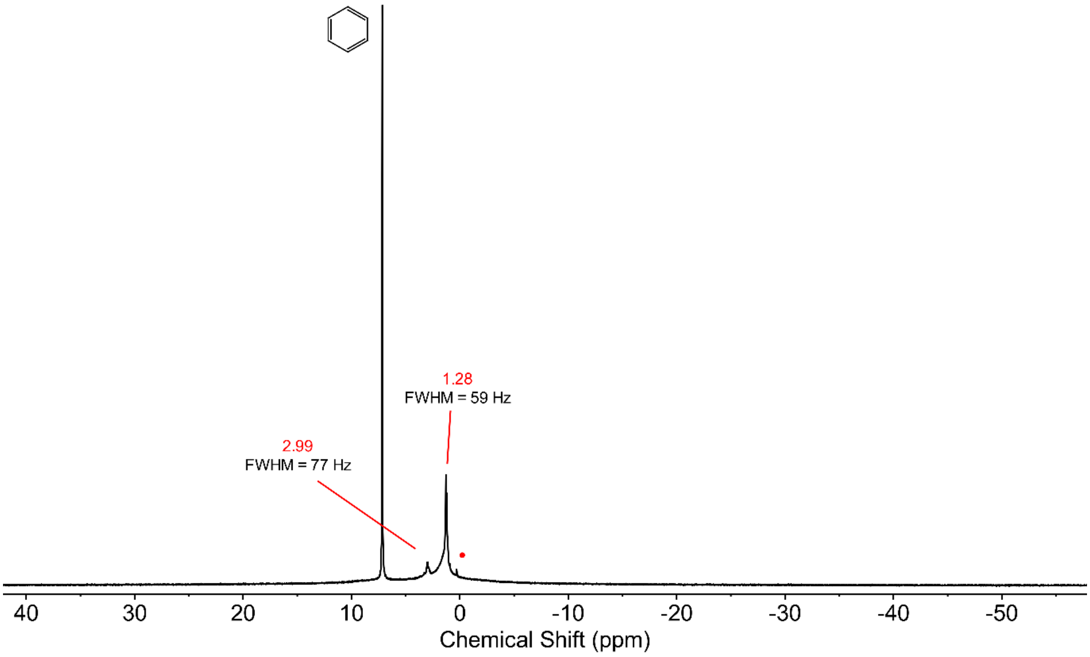

Figure S130. ^{1}H NMR spectrum of **1Eu** in d_6-benzene, • denotes silicone grease.

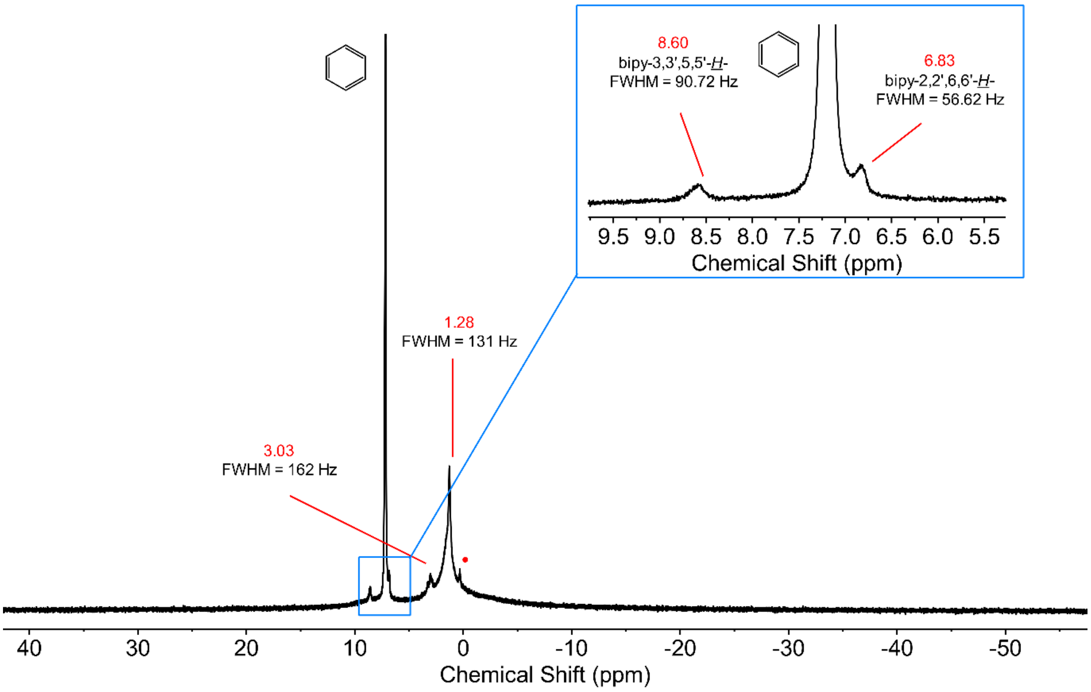

Figure S131. ^{1}H NMR spectrum of **1Eu** and 4,4′-bipyridine in d_6-benzene, • denotes silicone grease.

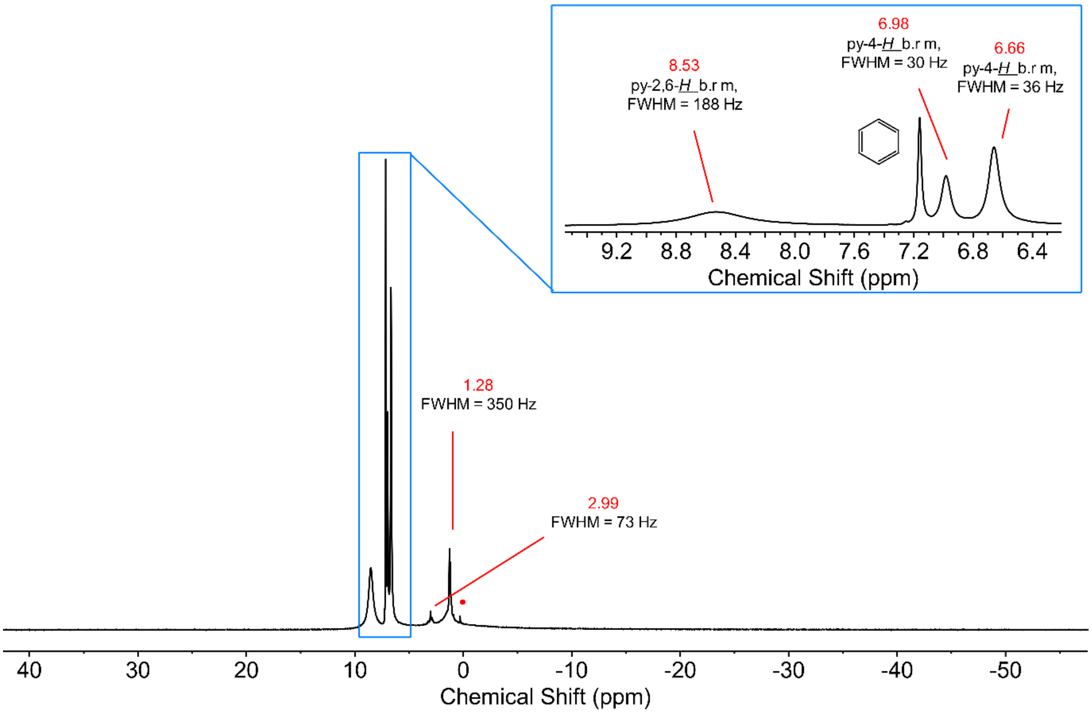

Figure S132. ^{1}H NMR spectrum of **1Eu** and pyridine in d_6-benzene, • denotes silicone grease.

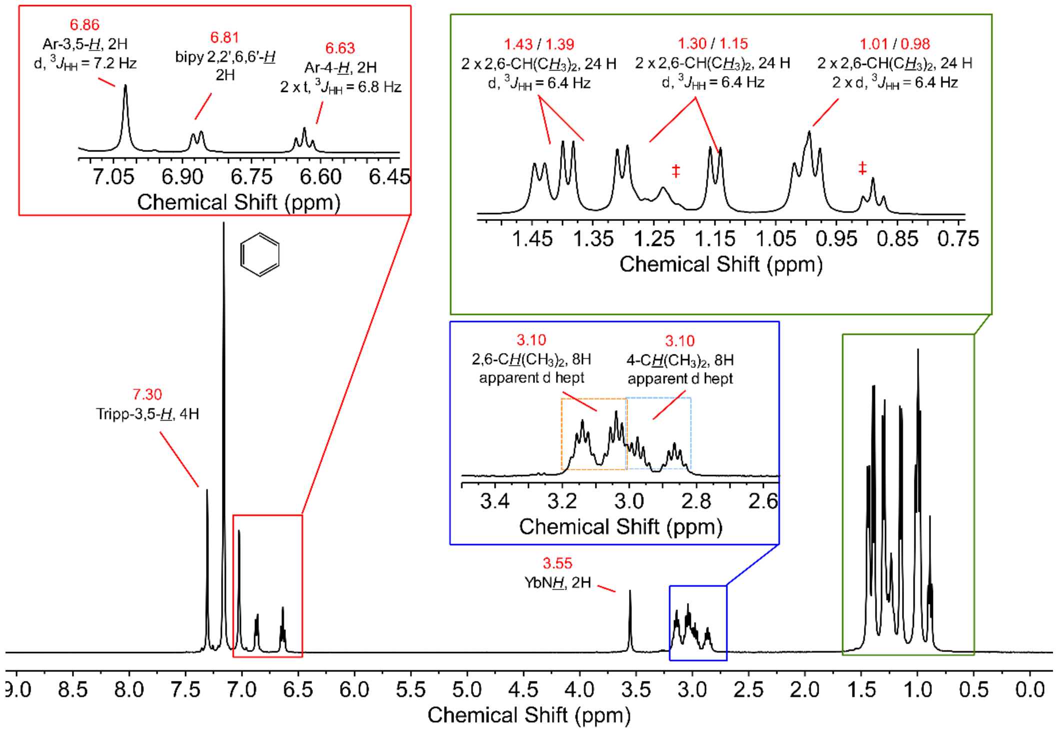

Figure S133. ^{1}H NMR spectrum of **1Yb** in d_6-benzene. ‡ denotes residual *n*-hexane.

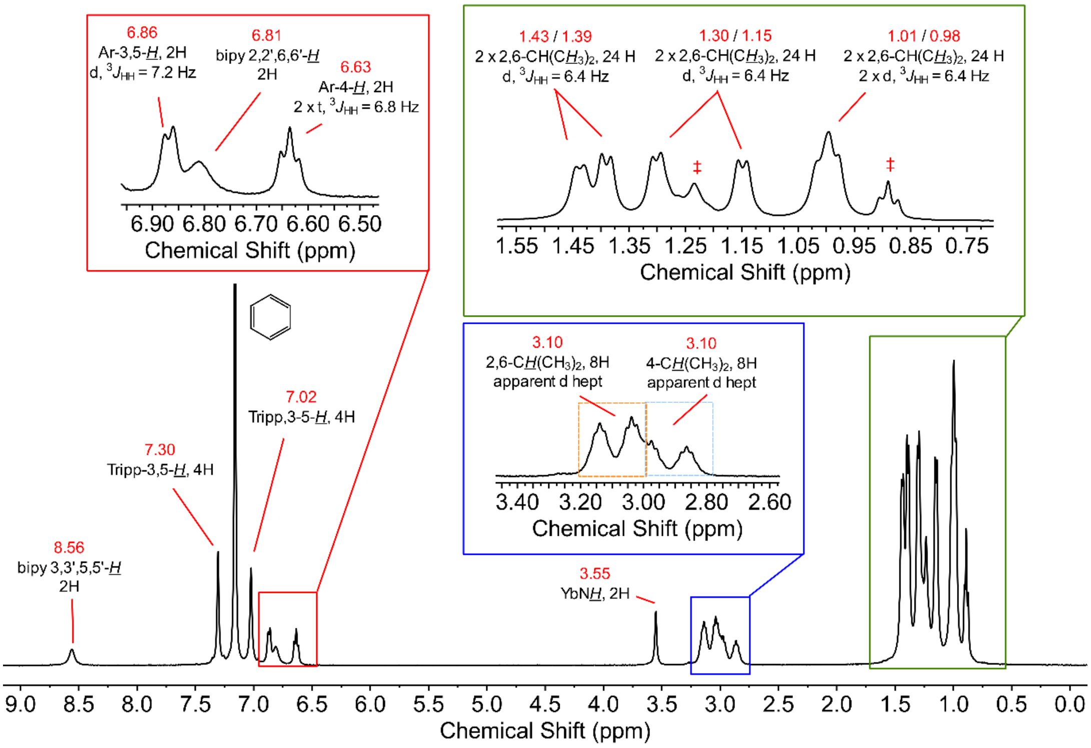

Figure S134. ^{1}H NMR spectrum of **1Yb** and 4,4′-bipyridine in d_6-benzene. ‡ denotes residual *n*-hexane.

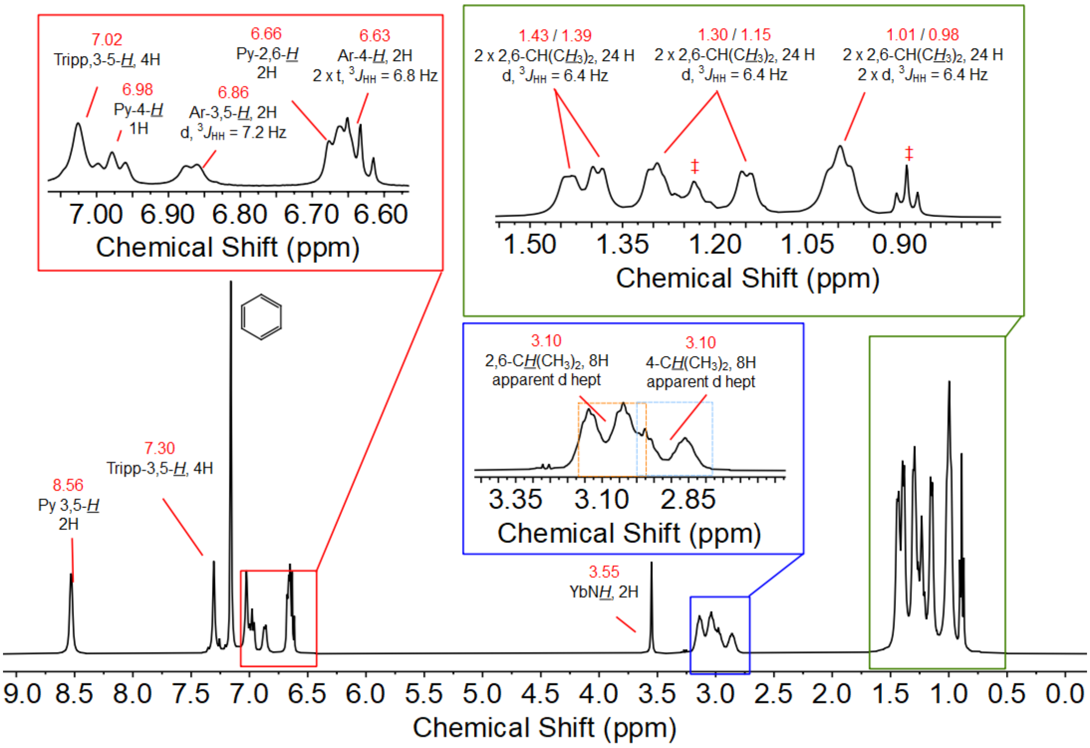

Figure S135. ^{1}H NMR spectrum of **1Yb** and pyridine in d_6-benzene. ‡ denotes residual *n*-hexane.

UV-Vis-NIR spectroscopy

In-situ UV-Vis-NIR spectroscopy measurements were performed on **1Sm**, **1Eu**, and **1Yb** to further investigate the interaction between the metal centres and 4,4′-bipyridine or pyridine, and to corroborate the oxidation states suggested by the magnetic susceptibility data from NMR studies.

Solutions of **1Sm**, **1Eu**, and **1Yb** in toluene were diluted using stock solutions of 4,4′-bipyridine and pyridine such that the final concentration of each complex was 1 mM, and 4,4′-bipyridine and pyridine were present in ratios of 1:0.5 and 1:1, respectively. UV-Vis-NIR spectra were recorded from 2,000–200 nm (5,000–50,000 cm^{-1}), and show resultant spectra that are identical to those of **1M**, suggesting no change in oxidation state nor a change in the coordination environment.

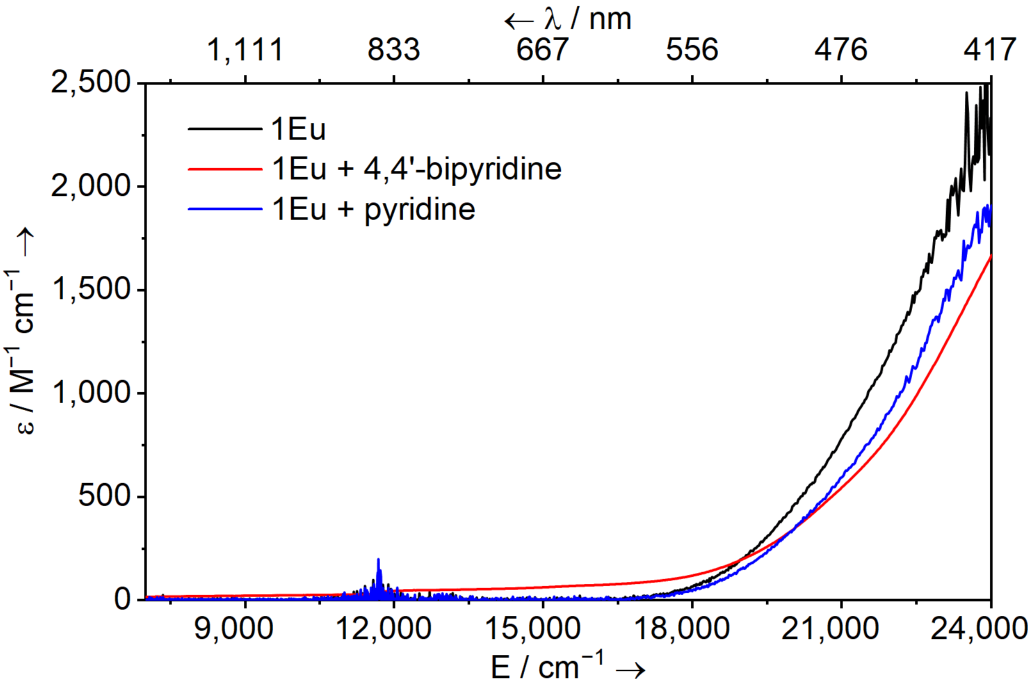

Figure S136. Solution UV-Vis-NIR spectra of **1Eu** (black), **1Eu** + 4,4′-bipyidine (red), and **1Eu** + pyridine (blue) (1.0 mM) in toluene shown between 8,000–25,000 cm^{-1} (1,250–400 nm) at ambient temperature.

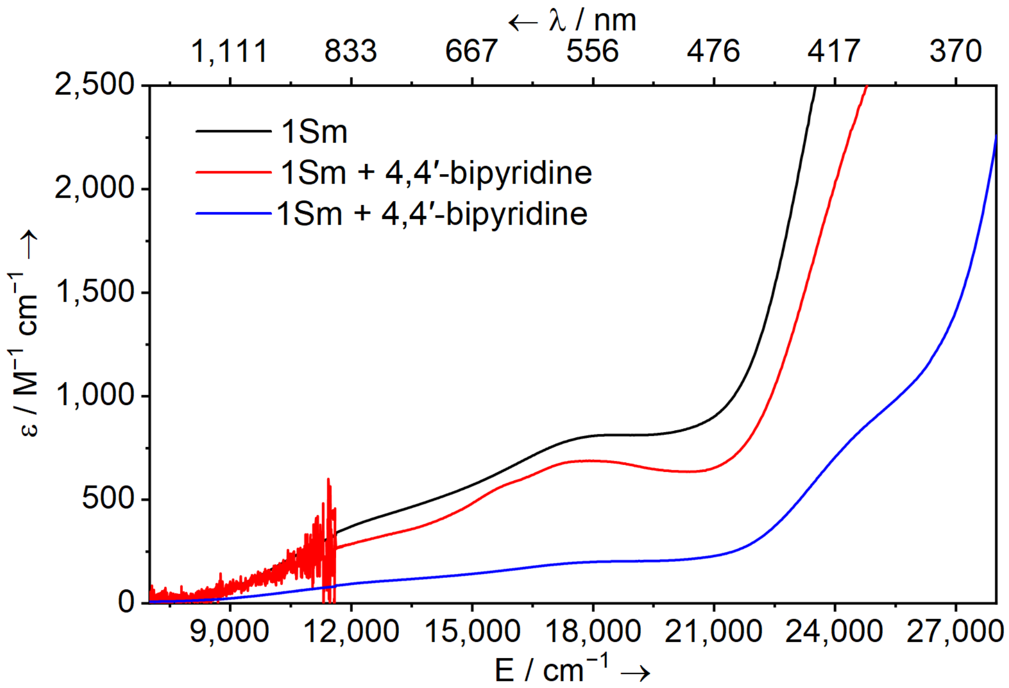

Figure S137. Solution UV-Vis-NIR spectra of **1Sm** (black), **1Sm** + 4,4-bipyidine (red), and **1Sm** + pyridine (blue) (1.0 mM) in toluene shown between 8,000–25,000 cm^{-1} (1,250–400 nm) at ambient temperature.

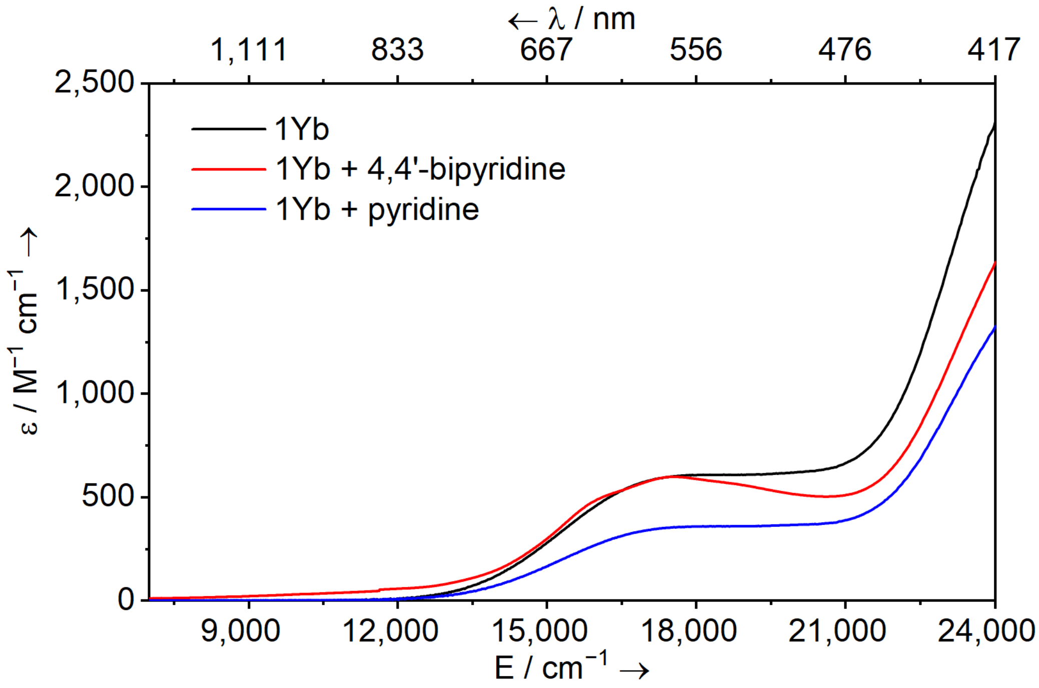

Figure S138. Solution UV-Vis-NIR spectra of **1Yb** (black), **1Yb** + 4,4′-bipyidine (red), and **1Yb** + pyridine (blue) (1.0 mM) in toluene shown between 8,000–25,000 cm^{-1} (1,250–400 nm) at ambient temperature.

S9. Reactivity study with 1M (M = Y, La, Tm, Lu)

NMR spectroscopy

Solutions of **1M** (M = Y, La, Lu) and a known quantity of hexamethylbenzene (HMB) to act as an internal standard were prepared in d_6-benzene. Aliquots of a stock solution of 4,4′-bipyridine in d_6-benzene were added, and the ^{1}H NMR spectrum was recorded. This process was repeated until approximately one equivalent of 4,4′-bipyridine, with respect to the starting metal content, had been added to each sample – noting that only 0.5 equivalents of 4,4′-bipyridine are required to produce **2M**.

The **2M** complexes formed during this process had a tendency to slowly precipitate from the d_6-benzene solution, so attempts to quantify reaction products and intermediates were not possible. **Table S11** details the quantity of 4,4′-bipyridine added for each of the six NMR spectra given for each complex in **Figure S139** to **Figure S141**.

Table S11. Number of equivalents of 4,4′-bipyridine in solution for **1Y**, **1La**, and **1Lu** for NMR spectra given in **Figure S139** to **Figure S141**

**Run #**	**1Y**	**1La**	**1Lu**
	Equivalents of 4,4′-bipyridine added		
**1**	0	0	0
**2**	0.14	0.11	0.15
**3**	0.47	0.36	0.34
**4**	0.63	0.47	0.51
**5**	0.8	0.66	0.69
**6**	1.05	0.94	1.25

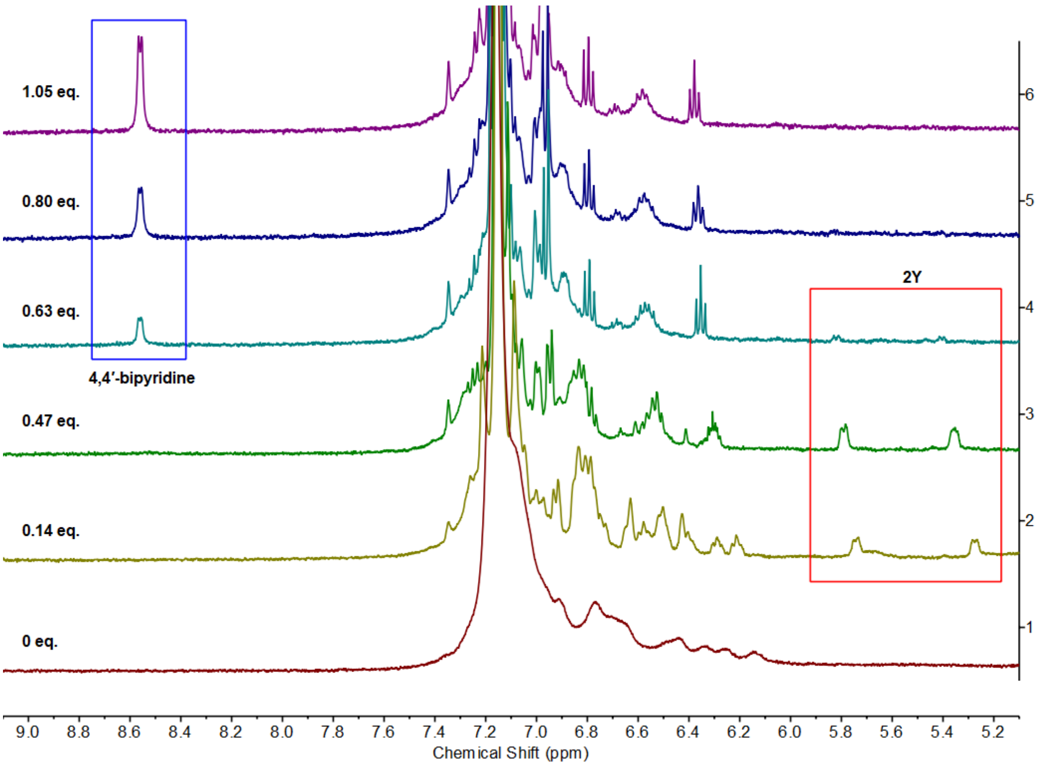

Figure S139. Stacked ^{1}H NMR spectra of **1Y** with increasing 4,4′-bipyridine concentration. Spectral range from 5.0 to 9.5 ppm.

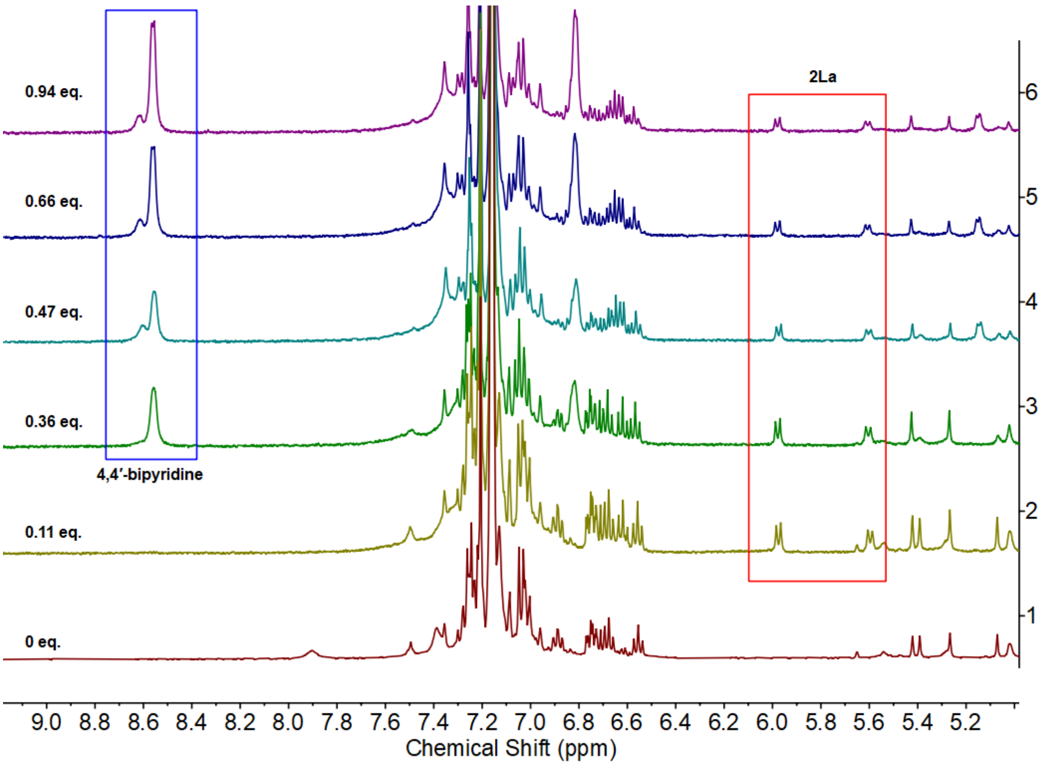

Figure S140. Stacked ^{1}H NMR spectra of **1La** with increasing 4,4′-bipyridine concentration. Spectral range from 5.0 to 9.5 ppm.

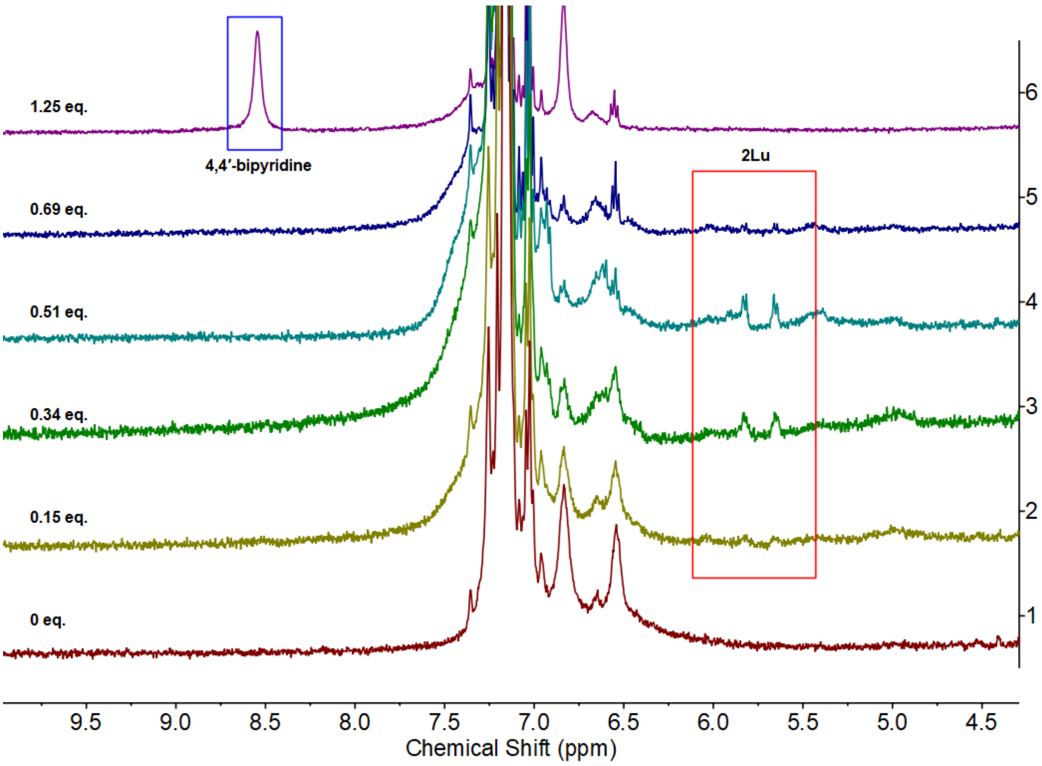

Figure S141. Stacked ^{1}H NMR spectra of **1Lu** with increasing 4,4′-bipyridine concentration. Spectral range from 5.0 to 9.5 ppm.

Reactions with naphthalene and anthracene

Naphthalene: Naphthalene (*ca*.1 eq.) was added to a d_6-benzene solution of **1M** (**1Y** = 9.7 mg, naphthalene = 1.21 mg; **1La** = 10.4 mg, naphthalene = 1.18 mg; **1Lu** = 10.8 mg, naphthalene = 0.98 mg) and the ^{1}H NMR spectra collected. No colour change was observed with the spectra, only showing naphthalene unchanged and small amounts of protic ligand (H_2NAr^{iPr6}) from decomposition. See **Figure S142** to **Figure S144** for ^{1}H NMR spectra.

Anthracene: Anthracene (*ca*.1 eq.) was added to a d_6-benzene solution of **1M** (**1Y** = 7.8 mg, anthracene = 3.2 mg; **1La** = 9.5 mg, anthracene = 5.2 mg; **1Lu** = 8.8 mg, anthracene = 5.3 mg) and the ^{1}H NMR spectra collected. No colour change was observed with the spectra, only showing anthracene unchanged and small amounts of protic ligand (H_2NAr^{iPr6}) from decomposition. See **Figure S145** to **Figure S147** for ^{1}H NMR spectra.

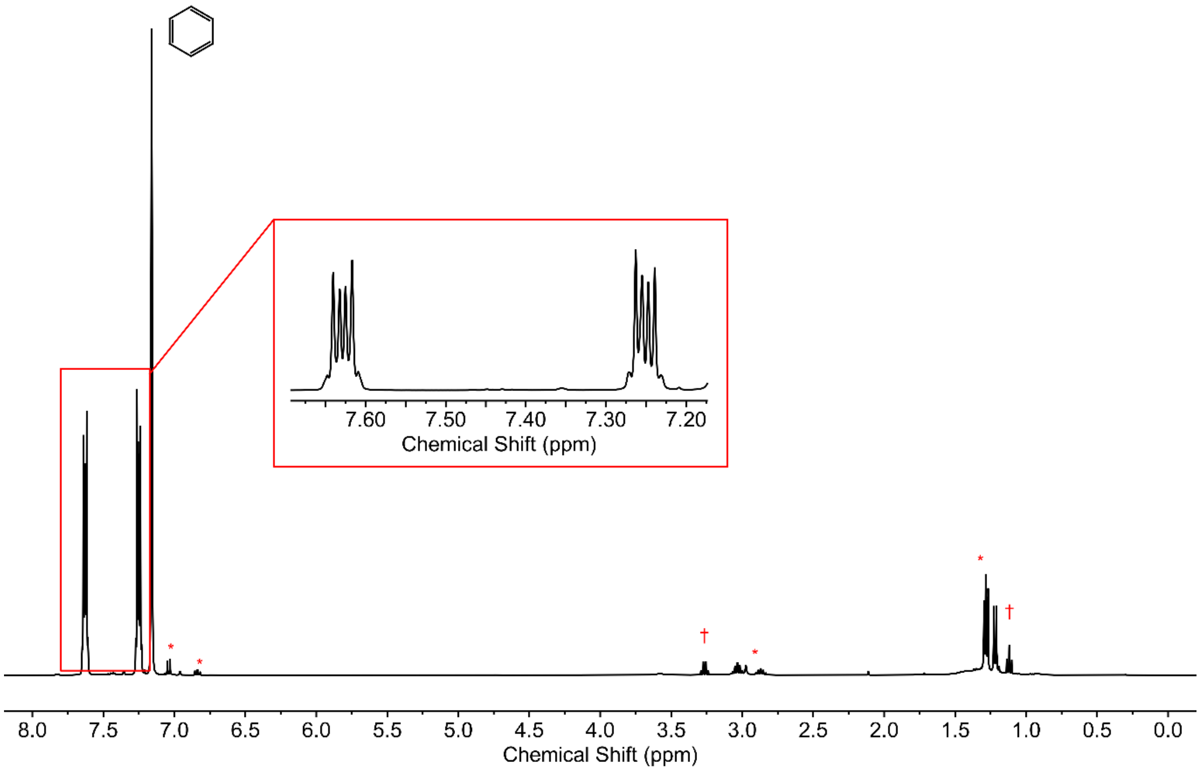

Figure S142. ^{1}H NMR spectrum of **1Y** and naphthalene in d_6-benzene. † denotes residual Et_2O, * denotes protic ligand (H_2NAr^{iPr6}).

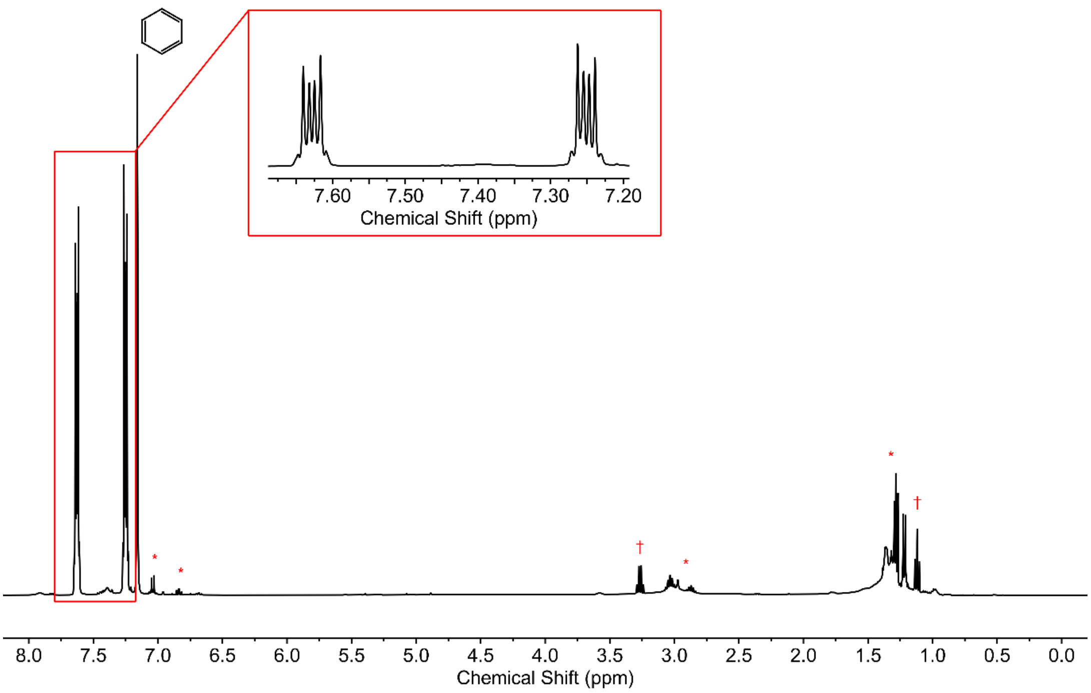

Figure S143. ^{1}H NMR spectrum of **1La** and naphthalene in d_6-benzene. † denotes residual Et_2O, * denotes protic ligand (H_2NAr^{iPr6}).

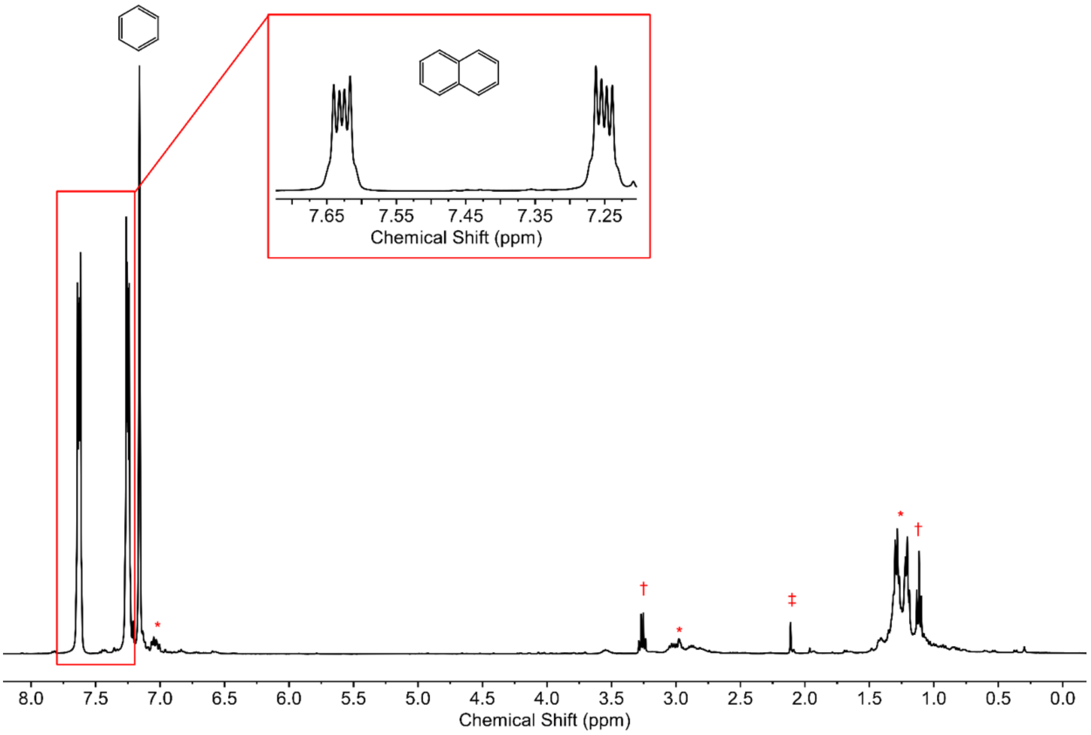

Figure S144. ^{1}H NMR spectrum of **1Lu** and naphthalene in d_6-benzene. ‡ and † denote residual toluene and Et_2O respectively, * denotes protic ligand (H_2NAr^{iPr6}).

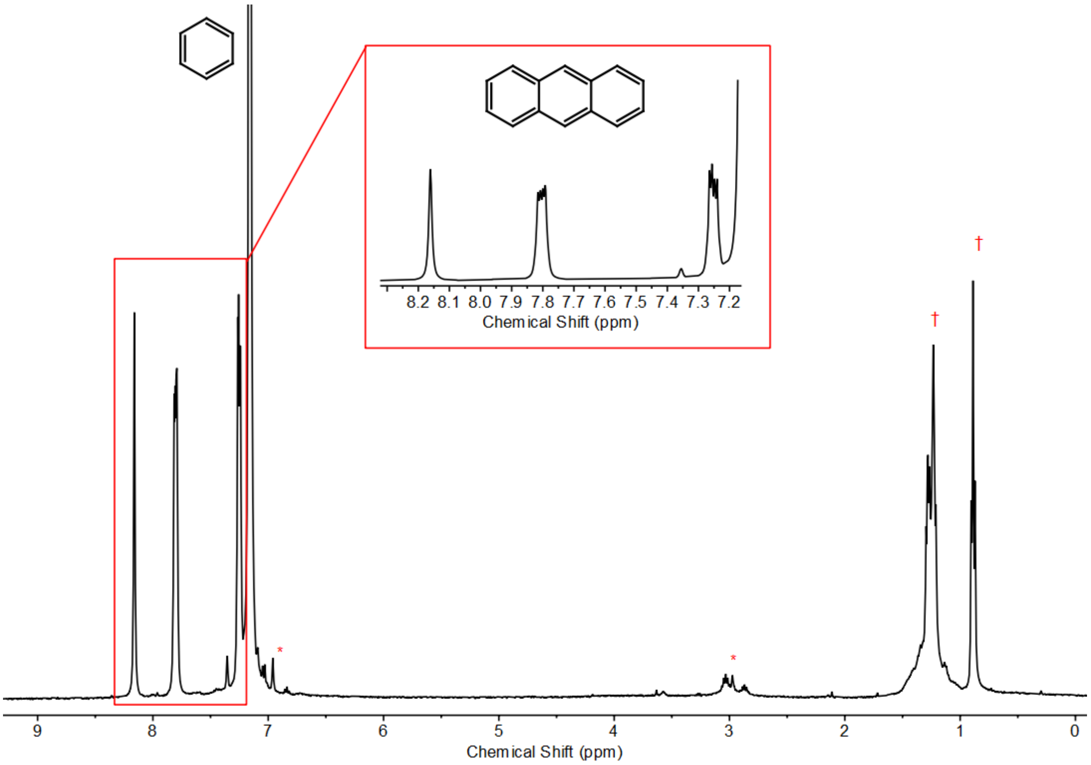

Figure S145. ^{1}H NMR spectrum of **1Y** and anthracene in d_6-benzene. † denotes residual hexane, * denotes protic ligand (H_2NAr^{iPr6}).

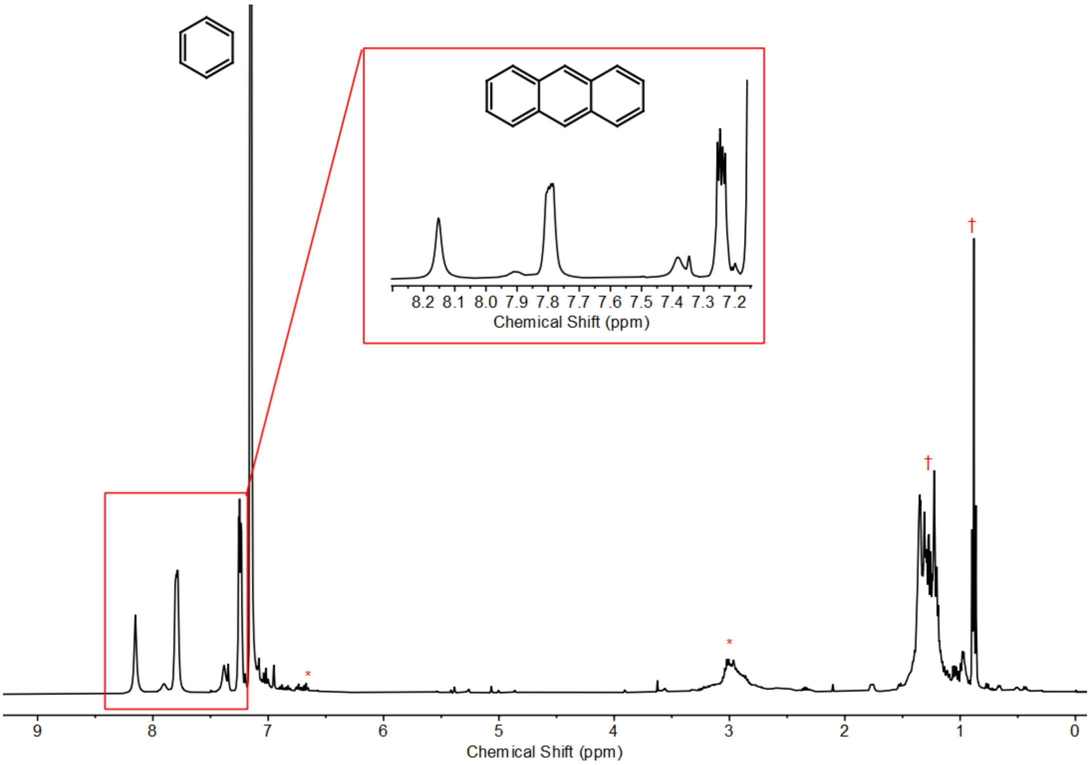

Figure S146. ^{1}H NMR spectrum of **1La** and anthracene in d_6-benzene. † denotes residual hexane, * denotes protic ligand (H_2NAr^{iPr6}).

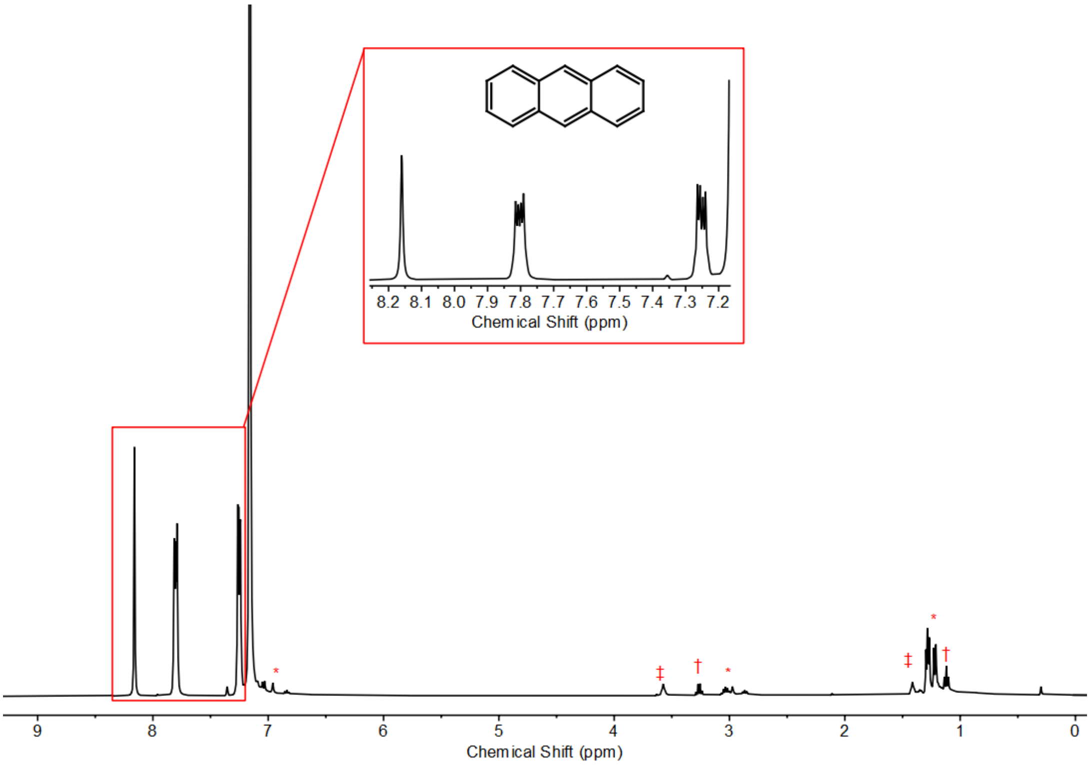

Figure S147. ^{1}H NMR spectrum of **1Lu** and anthracene in d_6-benzene. † and ‡ denote residual Et_2O and THF respectively, * denotes protic ligand (H_2NAr^{iPr6}).

UV-Vis-NIR spectroscopy

Separate stock solutions containing known concentrations of **1M** (M = Y, La, Tm, Lu), or 4,4′-bipyridine, in toluene were prepared. Measured quantities of each were then transferred to a 4 mL J-Youngs quartz cuvette with a 1 cm path length and diluted further with weighed quantities of additional toluene such that the total metal concentration, and the ratio of **1M** to 4,4′-bipyridine were known.

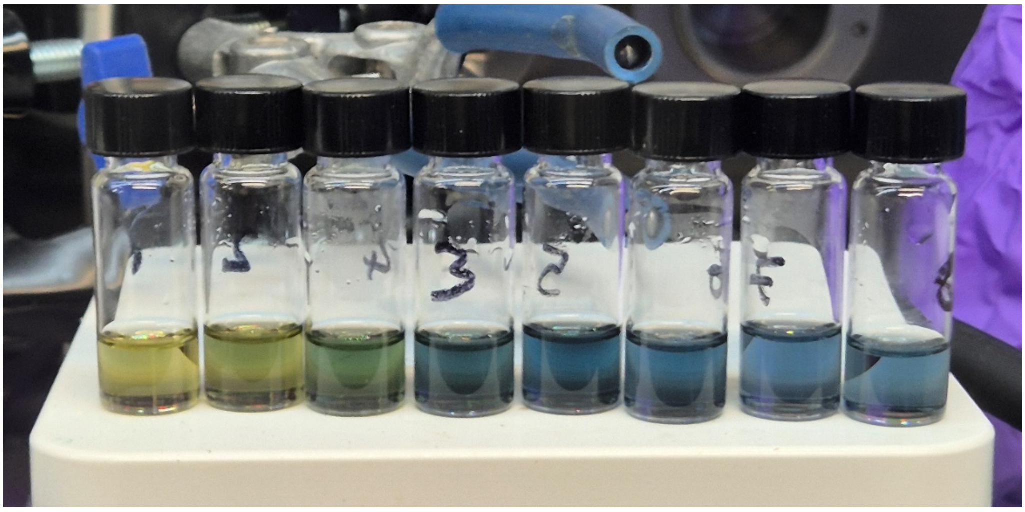

Figure S148. Photo taken during preparation of **1Lu** solutions showing the colour change from green to blue as the concentration of 4,4′-bipyridine increases (left to right).

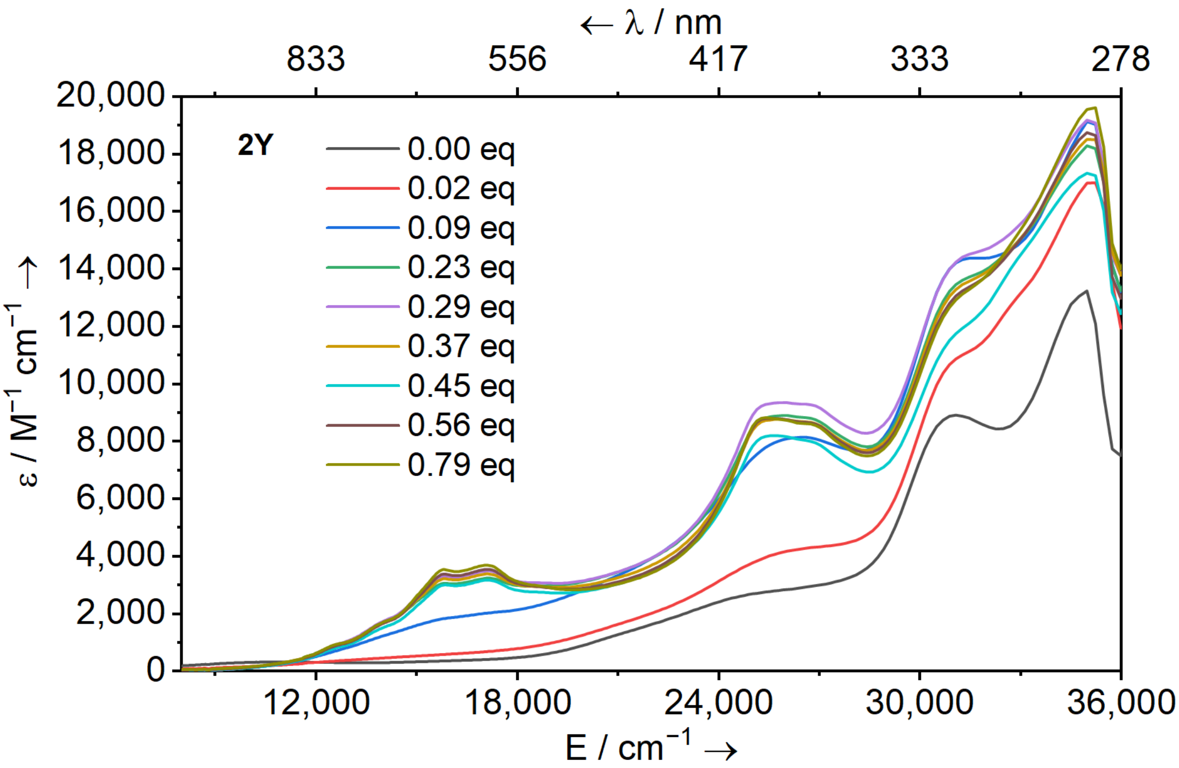

Figure S149. Solution of UV-Vis-NIR of **1Y** with increasing concentration of 4,4′-bipyridine in toluene in a 1 cm path length shown between 8,000–36,000 cm^{-1} (1,250–278 nm) at ambient temperature.

Table S12. Masses, concentrations, and equivalents of 4,4′-bipyridine added to **1Y**.

Run	Equivalents of 4,4′-bipyridine	Concentration (mM)
1	0.03	0.086
2	0.23	0.092
3	0.10	0.095
4	0.29	0.092
5	0.37	0.087
6	0.45	0.099
7	0.56	0.099
8	0.79	0.086

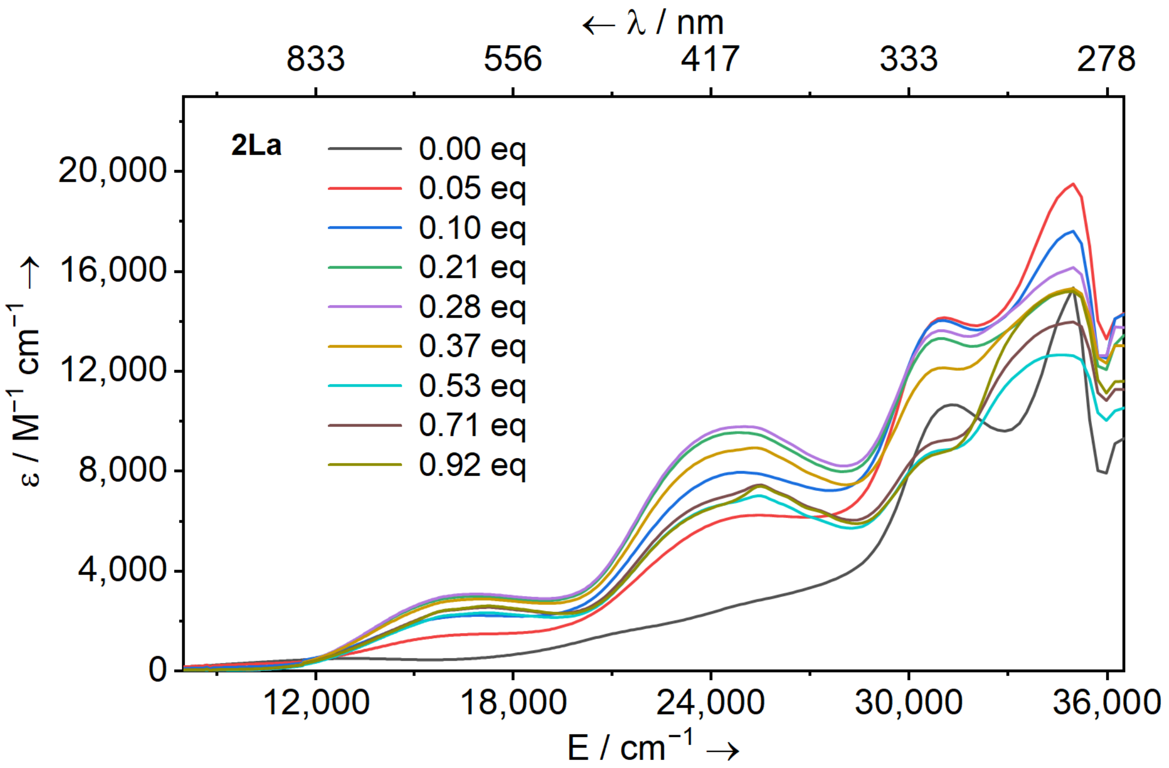

Figure S150. Solution of UV-Vis-NIR of **1La** with increasing concentration of 4,4′-bipyridine in toluene in a 1 cm path length shown between 8,000–36,000 cm^{-1} (1,250–278 nm) at ambient temperature.

Table S13. Masses, concentrations, and equivalents of 4,4′-bipyridine added to **1La**.

Run	Equivalents of 4,4′-bipyridine	Concentration (mM)
**1**	0.05	0.090
**2**	0.10	0.102
**3**	0.21	0.099
**4**	0.28	0.098
**5**	0.37	0.091
**6**	0.53	0.105
**7**	0.71	0.102
**8**	0.92	0.101

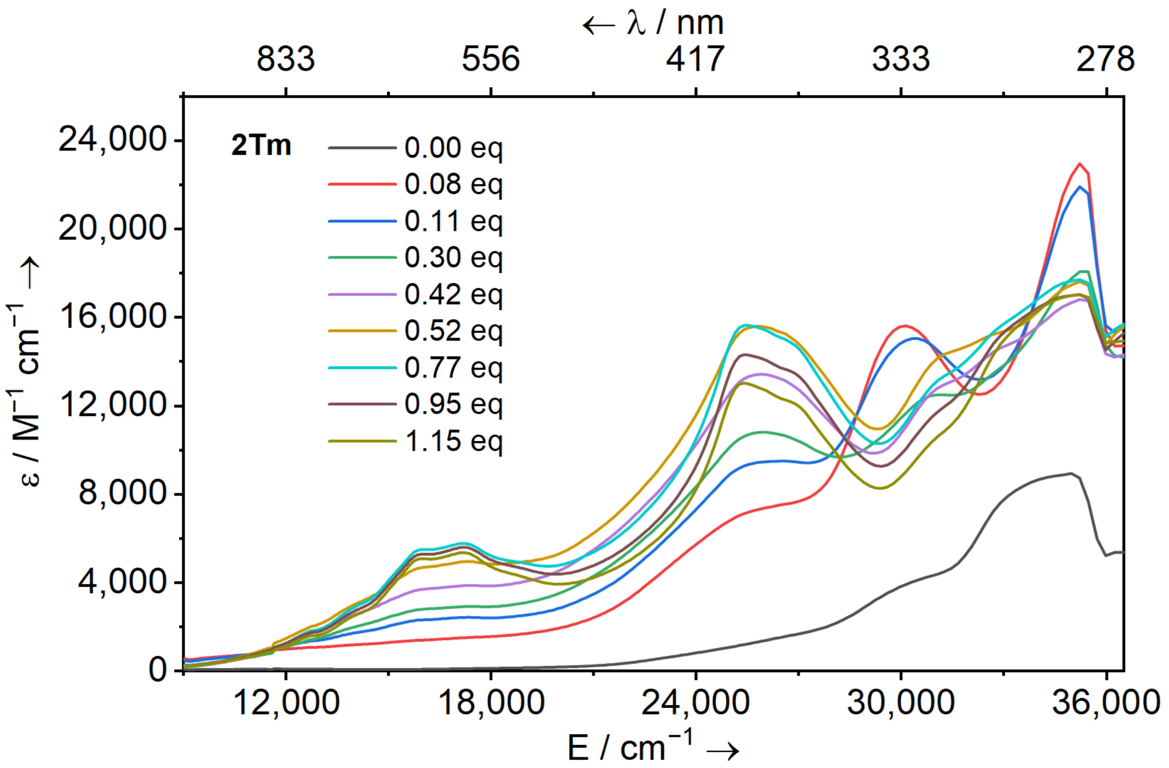

Figure S151. Solution of UV-Vis-NIR of **1Tm** with increasing concentration of 4,4′-bipyridine in toluene in a 1 cm path length shown between 8,000–36,000 cm^{-1} (1,250–278 nm) at ambient temperature.

Table S14. Masses, concentrations, and equivalents of 4,4′-bipyridine added to **1Tm**.

Run	Equivalents of 4,4′-bipyridine	Concentration (mM)
**1**	0.08	0.088
**2**	0.11	0.088
**3**	0.30	0.078
**4**	0.42	0.079
**5**	0.52	0.086
**6**	0.77	0.079
**7**	0.95	0.082
**8**	1.30	0.071

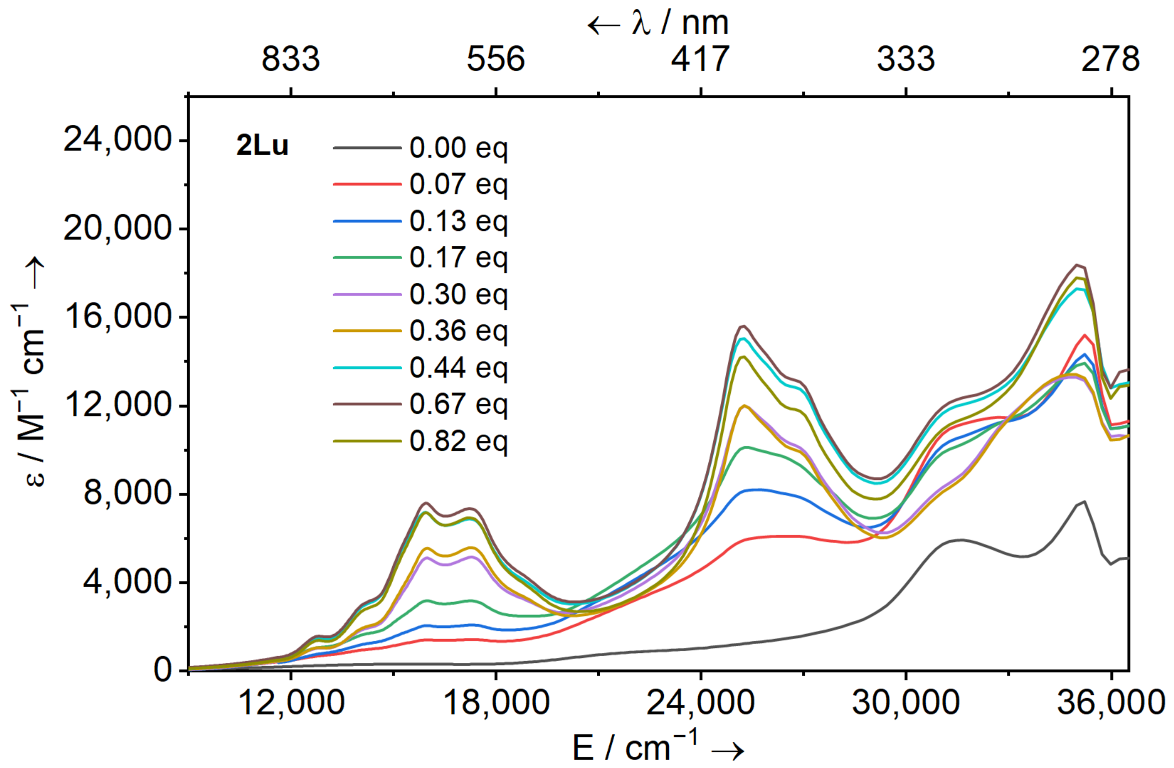

Figure S152. Solution of UV-Vis-NIR of **1Lu** with increasing concentration of 4,4′-bipyridine in toluene in a 1 cm path length shown between 8,000–36,000 cm^{-1} (1,250–278 nm) at ambient temperature.

Table S15. Masses, concentrations, and equivalents of 4,4′-bipyridine added to **1Lu**.

Run	Equivalents of 4,4′-bipyridine	Concentration (mM)
**1**	0.07	0.081
**2**	0.13	0.084
**3**	0.30	0.083
**4**	0.17	0.079
**5**	0.36	0.084
**6**	0.44	0.078
**7**	0.67	0.088
**8**	0.82	0.089

S10. EPR Spectroscopy

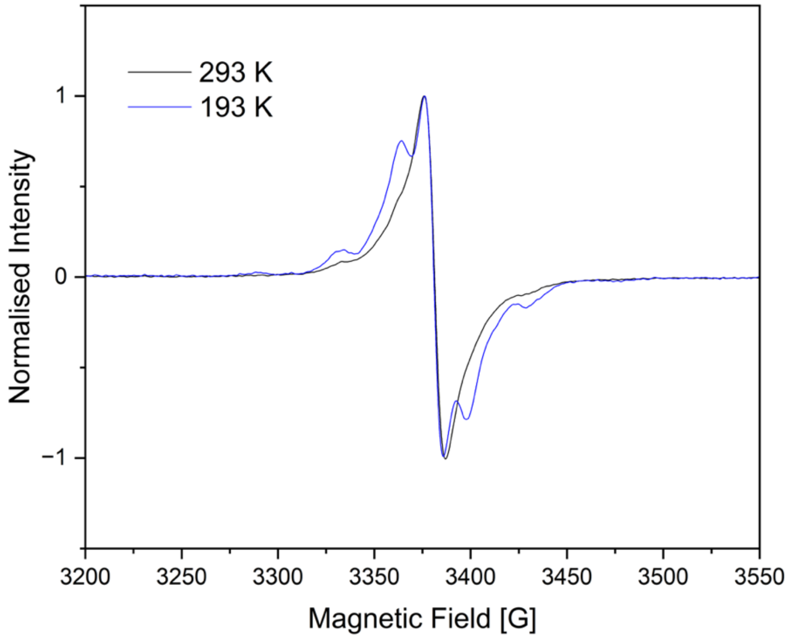

Figure S153. X-band c.w. EPR spectrum of 2.5 mM solution of **2Y** in mesitylene recorded at 293 K and 193 K.

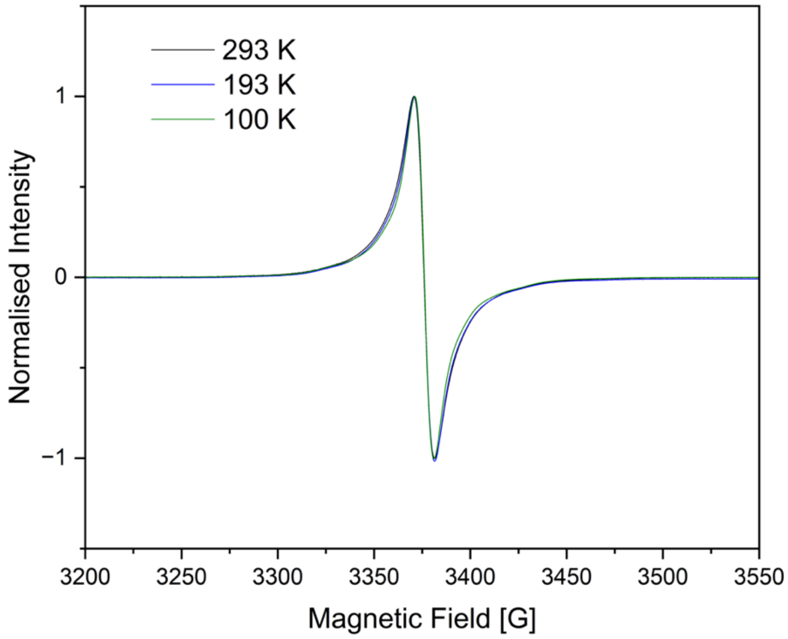

Figure S154. X-band polycrystalline **2Y** recorded at 293 K, 193 K, and 100 K.

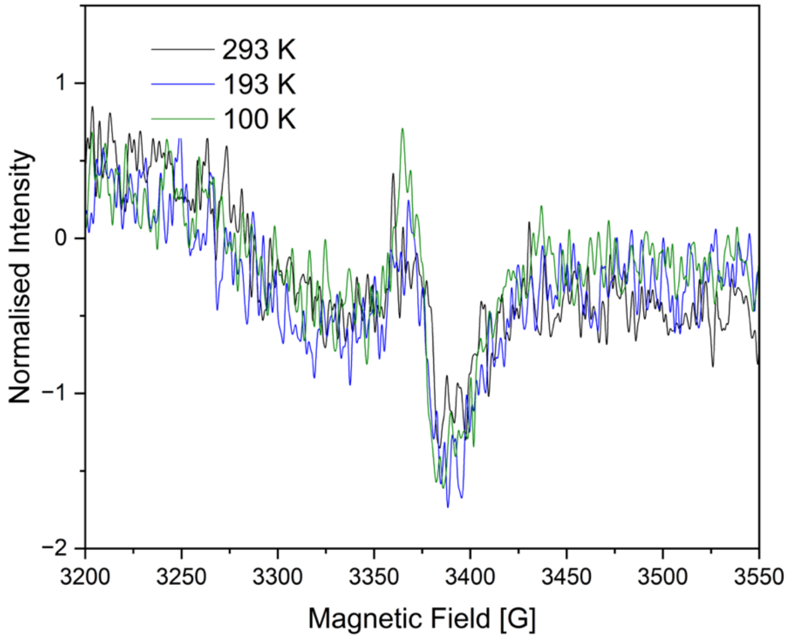

Figure S155. X-band c.w. EPR spectrum of 2.5 mM solution of **2La** in mesitylene recorded at 293 K, 193 K, and 100 K.

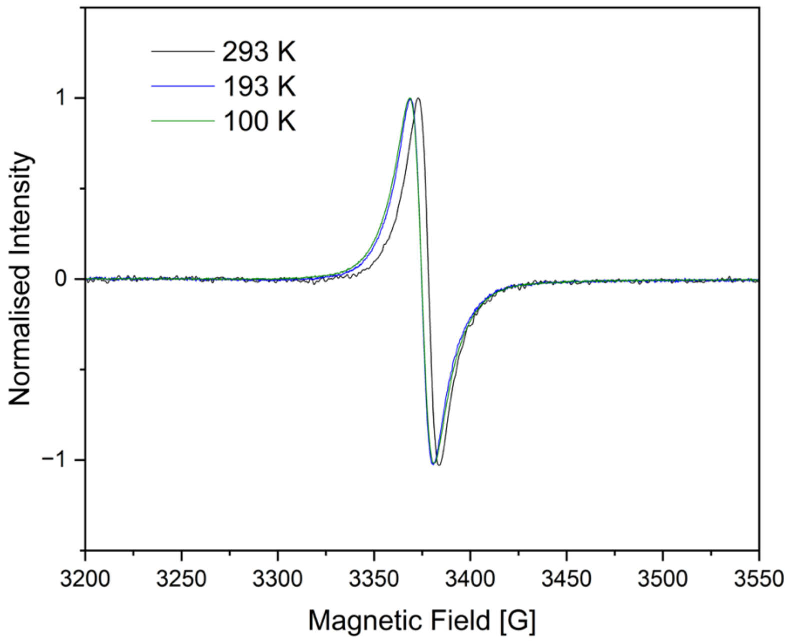

Figure S156. X-band polycrystalline **2La** recorded at 293 K, 193 K, and 100 K.

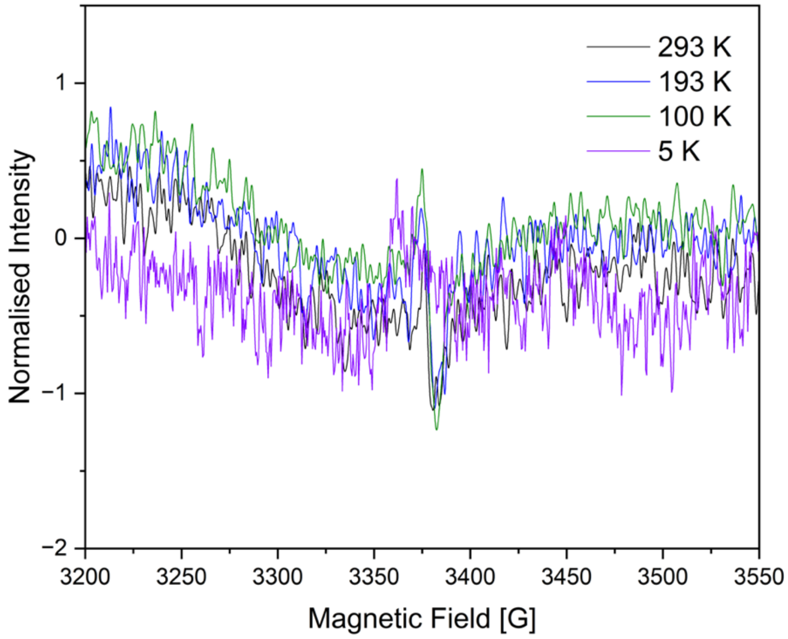

Figure S157. X-band c.w. EPR spectrum of 2.5 mM solution of **2Tm** in mesitylene recorded at 293 K, 193 K, 100 K, and 5K

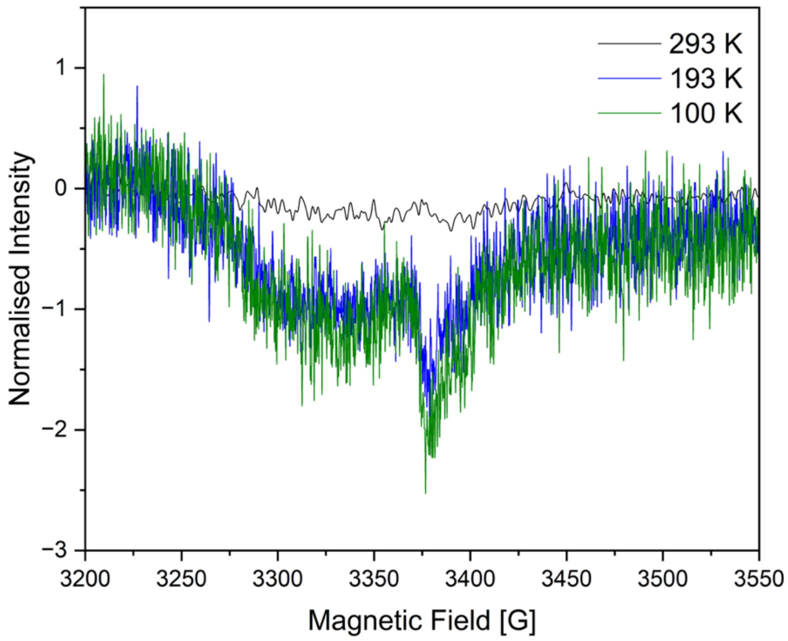

Figure S158. X-band polycrystalline **2Tm** recorded at 293 K, 193 K, and 100 K.

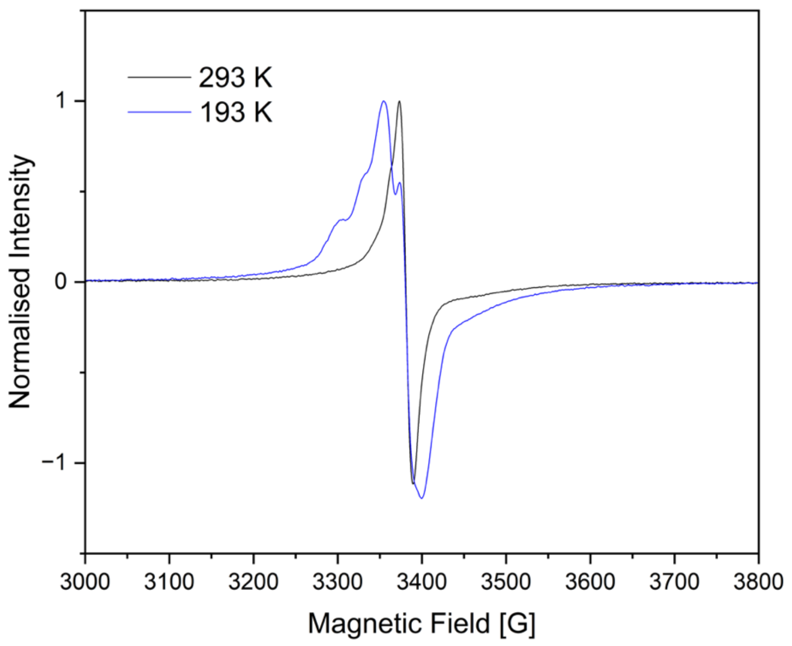

Figure S159. X-band c.w. EPR spectrum of 2.5 mM solution of **2Lu** in mesitylene recorded at 293 K and 193 K.

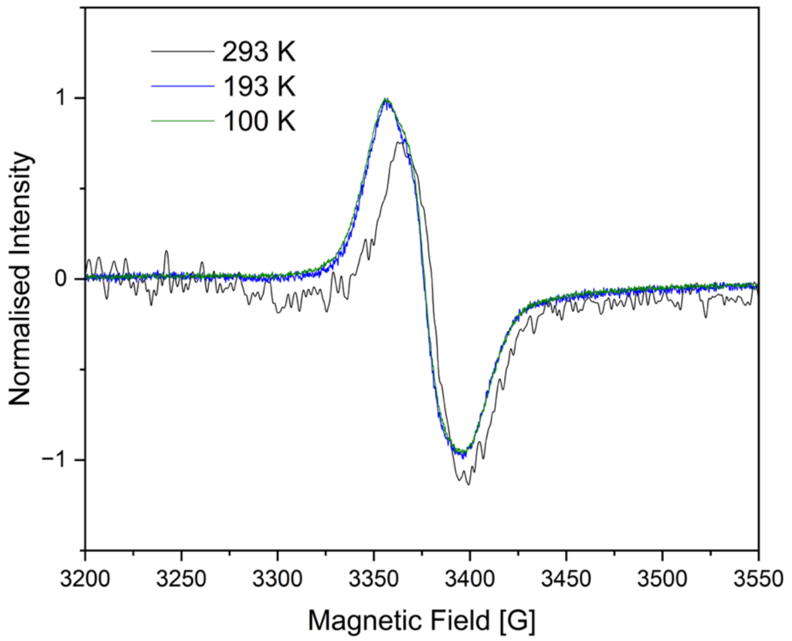

Figure S160. X-band polycrystalline **2Lu** recorded at 293 K, 193 K, and 100 K.

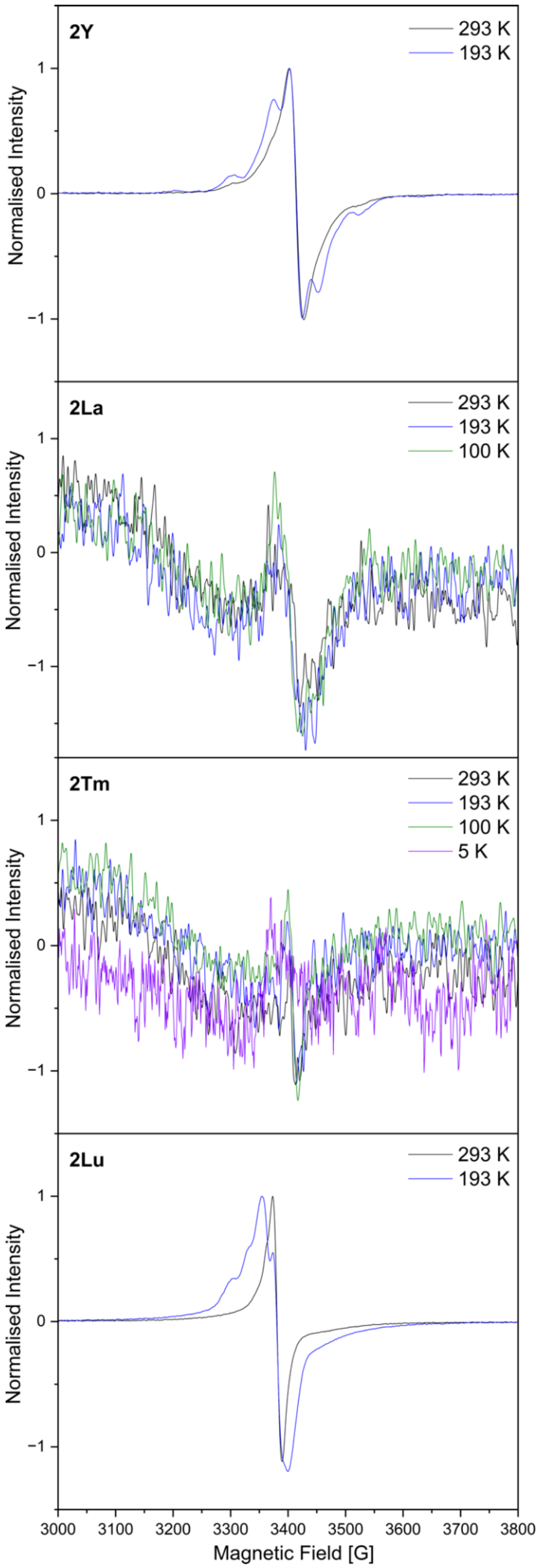

Figure S161. Stacked X-band c.w. EPR spectra of 2.5 mM solutions of **2M** (M = Y, La, Tm, Lu) complexes in mesitylene.

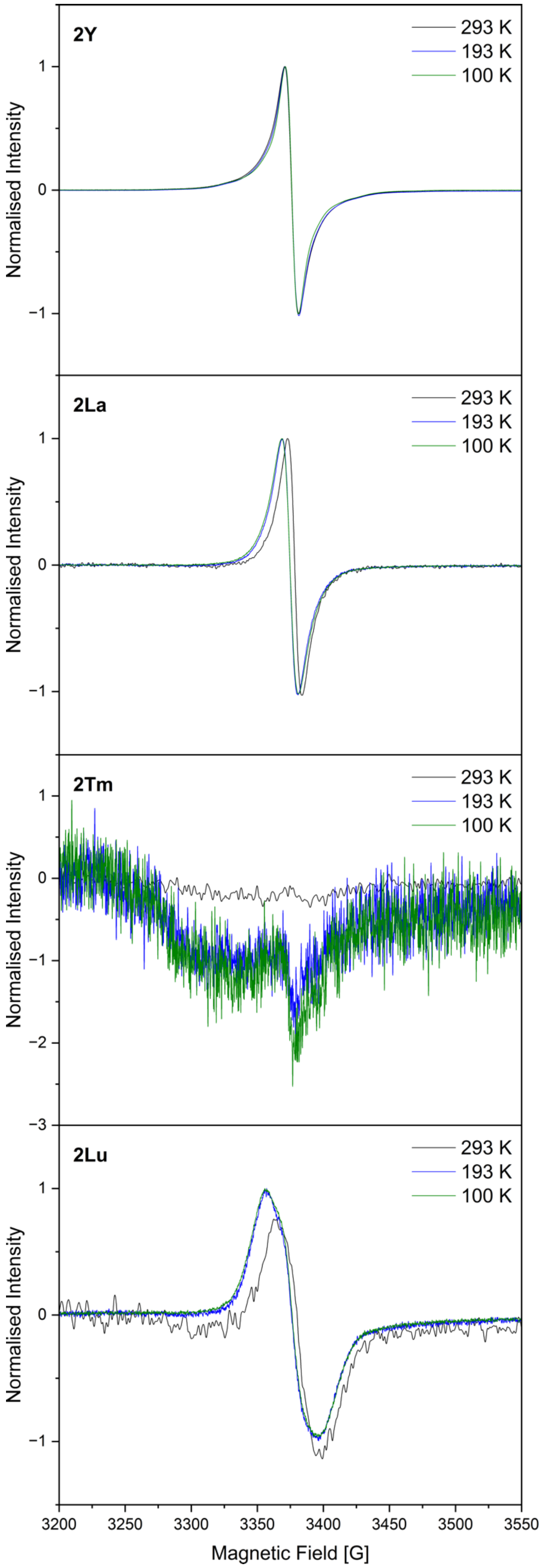

Figure S162. Stacked X-band polycrystalline EPR spectra of **2M** (M = Y, La, Tm, Lu) complexes.

S11. Computational methodology

General Considerations

Unrestricted Kohn-Sham DFT calculations were performed on *S* = ½ [$Y^{II}(NHAr^{iPr6})_2$] (**1Y**) at the following geometries:

(i) coordinates from the SC-XRD data, with H-atom positions optimised – denoted as "**1Y-SCXRD**";

(ii) an alternative geometry for **1Y** was derived from the structure of **1La**, all atom positions were optimised and the geometry was confirmed to be a local minimum by harmonic vibrational analysis – denoted as "**1Y-Opt**";

(iii) where the atom positions for the [$Y^{II}(NHAr^{iPr6})_2$] fragment were derived from the structure of **2Y**, with the rest of the structure removed, and the H-atom positions were optimised – denoted as "**1Y@2Y**".

The coordinates in (i) and (ii) have been determined previously, and were used here without modification.[1] All three coordinate sets are available from FigShare.

Calculations were performed using the ORCA 6.1 quantum chemistry program suite.[9] Geometry optimisations were performed using the TPSSh hybrid-meta functional,[10,11] with Grimme's D3BJ dispersion correction,[12-14] and the resolution of the identity 'chain of spheres' (RIJCOSX) approximation.[15,16] Solvation effects were accounted for using the conductor-like polarizable continuum model (CPCM).[17] No symmetry constraints were imposed. The SlowConv switch was used to ensure SCF convergence, along with the DefGrid3 (tight integration grid) switch, and tight geometry convergence criteria were used. **Table S16** shows key structural parameters for **1Y-SCXRD**, **1Y-Opt**, and **1Y@2Y**.

Table S16. Bond lengths (Å) and angles (°) for **1Y-SCXRD**, **1Y-Opt**, and **1Y@2Y**.

	**1Y-SCXRD**	**1Y-Opt**	**1Y@2Y**
Y(1)–N(1)	2.260	2.274	2.275
Y(1)–N(2)	– [A]	2.273	2.225
Y(1)–C_{range} (ring1)	2.728–2.980	2.559–2.757	2.769–3.146
Y(1)–C_{range} (ring2)	– [A]	2.930–3.520	–
Y(1)···$C_{6\text{-centroid}}$(1)	2.448	2.247	2.602
Y(1)···$C_{6\text{-centroid}}$(2)	– [A]	2.894	3.317
N(1)–Y(1)–N(2)	101.9	114.9	145.9

[A] The two ligands are related by symmetry in the SCXRD structure, and so the bond metrics for each are identical in this H-atom optimised coordinate set.

S12. References

1. R. E. MacKenzie, T. Hajdu, J. A. Seed, G. F. S. Whitehead, R. W. Adams, N. F. Chilton, D. Collison, E. J. L. McInnes and C. A. P. Goodwin, *Chem. Sci.*, 2024, **15**, 15160-15169.
2. N. Muller, P. C. Lauterbur and J. Goldenson, *J. Am. Chem. Soc.*, 2002, **78**, 3557-3561.
3. R. E. H. Kuveke, L. Barwise, Y. van Ingen, K. Vashisth, N. Roberts, S. S. Chitnis, J. L. Dutton, C. D. Martin and R. L. Melen, *ACS Cent. Sci.*, 2022, **8**, 855-863.
4. O. V. Dolomanov, L. J. Bourhis, R. J. Gildea, J. A. K. Howard and H. Puschmann, *J. Appl. Crystallogr.*, 2009, **42**, 339-341.
5. G. M. Sheldrick, *Acta Crystallogr., Sect. C*, 2015, **71**, 3-8.
6. G. M. Sheldrick, *Acta Crystallogr., Sect. A*, 2008, **64**, 112-122.
7. W. Clegg, A. J. Blake, J. M. Cole, J. S. O. Evans, P. Main, S. Parsons and D. J. Watkin, *Crystal Structure Analysis*, Oxford University Press, Oxford, 2nd edn., 2009.
8. A. C. Boggiano, C. M. Studvick, A. Steiner, J. Bacsa, I. A. Popov and H. S. La Pierre, *Chem. Sci.*, 2023, **14**, 11708-11717.
9. F. Neese, *WIREs Comput. Mol. Sci.*, 2022, **12**, e1606.
10. J. P. Perdew, M. Ernzerhof and K. Burke, *J. Chem. Phys.*, 1996, **105**, 9982-9985.
11. C. Adamo and V. Barone, *J. Chem. Phys.*, 1999, **110**, 6158-6170.
12. S. Grimme, J. Antony, S. Ehrlich and H. Krieg, *J. Chem. Phys.*, 2010, **132**, 154104.
13. S. Grimme, *J. Comput. Chem.*, 2006, **27**, 1787-1799.
14. S. Grimme, S. Ehrlich and L. Goerigk, *J. Comput. Chem.*, 2011, **32**, 1456-1465.
15. F. Neese, F. Wennmohs, A. Hansen and U. Becker, *Chem. Phys.*, 2009, **356**, 98-109.
16. R. Izsak and F. Neese, *J. Chem. Phys.*, 2011, **135**, 144105.
17. M. Garcia-Ratés and F. Neese, *J. Comput. Chem.*, 2020, **41**, 922-939.
